# Treatment with brivaracetam has no apparent long‐term effects on body weight in pediatric patients with epilepsy

**DOI:** 10.1002/epi4.13045

**Published:** 2024-10-02

**Authors:** Florin I. Floricel, Paula E. Reichel, Najla Dickson, Sofia Fleyshman, Christoph Reichel, Jan‐Peer Elshoff

**Affiliations:** ^1^ UCB Pharma Monheim am Rhein Germany; ^2^ Institute of Nutritional Medicine University of Lübeck and University Hospital Schleswig‐Holstein Lübeck Germany; ^3^ UCB Pharma Morrisville North Carolina USA

**Keywords:** Antiseizure medication, body weight, Brivaracetam, epilepsy, long‐term study

## Abstract

**Objective:**

The objective of this study is to evaluate possible long‐term effects of treatment with brivaracetam (BRV) on body weight in children with epilepsy.

**Method:**

Post hoc analysis of data from patients (aged 1 month to <17 years) with ≥6 months of BRV treatment in a long‐term, open‐label trial (N01266 [NCT01364597]). Outcomes included body weight and body mass index (BMI) over time (*z*‐score growth curves), and treatment‐emergent adverse events (TEAEs). Previous/ongoing medical conditions that may affect body weight (gastrointestinal and metabolic/nutritional disorders); concomitant antiseizure medications (ASMs) were also evaluated.

**Results:**

Two hundred nine patients (mean [standard deviation] age 7.9 [4.6] years) were analyzed. Most (154 [73.7%]) had focal‐onset seizures. At study initiation, median (range) BRV dose was 1.0 (0.4–7.5) mg/kg/day. Overall, 189 (90.4%) patients had a previous or ongoing medical condition (gastrointestinal disorders: 43 [20.6%]; metabolism/nutritional disorders: 26 [12.4%]). Most patients followed *z*‐score curves for body weight and BMI during BRV treatment, although there were outliers in both directions. Incidences of appetite/weight‐change TEAEs were low. Twenty‐three (11.0%) patients had a TEAE of decreased appetite and 14 (6.7%) had a TEAE of weight decreased.

**Significance:**

Long‐term adjunctive BRV was well tolerated in growing children with no indication of detrimental effects on body weight.

**Plain Language Summary:**

Brivaracetam is an antiseizure medication (ASM) used to treat seizures in people with epilepsy. Some ASMs can lead to changes in people's appetite and weight. Knowing the effect a drug has on appetite and weight is particularly important in children. We looked at 209 children with epilepsy taking brivaracetam and studied changes in their body weight and body mass index over time. The number of reported side effects related to appetite or weight change was low. There was no apparent long‐term effect on their body weight, even when taking their medical history and use of other ASMs into account.

## Graphical abstract

This post hoc analysis was conducted to evaluate the possible long‐term effects of treatment with brivaracetam on body weight in children with epilepsy. The results indicate that brivaracetam was well tolerated in growing children with no indication of detrimental effects on body weight.

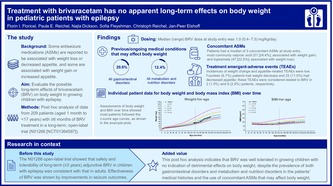

**Key Points**

- Long‐term body weight data for children with epilepsy taking adjunctive brivaracetam.
- Weight and BMI‐for‐age were plotted over time for growing children.
- No indication of detrimental effects of long‐term BRV on childrens' body weight.

## 1 INTRODUCTION

Multiple factors may affect the growth and development of children and adolescents with epilepsy, such as etiology, limitations on physical activity, the effect of comorbidities, and drug therapies.^1^ Chronic illness is a known risk factor for malnutrition, which occurs when the needs for normal body function are not met by nutritional intake.^2^ Malnutrition may contribute to the onset of seizures, or conversely epilepsy may contribute to malnutrition due to socioeconomic factors, disease severity, and use of antiseizure medications (ASMs).^3^ Children with epilepsy may be more prone to metabolic syndrome (a group of metabolic risk factors such as glucose intolerance, dyslipidemia, hypertension, and central obesity) than healthy individuals.^4^, ^5^ Some ASMs are reported to be associated with weight loss or decreased appetite, and conversely, some are associated with weight gain or increased appetite. Other ASMs have a minimal effect on weight and appetite.^1^, ^6^

Brivaracetam (BRV) is indicated for adjunctive treatment of focal‐onset (partial‐onset) seizures in patients aged ≥2 years in the European Union^7^ and as monotherapy and adjunctive treatment in patients aged ≥1 month in the United States.^8^ The long‐term safety and effectiveness of BRV in pediatric patients (aged ≥1 month to <17 years) with epilepsy was investigated in an open‐label, long‐term follow‐up trial (N01266).^9^ Enrolled patients had a mean of 3.2 patient‐years of BRV exposure, with 19.5% remaining in the trial for more than 6 years.^9^ Safety and tolerability of long‐term adjunctive BRV in pediatric patients with epilepsy was consistent with that in adults. Effectiveness was shown by improvements in seizure frequency, responder rates, and seizure freedom. This post hoc analysis of N01266 trial data was conducted to evaluate the possible long‐term effects of treatment with BRV on body weight in pediatric patients with epilepsy.

## 2 METHOD

### 2.1 Trial design

N01266 (NCT01364597)^9^ was a phase III, open‐label, single‐arm, multicenter, long‐term follow‐up trial that enrolled patients aged ≥1 month to <16 years with epilepsy who completed other BRV trials (N01263 [NCT00422422],^10^ EP0065 [NCT03405714],^11^ and N01349 [NCT03325439]^12^), and directly enrolled patients aged ≥4 to <17 years with a clinical diagnosis of focal‐onset seizures. Full details of the trial methodology have been published previously.^9^

Eligible patients from the core trials had a confirmed epilepsy diagnosis, were aged <16 years upon entry in the core trial, and were receiving treatment with at least one concomitant ASM. Eligibility criteria for direct enrollers included uncontrolled focal‐onset seizures after an adequate course of treatment with at least one ASM, and at least one focal‐onset seizure during the 3 weeks before screening. Patients were excluded if they had severe medical, neurological, or psychiatric disorders, or other medical histories that affected their safety; were hypersensitive to BRV or excipients or comparative drugs; had poor compliance with the visit schedule or medication intake in their previous trial; or had a lifetime history of suicide attempt or suicidal ideation in the past 6 months. Written informed consent was provided by the parent(s) or legal representative(s) of all patients before trial participation; assent/consent was also provided by all patients who were able to do so.

All patients enrolled from core trials must have been able to tolerate the minimum dose specified in the core trial to be eligible for entry into the evaluation period of N01266. At completion of their core trial, patients continued BRV treatment in N01266 on their existing dose. Directly enrolled patients were screened and participated in up to 3 weeks of an up‐titration period. If these patients demonstrated acceptable tolerability and seizure control on a stable daily dose of BRV (≥1 mg/kg/day) for 7 ± 2 days, they were eligible to enter the evaluation period; patients who were unable to tolerate a dose of ≥1 mg/kg/day were excluded.

BRV was administered twice daily either as tablets or an oral solution as appropriate. Dose adjustments of concomitant ASMs were permitted as needed based on clinical judgment. According to the study protocol, the maximum permitted BRV dose was 5 mg/kg/day (2.5 mg/kg twice daily) and could not exceed 200 mg/day. Planned trial participation was ≥3 years. Further inclusion and withdrawal criteria are specified in the Supporting information.

The trial was conducted in compliance with the International Council for Harmonisation‐Good Clinical Practice and the Declaration of Helsinki, and with the Health Insurance Portability and Accountability Act for US sites. The trial protocol and amendments were approved by local Institutional Review Boards/Independent Ethics Committees, as defined in local regulations.

### 2.2 Post hoc analyses

The analyses were performed for patients with ≥6 months of BRV treatment in N01266. The data were analyzed for all patients, and for subgroups split by sex, age (1 month to <2 years, 2 to <4 years, 4 to <8 years, 8 to <12 years, and ≥ 12 years), and seizure type (focal‐onset and primary generalized/unknown seizures).

The outcomes included: patient disposition, baseline characteristics, BRV dose, previous and ongoing medical conditions, concomitant ASM use assessed at BRV initiation and at 3‐monthly intervals thereafter, body weight (kg) and body mass index (BMI) assessed at 3‐monthly intervals, and treatment‐emergent adverse events (TEAEs; common TEAEs, weight‐change TEAEs, and appetite‐change TEAEs). Weight‐ and appetite‐change TEAEs did not have a reference level but were entered and used by the study investigators at their own discretion. Analyses of previous and ongoing medical conditions focused on gastrointestinal (GI) disorders and metabolism and nutrition disorders, as these conditions may affect body weight. Data for the most common medical conditions in the overall N01266 population have been reported previously.^9^

For each patient, body weight and BMI over time were plotted against *z*‐score curves which were calculated using the 2000 Centers for Disease Control and Prevention (CDC) Growth Charts for the United States as a reference.^13^, ^14^ *Z*‐scores (standard deviation [SD] scores) are used to quantify the extent that a growth metric is deviating from the median (*z* = 0). *Z*‐scores of −2 and +2 correspond to the 3rd and 97th percentiles, respectively, and represent the lower and upper bounds of a “normal” weight or BMI range (Table S1). In general, weight‐for‐age growth charts are used clinically for children up to the age of 2 years, and BMI‐for‐age is used for older children.^15^, ^16^ However, there is evidence that BMI may be an appropriate indicator of growth even below the age of 2 years.^17^ We provided BMI‐for‐age for the whole age range to facilitate review of body weight evolution over time and inter‐patient comparison.

## 3 RESULTS

### 3.1 Patients

A total of 257 patients received at least one dose of BRV, of whom 209 had ≥6 months BRV exposure and were included in the current analyses (Table 1). One hundred twenty‐four (59.3%) patients completed the trial. The most common reasons for discontinuation were lack of efficacy (9.6%) and adverse events (9.1%). At study entry, the median (range) BRV dose was 1.0 (0.4–7.5) mg/kg/day (*n* = 209) and at 12 months it was 4.0 (0.6–7.5) mg/kg/day (*n* = 184). The median dose was generally stable thereafter.

**TABLE 1:** Patient disposition and discontinuations (SS).

	Overall population									Seizure type subgroup	
	All (*N* = 209)	Sex		Age group							
		Male (*n* = 113)	Female (*n* = 96)	1 mo to <1 year (*n* = 13)	1 to <2 years (*n* = 19)	2 to <4 years (*n* = 11)	4 to <8 years (*n* = 61)	8 to <12 years (*n* = 52)	≥12 years (*n* = 53)	Focal‐onset (*n* = 154)	Primary generalized/unknown (*n* = 55)
Completed, *n* (%)	124 (59.3)	74 (65.5)	50 (52.1)	6 (46.2)	12 (63.2)	4 (36.4)	37 (60.7)	28 (53.8)	37 (69.8)	94 (61.0)	30 (54.5)
Discontinued, *n* (%)	85 (40.7)	39 (34.5)	46 (47.9)	7 (53.8)	7 (36.8)	7 (63.6)	24 (39.3)	24 (46.2)	16 (30.2)	60 (39.0)	25 (45.5)
Primary reasons for discontinuation, *n* (%)											
Lack of efficacy	20 (9.6)	10 (8.8)	10 (10.4)	2 (15.4)	2 (10.5)	2 (18.2)	4 (6.6)	5 (9.6)	5 (9.4)	14 (9.1)	6 (10.9)
Adverse event	19 (9.1)	9 (8)	10 (10.4)	1 (7.7)	2 (10.5)	3 (27.3)	3 (4.9)	7 (13.5)	3 (5.7)	12 (7.8)	7 (12.7)
Other	19 (9.1)	7 (6.2)	12 (12.5)	2 (15.4)	2 (10.5)	1 (9.1)	6 (9.8)	5 (9.6)	3 (5.7)	15 (9.7)	4 (7.3)
Withdrawal by patient	17 (8.1)	8 (7.1)	9 (9.4)	2 (15.4)	1 (5.3)	1 (9.1)	7 (11.5)	5 (9.6)	1 (1.9)	10 (6.5)	7 (12.7)
Lost to follow‐up	8 (3.8)	3 (2.7)	5 (5.2)	0	0	0	4 (6.6)	1 (1.9)	3 (5.7)	7 (4.5)	1 (1.8)
Protocol deviation	1 (0.5)	1 (0.9)	0	0	0	0	0	1 (1.9)	0	1 (0.6)	0
Unknown	1 (0.5)	1 (0.9)	0	0	0	0	0	0	1 (1.9)	1 (0.6)	0

Abbreviations: mo, month; SS, safety set.

At baseline, patients had a mean (SD) age of 7.9 (4.6) years, and most were aged ≥4 years (Table 2). The median (Q1, Q3) number of previous ASMs was 2.0 (0.0, 4.0), and most patients (154 [73.7%]) had focal‐onset seizures. Overall, 189 (90.4%) patients had a previous or ongoing medical condition (Table 2). GI disorders were reported in 43 (20.6%) patients and were more common in patients aged <4 years (1 month to <1 year [30.8%], 1 to <2 years [31.6%], 2 to <4 years [63.6%]) than in older patients (4 to <8 years [19.7%], 8 to <12 years [15.4%], ≥12 years [11.3%]). Overall, the most common GI disorders were gastroesophageal reflux disease (17 [8.1%]) and constipation (16 [7.7%]). Metabolism and nutrition disorders were reported in 26 (12.4%) patients, most commonly feeding disorder (four [1.9%]), obesity (four [1.9%]), decreased appetite (three [1.4%]), and failure to thrive (three [1.4%]). Malnutrition, overweight, underweight, and poor weight gain were each reported by one (0.5%) patient.

**TABLE 2:** Baseline demographics, epilepsy characteristics, and previous and ongoing medical conditions that may influence body weight (SS).

	Overall population									Seizure type subgroup	
	All (*N* = 209)	Sex		Age group							
		Male (*n* = 113)	Female (*n* = 96)	1 mo to <1 year (*n* = 13)	1 to <2 years (*n* = 19)	2 to <4 years (*n* = 11)	4 to <8 years (*n* = 61)	8 to <12 years (*n* = 52)	≥12 years (*n* = 53)	Focal‐onset (*n* = 154)	Primary generalized/unknown (*n* = 55)
Age, mean (SD), years^a^	7.9 (4.6)	8.0 (4.3)	7.8 (4.8)	0.7 (0.2)	1.4 (0.4)	2.7 (0.6)	5.7 (1.1)	10.0 (1.1)	13.7 (1.2)	8.6 (4.3)	6.1 (4.7)
Body weight, mean (SD), kg^a^	30.7 (21.1)	30.6 (18.5)	30.9 (23.9)	6.9 (2.0)	9.9 (2.0)	13.4 (2.5)	21.4 (6.3)	33.9 (11.4)	55.2 (22.6)	33.2 (21.0)	23.9 (20.0)
BMI, mean (SD), kg/m^2^	18.1 (5.0)	17.9 (4.2)	18.3 (5.8)	16.5 (2.7)	16.1 (2.2)	16.8 (2.4)	16.3 (3.2)	17.5 (3.8)	21.9 (6.9)	18.4 (5.2)	17.1 (4.3)
Racial group, *n* (%)											
White	156 (74.6)	82 (72.6)	74 (77.1)	10 (76.9)	15 (78.9)	11 (100.0)	44 (72.1)	38 (73.1)	38 (71.7)	107 (69.5)	49 (89.1)
Other	50 (23.9)	29 (25.7)	21 (21.9)	3 (23.1)	4 (21.1)	0	16 (26.2)	13 (25.0)	14 (26.4)	44 (28.6)	6 (10.9)
Black	3 (1.4)	2 (1.8)	1 (1.0)	0	0	0	1 (1.6)	1 (1.9)	1 (1.9)	3 (1.9)	0
Epilepsy history											
Duration of epilepsy, mean (SD), years^b^	4.7 (3.7)^d^	5.1 (3.8)	4.2 (3.6)^e^	0.4 (0.3)^f^	0.8 (0.5)	2.0 (0.9)	3.4 (1.7)	6.0 (3.0)	7.9 (4.1)	5.0 (3.8)	3.9 (3.5)^g^
Age at diagnosis, mean (SD), years	3.3 (3.3)^d^	3.0 (3.1)	3.7 (3.6)^e^	0.2 (0.3)^f^	0.7 (0.5)	0.8 (0.9)	2.4 (1.8)	4.0 (3.2)	5.9 (4.0)	3.7 (3.3)	2.4 (3.2)^g^
Percentage of life with epilepsy, mean (SD), %^c^	59.4(29.3)^d^	64.1 (28.9)	53.8 (28.8)^e^	65.3 (32.7)^f^	53.3 (30.2)	71.2 (30.4)	58.4 (28.3)	61.1 (29.7)	57.2 (28.9)	58.1 (29.5)	63.1 (28.6)^g^
Number of previous ASMs^h^											
Median (Q1–Q3)	2 (0–4)	2 (1–3)	1 (0–4)	1 (0–2)	1 (0–2)	1 (0–5)	2 (0–3)	1.5 (0–4)	3 (1–4)	2 (1–4)	1 (0–3)
0 to 1, *n* (%)	102 (48.8)	51 (45.1)	51 (53.1)	9 (69.2)	14 (73.7)	6 (54.5)	25 (41.0)	26 (50.0)	22 (41.5)	69 (44.8)	33 (60.0)
2 to 3, *n* (%)	54 (25.8)	35 (31.0)	19 (19.8)	4 (30.8)	4 (21.1)	1 (9.1)	22 (36.1)	10 (19.2)	13 (24.5)	43 (27.9)	11 (20.0)
≥4, *n* (%)	53 (25.4)	27 (23.9)	26 (27.1)	0	1 (5.3)	4 (36.4)	14 (23.0)	16 (30.8)	18 (34.0)	42 (27.3)	11 (20.0)
Previous and ongoing medical conditions											
Any previous and ongoing conditions, *n* (%)^i^	189 (90.4)	104 (92.0)	85 (88.5)	10(76.9)	18 (94.7)	11 (100)	55 (90.2)	44 (84.6)	51 (96.2)	138 (89.6)	51 (92.7)
Gastrointestinal disorders											
Any gastrointestinal disorder, *n* (%)^j^	43 (20.6)	22 (19.5)	21 (21.9)	4 (30.8)	6 (31.6)	7 (63.6)	12 (19.7)	8 (15.4)	6 (11.3)	28 (18.2)	15 (27.3)
Gastrointestinal disorders reported by ≥5% of all patients, *n* (%)											
Gastroesophageal reflux disease	17 (8.1)	8 (7.1)	9 (9.4)	2 (15.4)	3 (15.8)	4 (36.4)	6 (9.8)	1 (1.9)	1 (1.9)	9 (5.8)	8 (14.5)
Constipation	16 (7.7)	8 (7.1)	8 (8.3)	0	2 (10.5)	4 (36.4)	4 (6.6)	3 (5.8)	3 (5.7)	9 (5.8)	7 (12.7)
Metabolism and nutrition disorders											
Any metabolism and nutrition disorder, *n* (%)^j^	26 (12.4)	13 (11.5)	13 (13.5)	1 (7.7)	1 (5.3)	6 (54.5)	7 (11.5)	4 (7.7)	7 (13.2)	18 (11.7)	8 (14.5)
Metabolism and nutrition disorders reported by ≥1% of all patients, *n* (%)											
Feeding disorder	4 (1.9)	4 (3.5)	0	1 (7.7)	0	1 (9.1)	1 (1.6)	1 (1.9)	0	2 (1.3)	2 (3.6)
Obesity	4 (1.9)	2 (1.8)	2 (2.1)	0	0	1 (9.1)	1 (1.6)	0	2 (3.8)	2 (1.3)	2 (3.6)
Decreased appetite	3 (1.4)	0	3 (3.1)	0	0	0	2 (3.3)	1 (1.9)	0	1 (0.6)	2 (3.6)
Failure to thrive	3 (1.4)	2 (1.8)	1 (1.0)	0	0	1 (9.1)	2 (3.3)	0	0	2 (1.3)	1 (1.8)
Metabolic acidosis	2 (1.0)	0	2 (2.1)	0	0	0	1 (1.6)	1 (1.9)	0	1 (0.6)	1 (1.8)

Abbreviations: ASM, antiseizure medication; BMI, body mass index; BRV, brivaracetam; mo, month; SD, standard deviation; SS, safety set.

^a^ At BRV initiation in N01266.

^b^ Relative to date of diagnosis.

^c^ Percentage of life with epilepsy is calculated as epilepsy duration based on first diagnosis divided by age at informed consent * 100.

^d^ *n* = 208.

^e^ *n* = 95.

^f^ *n* = 12.

^g^ *n* = 54.

^h^ Previous ASMs are those taken before BRV and which stopped before the date of first study dose in N01266.

^i^ Includes both resolved and ongoing medical conditions at the day of first study dose in N01266.

^j^ System Organ Class (Medical Dictionary for Regulatory Activities, Version 18.1).

### 3.2 Concomitant ASM use during BRV treatment

**FIGURE 1:**
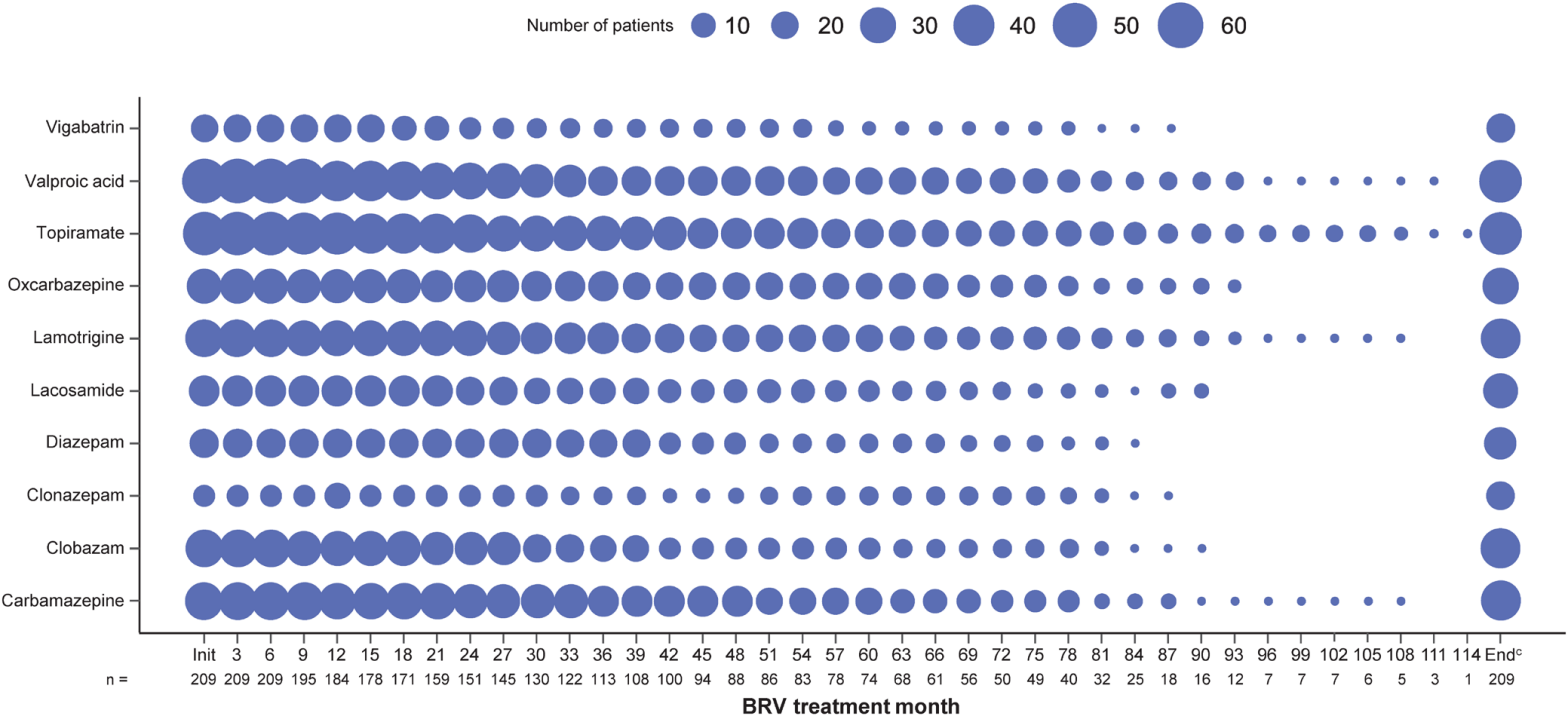
Ten most commonly used concomitant ASMs during the BRV treatment period (SS).^a,b^ ASM, antiseizure medication; BRV, brivaracetam; SS, safety set. ^a^Figure shows the number of patients at each time point, not percentage values. ^b^The ten most common concomitant ASMs in patients with ≥6 months of BRV exposure. ^c^‘End’ is defined as the final visit in the study, irrespective of when in the study that visit would have taken place.

Patients had a median of two concomitant ASMs at study entry (*n* = 209) and at all 3‐month time points up to month 78 (*n* = 40). The median number of concomitant ASMs generally decreased thereafter. Valproic acid and topiramate were the most common concomitant ASMs at study entry (Table 3) and at all assessed time points up to month 33, after which generally the most common concomitant ASMs were valproic acid, topiramate, lamotrigine, and carbamazepine (Figure 1).

**TABLE 3:** Common concomitant ASMs at study entry (SS).^a^ ^,^ ^b^

	Overall population									Seizure type subgroup	
	All (*N* = 209)	Sex		Age group							
		Male (*n* = 113)	Female (*n* = 96)	1 mo to <1 year (*n* = 13)	1 to <2 years (*n* = 19)	2 to <4 years (*n* = 11)	4 to <8 years (*n* = 61)	8 to <12 years (*n* = 52)	≥12 years (*n* = 53)	Focal‐onset (*n* = 154)	Primary generalized/unknown (*n* = 55)
Most common concomitant ASMs (ten most commonly used ASMs in the overall population, and in the seizure type subgroups), *n* (%)^c^, ^d^, ^e^											
Valproic acid	51 (24.4)	22 (19.5)	29 (30.2)	2 (15.4)	10 (52.6)	1 (9.1)	15 (24.6)	9 (17.3)	14 (26.4)	23 (14.9)	28 (50.9)
Topiramate	47 (22.5)	27 (23.9)	20 (20.8)	4 (30.8)	6 (31.6)	1 (9.1)	16 (26.2)	11 (21.2)	9 (17.0)	35 (22.7)	12 (21.8)
Carbamazepine	37 (17.7)	16 (14.2)	21 (21.9)	1 (7.7)	2 (10.5)	1 (9.1)	11 (18.0)	13 (25.0)	9 (17.0)	37 (24.0)	–
Clobazam	34 (16.3)	20 (17.7)	14 (14.6)	1 (7.7)	3 (15.8)	4 (36.4)	9 (14.8)	7 (13.5)	10 (18.9)	22 (14.3)	12 (21.8)
Lamotrigine	34 (16.3)	21 (18.6)	13 (13.5)	0	1 (5.3)	0	12 (19.7)	5 (9.6)	16 (30.2)	27 (17.5)	7 (12.7)
Oxcarbazepine	27 (12.9)	13 (11.5)	14 (14.6)	0	0	1 (9.1)	12 (19.7)	6 (11.5)	8 (15.1)	26 (16.9)	–
Lacosamide	20 (9.6)	12 (10.6)	8 (8.3)	1 (7.7)	0	1 (9.1)	5 (8.2)	5 (9.6)	8 (15.1)	16 (10.4)	–
Diazepam	19 (9.1)	13 (11.5)	6 (6.3)	0	0	1 (9.1)	5 (8.2)	6 (11.5)	7 (13.2)	18 (11.7)	1 (1.8)
Vigabatrin	15 (7.2)	8 (7.1)	7 (7.3)	2 (15.4)	5 (26.3)	2 (18.2)	4 (6.6)	2 (3.8)	0	–	7 (12.7)
Clonazepam	10 (4.8)	6 (5.3)	4 (4.2)	0	0	0	3 (4.9)	4 (7.7)	3 (5.7)	9 (5.8)	–
Valproate magnesium	–	–	–	–	–	–	–	–	–	18 (11.7)	–
Phenobarbital	–	–	–	–	–	–	–	–	–	–	9 (16.4)
Ergenyl chrono	–	–	–	–	–	–	–	–	–	–	6 (10.9)
Ethosuximide	–	–	–	–	–	–	–	–	–	–	5 (9.1)
Valproate sodium	–	–	–	–	–	–	–	–	–	–	4 (7.3)

Abbreviations: ASM, antiseizure medication; BRV, brivaracetam; mo, month; SS, safety set.

^a^ Concomitant ASMs are those taken before, and still ongoing at study entry.

^b^ Study entry corresponds to date of first study dose in N01266.

^c^ WHODD SEP/2017 Preferred Drug Name.

^d^ Each patient is counted at most once for each Preferred Drug Name.

^e^ A dash is included in the table where the ASM was not one of the ten most commonly used ASMs in the relevant population.

### 3.3 Body weight and BMI over time

Most patients followed the *z*‐score curves for body weight and BMI, although there were outliers in both directions. There were no trends observed with regards to weight decrease or increase. A full set of plots (weight‐for‐age and BMI‐for‐age) for each patient in these analyses are available in Supporting information.

### 3.4 Incidence of TEAEs over time

**FIGURE 2:**
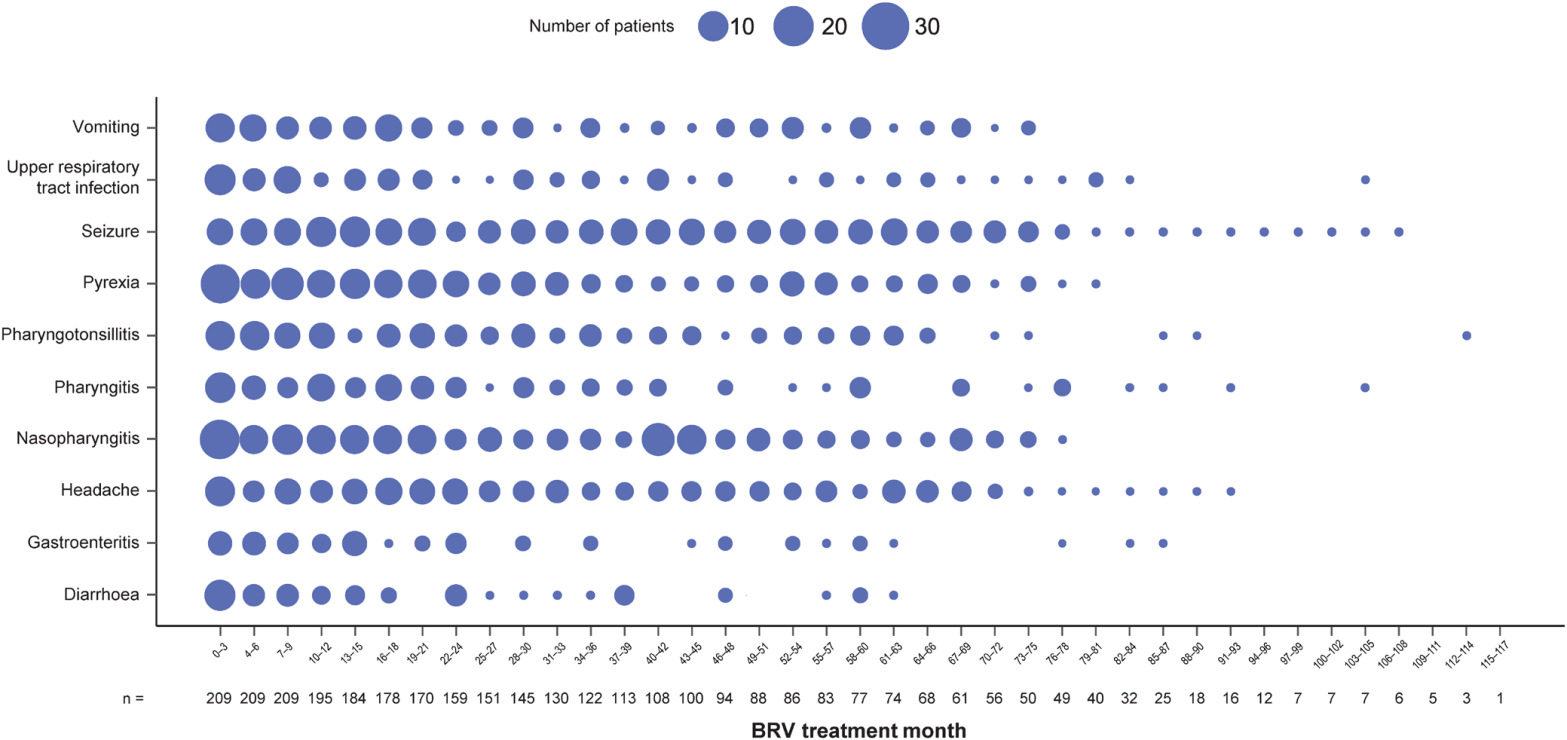
Incidence of ten most common TEAEs during the BRV treatment period (SS).^a,b^ BRV, brivaracetam; SS, safety set; TEAE, treatment‐emergent adverse event. ^a^Figure presents the number of patients reporting TEAEs with a start date within the specified 3‐month time interval. Patients were included in a 3‐month interval if they were receiving BRV at any time during that interval. One month was defined as 30 days. ^b^The ten most common TEAEs in patients with ≥6 months of BRV exposure.

During the overall BRV treatment period, the ten most common TEAEs in patients with ≥6 months BRV exposure were nasopharyngitis (74 [35.4%]), pyrexia (58 [27.8%]), pharyngitis (54 [25.8%]), vomiting (49 [23.4%]), upper respiratory tract infection (40 [19.1%]), headache (37 [17.7%]), seizure (37 [17.7%]), pharyngotonsillitis (35 [16.7%]), diarrhea (34 [16.3%]), and gastroenteritis (31 [14.8%]) (Figure 2).

**FIGURE 3:**
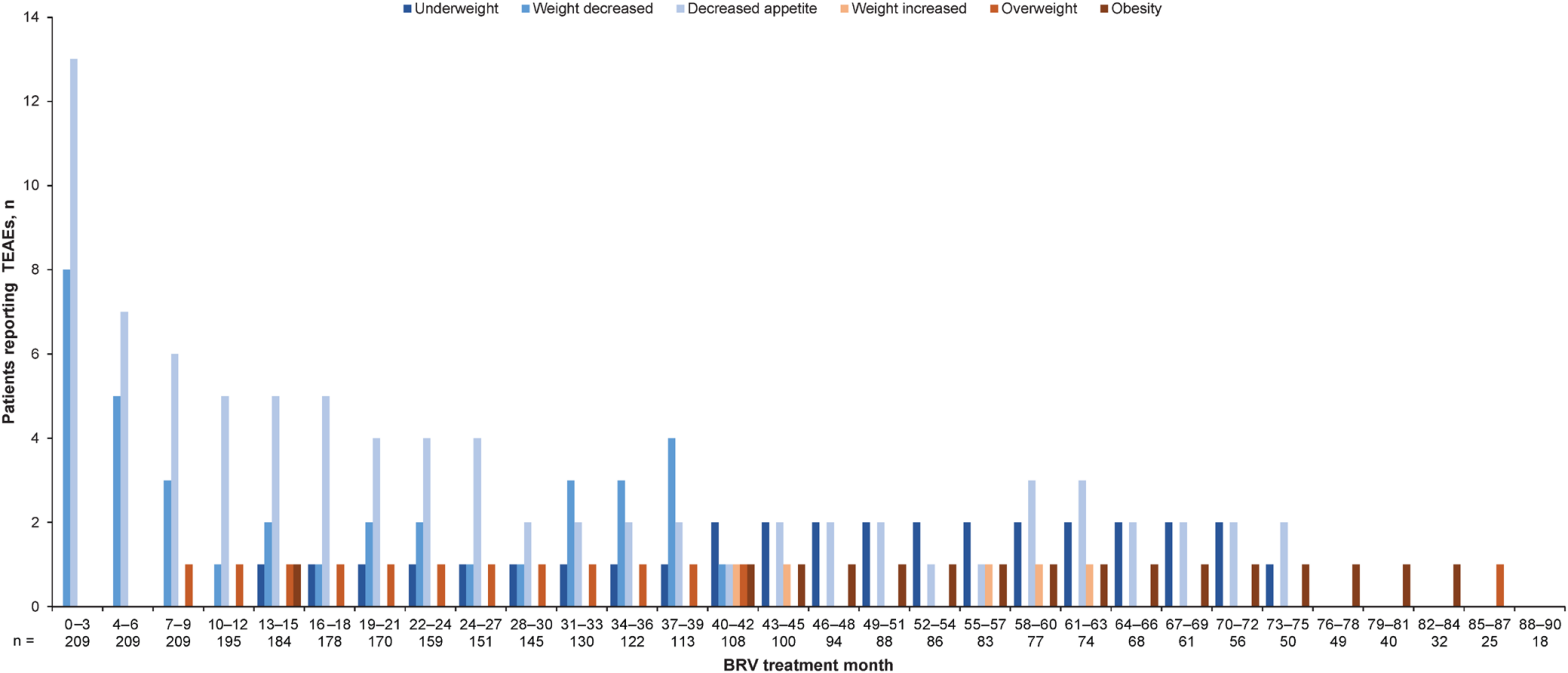
Incidence of weight change and appetite–related TEAEs during the BRV treatment period (SS).^a^ BRV, brivaracetam; SS, safety set; TEAE, treatment‐emergent adverse event. ^a^Figure presents the number of patients reporting TEAEs with a start date within the specified 3‐month time interval. Patients were included in a 3‐month interval if they were receiving BRV at any time during that interval. One month was defined as 30 days. No weight change or appetite–related TEAEs were reported after month 87.

Two (1.0%) patients had a TEAE of underweight. This TEAE was reported by one patient between months 13 and 72, and one patient between months 39 and 75 (Figure 3). Both patients had a diagnosis of primary generalized seizures, one was female (age group: ≥1 month to <2 years) and the other male (≥2 to <4 years). In both cases, the TEAE was continuous, moderate in intensity, and not considered to be related to BRV (Investigator's opinion). In one patient, the TEAE was serious.

A total of 18 TEAEs of weight decreased were reported in 14 (6.7%) patients. This TEAE was most common in the first 6 months of treatment (months 0 to 3: eight patients; months 4 to 6: five patients) and incidence generally decreased thereafter; no patients reported weight decrease after month 42 (Figure 3). Three patients with a TEAE of weight decreased had a diagnosis of primary generalized seizures, and 11 had focal‐onset seizures; seven were female (age group: ≥1 month to <2 years, two; ≥4 to <12 years, four; ≥12 to <17 years, one) and seven were male (age group: ≥1 month to <2 years, one; ≥4 to <12 years, five; ≥12 to <17 years, one). In most patients, “weight decrease” was continuous and mild or moderate in intensity. This TEAE was considered related to BRV (Investigator's opinion) in two patients, serious in one patient, and led to discontinuation in one patient.

Decreased appetite was reported in 23 (11.0%) patients (total of 28 TEAEs). In six (2.9%) patients, the TEAE of decreased appetite was considered related to BRV. Decreased appetite was reported in 13 patients in months 0–3, seven patients in months 4–6, and six patients in months 7–9. The incidence of this TEAE generally decreased thereafter, with three or fewer patients reporting this TEAE from month 28 onwards (Figure 3). Seven patients with a TEAE of decreased appetite had a diagnosis of primary generalized seizures, 15 patients had focal‐onset seizures, and one patient was uncategorized; 11 were female (age group: ≥1 month to <2 years, two; ≥4 to <12 years, six; ≥12 to <17 years, three) and 12 were male (age group: ≥1 month to <2 years, four; ≥2 to <4 years, one; ≥4 to <12 years, four; ≥12 to <17 years, three). One patient had a serious TEAE of decreased appetite, and no patients discontinued due to this TEAE. Five patients reported both weight decrease and decreased appetite (none had TEAEs of underweight).

Two (1.0%) patients had a TEAE of weight increased; this TEAE was reported in one patient in each 3‐month interval between months 40 and 45, and in one patient between months 55 and 63 (Figure 3). Both patients were male (age group: ≥12 to <17 years); one had a diagnosis of primary generalized seizures and one had focal‐onset seizures. In both cases, the TEAE of weight increased was mild in intensity, not serious, and not considered related to BRV (Investigator's opinion).

Two (1.0%) patients had a TEAE of overweight; this TEAE was reported in one patient in each 3‐month interval between months 7 and 42, and in the other patient between months 85 and 87 (Figure 3). Both patients were male (age group: ≥4 to <12 years) and had a diagnosis of focal‐onset seizures. In both cases, the TEAE of overweight was not considered to be serious or related to BRV (Investigator's opinion). The TEAE was mild in one patient (with TEAE onset 21 days after the last dose of BRV) and moderate in the other.

Obesity was reported in two (1.0%) patients; this TEAE was reported in one patient between months 13 and 15, and in one patient in each 3‐month interval between months 40 and 84 (Figure 3). Both patients had a diagnosis of focal‐onset seizures; one was female (age group: ≥12 to <17 years) and the other was male (≥4 to <12 years). In both patients, the TEAE of obesity was continuous, mild in intensity, not serious, and not considered related to BRV (Investigator's opinion). No patients reported more than one kind of TEAE related to weight increase (obesity, weight increased and/or overweight). One patient had TEAEs of both weight decreased and overweight; however, these events occurred approximately 4 years apart.

## 4 DISCUSSION

To our knowledge, this open‐label trial of BRV in pediatric patients with epilepsy represents the longest prospective study of body weight evolution in a pediatric population under a particular ASM treatment to date. This post hoc analysis assessed the effects of long‐term BRV treatment on body weight in a subgroup of patients from N01266 who had ≥6 months of BRV exposure. Overall, ~60% of patients completed the study; these patients maintained BRV treatment long term with no indication of detrimental effects on body weight.

Most patients were aged ≥4 years and had focal‐onset seizures. Before initiating BRV, 51.2% had been treated with two or more ASMs. Overall, 20.6% of patients had previous/ongoing GI disorders and 12.4% had metabolism and nutritional disorders which may have affected growth. The prevalence of GI disorders was as expected for a pediatric population,^18^, ^19^, ^20^ with the highest prevalence seen in patients aged <4 years.

Patients were receiving a median of two concomitant ASMs in addition to BRV. As concomitant ASM use may affect body weight, any weight changes cannot be solely attributed to BRV. Valproic acid and topiramate were the most common concomitant ASMs at most time points. Valproic acid has been associated with increased appetite and weight gain,^21^, ^22^ and topiramate has a known side effect of weight decrease.^1^

Assessments of body weight and BMI over time showed that most patients followed the *z*‐score age curves, although there were outliers in both directions. Although N01266 was an international trial (Belgium, Czech Republic, Germany, Hungary, Italy, Mexico, Poland, Spain, and the United States^9^), the post hoc analysis of BMI and body weight used the 2000 CDC Growth Charts for the United States. The CDC charts show the growth patterns of typical children in the United States and may not be fully representative of growth patterns in children from other countries^15^; however, they are more appropriate for the current analyses than the World Health Organization growth standards, which describe the growth of healthy breastfed children under optimal conditions.^15^ Body weight and BMI have known limitations as biomarkers for child development and, ideally, other metrics (such as body composition, body mass, fat mass) would be used to fully evaluate whether growth is adequate.^23^, ^24^, ^25^

In line with the data for the overall N01266 trial population,^9^ many of the common TEAEs in patients with >6 months of BRV exposure were infections (e.g., nasopharyngitis, pharyngitis, and upper respiratory tract infection). Incidences of these TEAEs were as expected for a pediatric population. Such conditions may affect growth, although not to the same extent as chronic infections, as the effect should typically be transient. Most weight‐related TEAEs reported during BRV treatment were associated with a decrease (underweight, weight decreased, decreased appetite) rather than an increase in weight (weight increased, overweight, obesity). These TEAEs did not appear to influence body weight evolution over time, as most patients either followed the same percentile or slightly improved. Incidences of weight decrease and decreased appetite TEAEs were highest in the first 18 months of BRV treatment, during which time patients were younger; however, it should be noted that the number of patients included in the analyses decreased over time, as patients discontinued. In the overall population in the N01266 trial, 30 (11.7%) patients had a TEAE of decreased appetite. This TEAE was drug‐related (Investigator's opinion) in 11 (4.3%) patients. Decreased appetite was also reported as a common drug‐related TEAE in one of the core trials that preceded N01266 (N01263). In a 3‐week open‐label study of adjunctive BRV in pediatric patients (aged 1 month to <16 years) 6.1% of patients had decreased appetite. One patient discontinued BRV due to this TEAE.^10^

A limitation of this study is that it is a post hoc analysis of an open‐label trial with no comparator group. This analysis focused on data from patients with ≥6 months of BRV treatment, as a shorter time period would not enable conclusions to be drawn about long‐term exposure; however, this may have introduced a potential bias, as patients who were less able to tolerate BRV may have discontinued in the first few months of treatment. Patients were also taking concomitant ASMs, as well as other medications, in addition to BRV, which may also have an influence on body weight. BMI‐for‐age was analyzed for patients aged <2 years; BMI is not a recommended measure for this age group despite evidence that it is a useful indicator of growth.^17^ Lastly, most patients were aged ≥4 years and had focal‐onset seizures, therefore younger children or those with different seizure types may be under‐represented in this study.

## 5 CONCLUSION

The results of this post hoc analysis indicate that BRV was well tolerated in growing children with no indication of detrimental effects on body weight, despite the prevalence of both GI disorders and metabolism and nutrition disorders in the patients' medical histories and the use of concomitant ASMs and other medications that may affect body weight.

## AUTHOR CONTRIBUTIONS

All authors made substantial contributions to study conception/design, or acquisition/analysis/interpretation of data; and drafting of the manuscript, or revising it critically for important intellectual content. All authors provided final approval of the manuscript.

## CONFLICT OF INTEREST STATEMENT

F. Floricel, N. Dickson, C. Reichel, and J‐P. Elshoff are employees of UCB Pharma. S. Fleyshman was an employee of UCB Pharma at the time of the study and is now an employee of Merck. P. Reichel was a consultant for UCB Pharma at the time of the study and is now affiliated with the University of Michigan Department of Pathology. We confirm that we have read the Journal's position on issues involved in ethical publication and affirm that this report is consistent with those guidelines.

## Supporting information


Data S1.

Data S2.

Table S1.

## Data Availability

Underlying data from this manuscript may be requested by qualified researchers 6 months after product approval in the United States and/or Europe, or global development is discontinued, and 18 months after trial completion. Investigators may request access to anonymized individual patient‐level data and redacted trial documents which may include: analysis‐ready datasets, study protocol, annotated case report form, statistical analysis plan, dataset specifications, and clinical study report. Prior to use of the data, proposals need to be approved by an independent review panel at www.Vivli.org and a signed data sharing agreement will need to be executed. All documents are available in English only, for a pre‐specified time, typically 12 months, on a password protected portal.
