# Supplementary material for: Treatment with brivaracetam has no apparent long‐term effects on body weight in pediatric patients with epilepsy
Source: Epilepsia Open. 2024 Oct 2;9(6):2230–40. doi: 10.1002/epi4.13045 (PMC11633704; doi:10.1002/epi4.13045)

Treatment with brivaracetam has no apparent long-term effects on body weight in pediatric patients with epilepsy

Florin I. Floricel | Paula E. Reichel | Najla Dickson | Sofia Fleyshman | Christoph Reichel | Jan-Peer Elshoff

Individual patient data for weight and BMI over time

Vertical line indicates 6 months of exposure to BRV on the N01266 study.

Abbreviations: BMI, body mass index; BRV, brivaracetam.

Patient Number: 1
Seizure History: Partial Onset Seizures

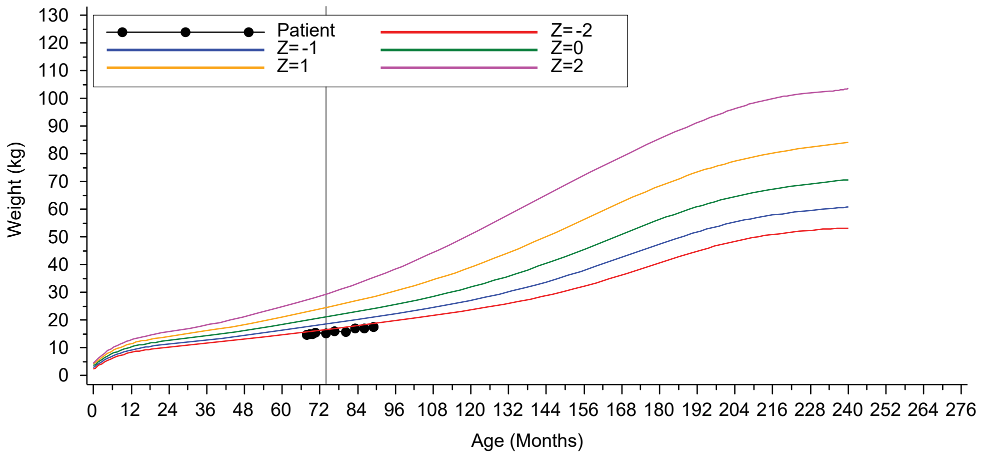

Patient Number: 1
Seizure History: Partial Onset Seizures

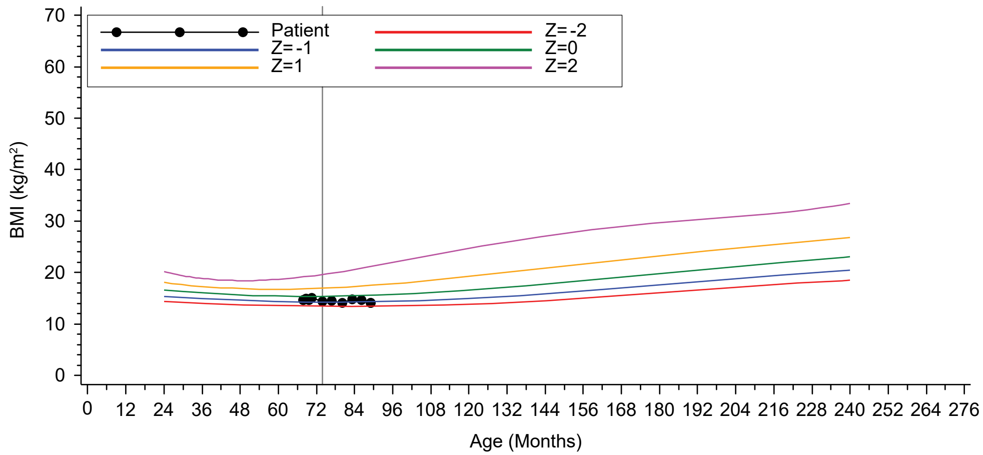

Patient Number: 2
Seizure History: Partial Onset Seizures

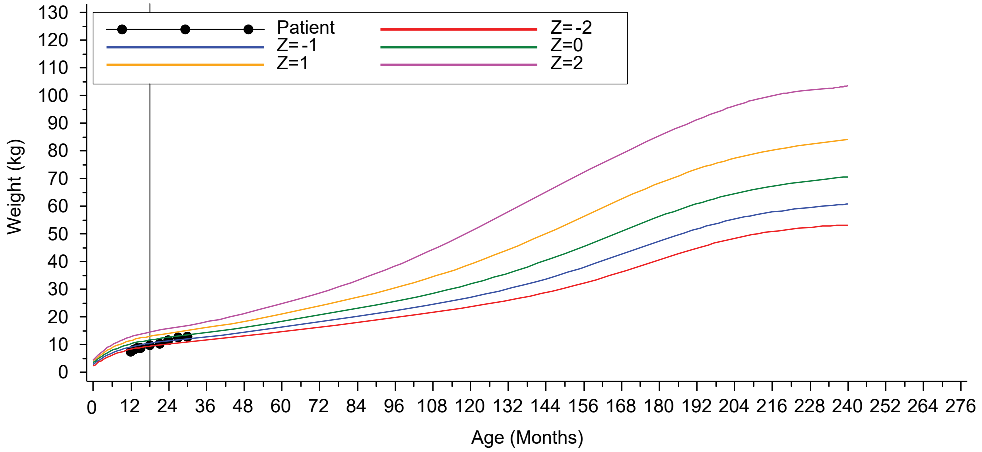

Patient Number: 2
Seizure History: Partial Onset Seizures

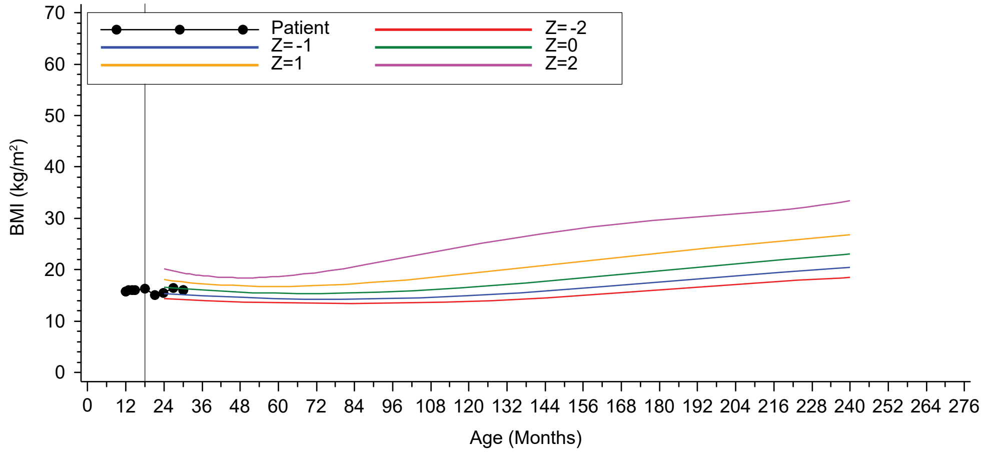

Patient 3
Seizure History: Partial Onset Seizures

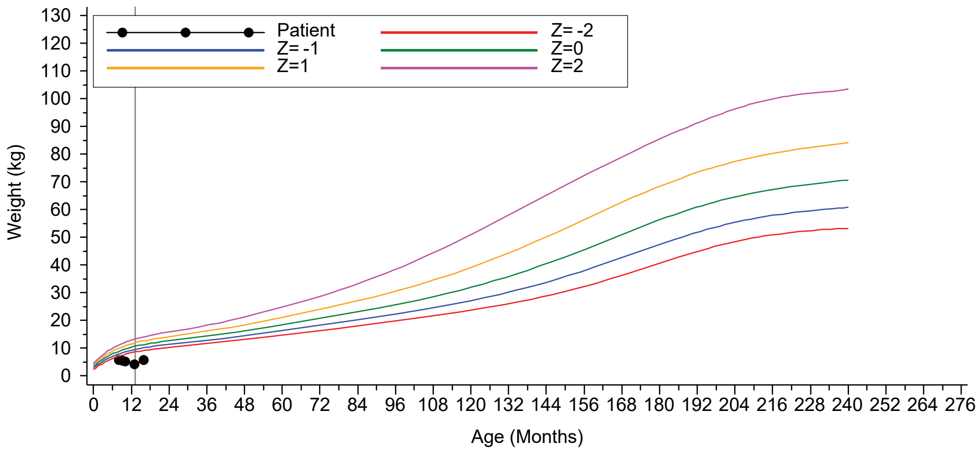

Patient 3
Seizure History: Partial Onset Seizures

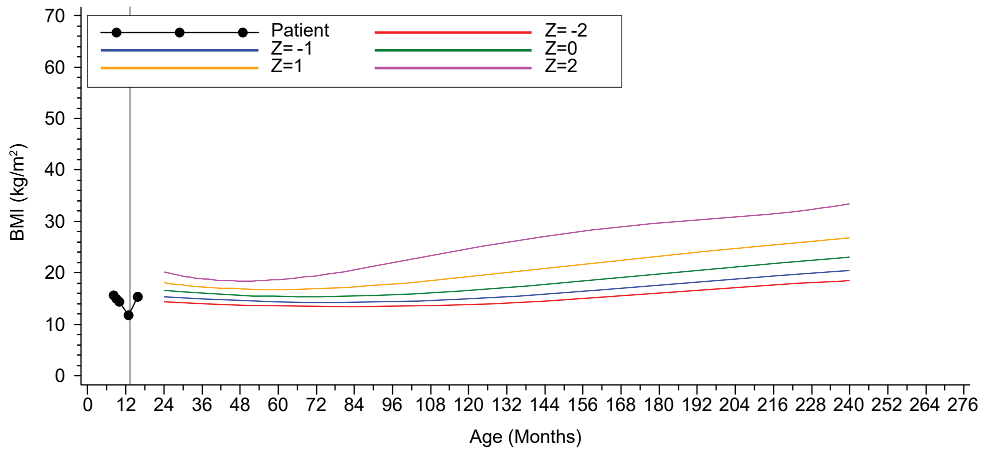

Patient 4
Seizure History: Partial Onset Seizures

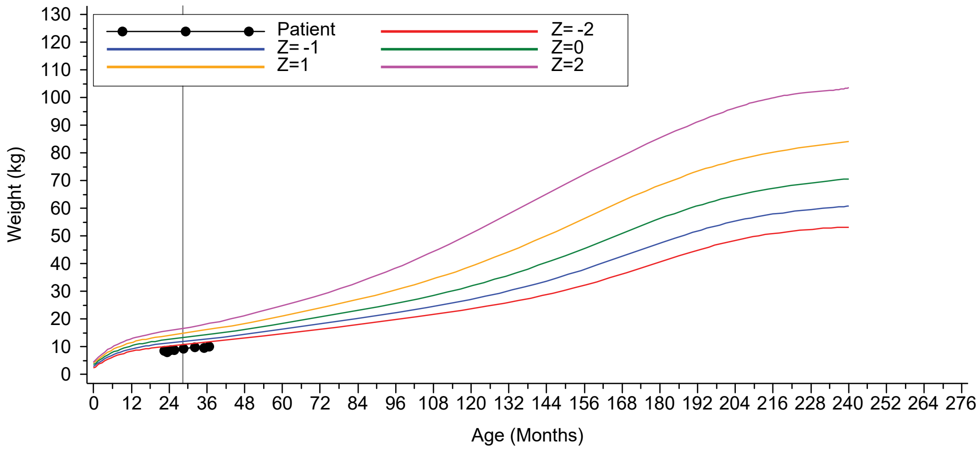

Patient 4
Seizure History: Partial Onset Seizures

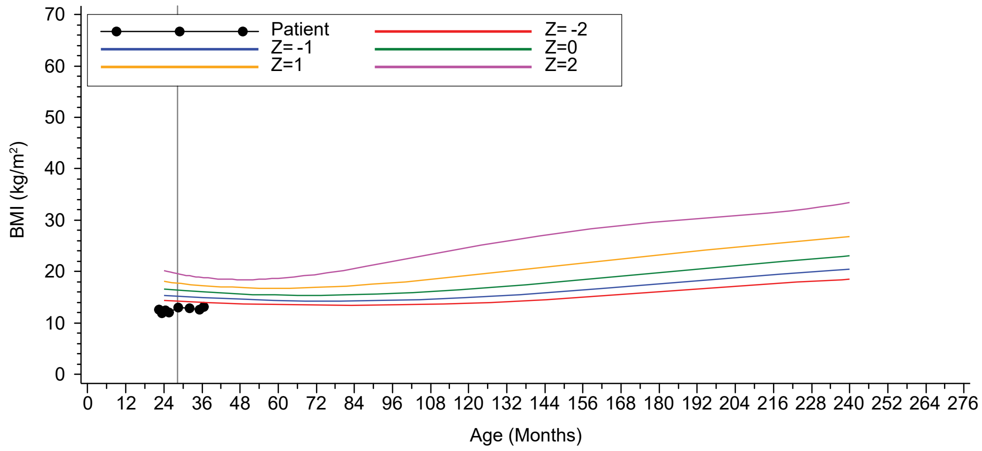

Patient 5
Seizure History: Partial Onset Seizures

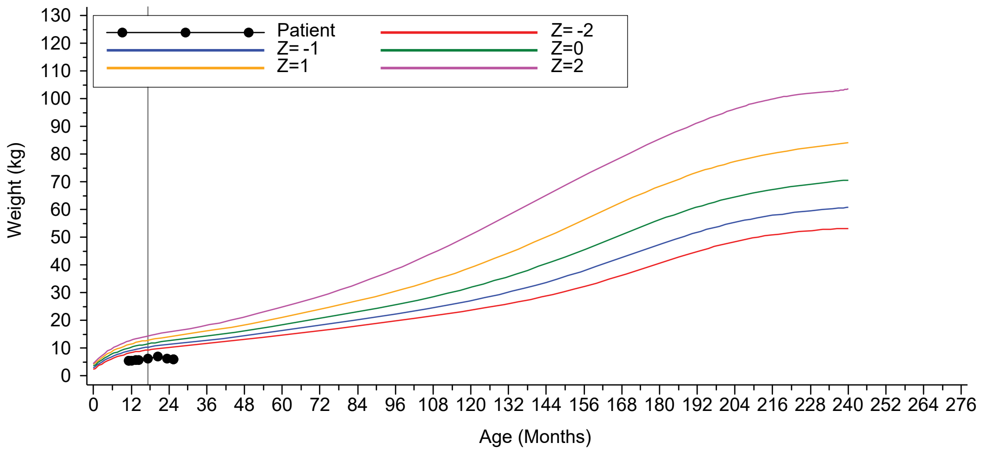

Patient 5
Seizure History: Partial Onset Seizures

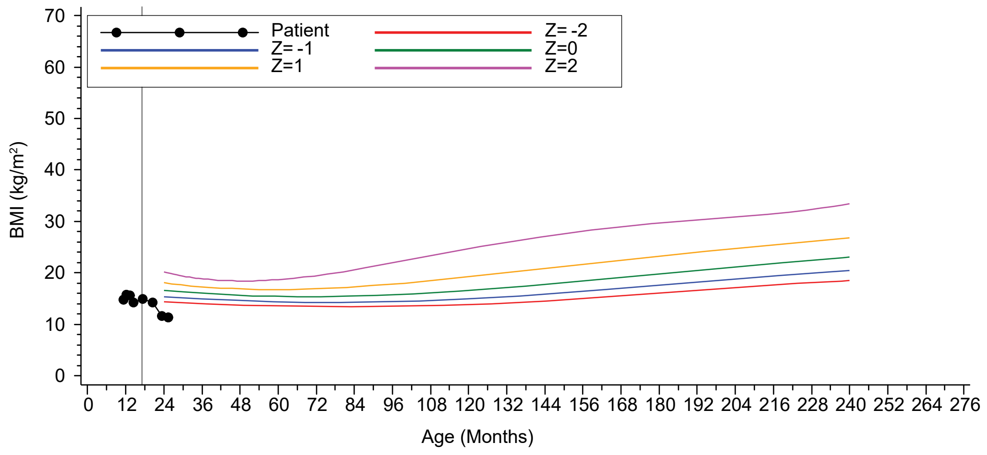

Patient 6
Seizure History: Partial Onset Seizures

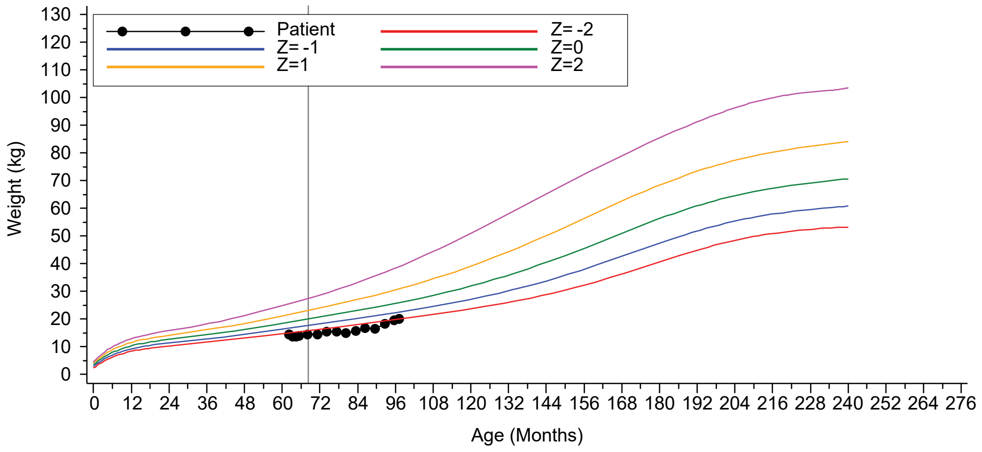

Patient 6
Seizure History: Partial Onset Seizures

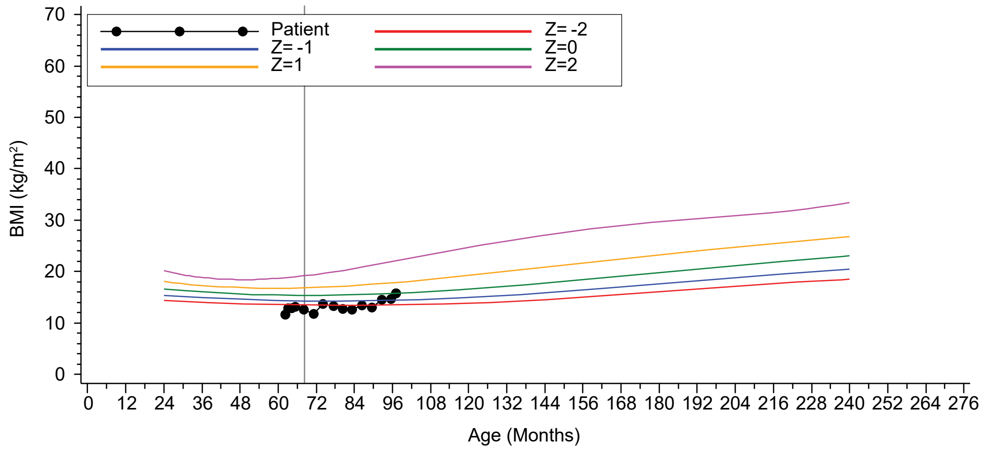

Patient 7
Seizure History: Partial Onset Seizures

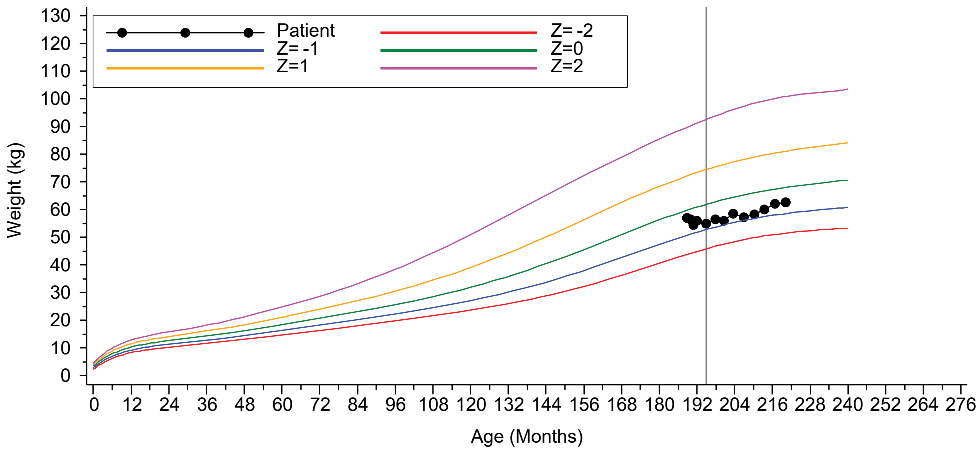

Patient 7
Seizure History: Partial Onset Seizures

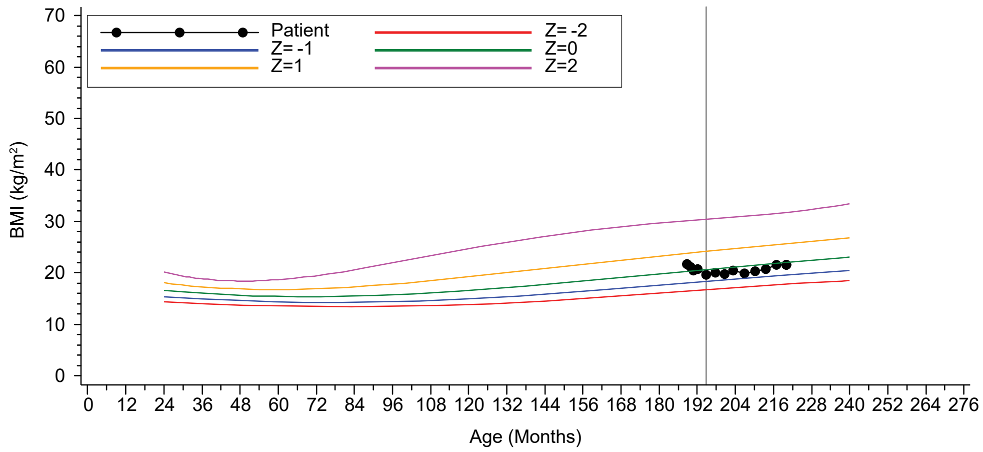

Patient 8
Seizure History: Partial Onset Seizures

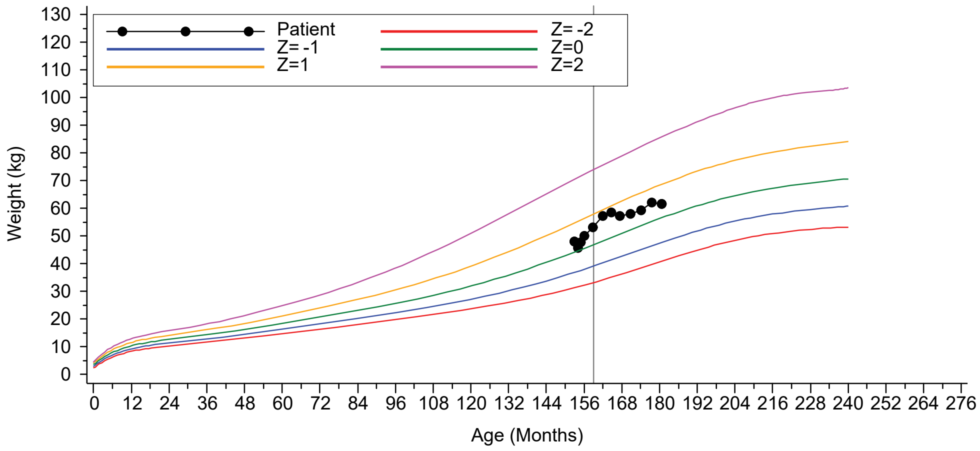

Patient 8
Seizure History: Partial Onset Seizures

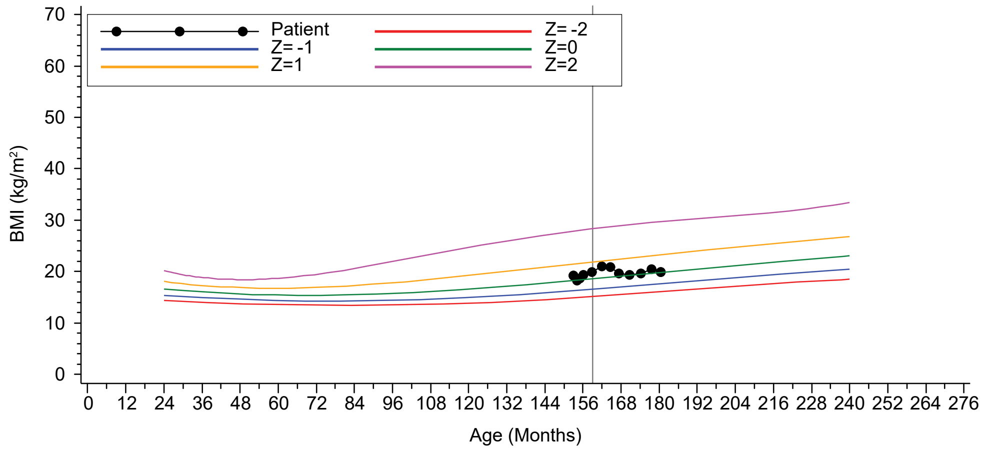

Patient 9
Seizure History: Partial Onset Seizures

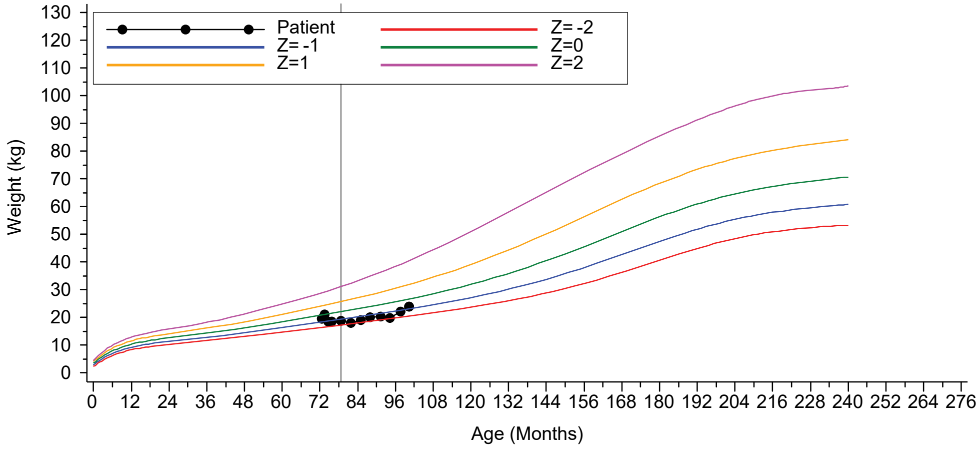

Patient 9
Seizure History: Partial Onset Seizures

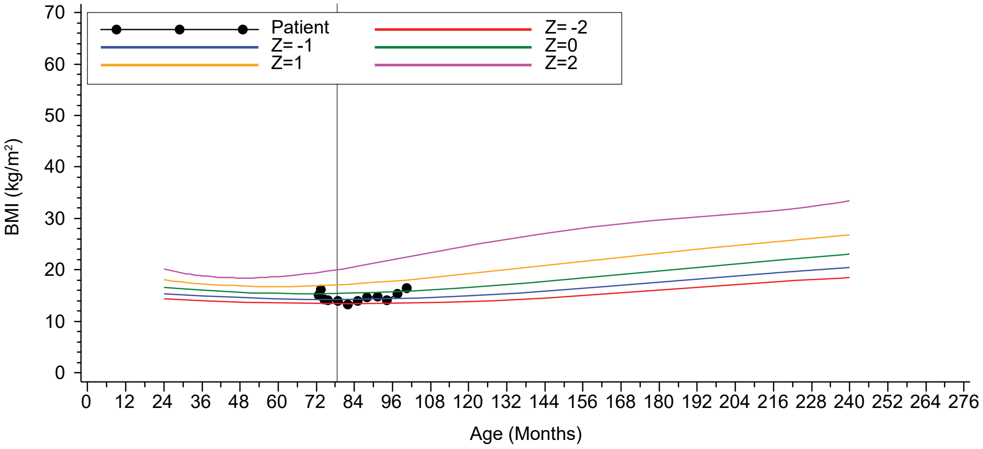

Patient 10
Seizure History: Partial Onset Seizures

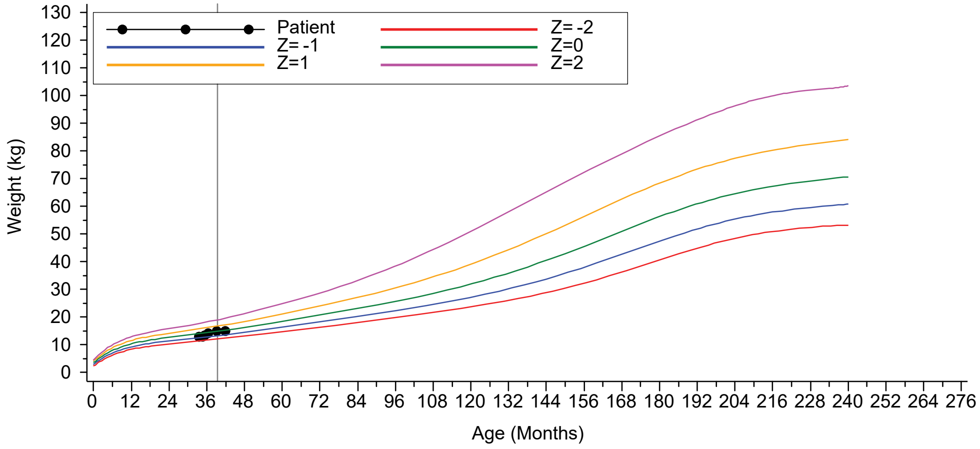

Patient 10
Seizure History: Partial Onset Seizures

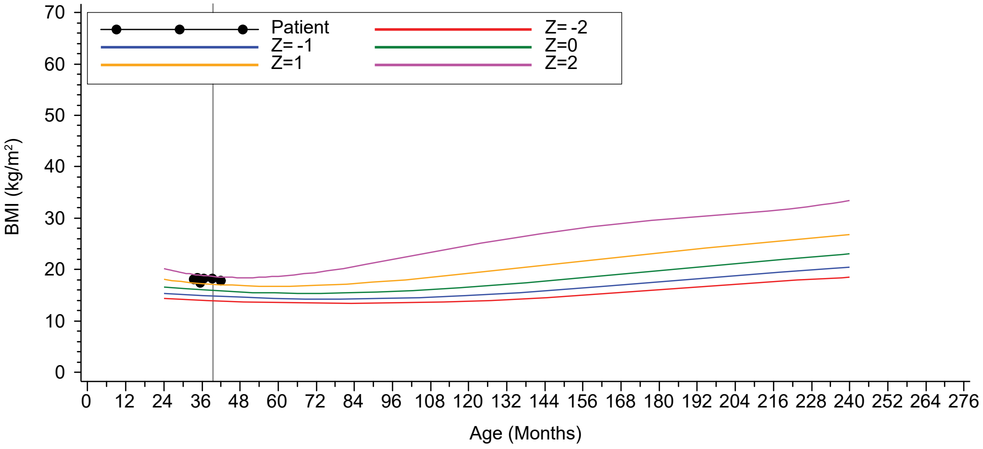

Patient 11
Seizure History: Partial Onset Seizures

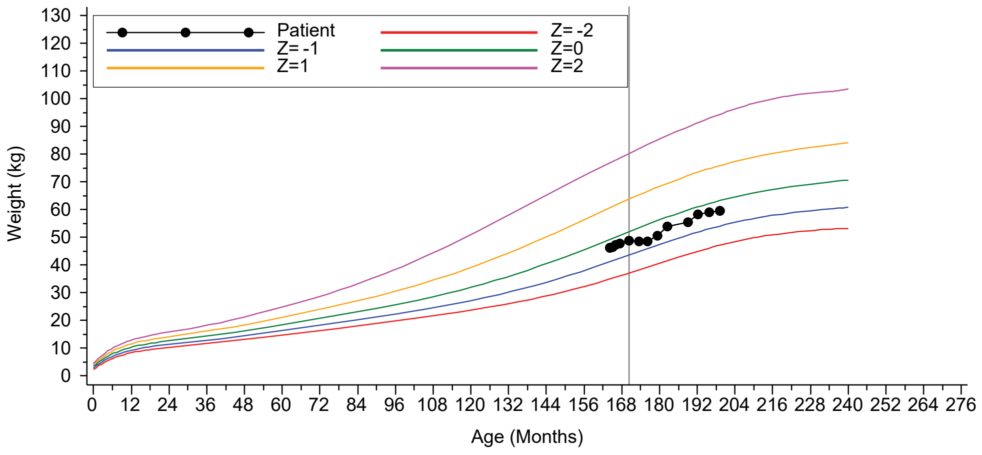

Patient 11
Seizure History: Partial Onset Seizures

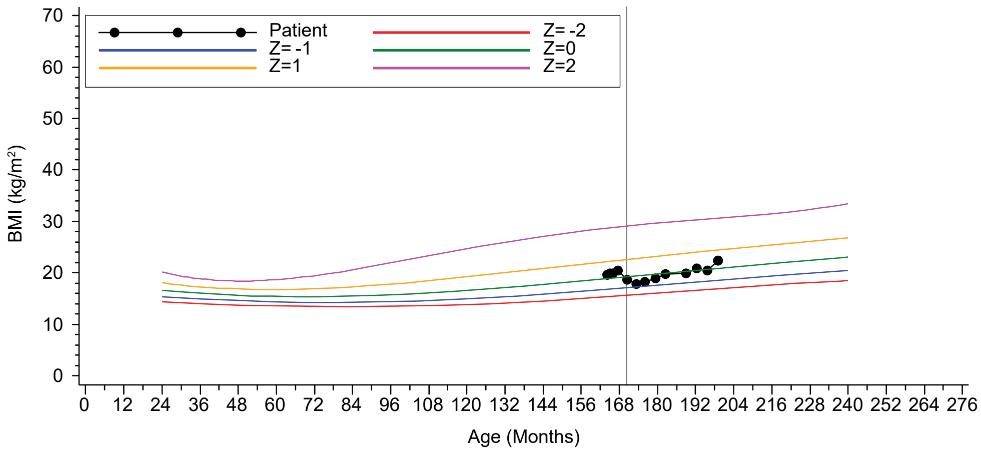

Patient 12
Seizure History: Partial Onset Seizures

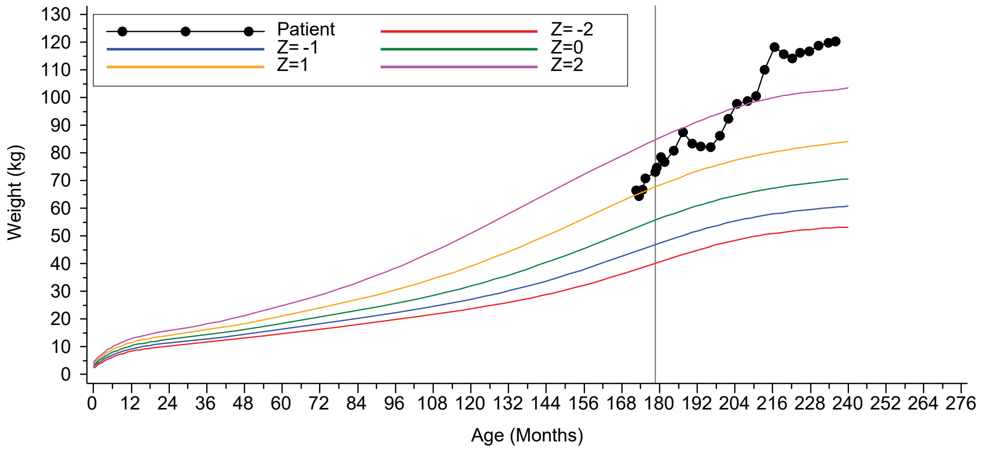

Patient 12
Seizure History: Partial Onset Seizures

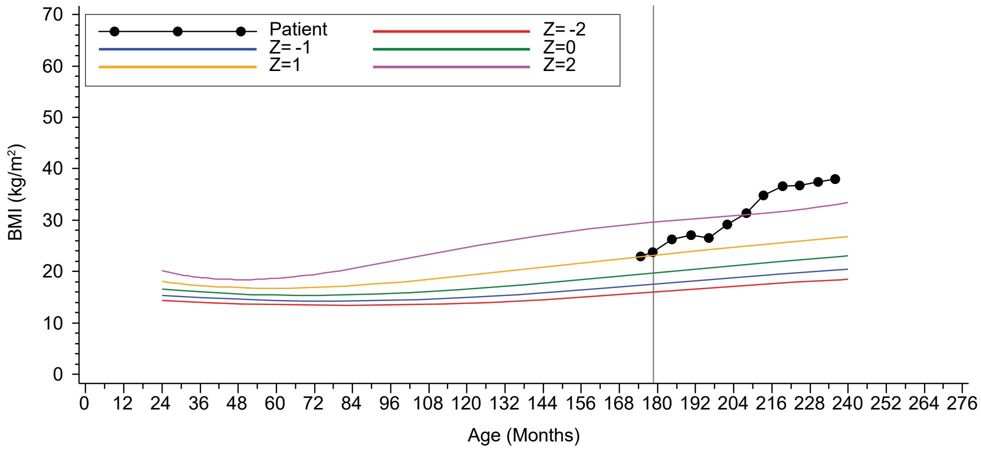

Patient 13
Seizure History: Partial Onset Seizures

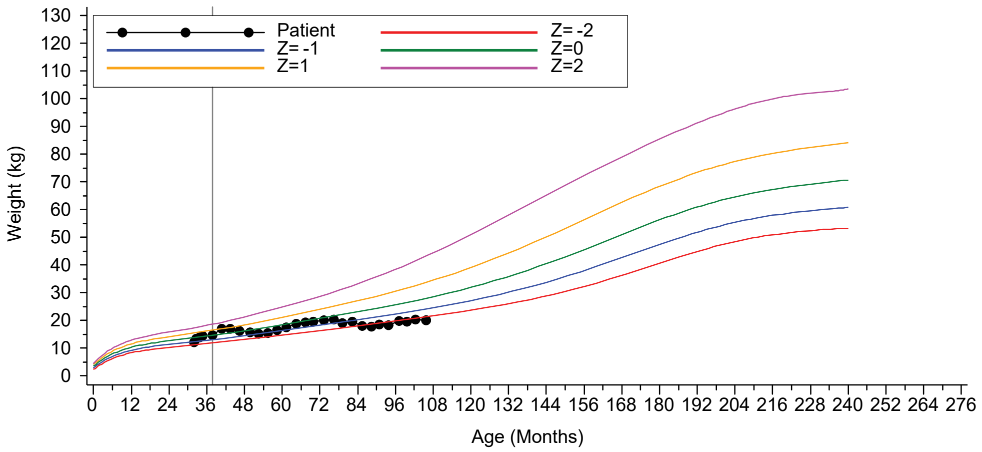

Patient 13
Seizure History: Partial Onset Seizures

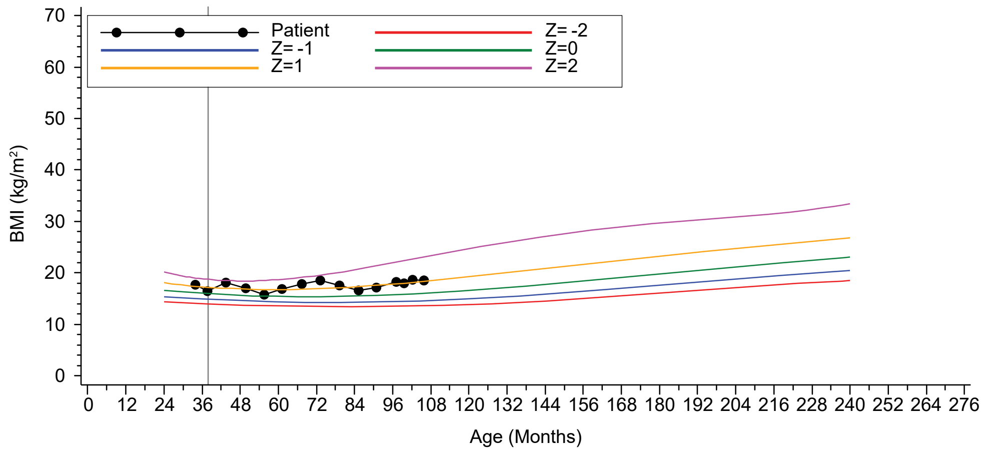

Patient 14
Seizure History: Partial Onset Seizures

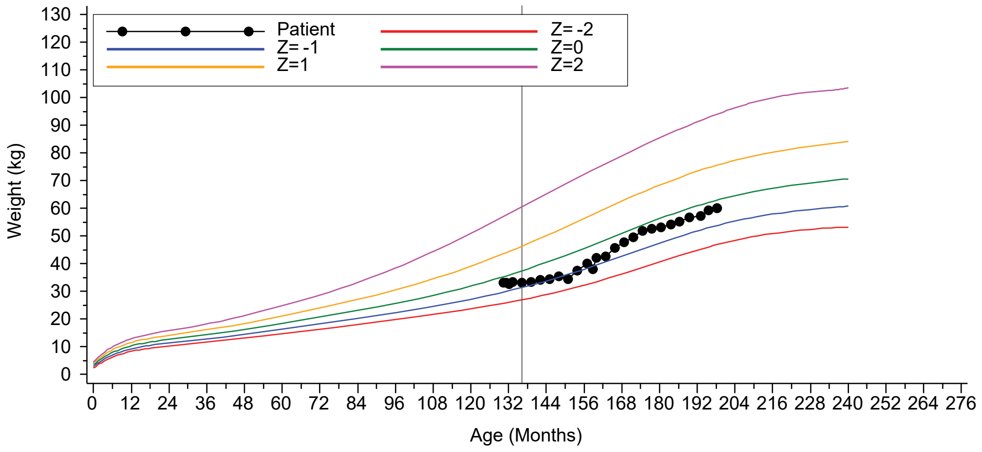

Patient 14
Seizure History: Partial Onset Seizures

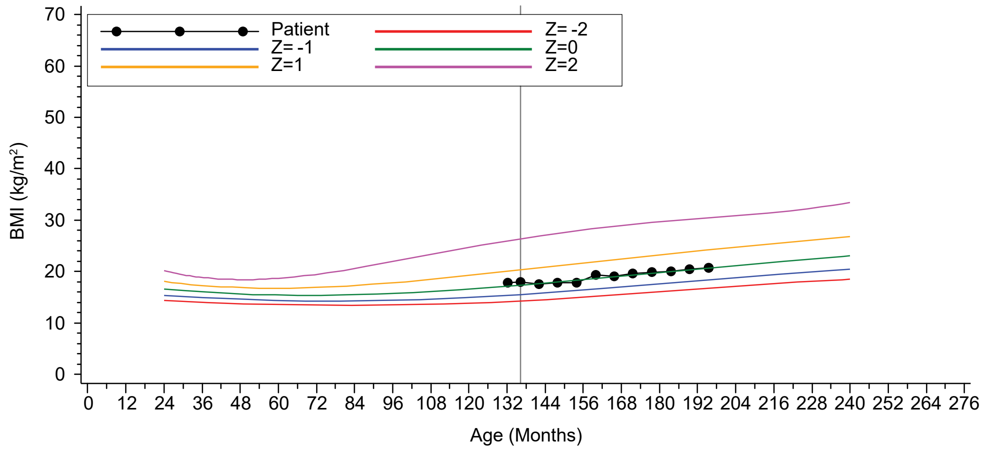

Patient 15
Seizure History: Partial Onset Seizures

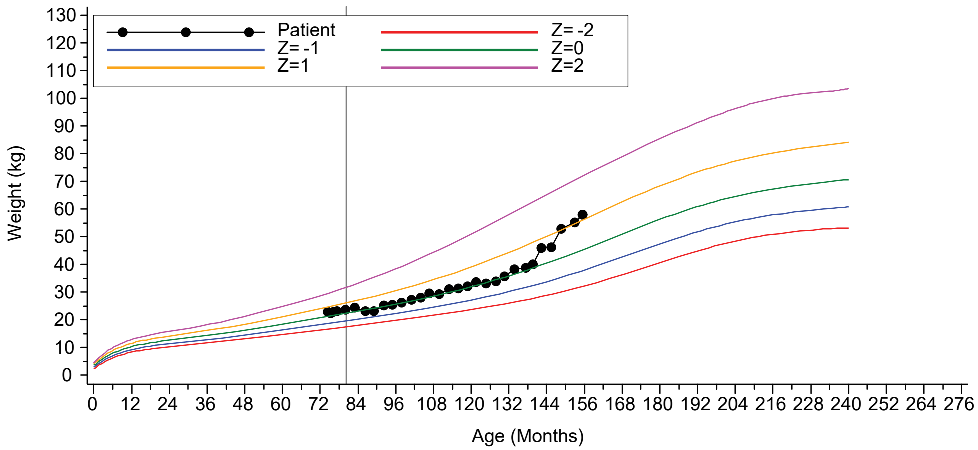

Patient 15
Seizure History: Partial Onset Seizures

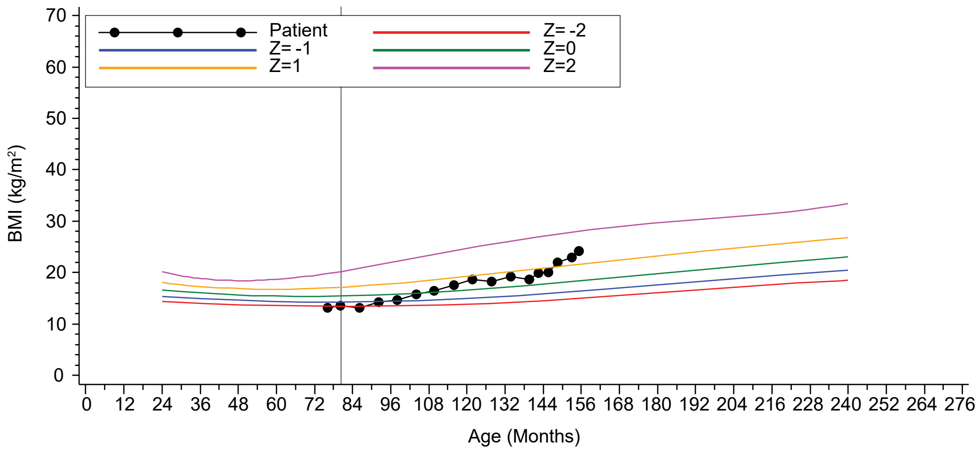

Patient 16
Seizure History: Partial Onset Seizures

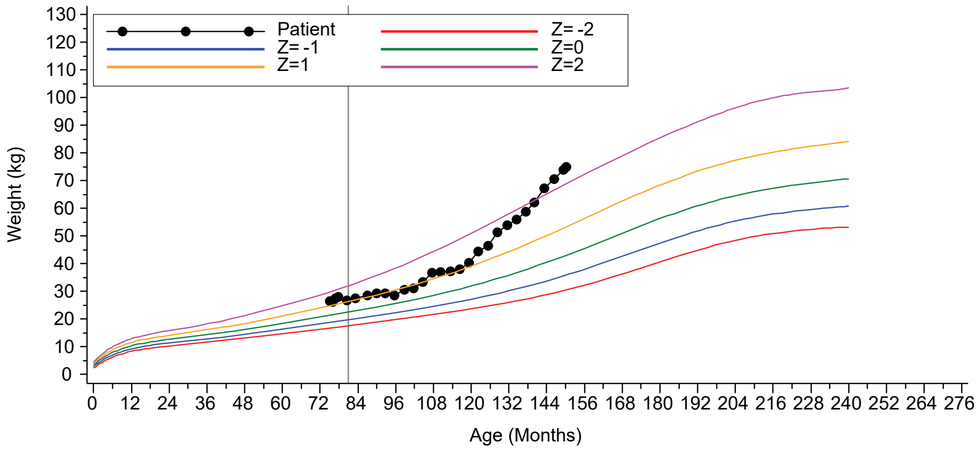

Patient 16
Seizure History: Partial Onset Seizures

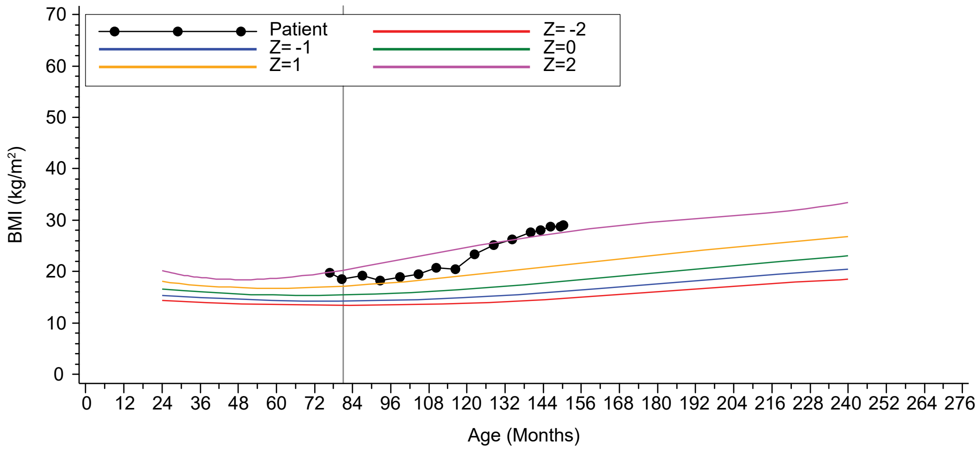

Patient 17
Seizure History: Partial Onset Seizures

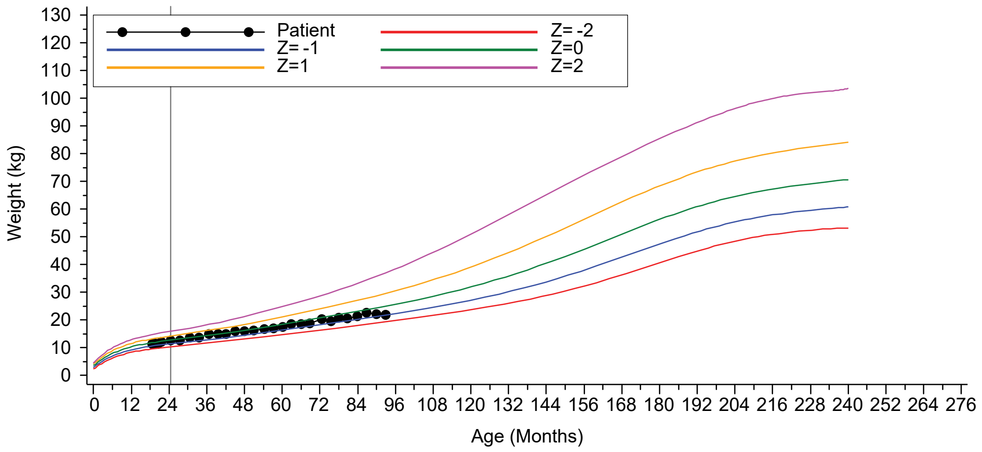

Patient 17
Seizure History: Partial Onset Seizures

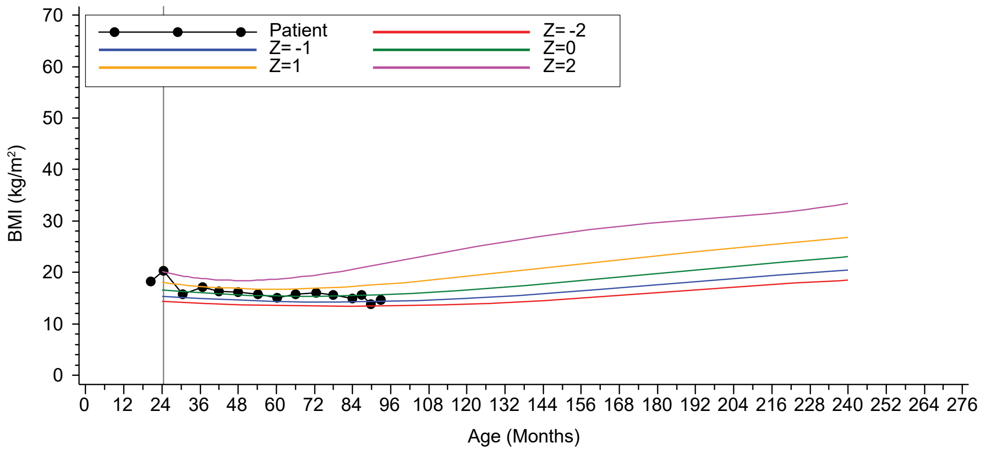

Patient 18
Seizure History: Partial Onset Seizures

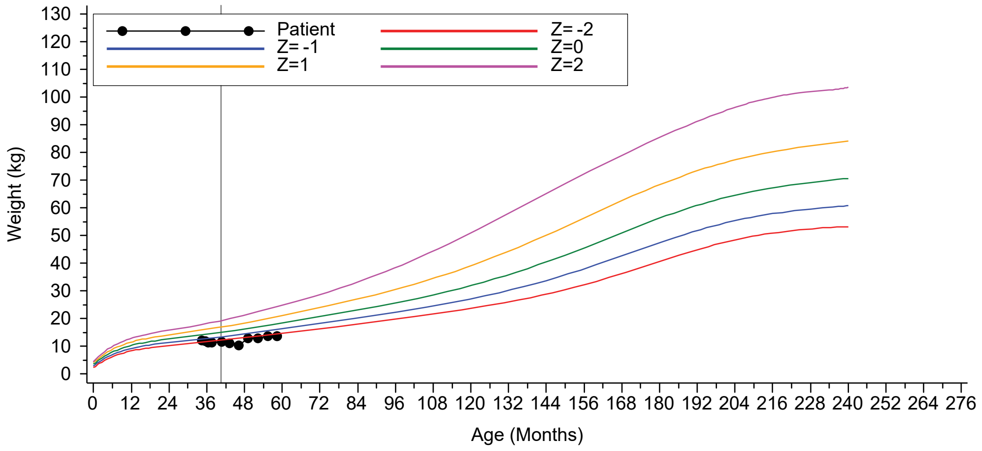

Patient 18
Seizure History: Partial Onset Seizures

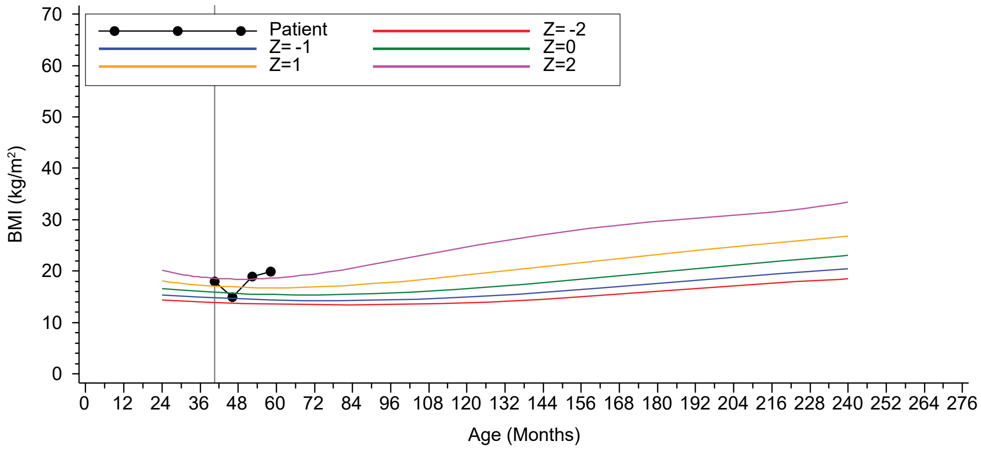

Patient 19
Seizure History: Partial Onset Seizures

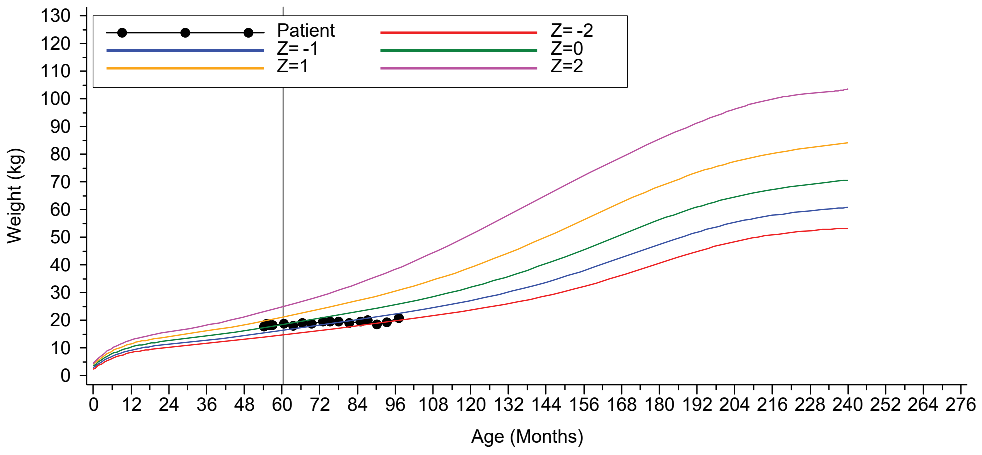

Patient 19
Seizure History: Partial Onset Seizures

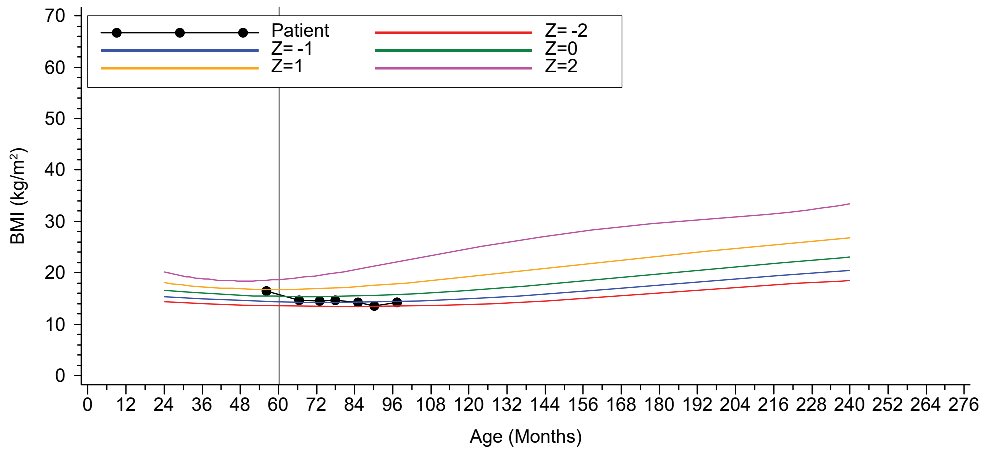

Patient 20
Seizure History: Partial Onset Seizures

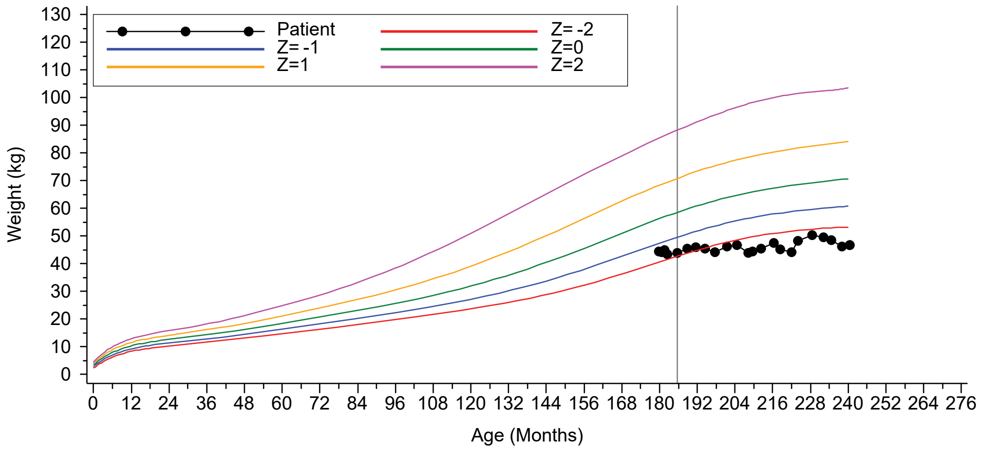

Patient 20
Seizure History: Partial Onset Seizures

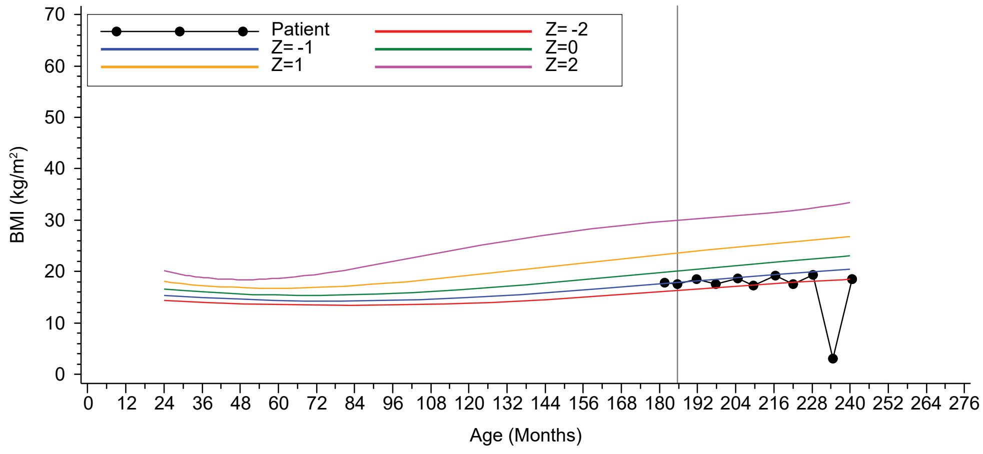

Patient 21
Seizure History: Partial Onset Seizures

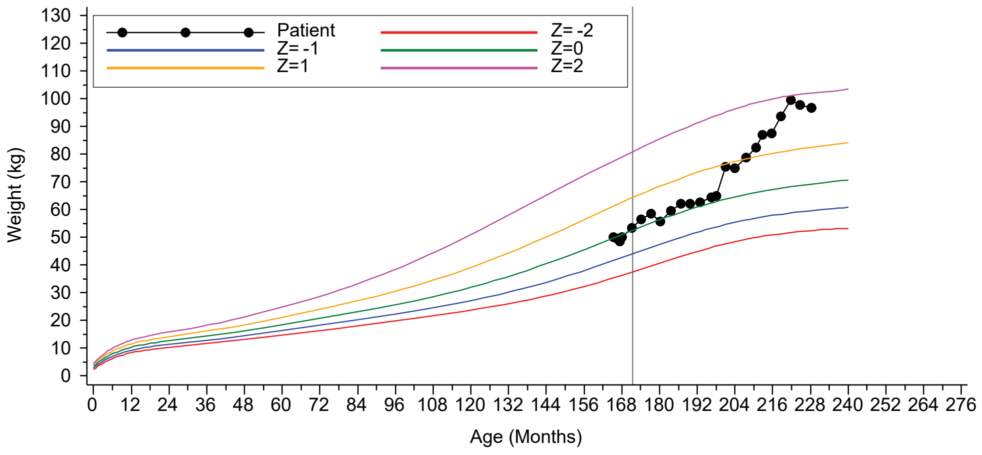

Patient 21
Seizure History: Partial Onset Seizures

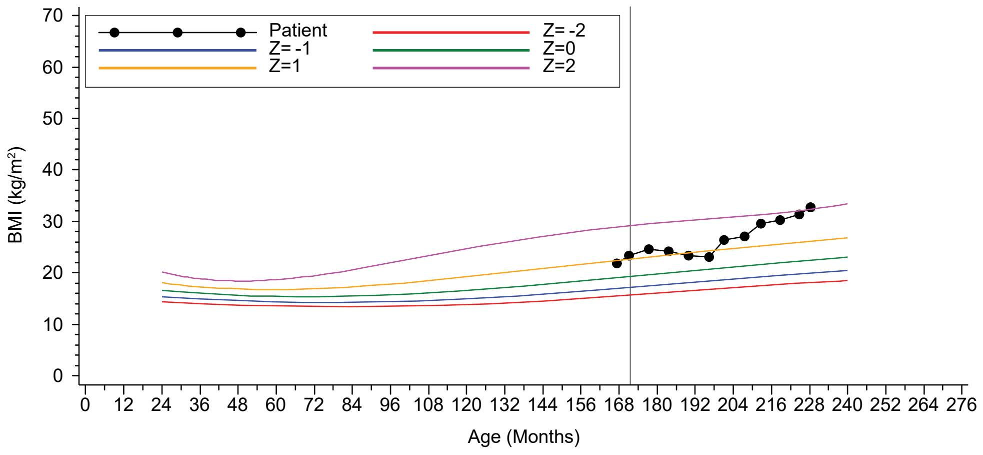

Patient 22
Seizure History: Partial Onset Seizures

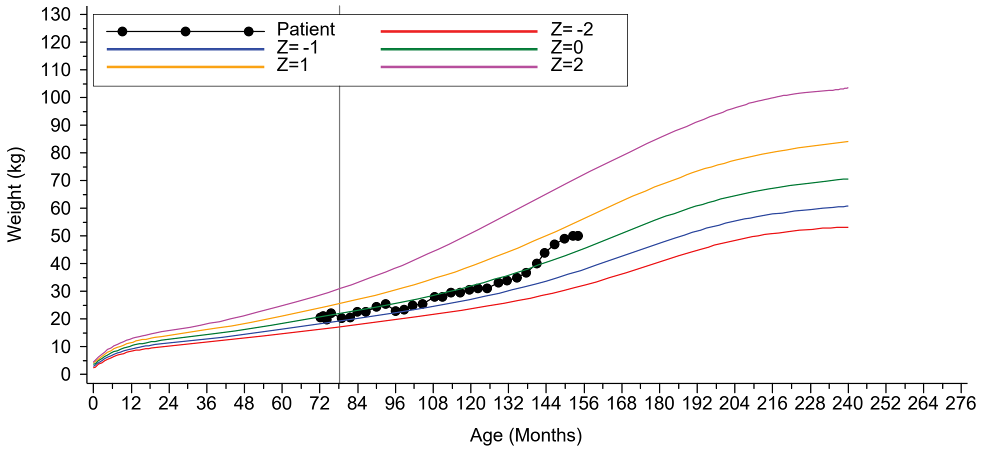

Patient 22
Seizure History: Partial Onset Seizures

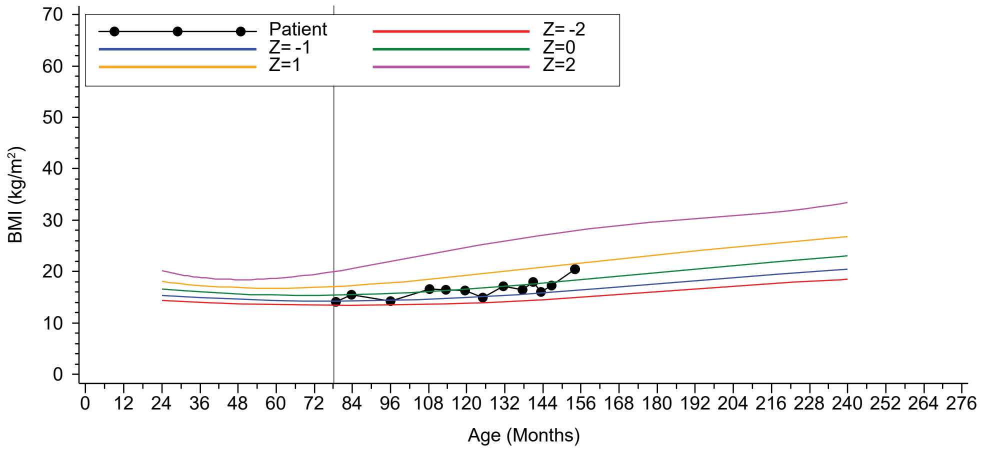

Patient 23
Seizure History: Partial Onset Seizures

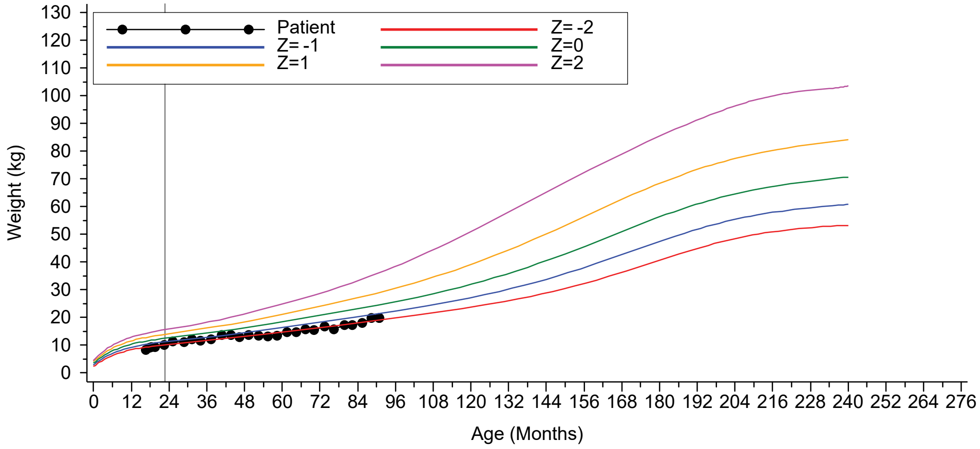

Patient 23
Seizure History: Partial Onset Seizures

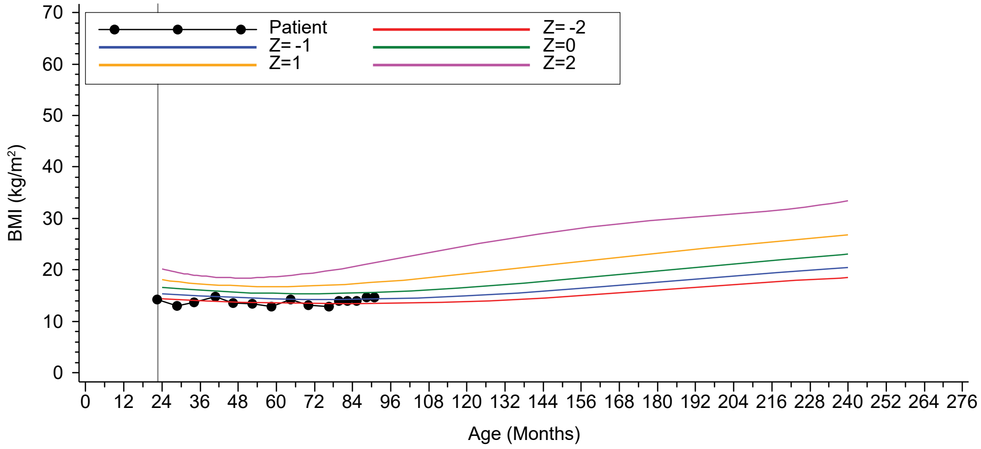

Patient 24
Seizure History: Partial Onset Seizures

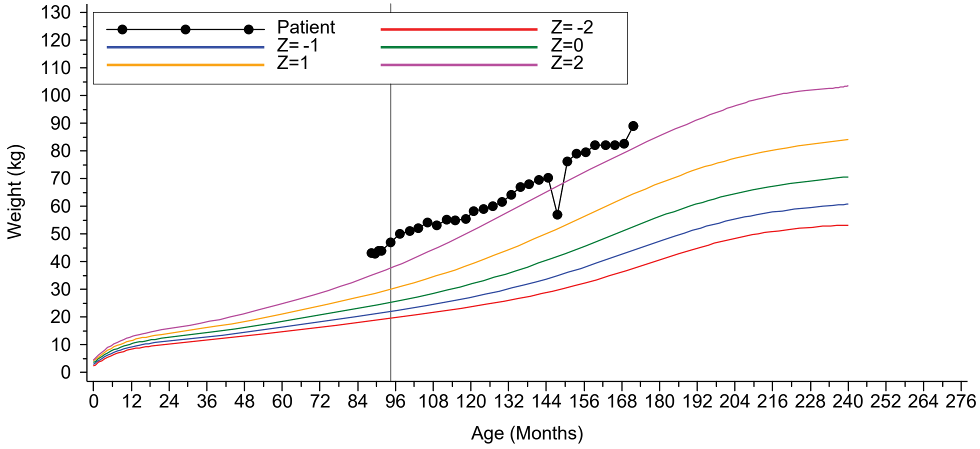

Patient 24
Seizure History: Partial Onset Seizures

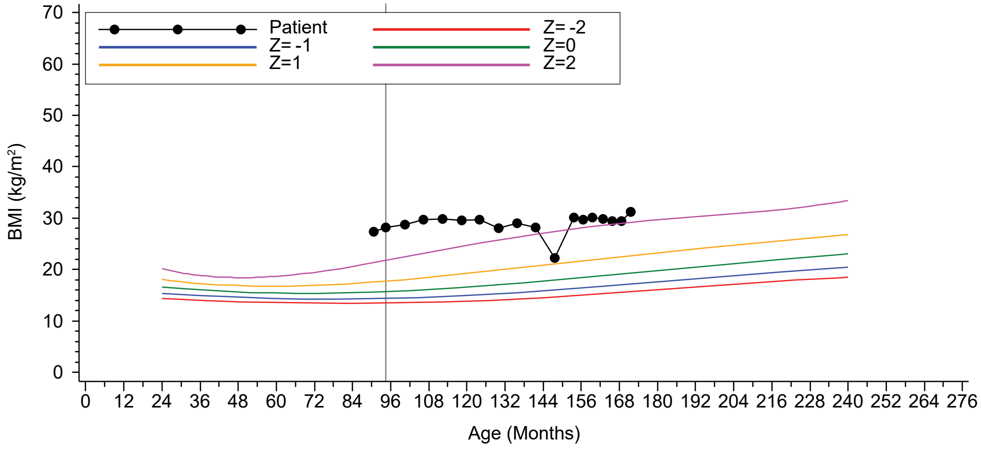

Patient 25
Seizure History: Partial Onset Seizures

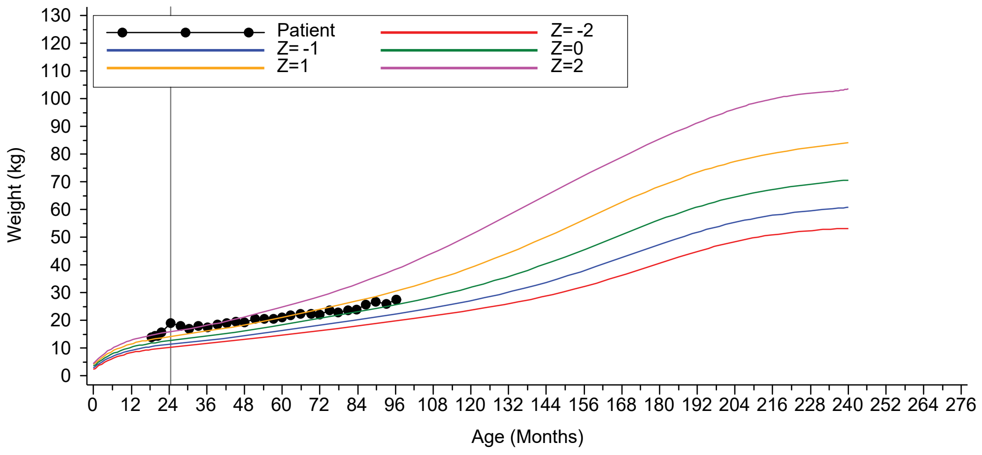

Patient 25
Seizure History: Partial Onset Seizures

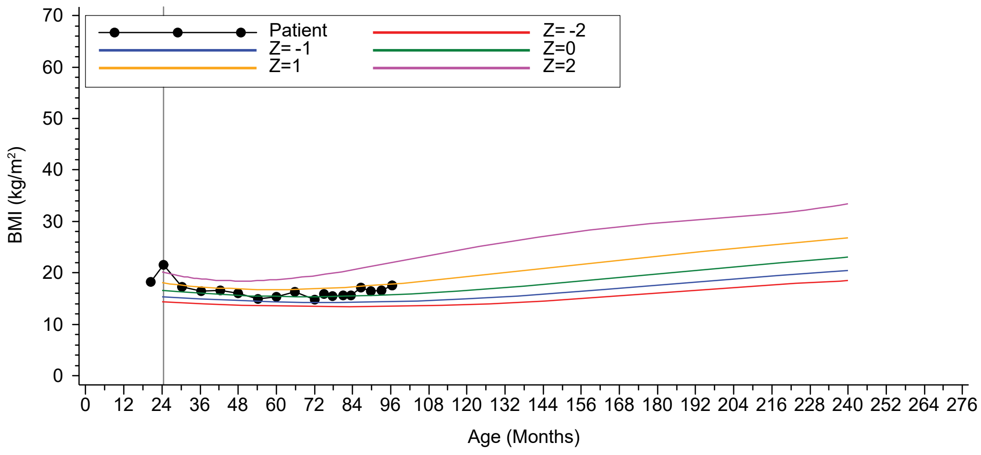

Patient 26
Seizure History: Partial Onset Seizures

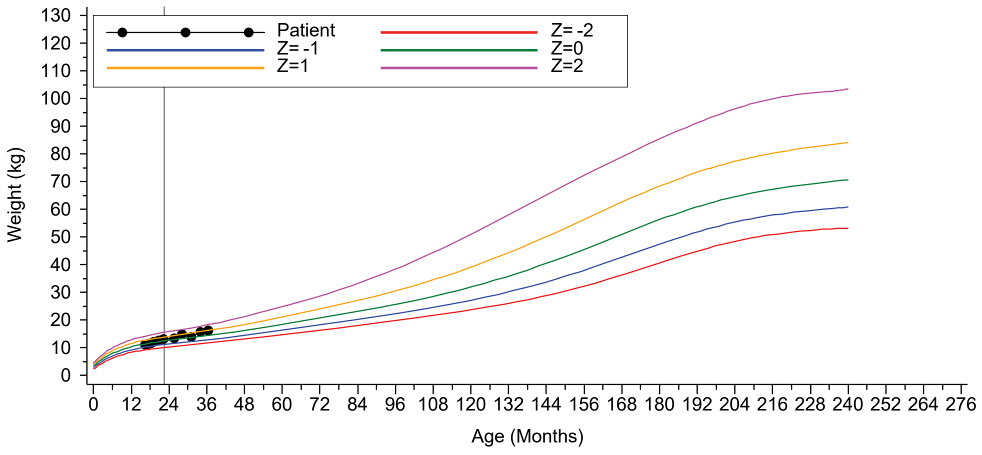

Patient 26
Seizure History: Partial Onset Seizures

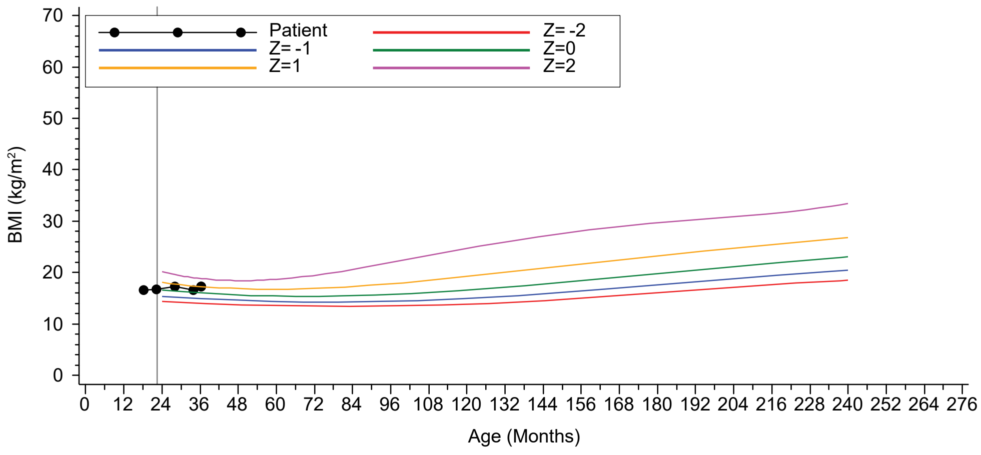

Patient 27
Seizure History: Partial Onset Seizures

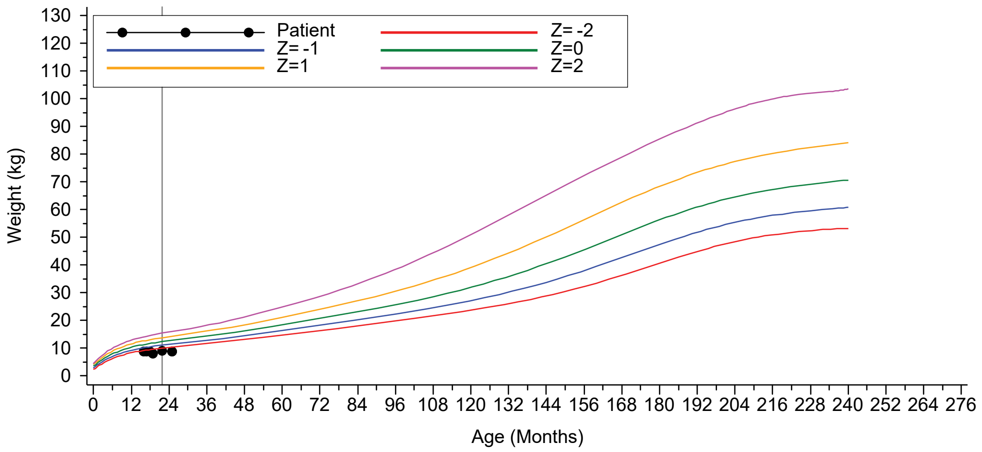

Patient 27
Seizure History: Partial Onset Seizures

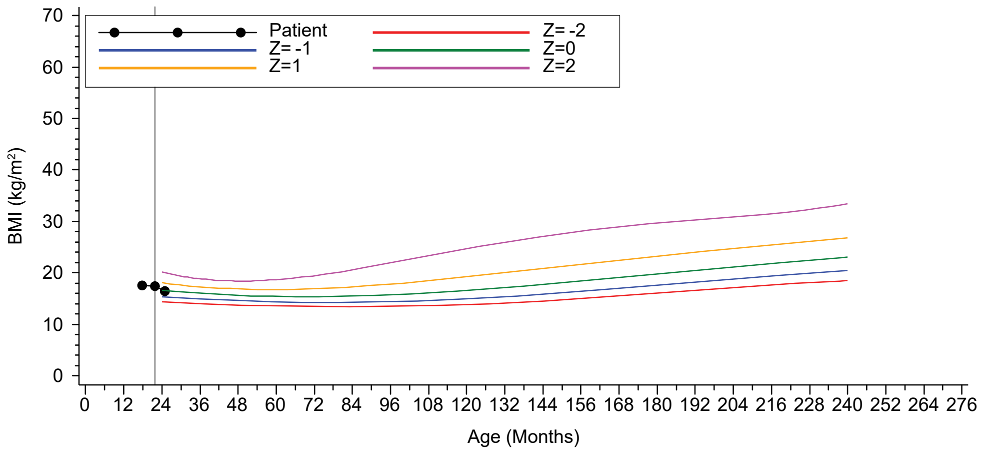

Patient 28
Seizure History: Partial Onset Seizures

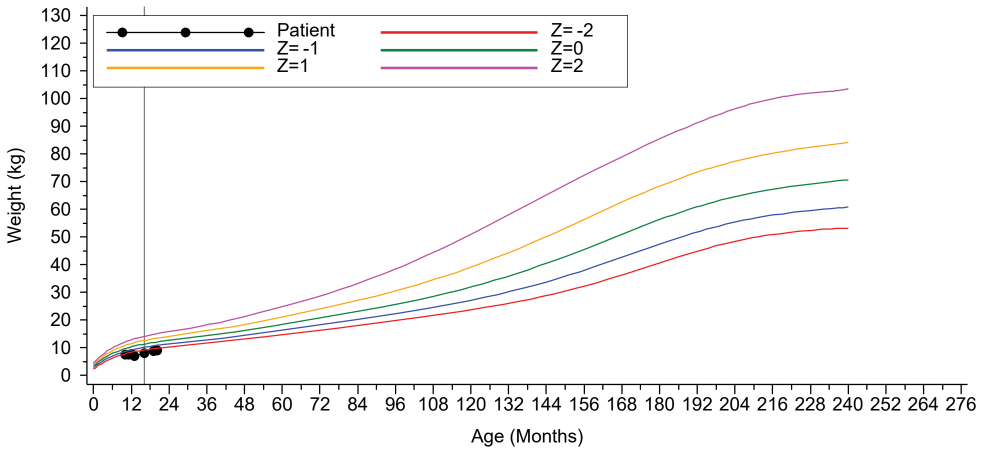

Patient 28
Seizure History: Partial Onset Seizures

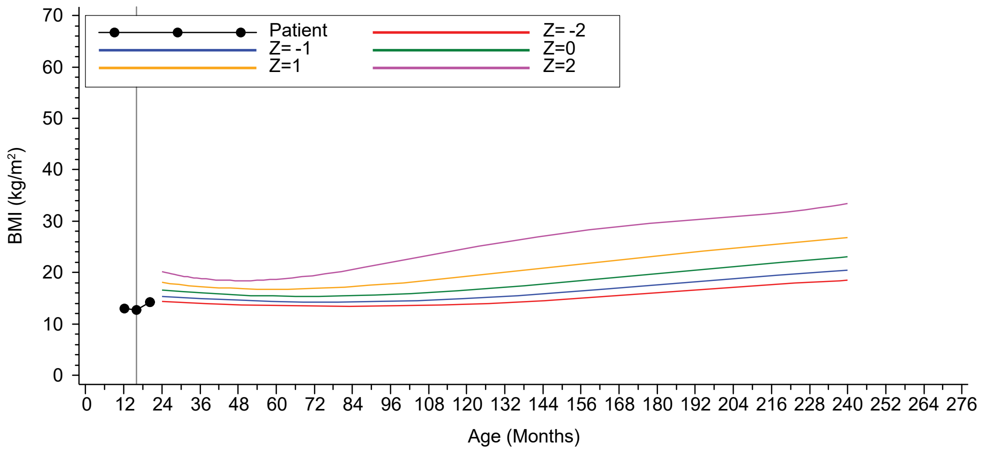

Patient 29
Seizure History: Partial Onset Seizures

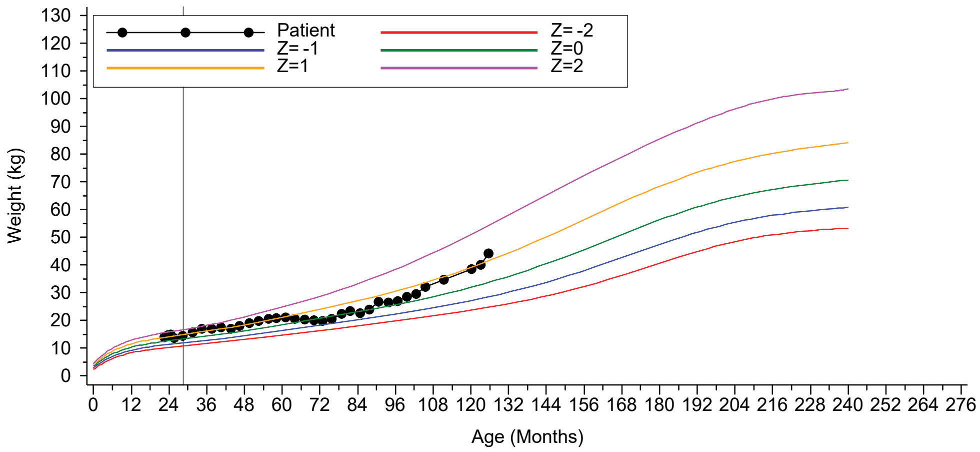

Patient 29
Seizure History: Partial Onset Seizures

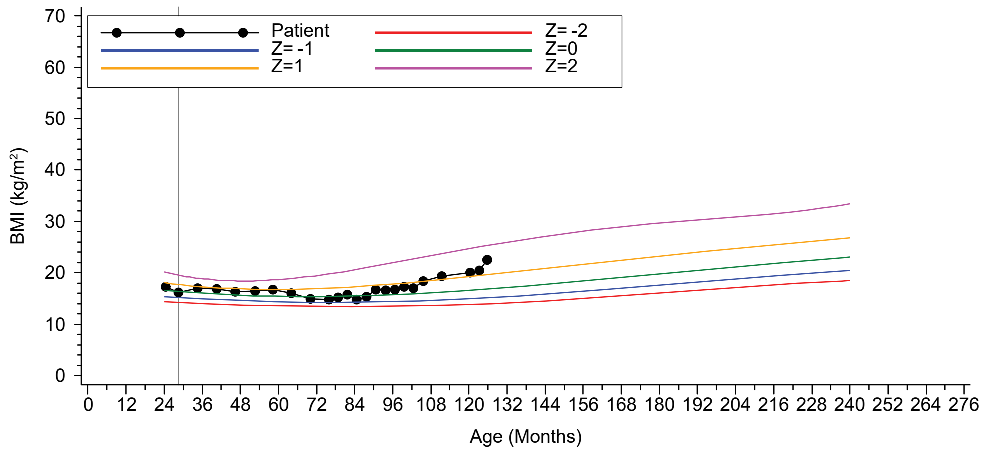

Patient 30
Seizure History: Partial Onset Seizures

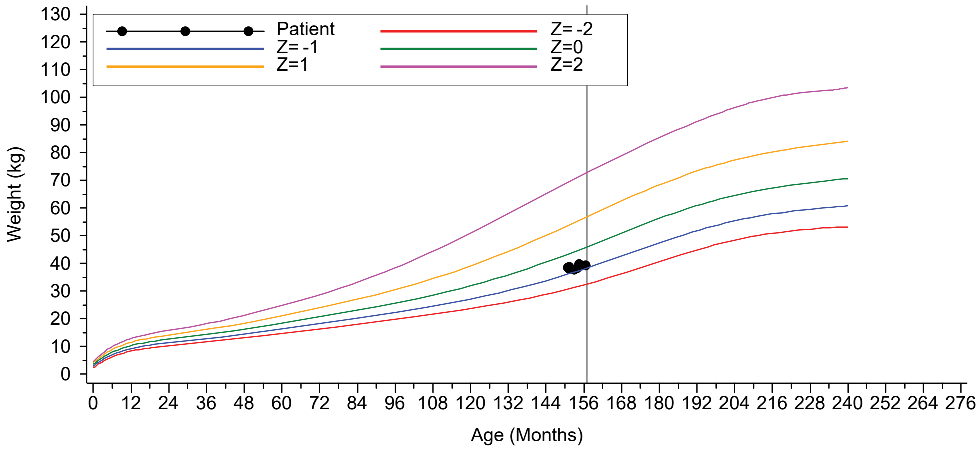

Patient 30
Seizure History: Partial Onset Seizures

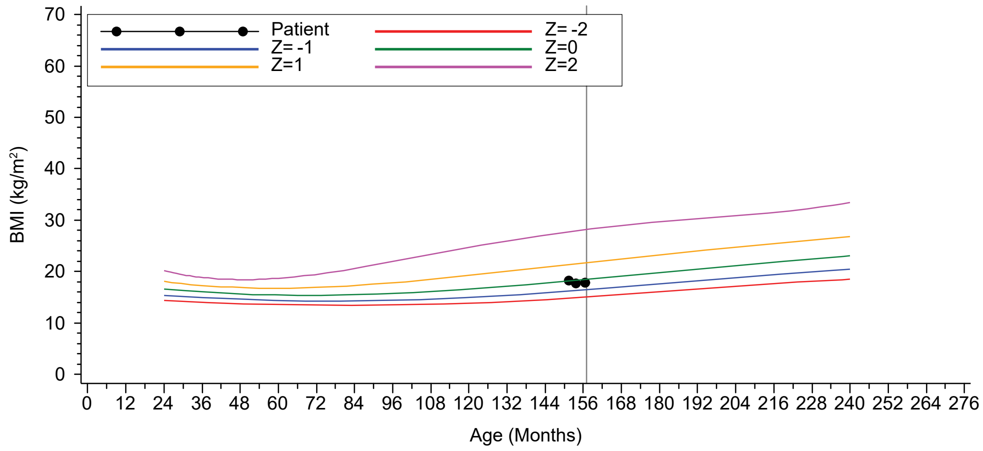

Patient 31
Seizure History: Partial Onset Seizures

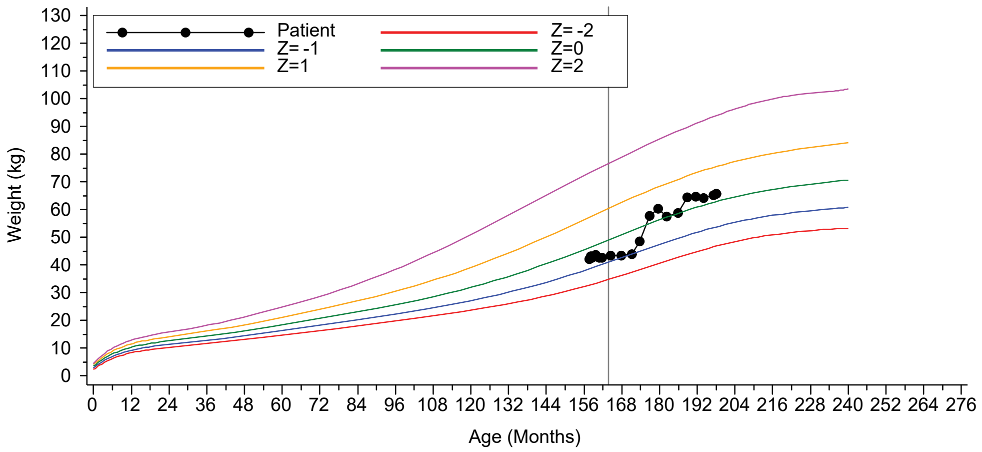

Patient 31
Seizure History: Partial Onset Seizures

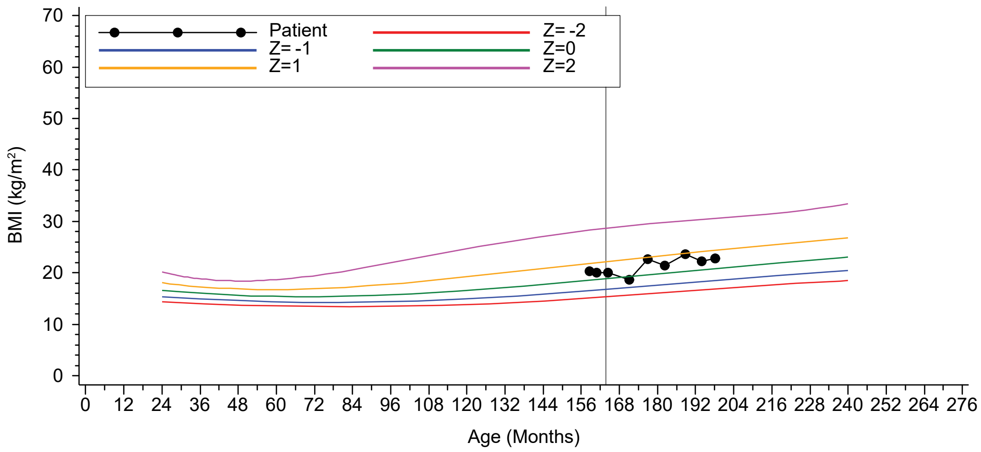

Patient 32
Seizure History: Partial Onset Seizures

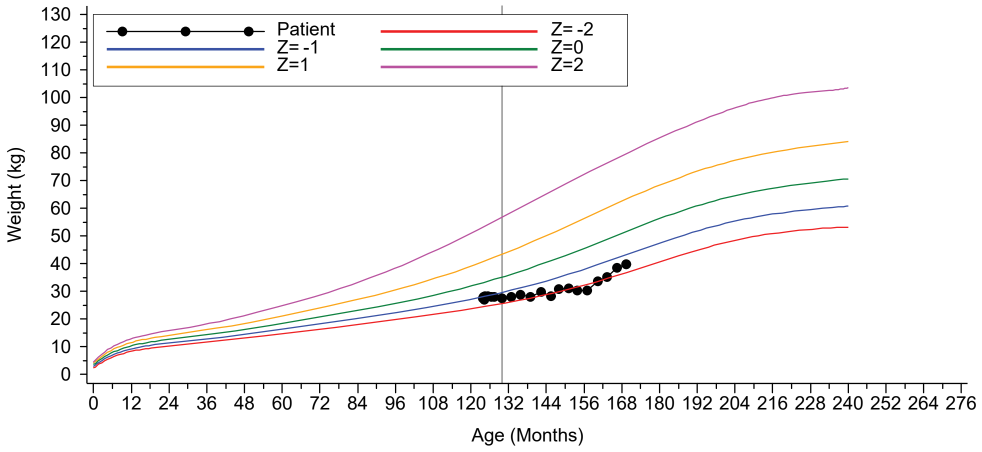

Patient 32
Seizure History: Partial Onset Seizures

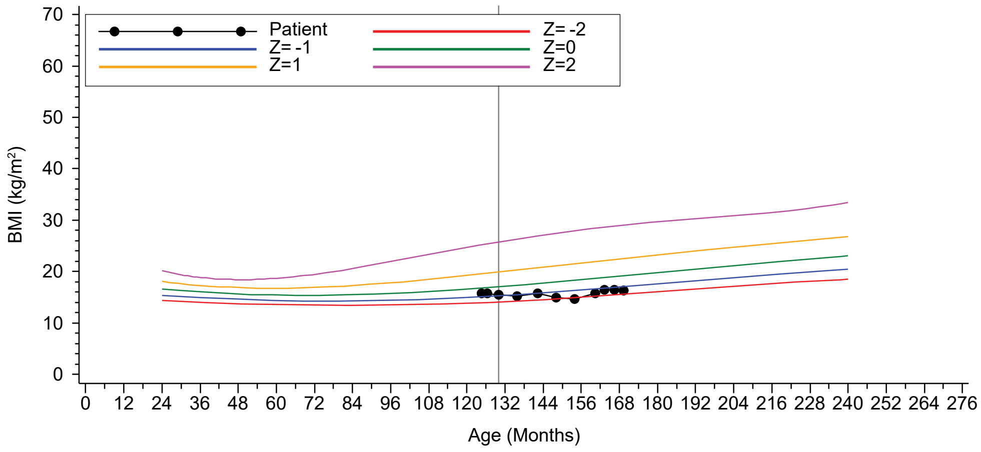

Patient 33
Seizure History: Partial Onset Seizures

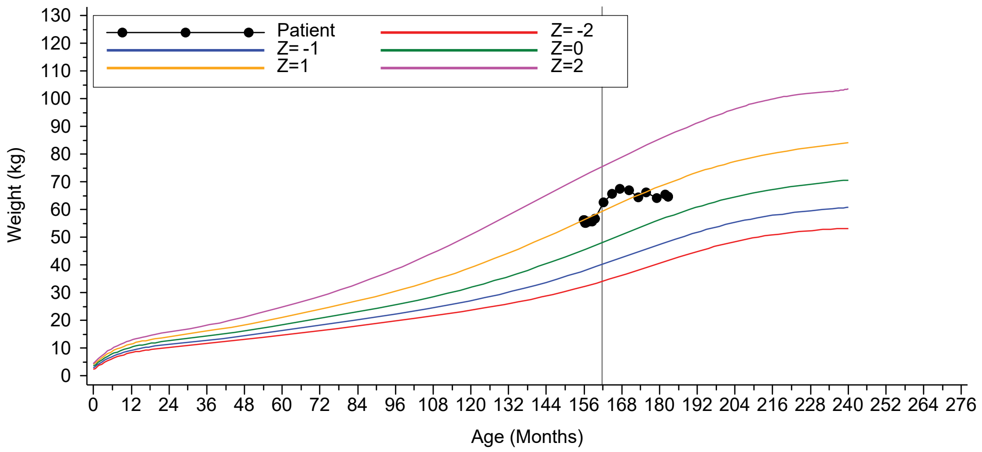

Patient 33
Seizure History: Partial Onset Seizures

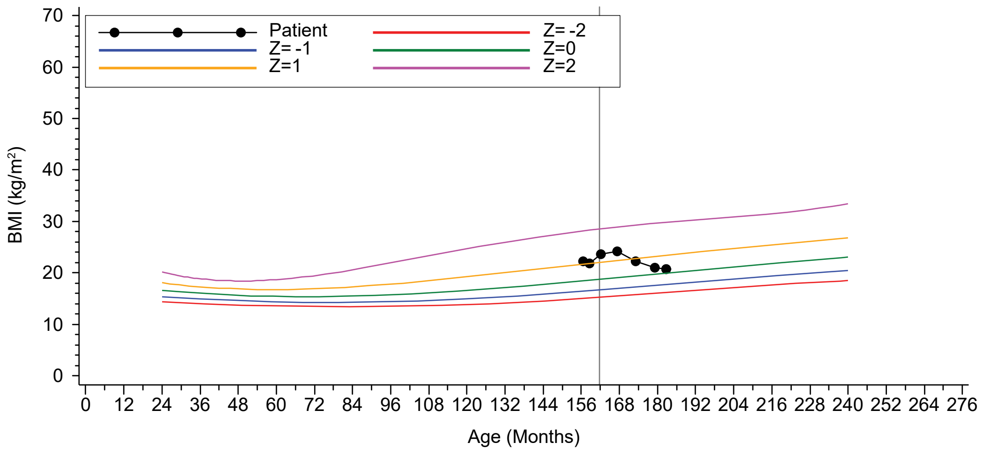

Patient 34
Seizure History: Partial Onset Seizures

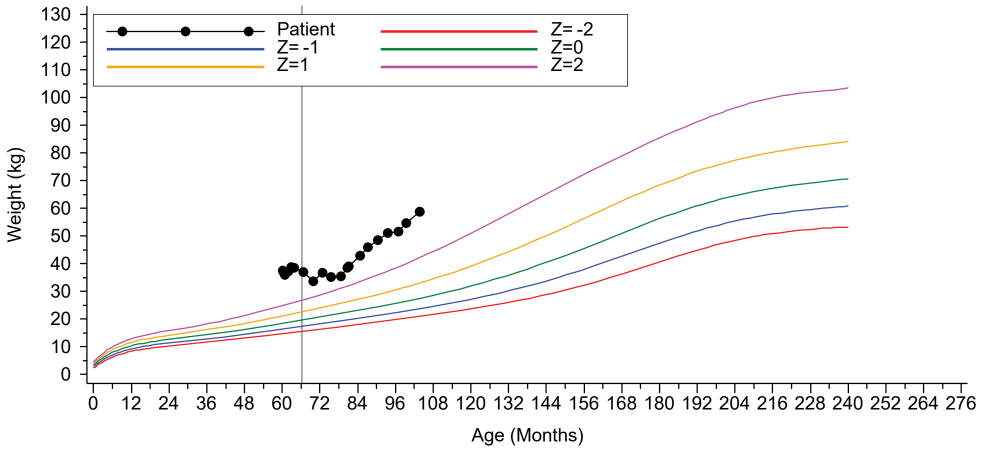

Patient 34
Seizure History: Partial Onset Seizures

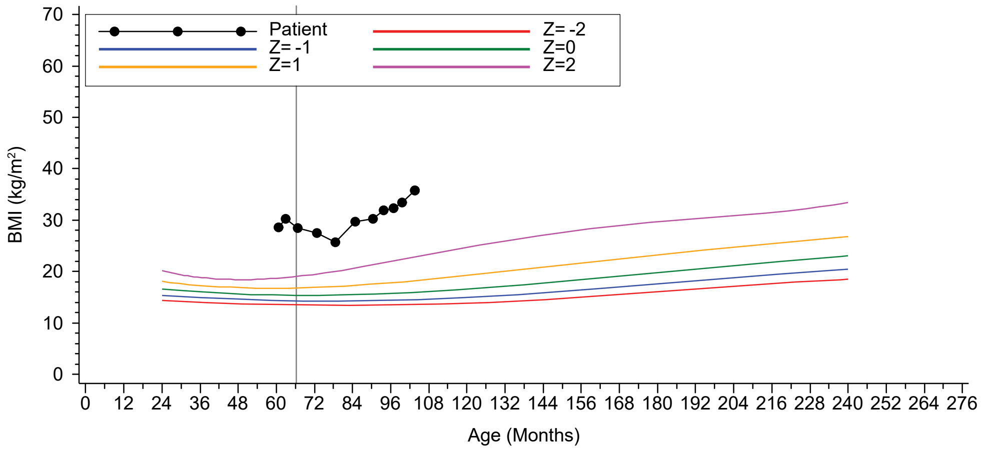

Patient 35
Seizure History: Partial Onset Seizures

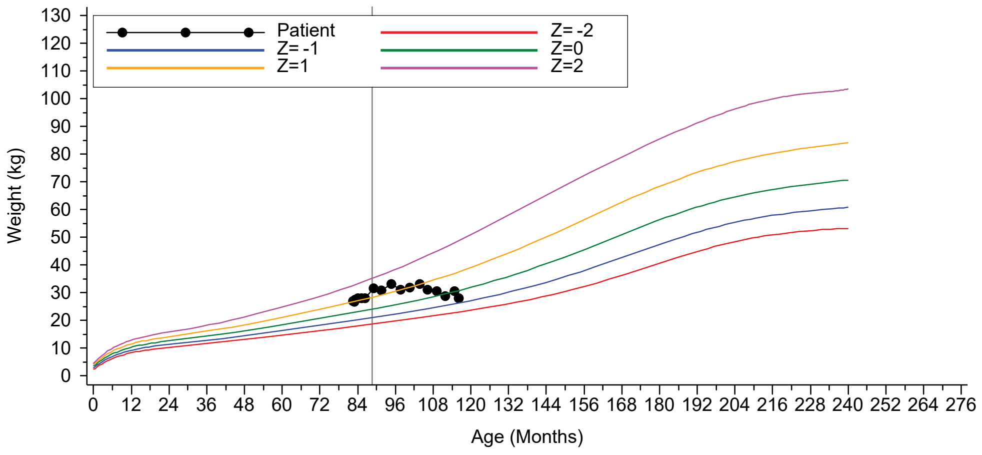

Patient 35
Seizure History: Partial Onset Seizures

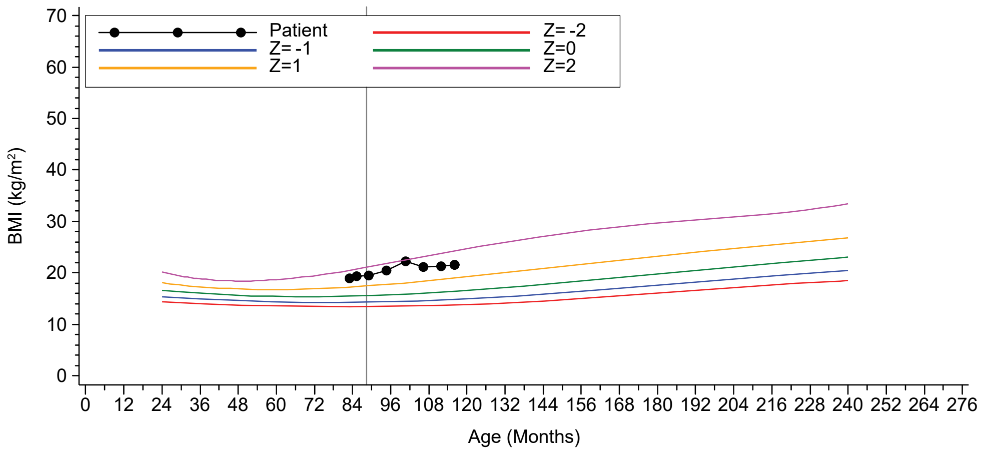

Patient 36
Seizure History: Partial Onset Seizures

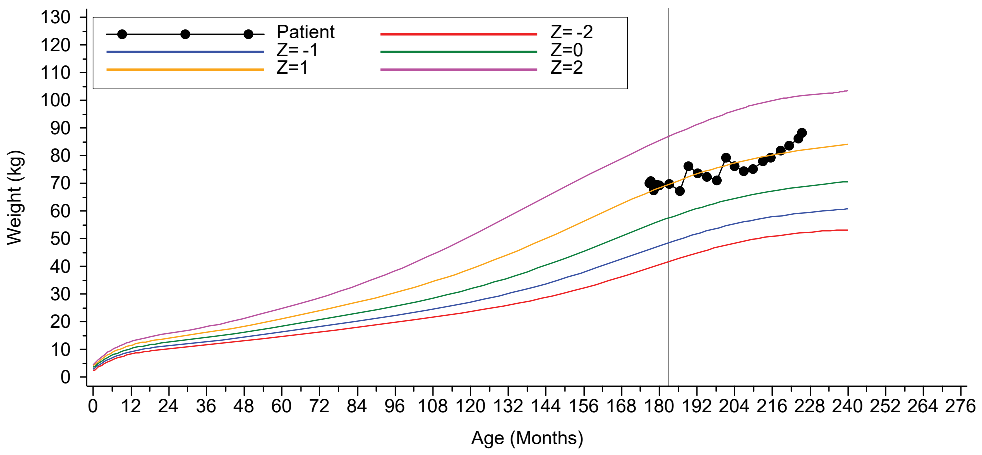

Patient 36
Seizure History: Partial Onset Seizures

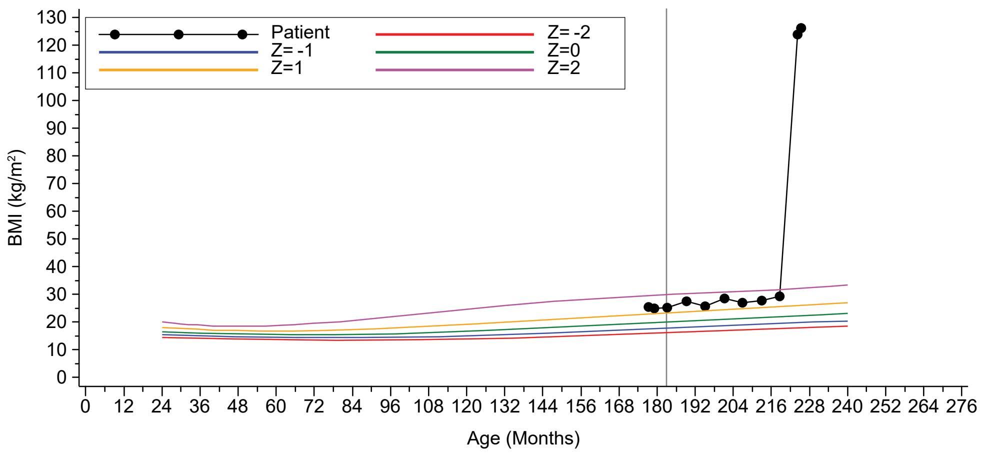

Patient 37
Seizure History: Partial Onset Seizures

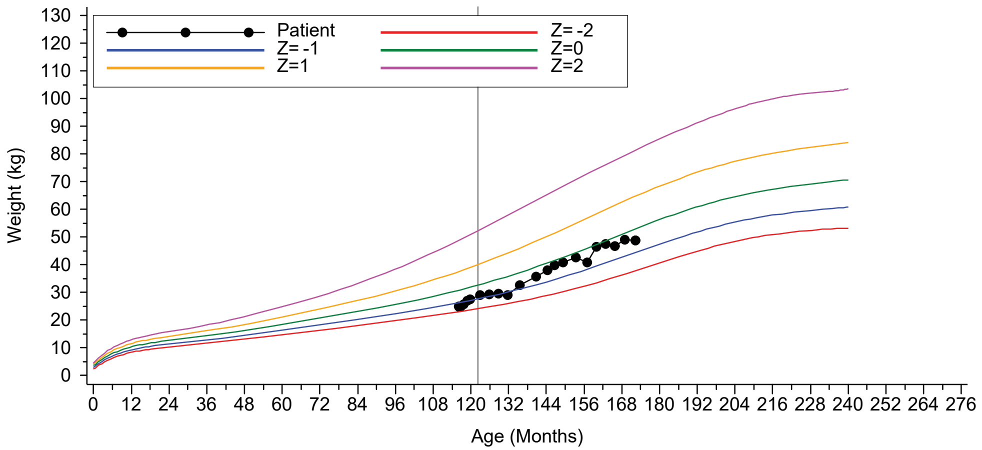

Patient 37
Seizure History: Partial Onset Seizures

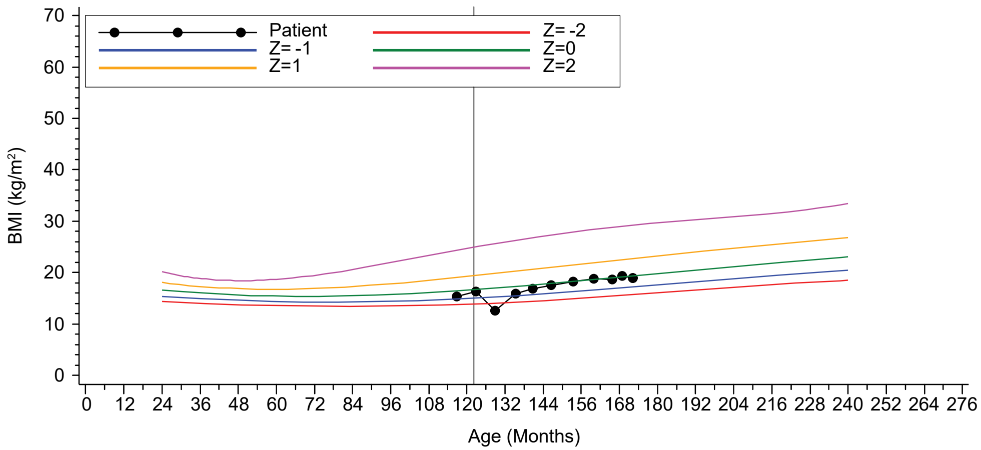

Patient 38
Seizure History: Partial Onset Seizures

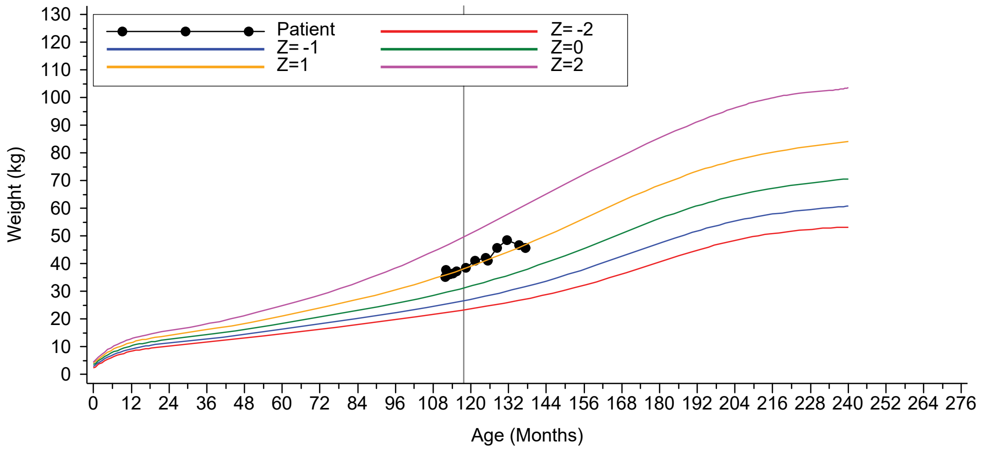

Patient 38
Seizure History: Partial Onset Seizures

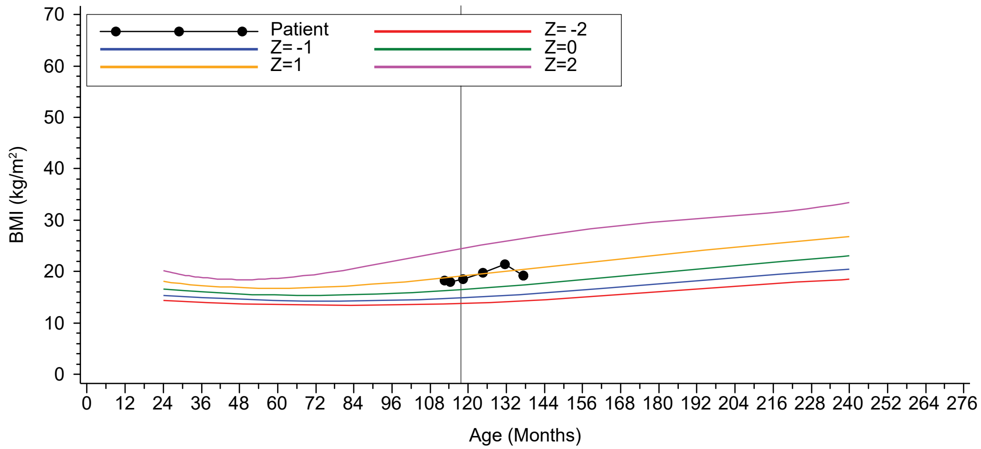

Patient 39
Seizure History: Partial Onset Seizures

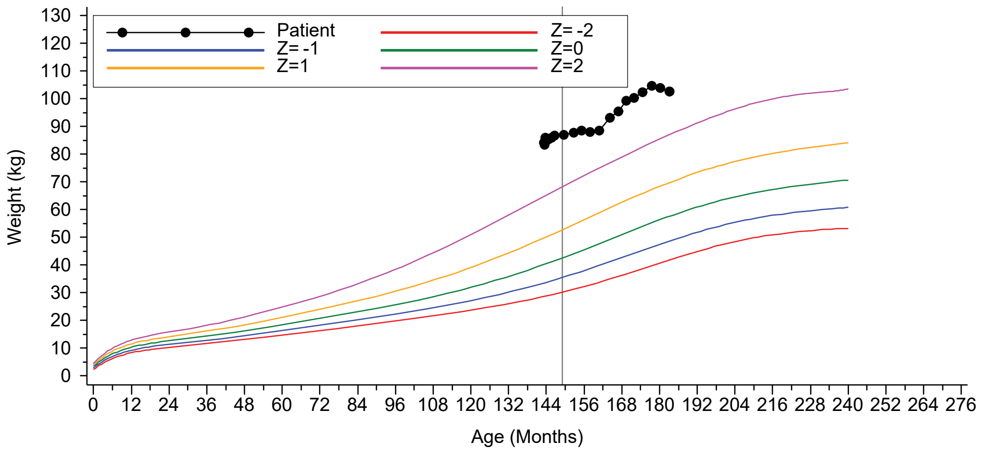

Patient 39
Seizure History: Partial Onset Seizures

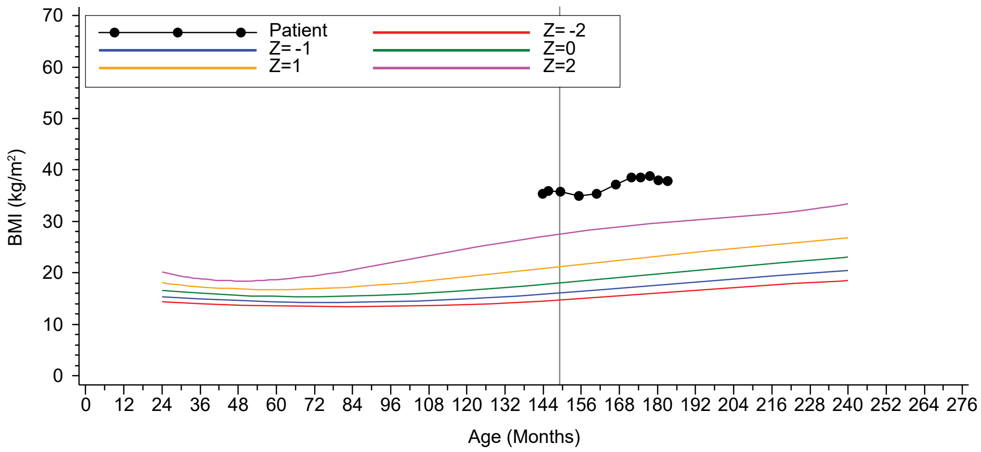

Patient 40
Seizure History: Partial Onset Seizures

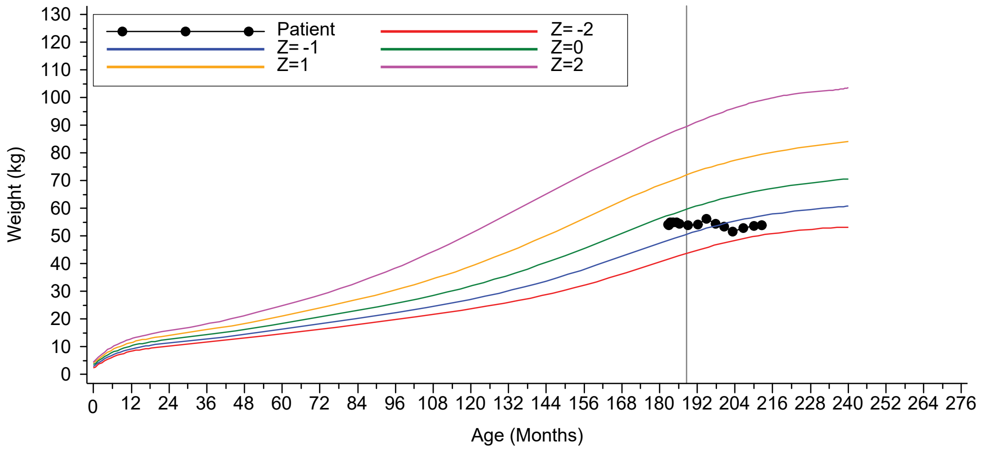

Patient 40
Seizure History: Partial Onset Seizures

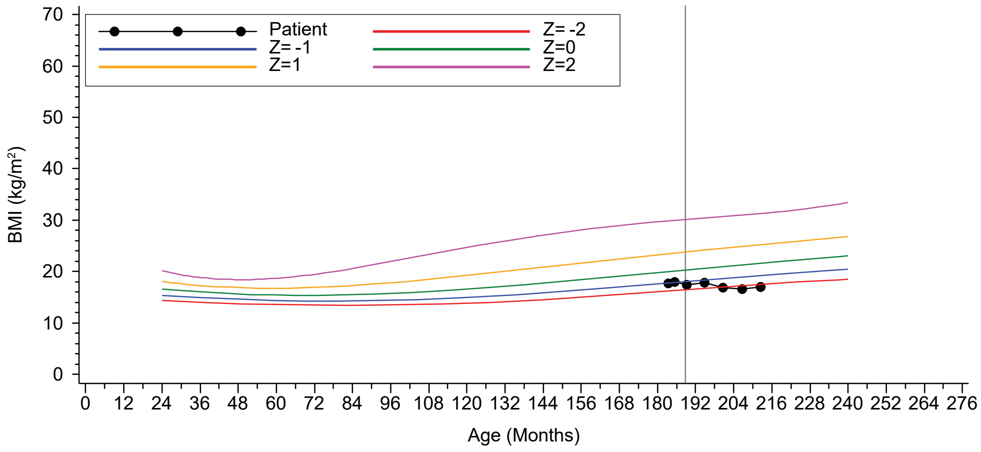

Patient 41
Seizure History: Partial Onset Seizures

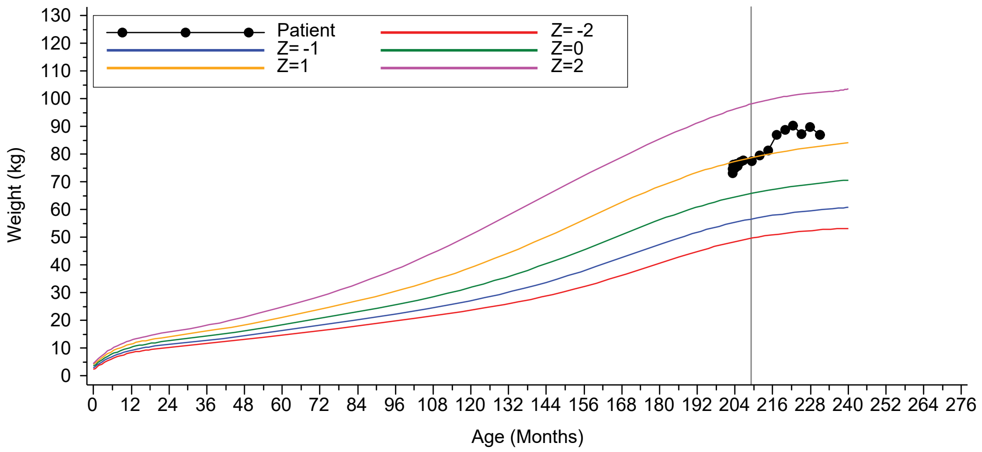

Patient 41
Seizure History: Partial Onset Seizures

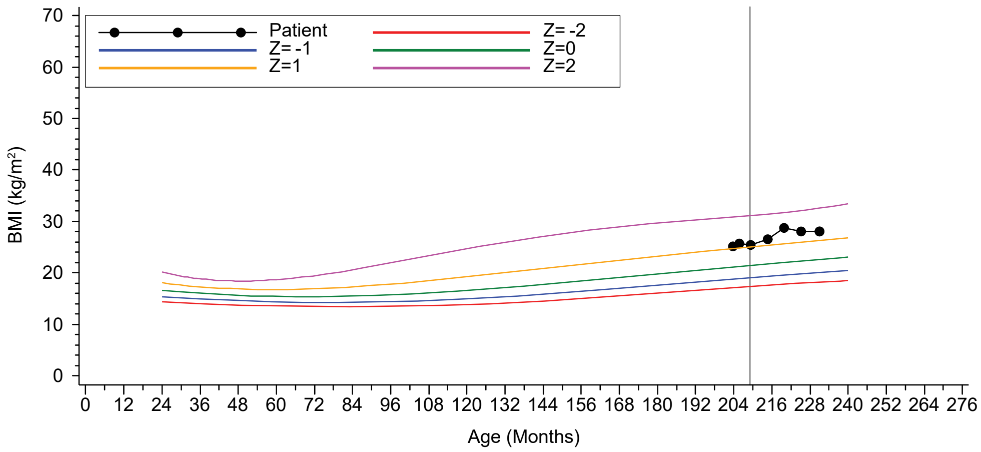

Patient 42
Seizure History: Partial Onset Seizures

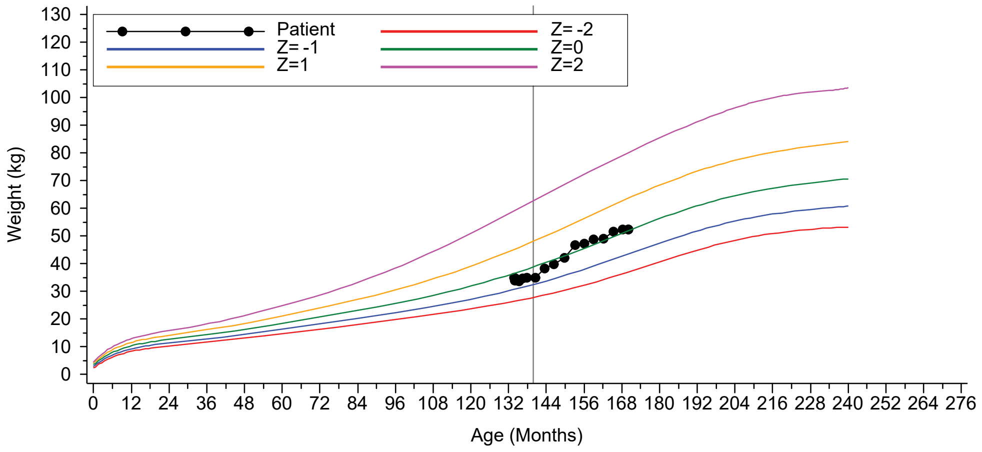

Patient 42
Seizure History: Partial Onset Seizures

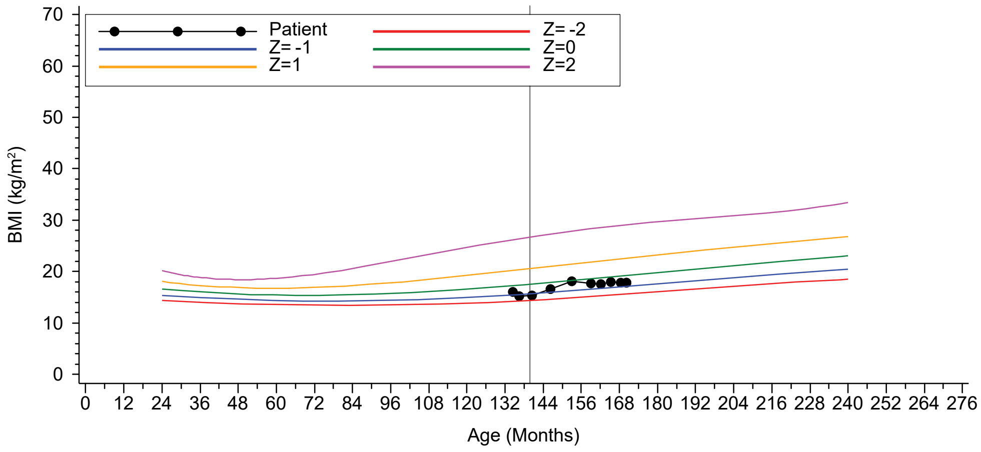

Patient 43
Seizure History: Partial Onset Seizures

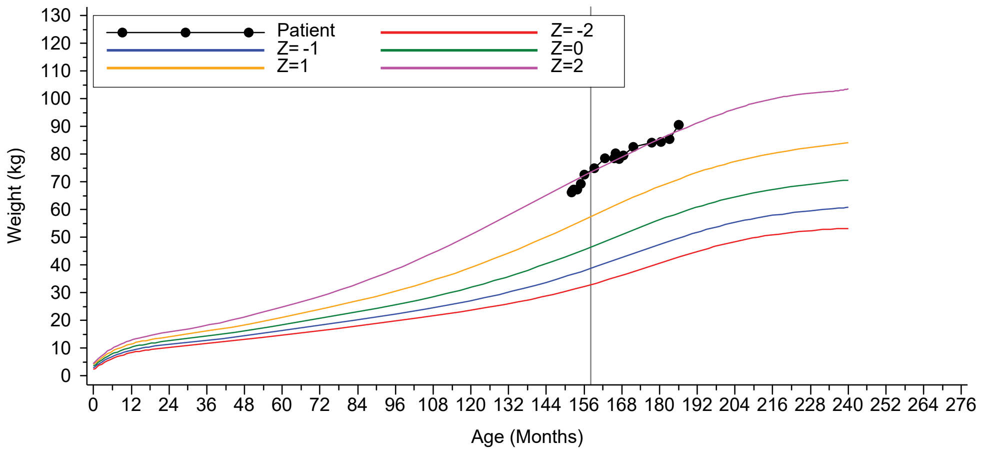

Patient 43
Seizure History: Partial Onset Seizures

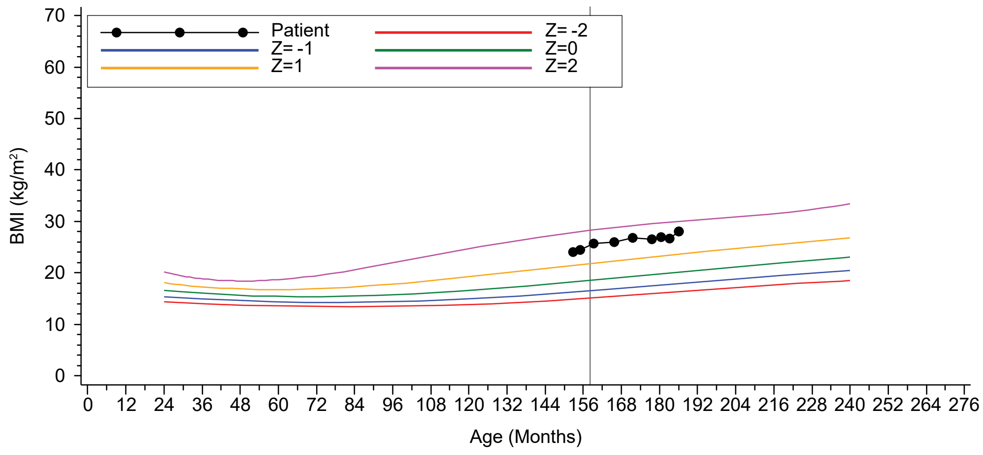

Patient 44
Seizure History: Partial Onset Seizures

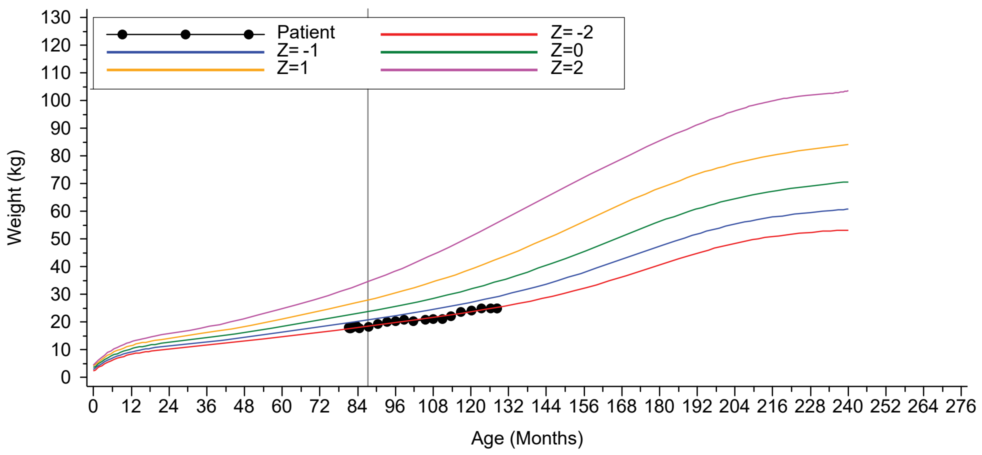

Patient 44
Seizure History: Partial Onset Seizures

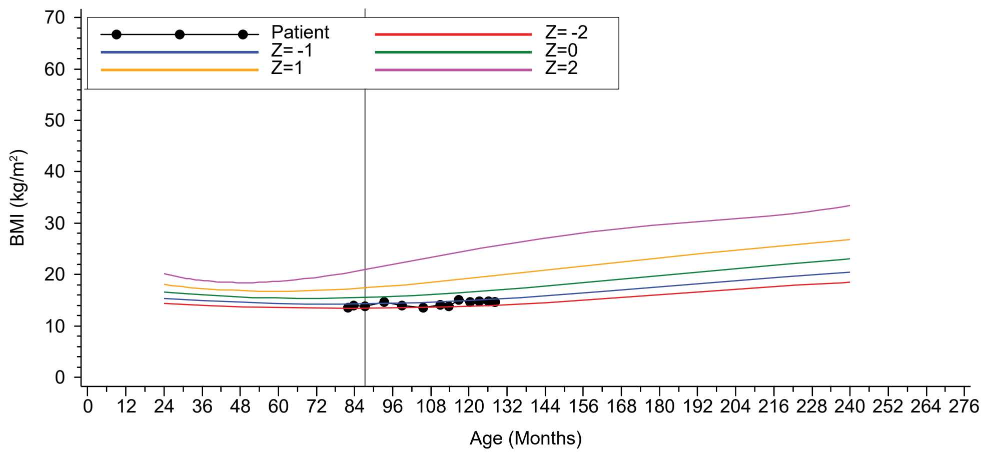

Patient 45
Seizure History: Partial Onset Seizures

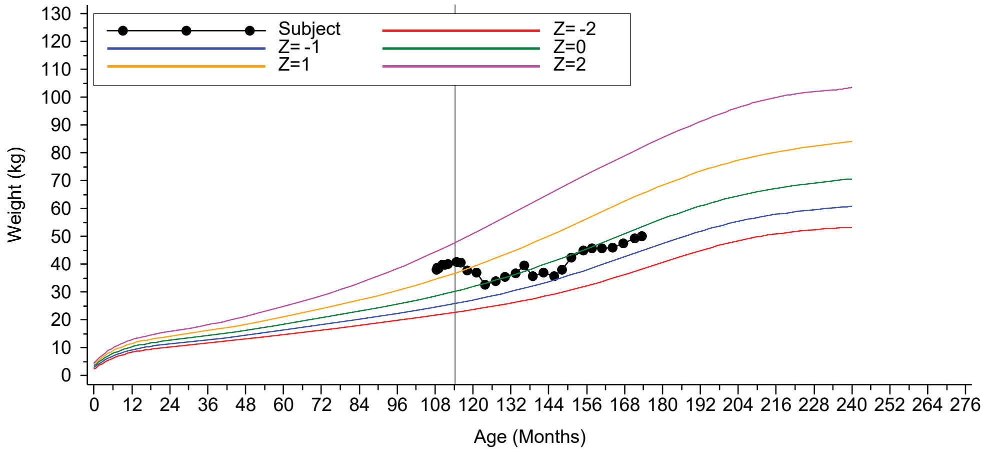

Patient 45
Seizure History: Partial Onset Seizures

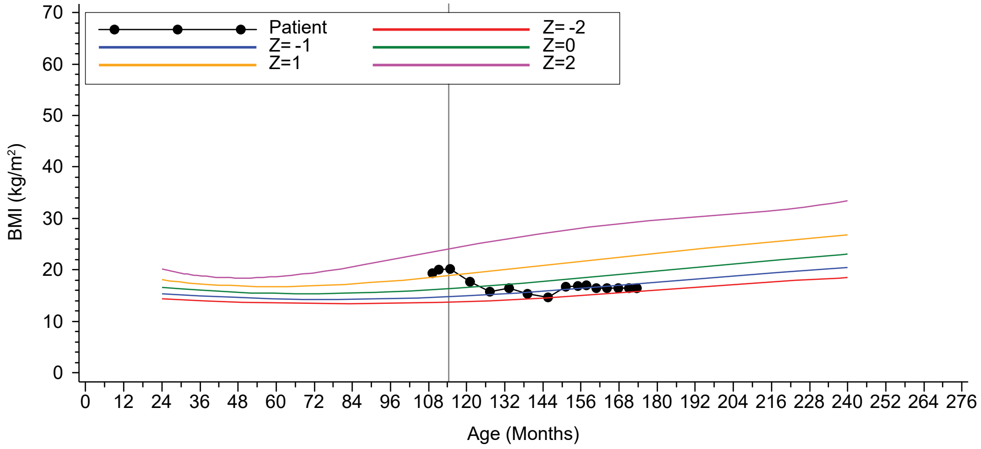

Patient 46
Seizure History: Partial Onset Seizures

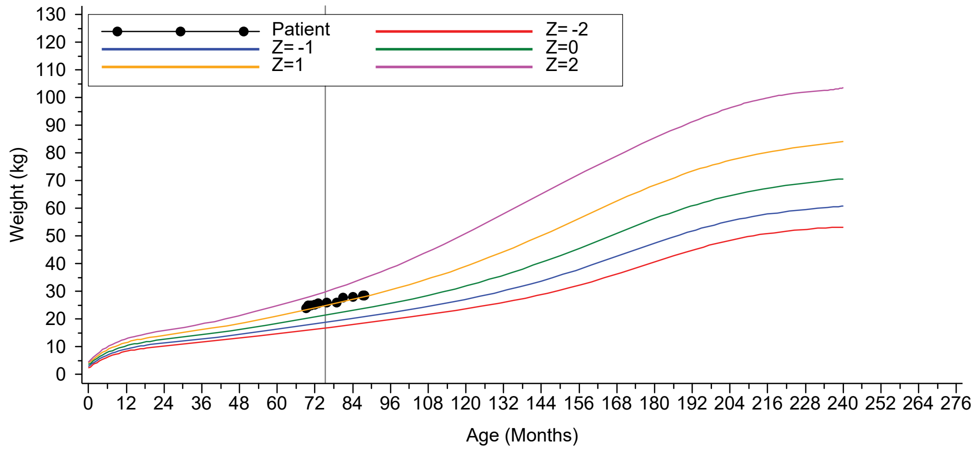

Patient 46
Seizure History: Partial Onset Seizures

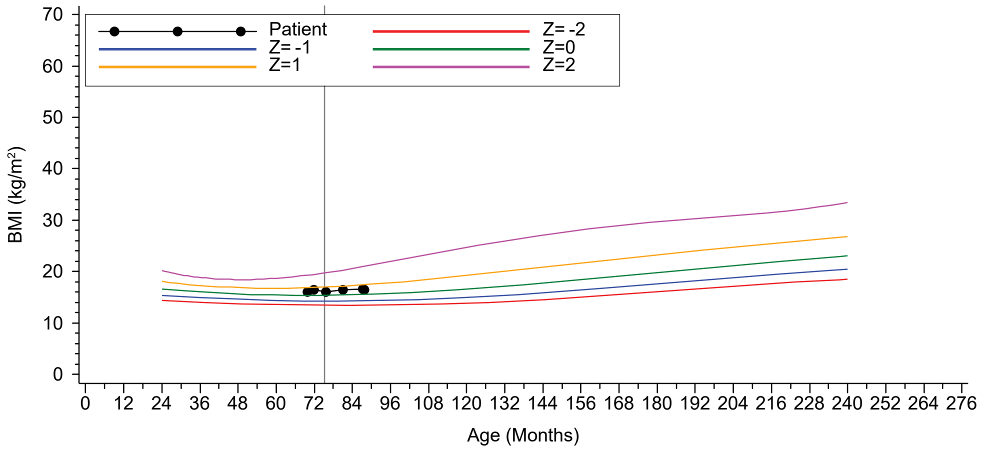

Patient 47
Seizure History: Partial Onset Seizures

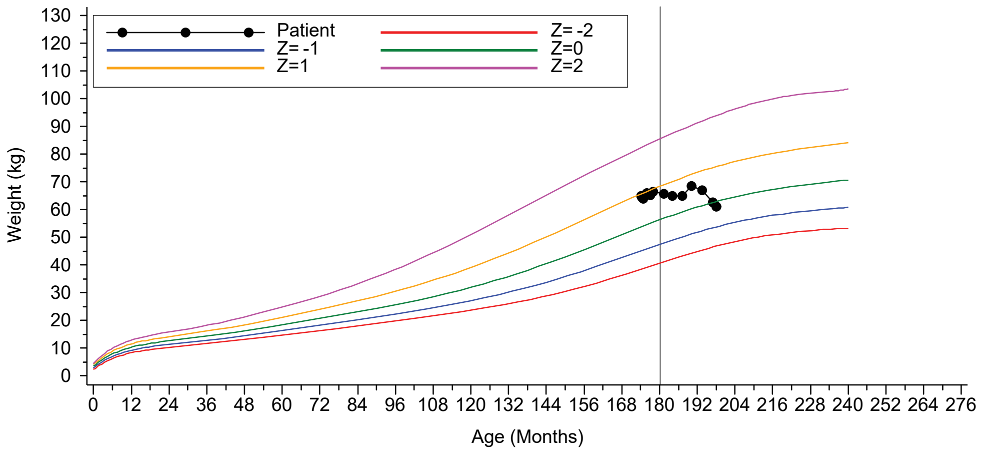

Patient 47
Seizure History: Partial Onset Seizures

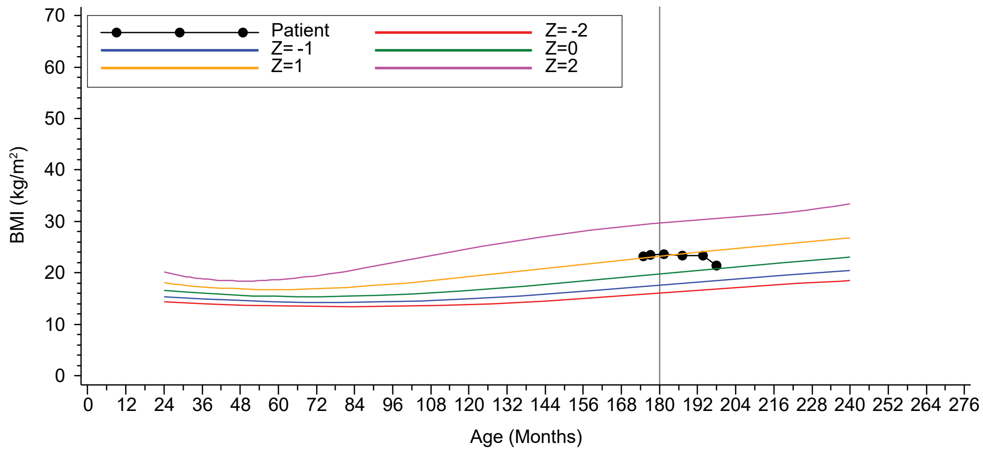

Patient 48
Seizure History: Partial Onset Seizures

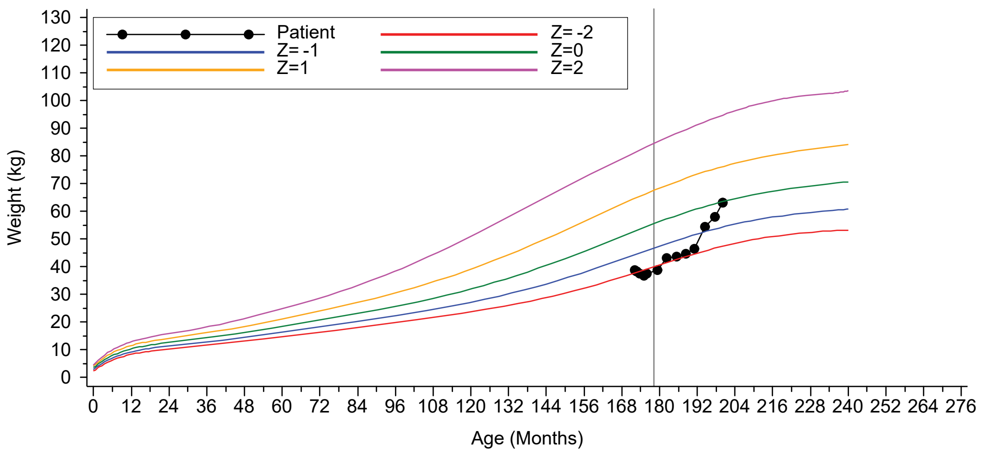

Patient 48
Seizure History: Partial Onset Seizures

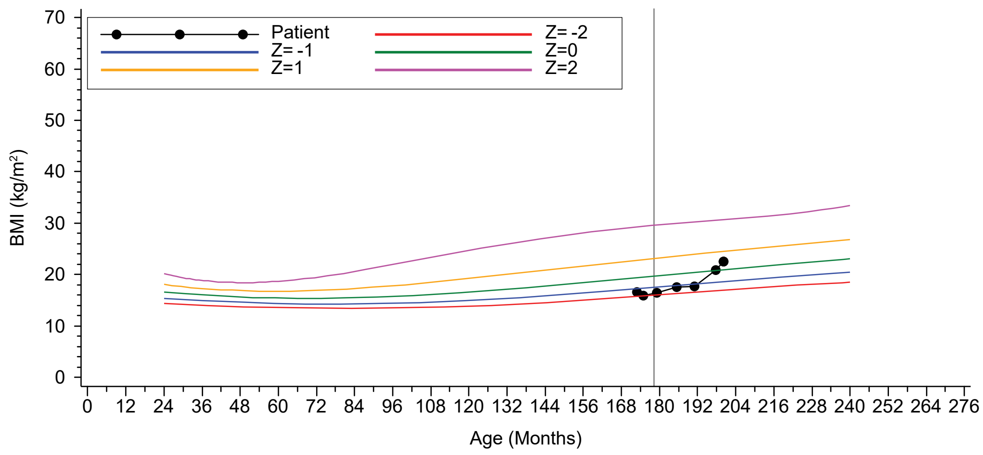

Patient 49
Seizure History: Partial Onset Seizures

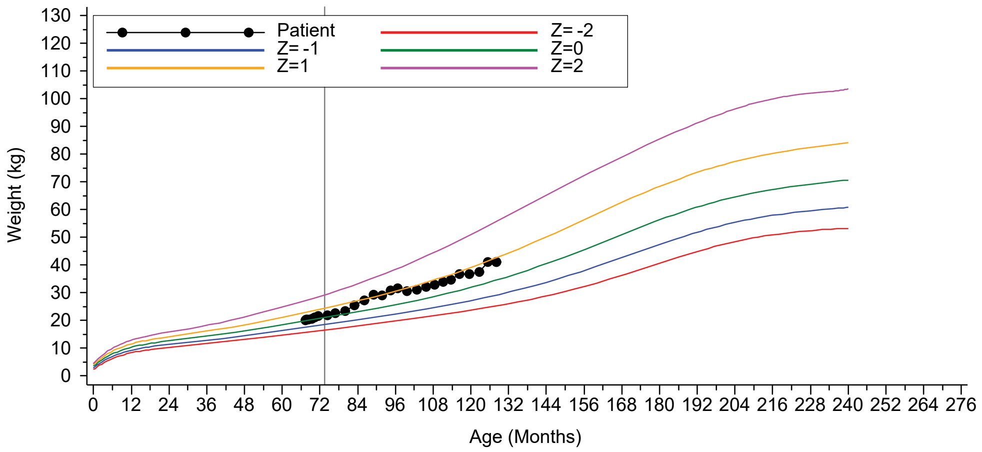

Patient 49
Seizure History: Partial Onset Seizures

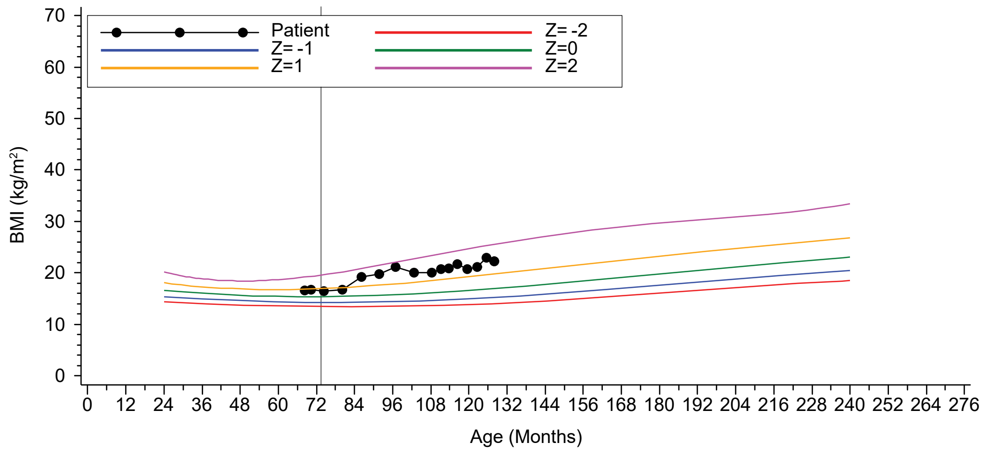

Patient 50
Seizure History: Partial Onset Seizures

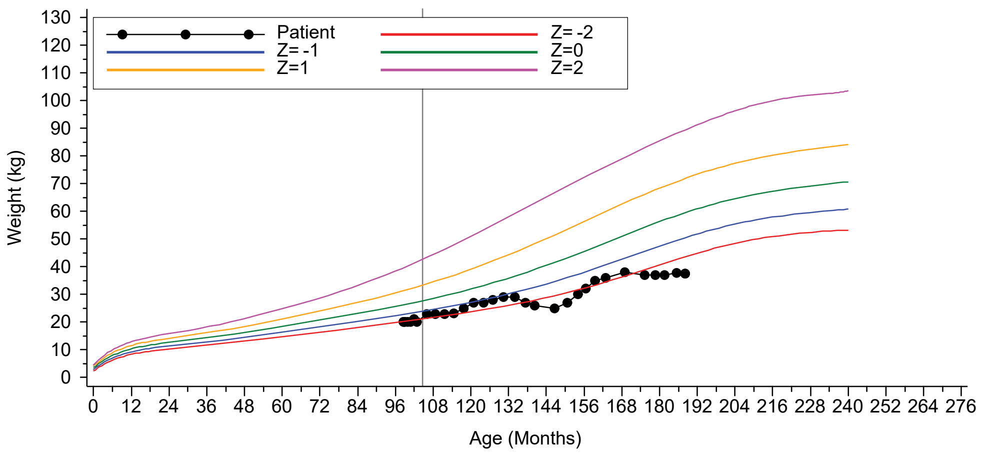

Patient 50
Seizure History: Partial Onset Seizures

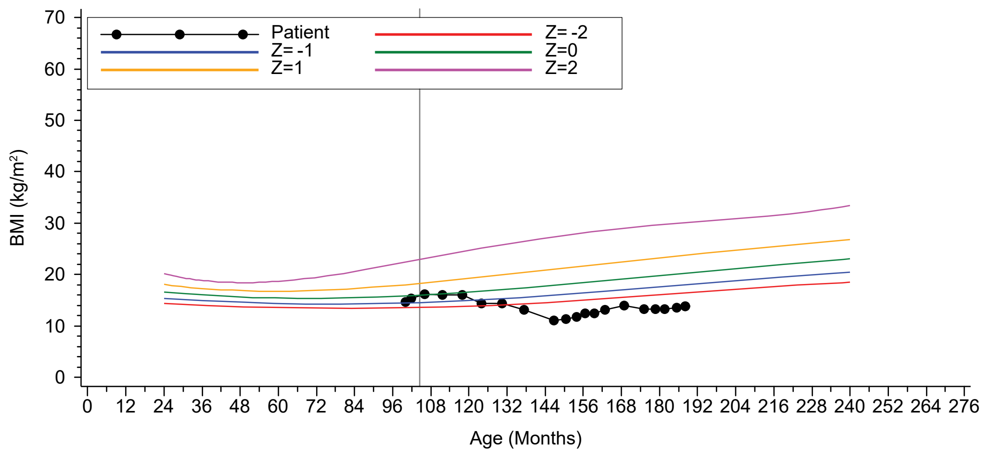

Patient 51
Seizure History: Partial Onset Seizures

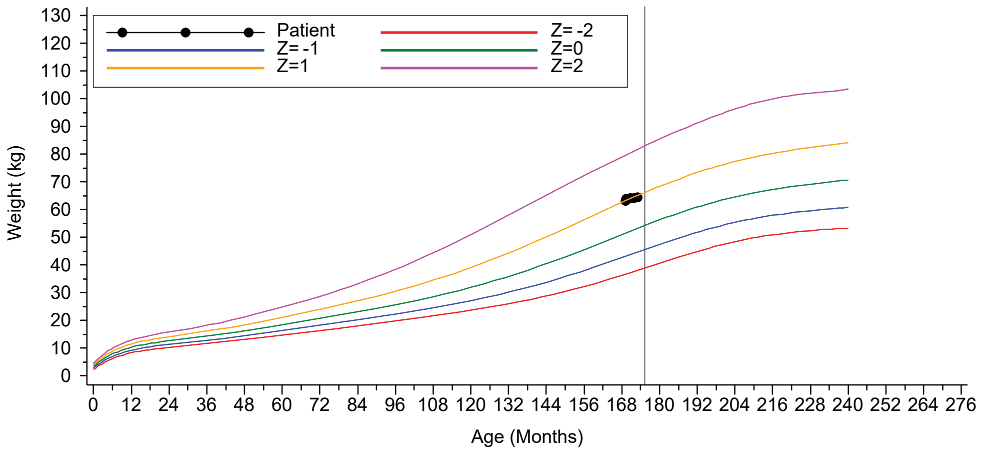

Patient 51
Seizure History: Partial Onset Seizures

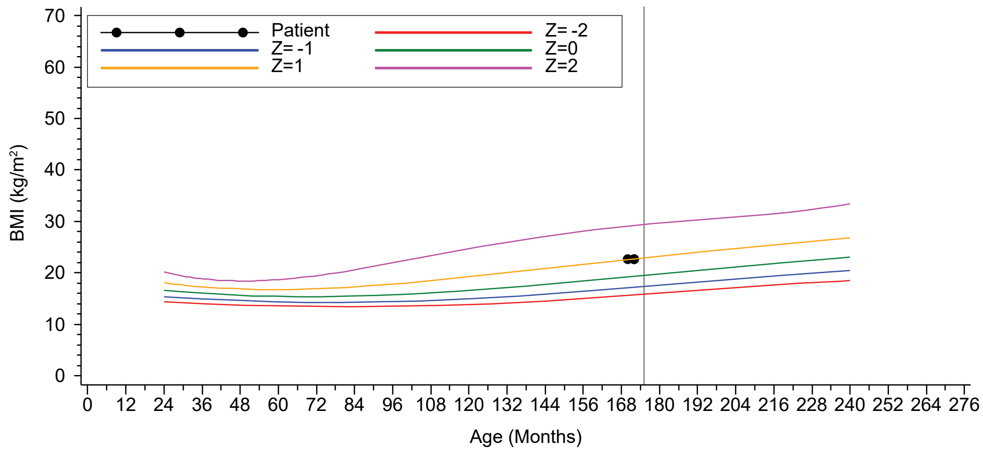

Patient 52
Seizure History: Partial Onset Seizures

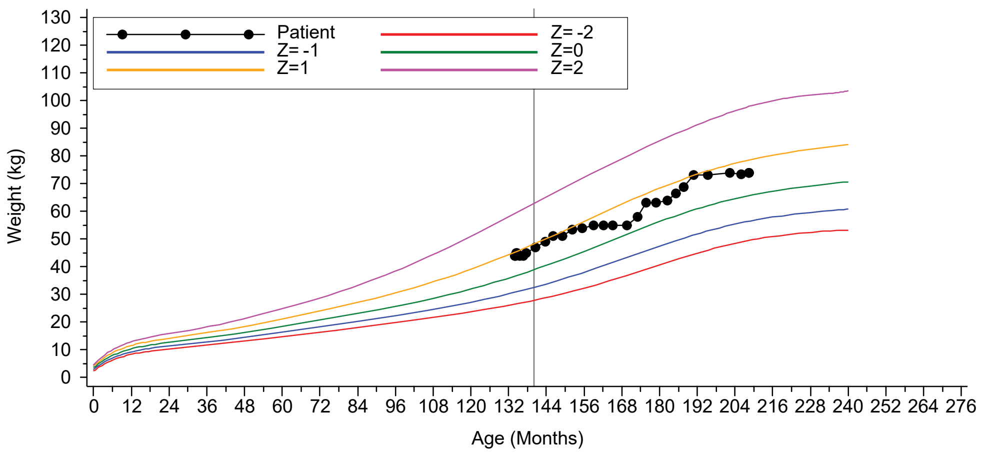

Patient 52
Seizure History: Partial Onset Seizures

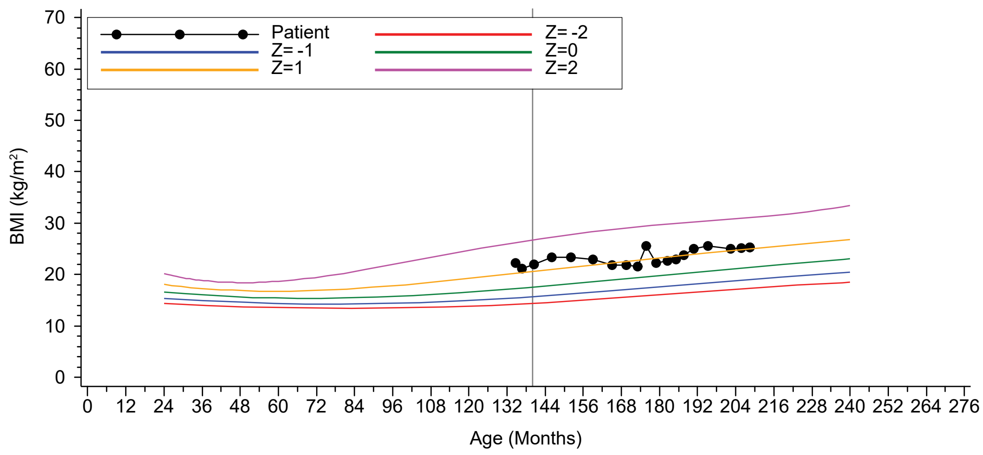

Patient 53
Seizure History: Partial Onset Seizures

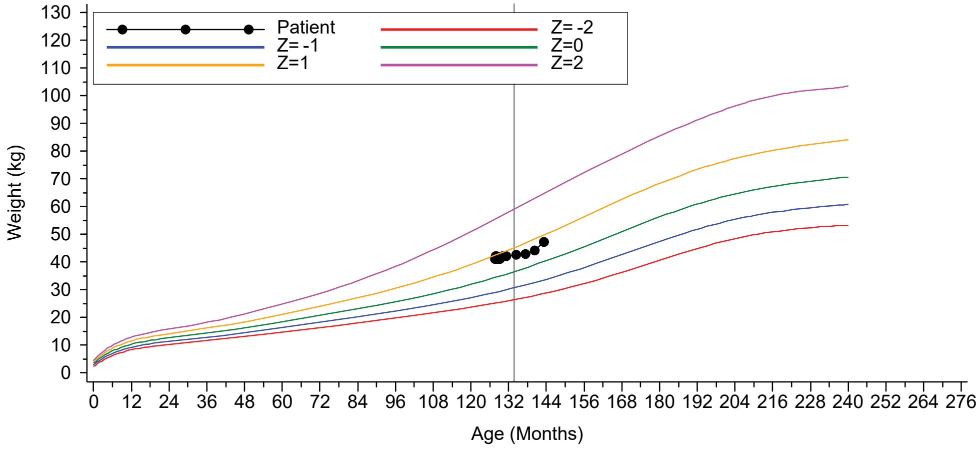

Patient 53
Seizure History: Partial Onset Seizures

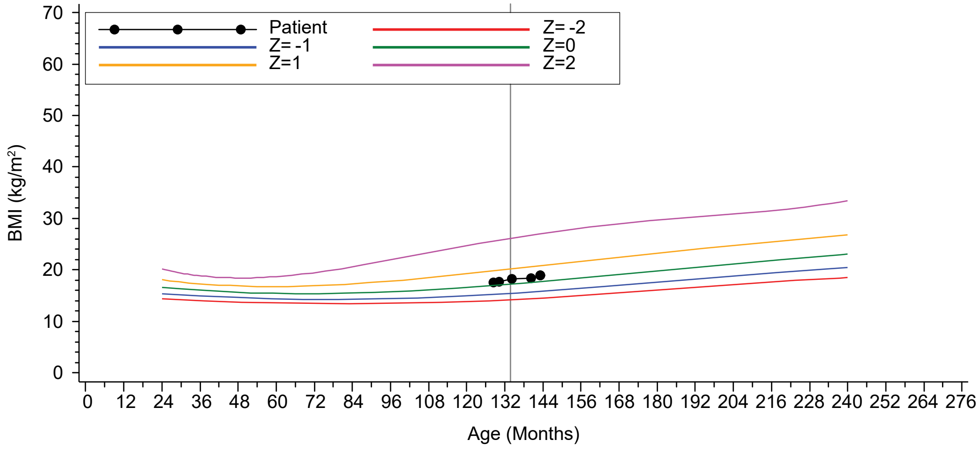

Patient 54
Seizure History: Partial Onset Seizures

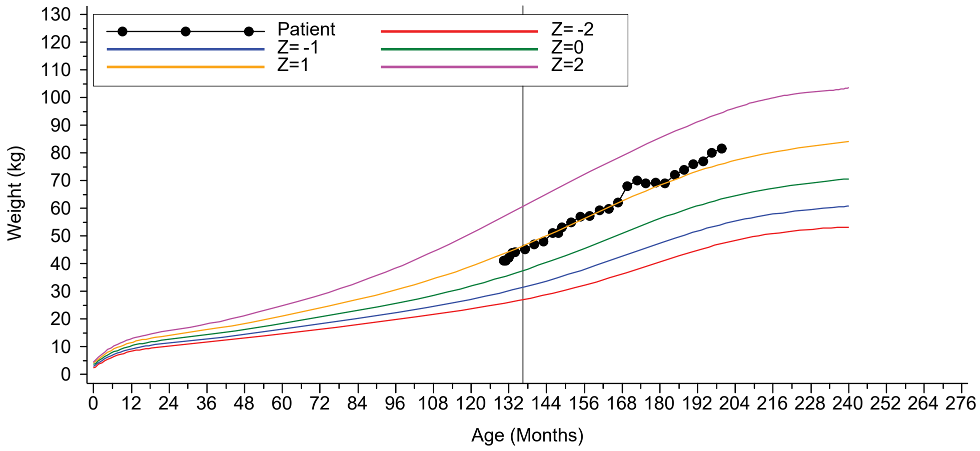

Patient 54
Seizure History: Partial Onset Seizures

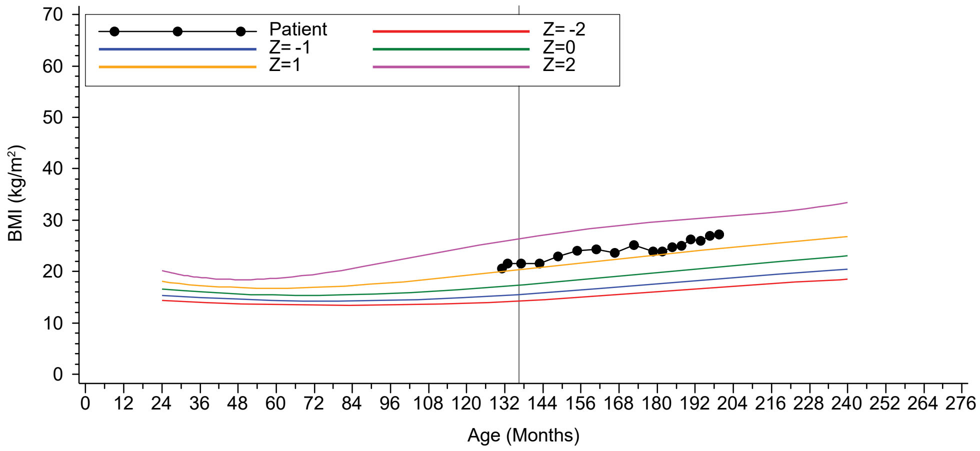

Patient 55
Seizure History: Partial Onset Seizures

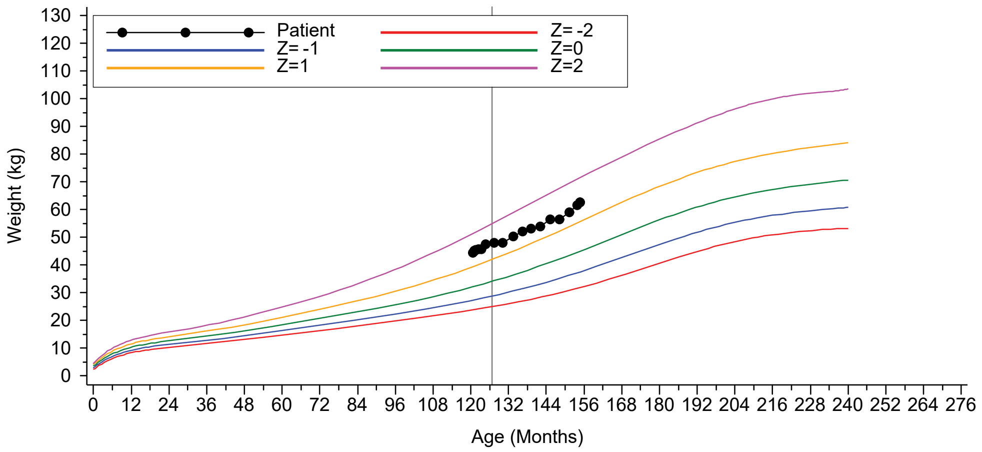

Patient 55
Seizure History: Partial Onset Seizures

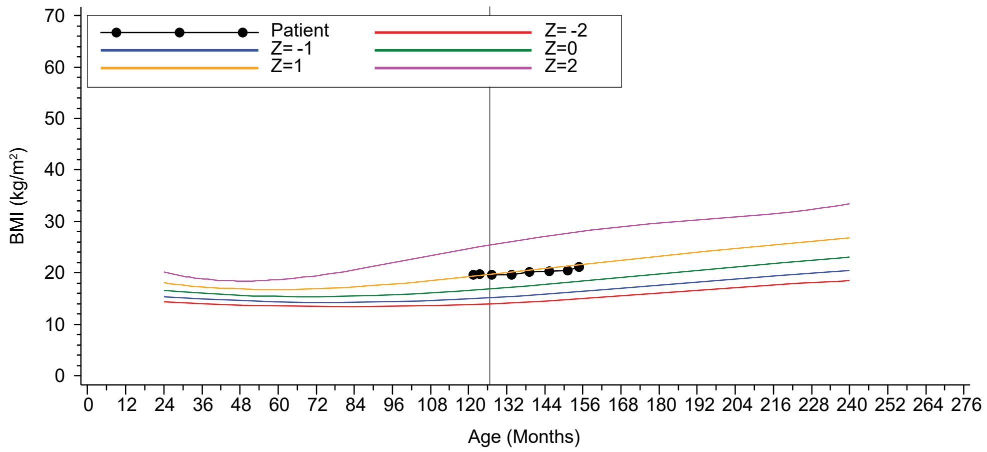

Patient 56
Seizure History: Partial Onset Seizures

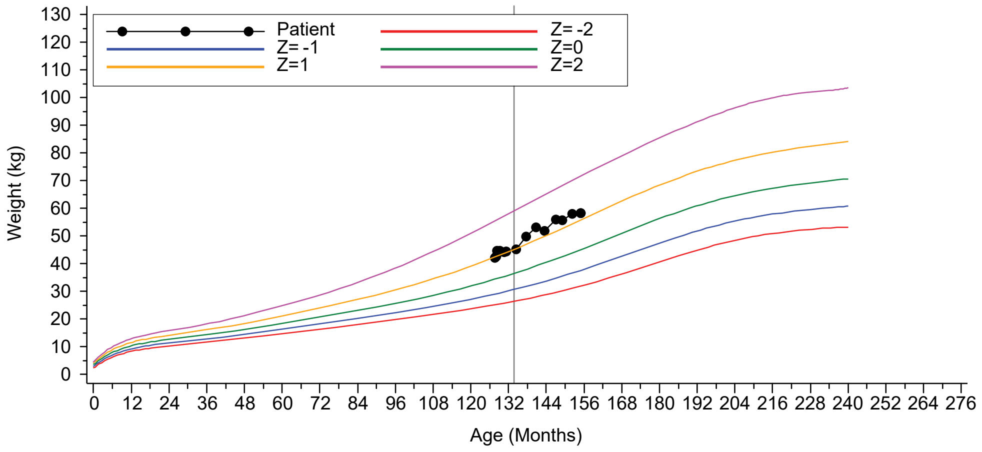

Patient 56
Seizure History: Partial Onset Seizures

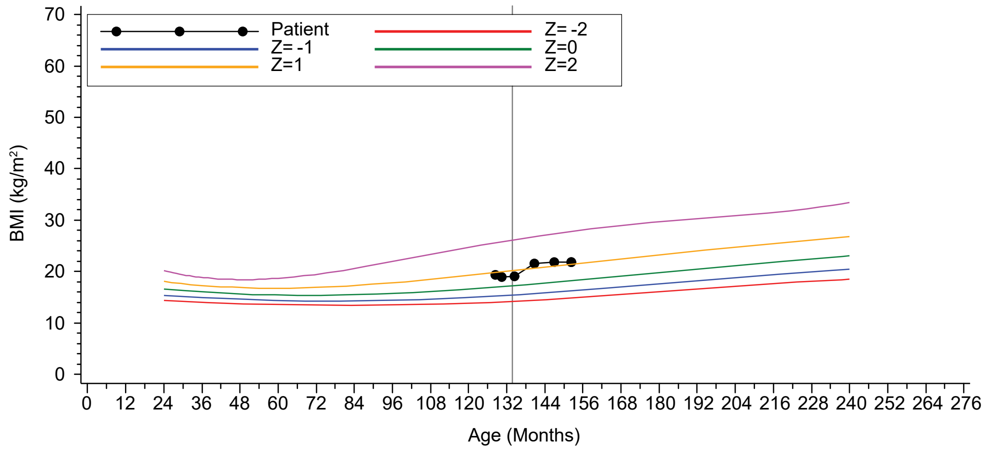

Patient 57
Seizure History: Partial Onset Seizures

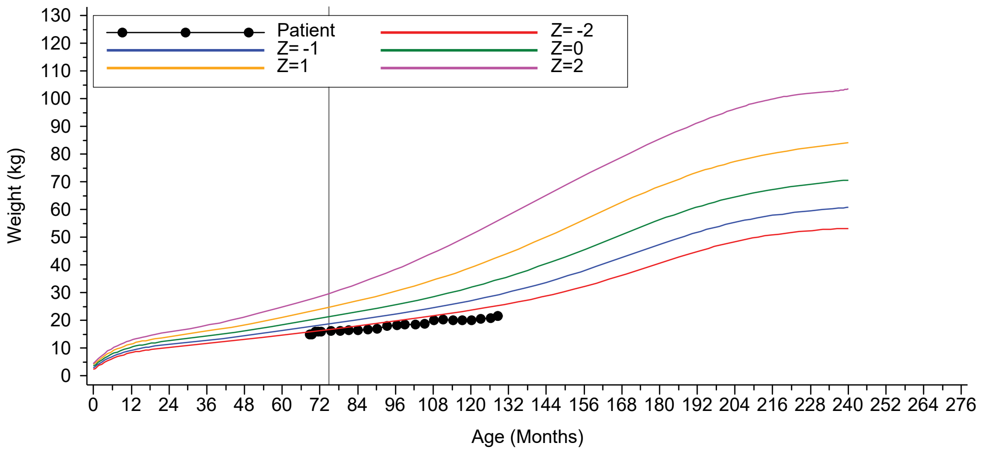

Patient 57
Seizure History: Partial Onset Seizures

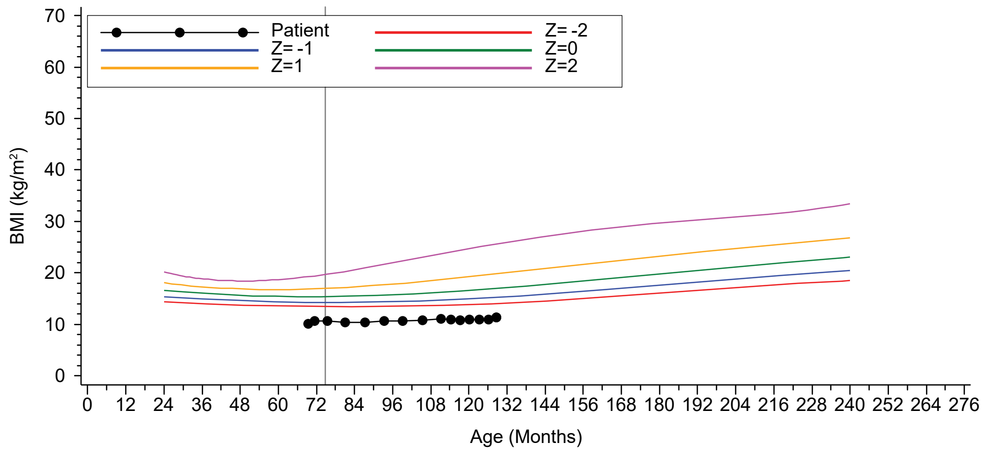

Patient 58
Seizure History: Partial Onset Seizures

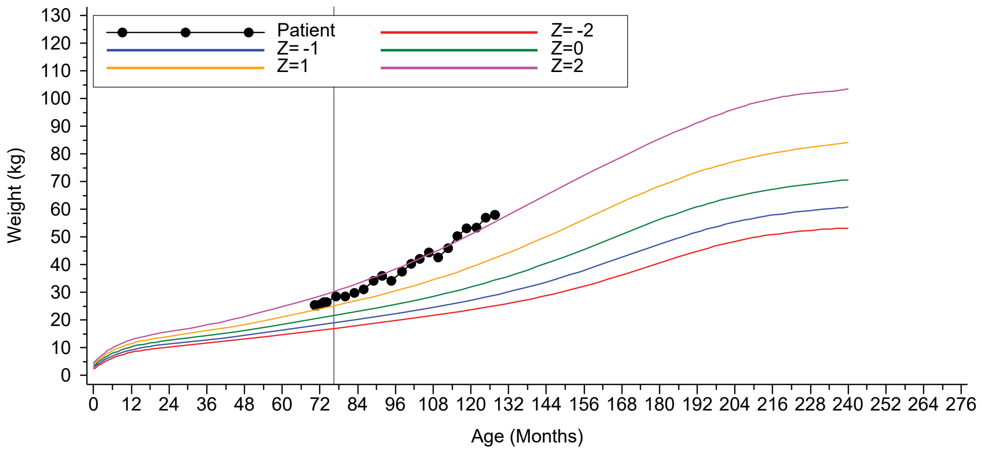

Patient 58
Seizure History: Partial Onset Seizures

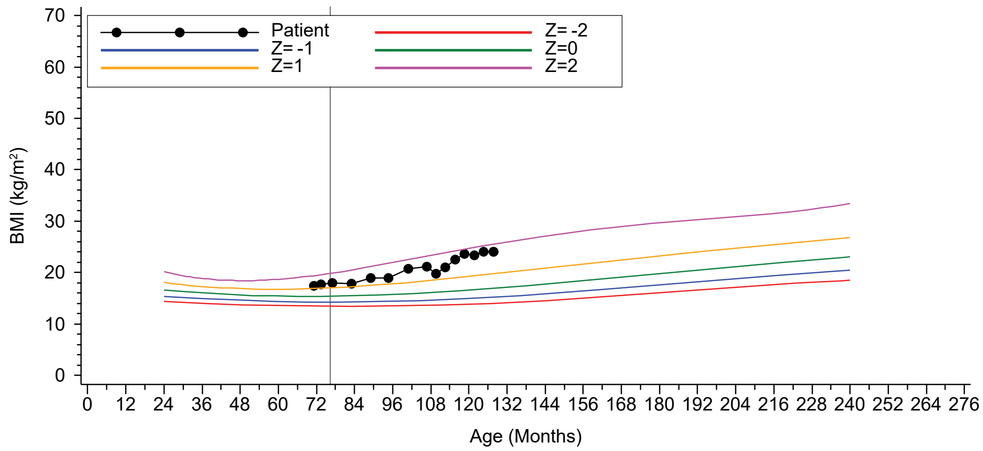

Patient 59
Seizure History: Partial Onset Seizures

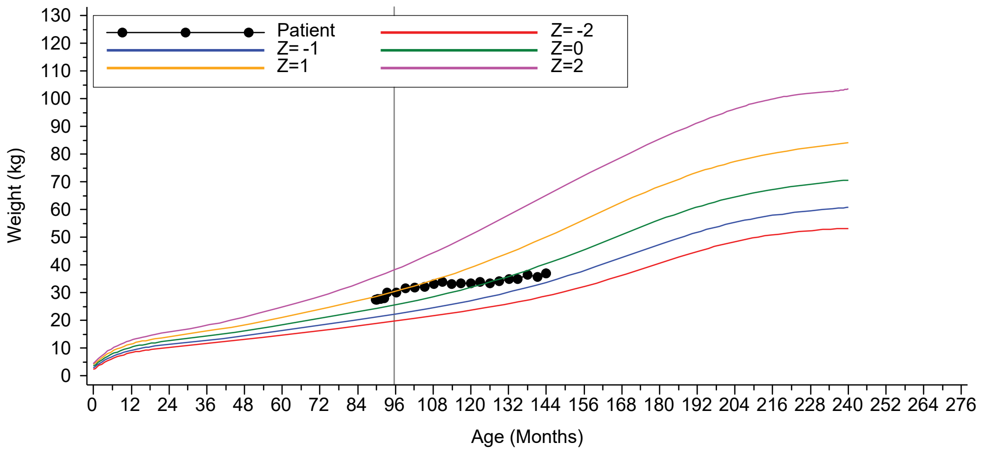

Patient 59
Seizure History: Partial Onset Seizures

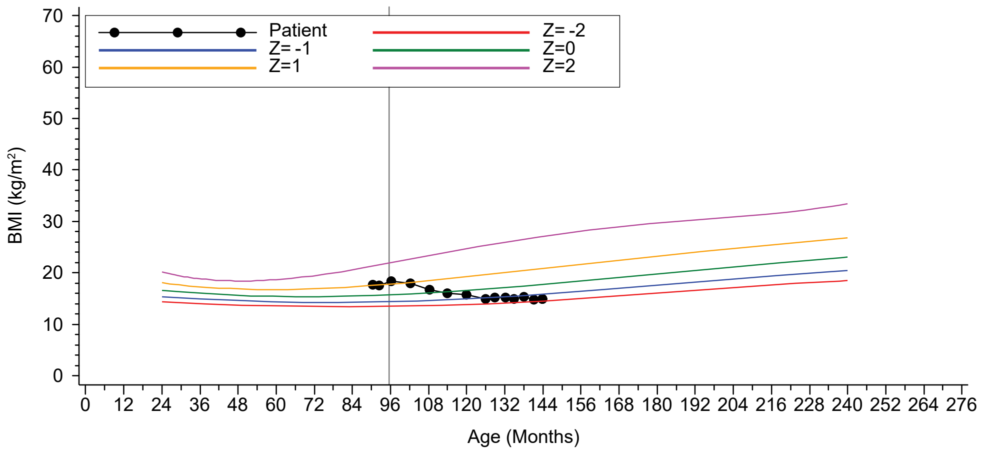

Patient 60
Seizure History: Partial Onset Seizures

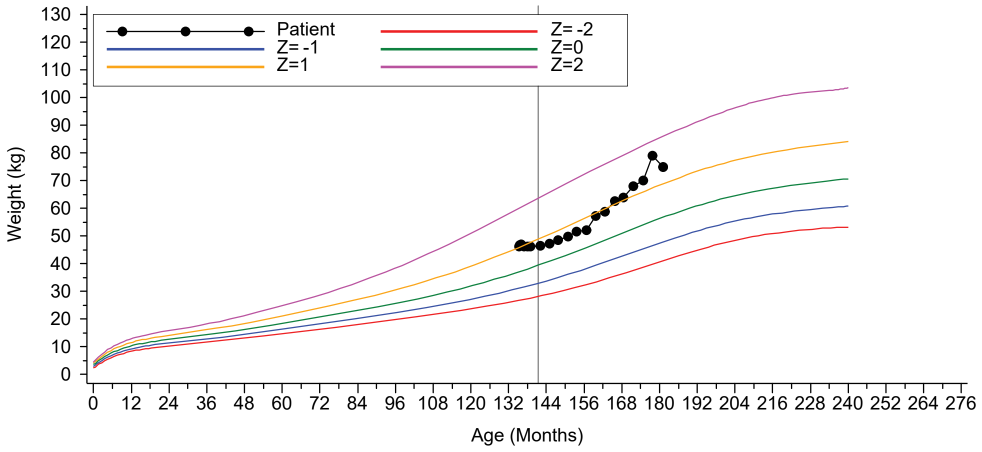

Patient 60
Seizure History: Partial Onset Seizures

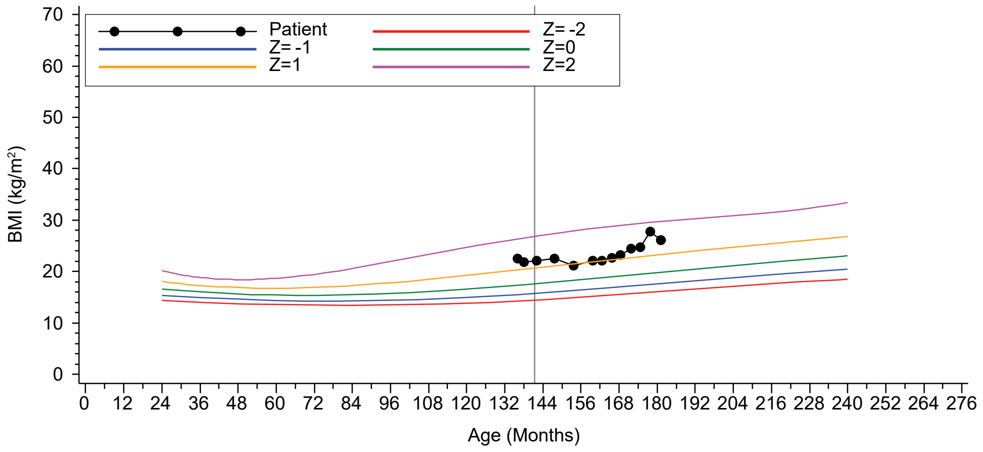

Patient 61

Seizure History: Partial Onset Seizures

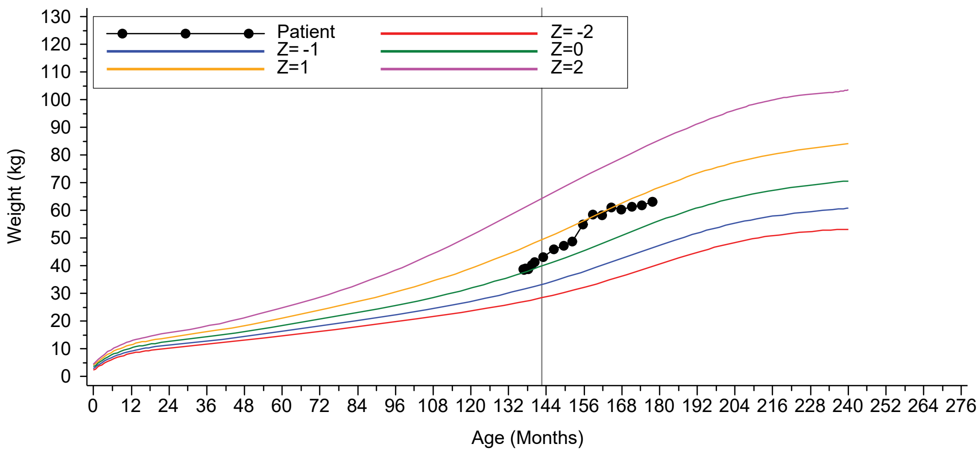

Patient 61

Seizure History: Partial Onset Seizures

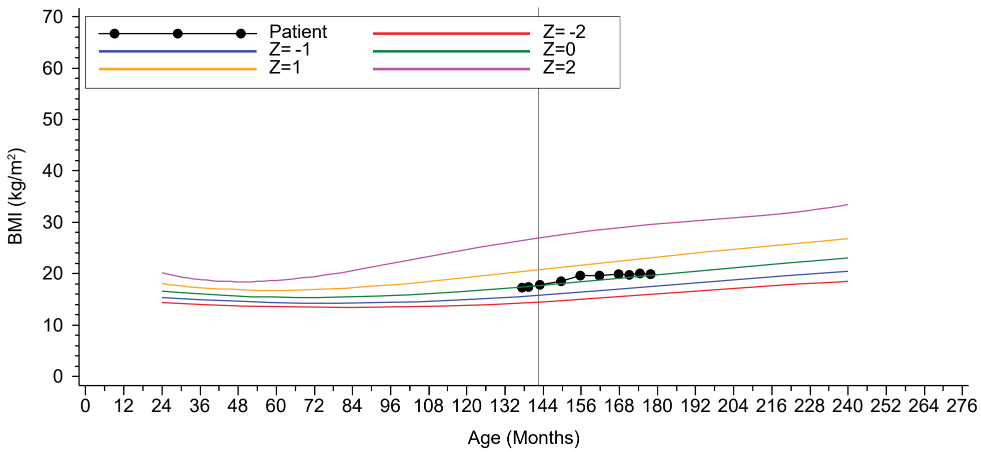

Patient 62

Seizure History: Partial Onset Seizures

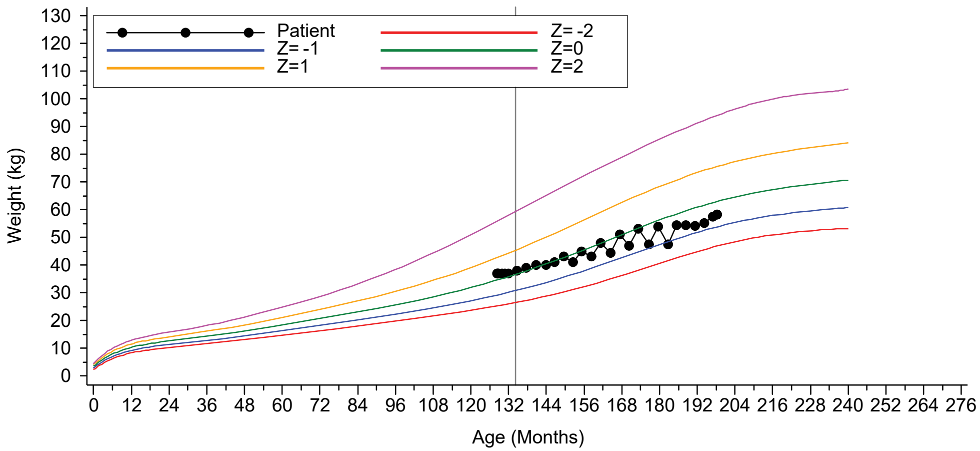

Patient 62

Seizure History: Partial Onset Seizures

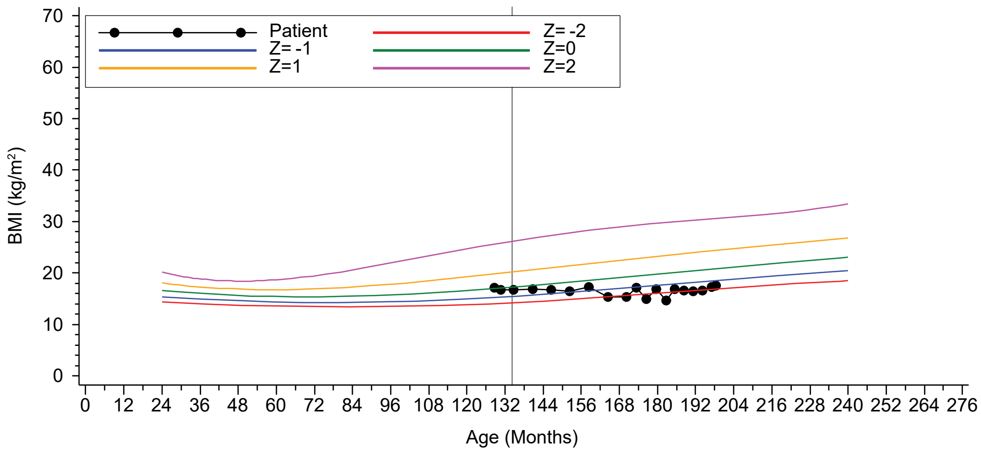

Patient 63

Seizure History: Partial Onset Seizures

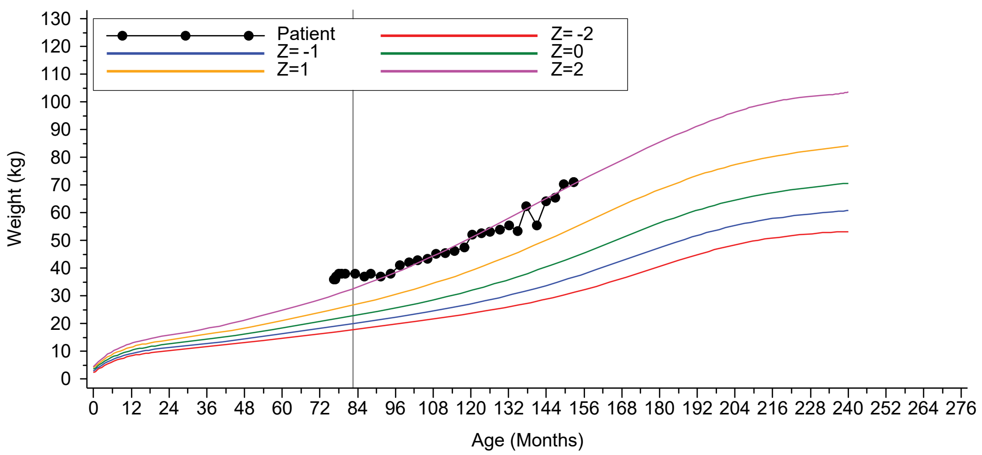

Patient 63

Seizure History: Partial Onset Seizures

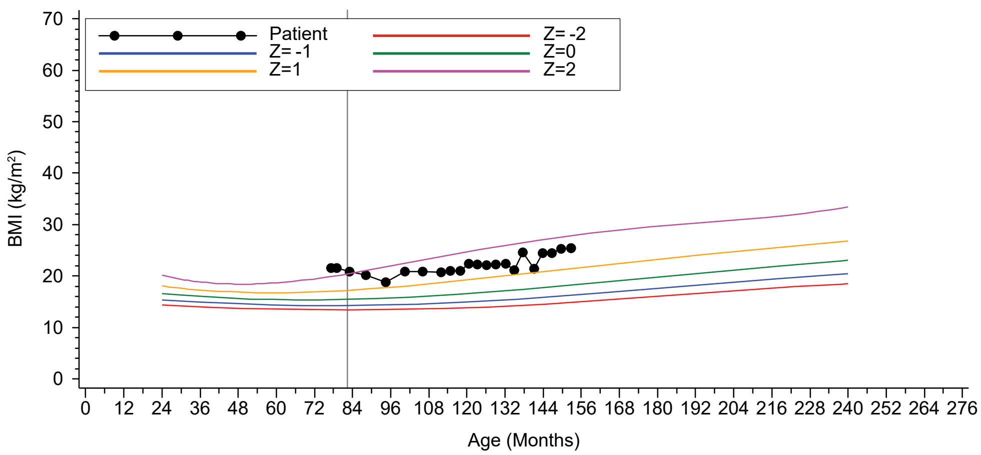

Patient 64

Seizure History: Partial Onset Seizures

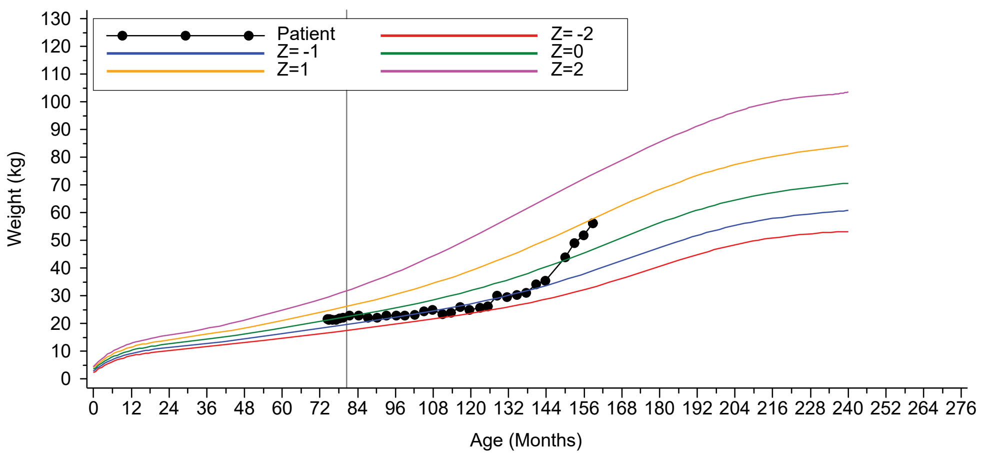

Patient 64

Seizure History: Partial Onset Seizures

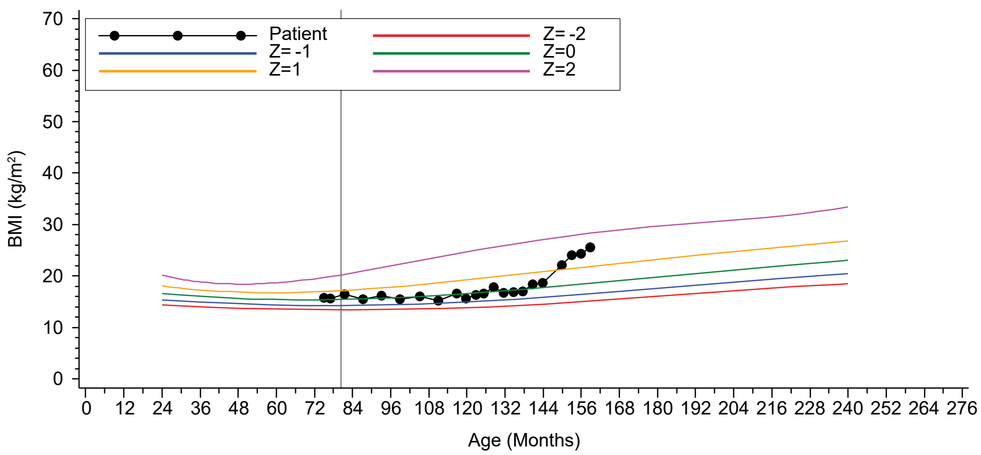

Patient 65

Seizure History: Partial Onset Seizures

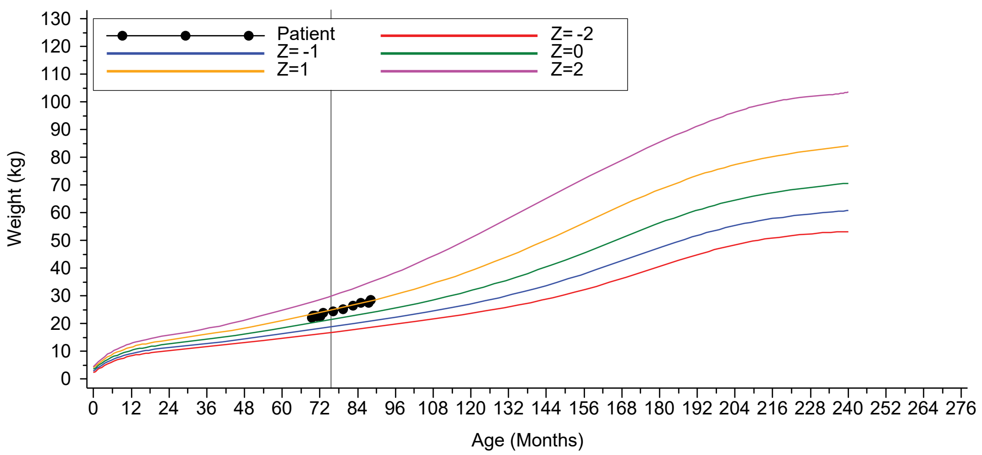

Patient 65

Seizure History: Partial Onset Seizures

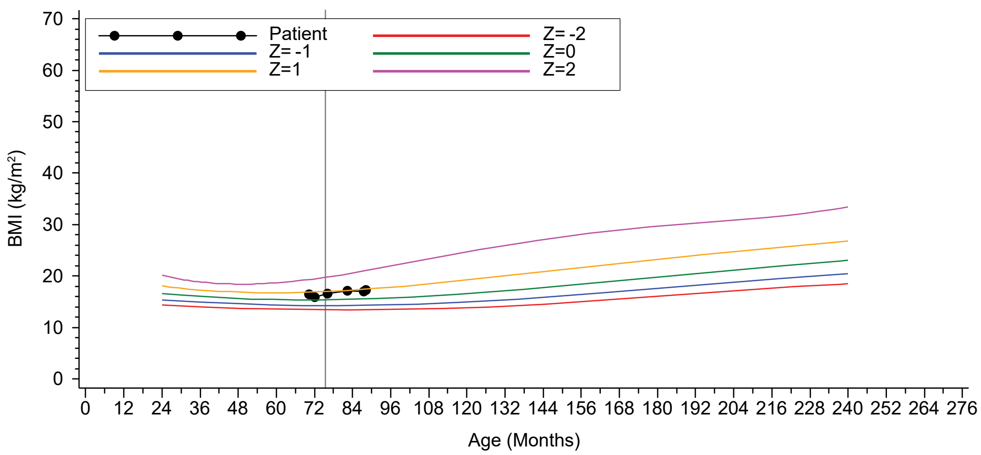

Patient 66

Seizure History: Partial Onset Seizures

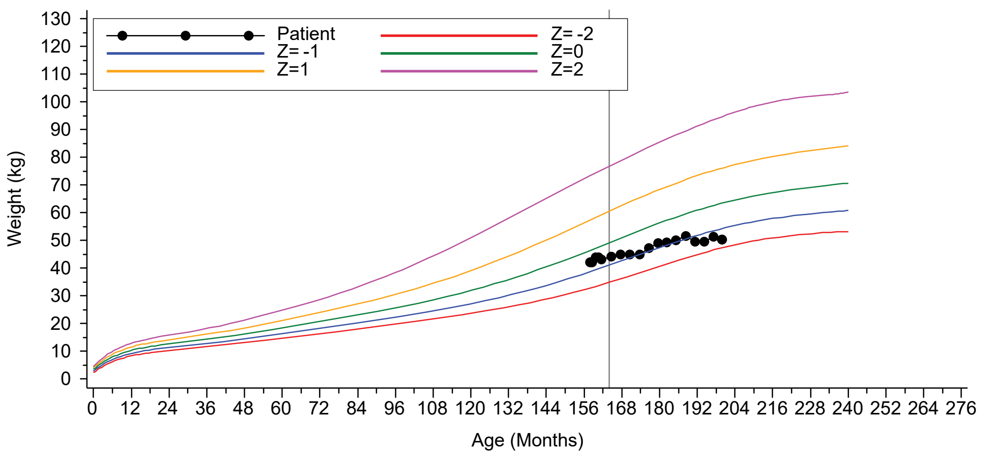

Patient 66

Seizure History: Partial Onset Seizures

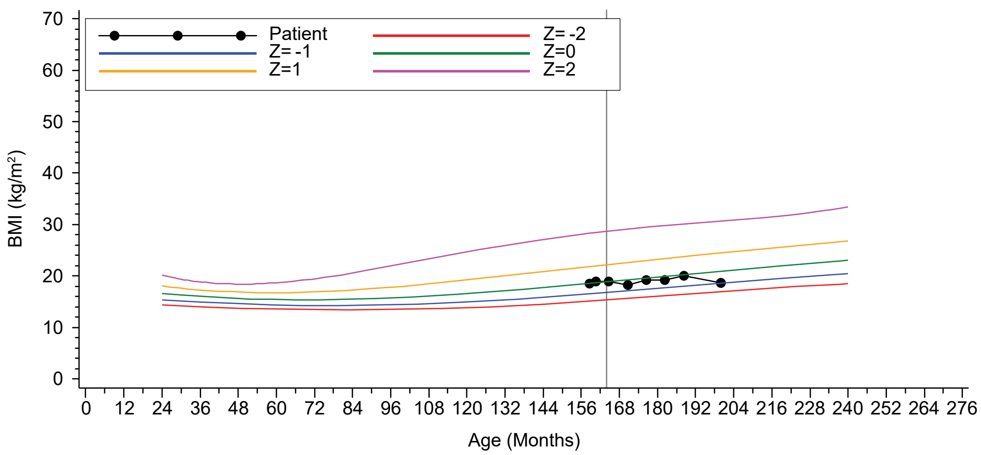

Patient 67

Seizure History: Partial Onset Seizures

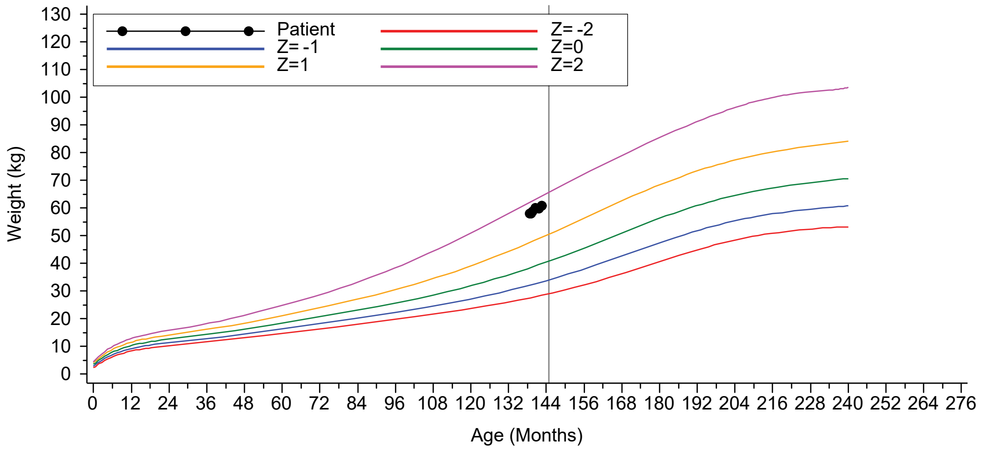

Patient 67

Seizure History: Partial Onset Seizures

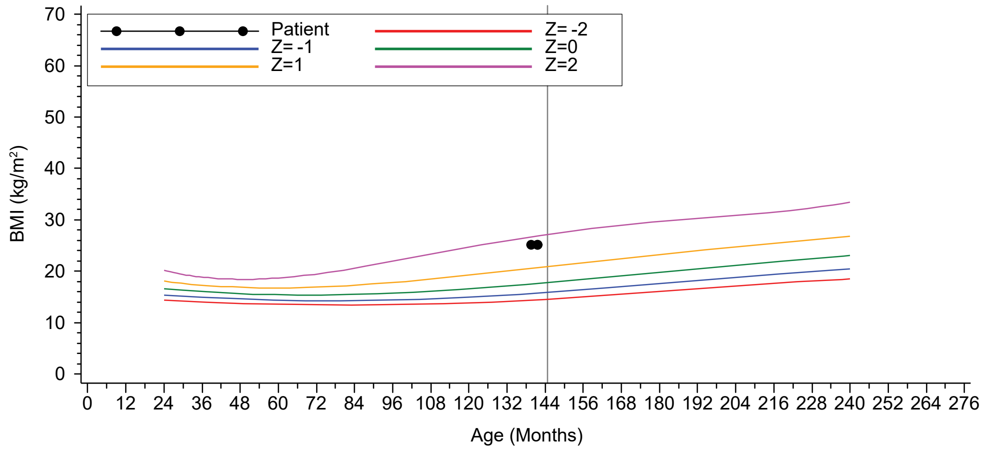

Patient 68

Seizure History: Partial Onset Seizures

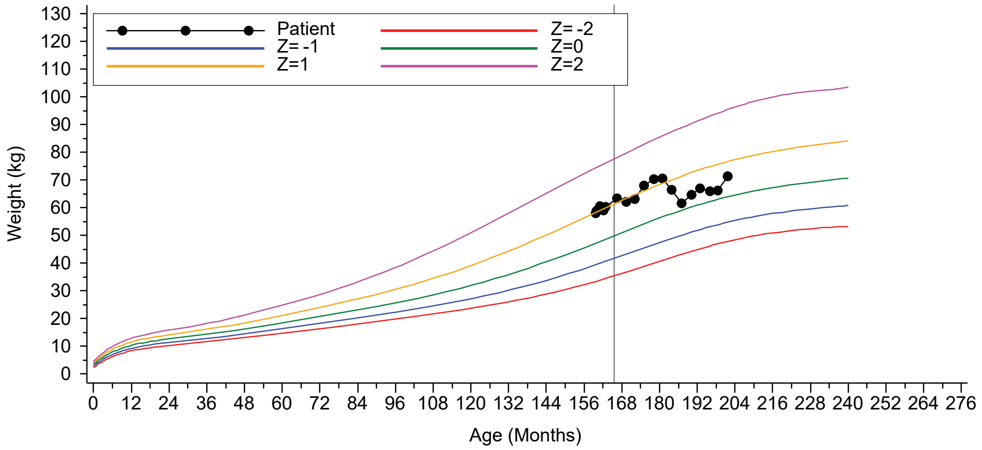

Patient 68

Seizure History: Partial Onset Seizures

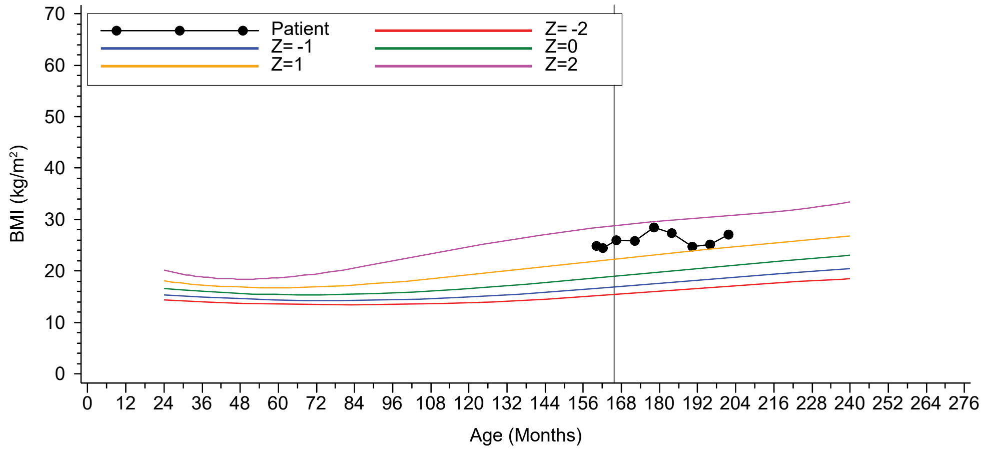

Patient 69

Seizure History: Partial Onset Seizures

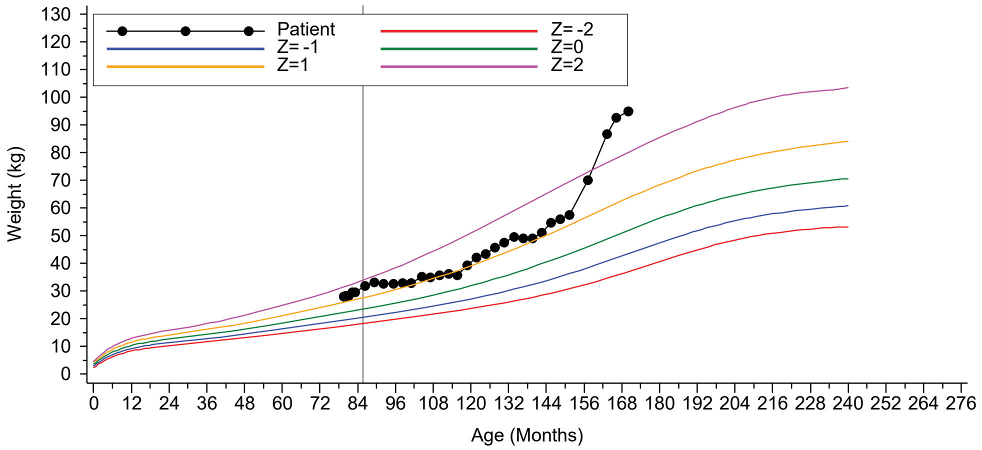

Patient 69

Seizure History: Partial Onset Seizures

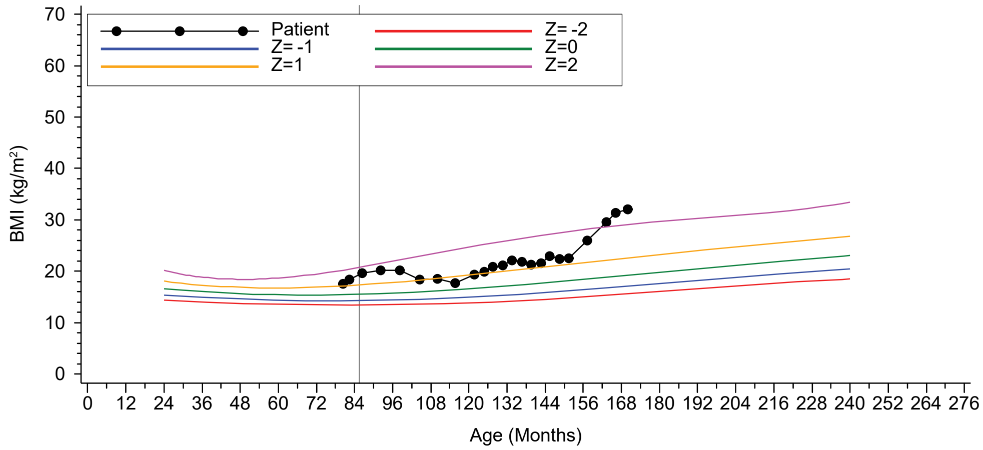

Patient 70

Seizure History: Partial Onset Seizures

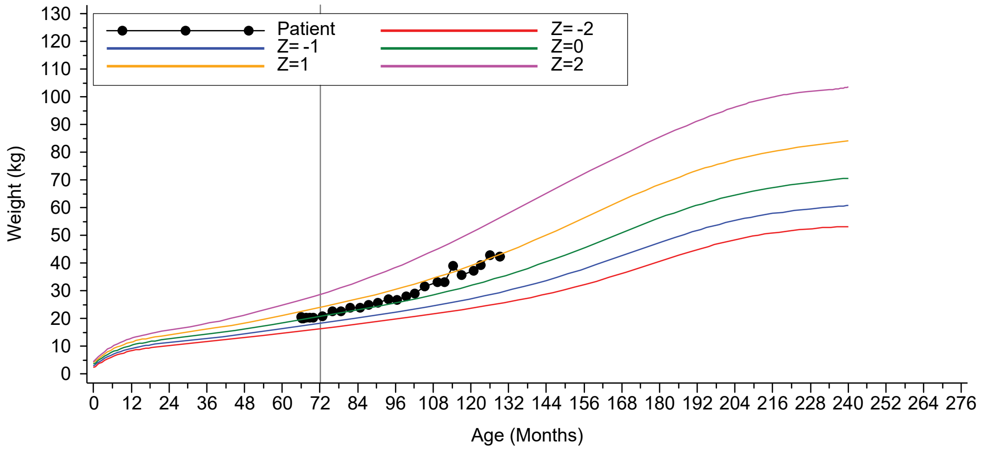

Patient 70

Seizure History: Partial Onset Seizures

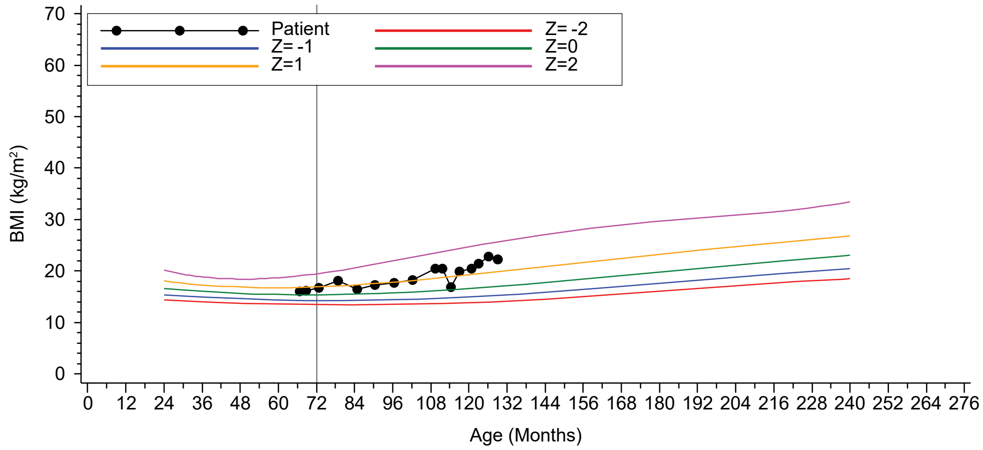

Patient 71

Seizure History: Partial Onset Seizures

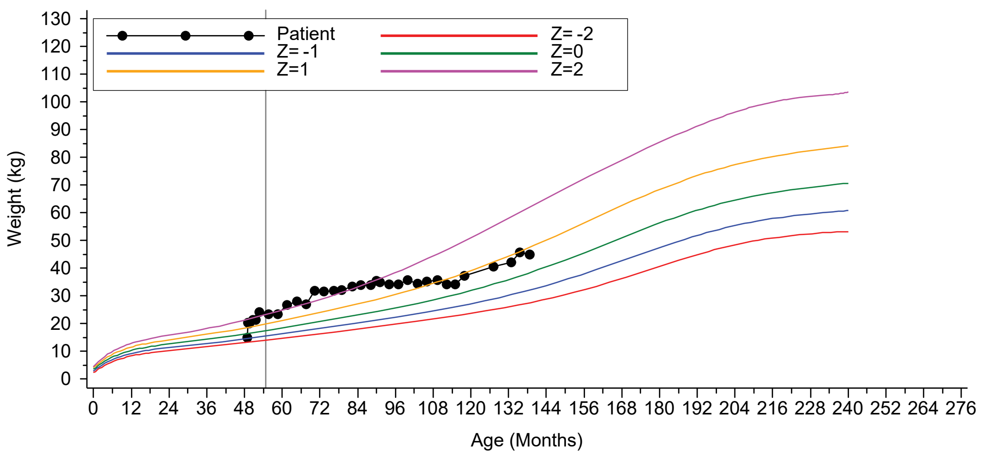

Patient 71

Seizure History: Partial Onset Seizures

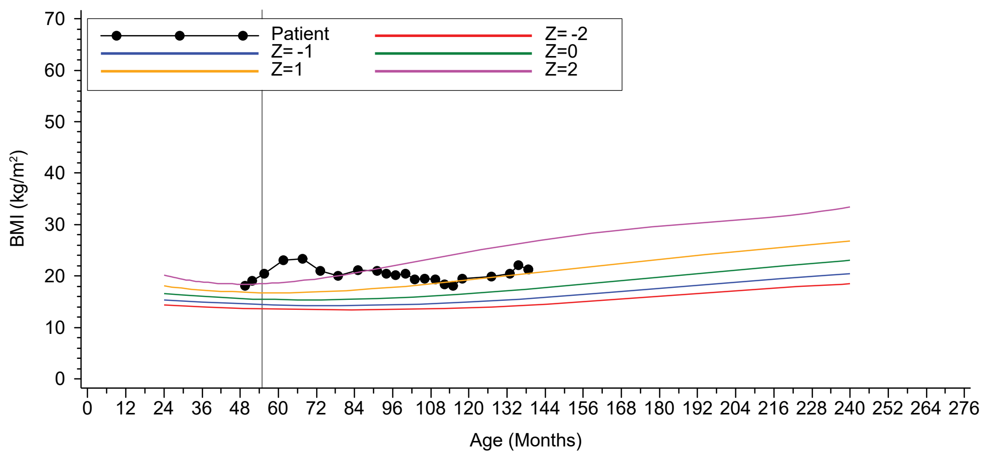

Patient 72

Seizure History: Partial Onset Seizures

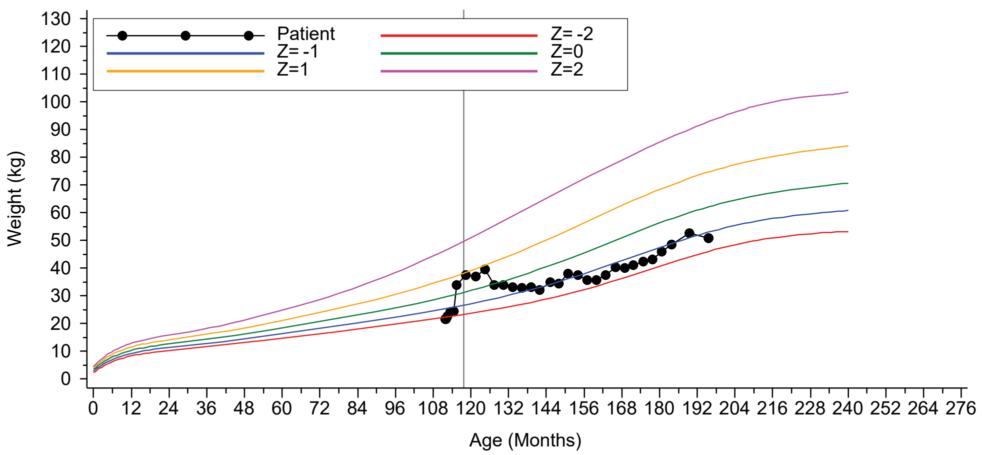

Patient 72

Seizure History: Partial Onset Seizures

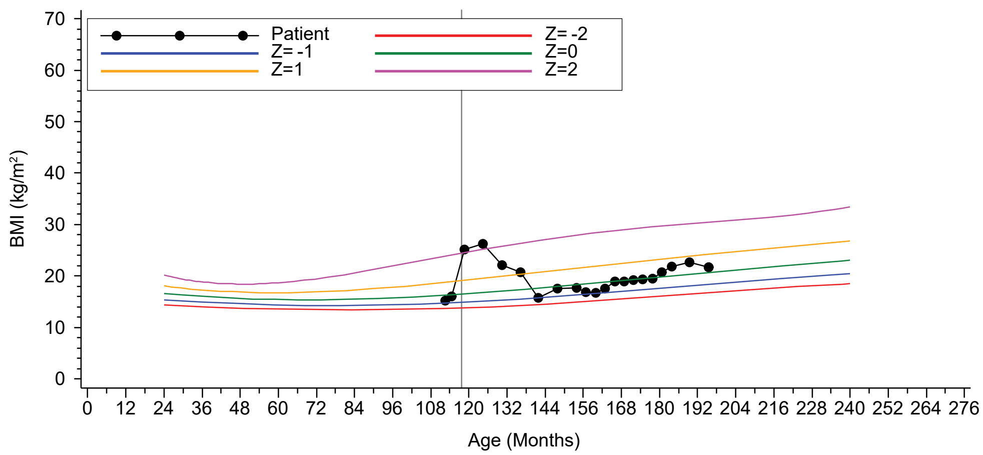

Patient 73

Seizure History: Partial Onset Seizures

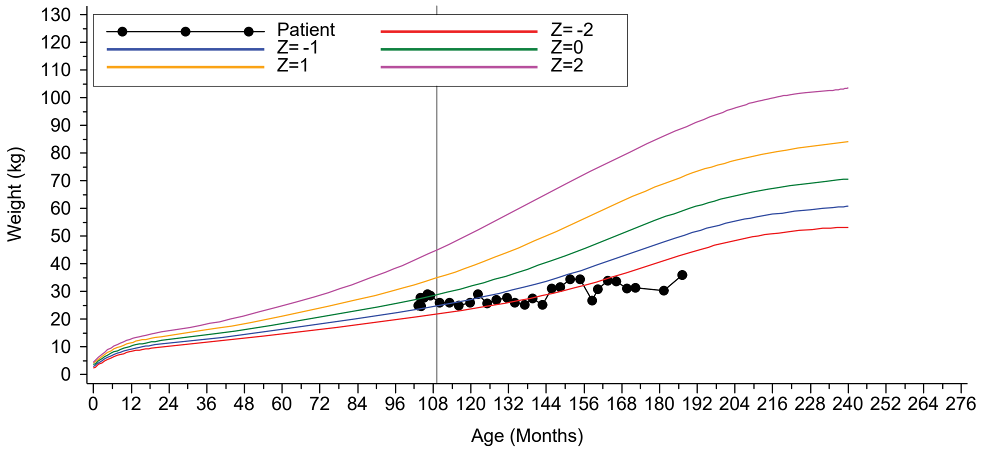

Patient 73

Seizure History: Partial Onset Seizures

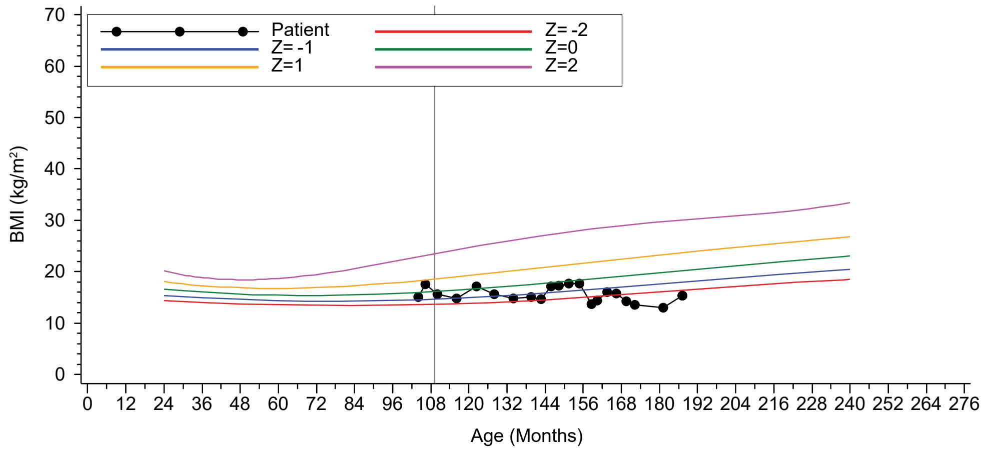

Patient 74

Seizure History: Partial Onset Seizures

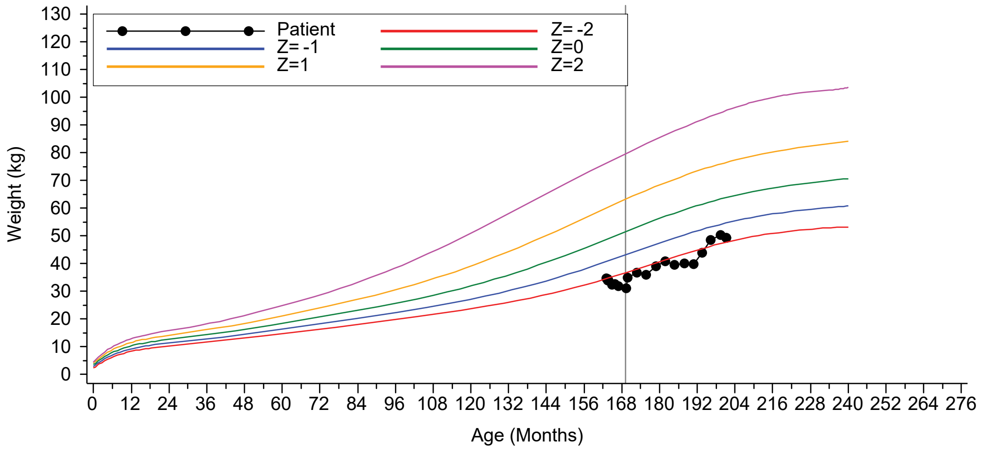

Patient 74

Seizure History: Partial Onset Seizures

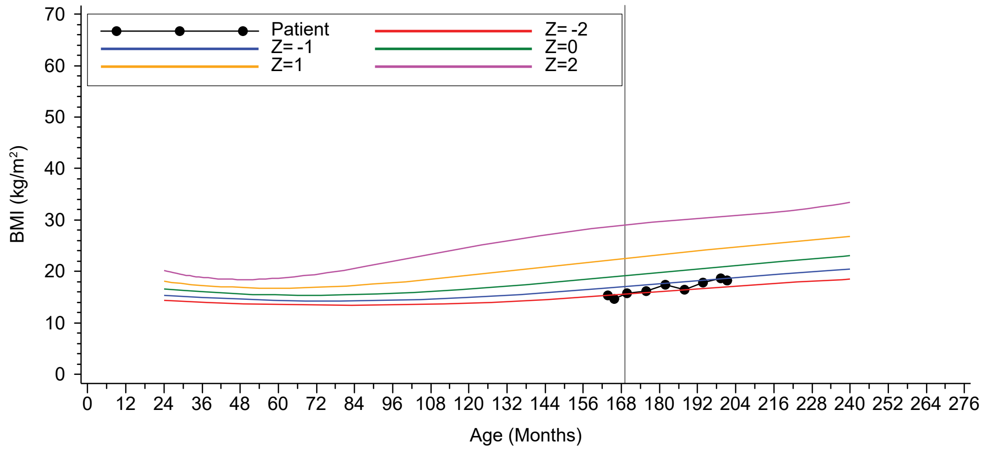

Patient 75

Seizure History: Partial Onset Seizures

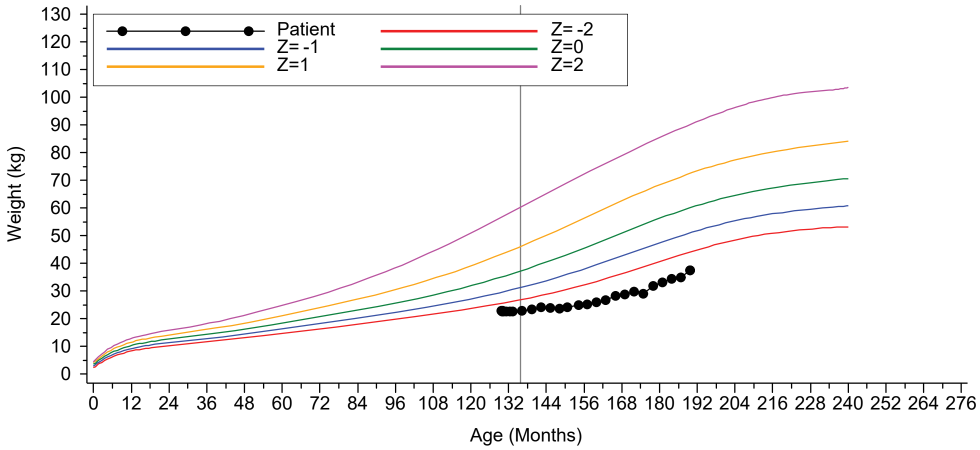

Patient 75

Seizure History: Partial Onset Seizures

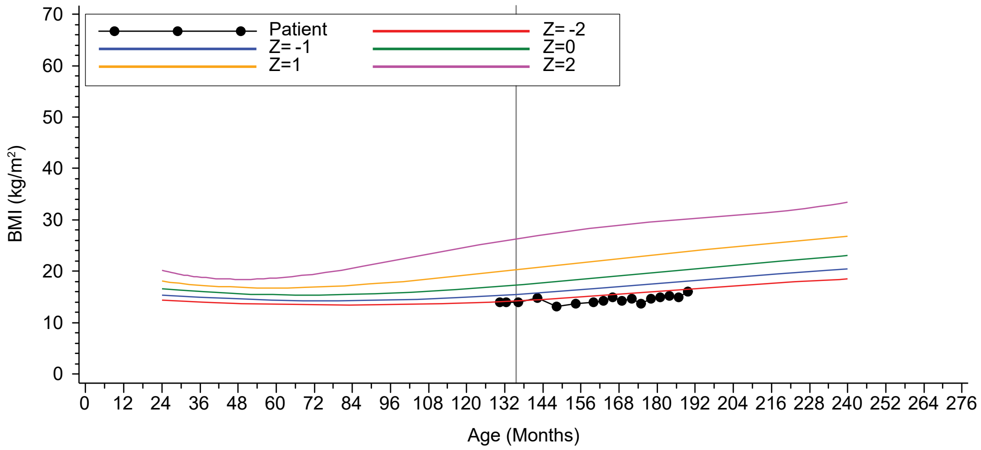

Patient 76

Seizure History: Partial Onset Seizures

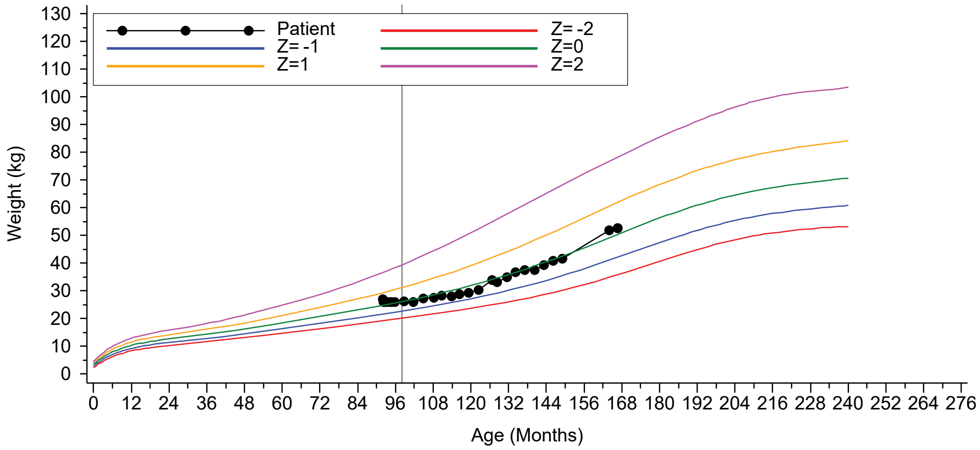

Patient 76

Seizure History: Partial Onset Seizures

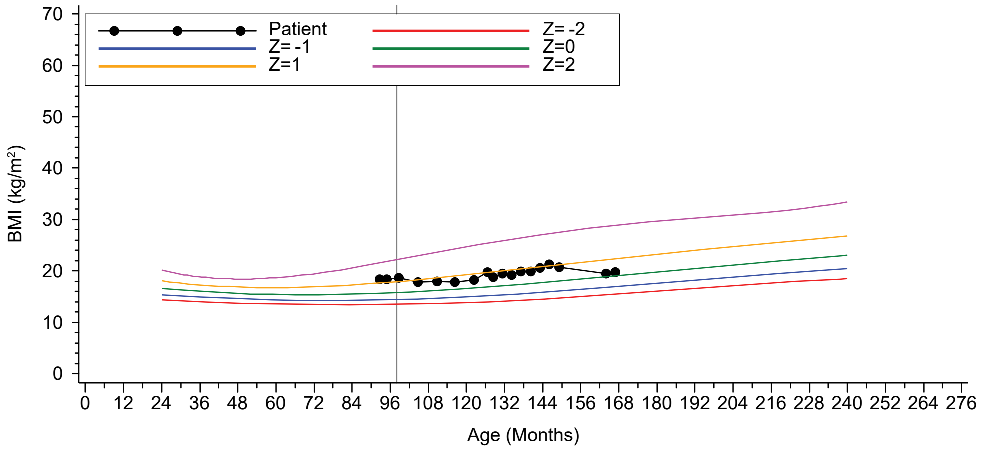

Patient 77

Seizure History: Partial Onset Seizures

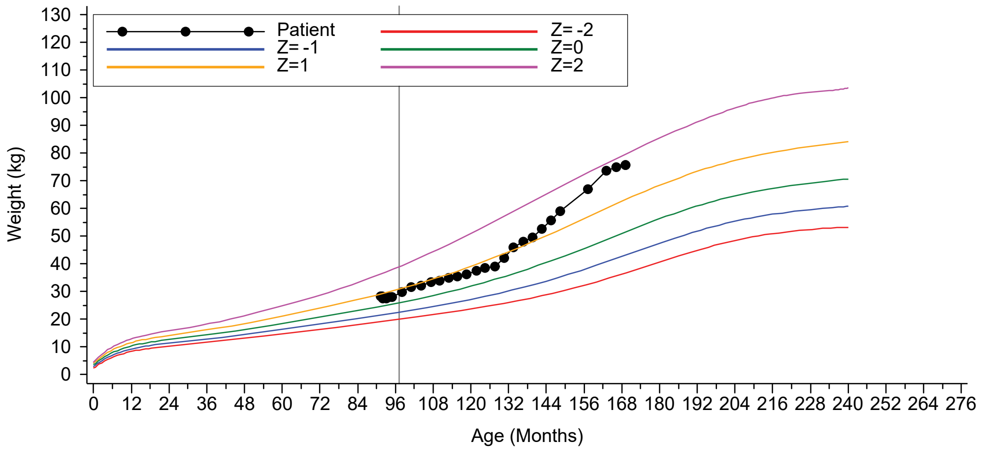

Patient 77

Seizure History: Partial Onset Seizures

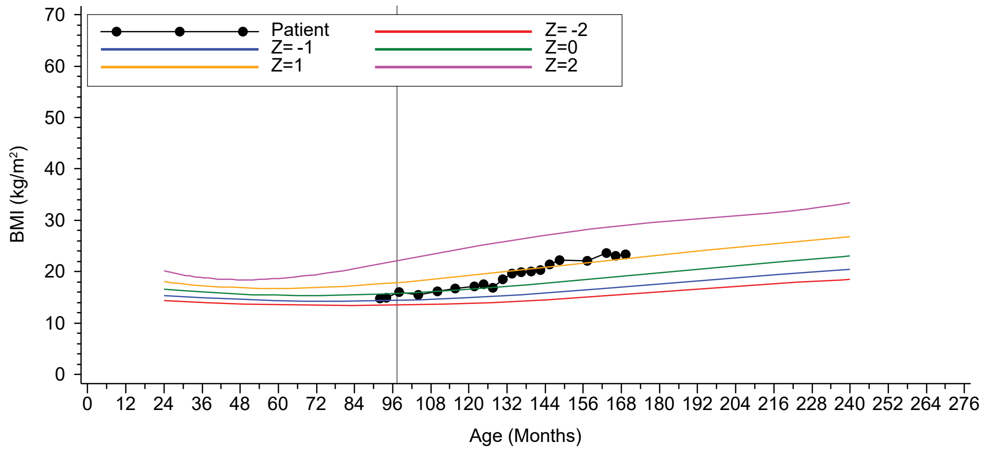

Patient 78

Seizure History: Partial Onset Seizures

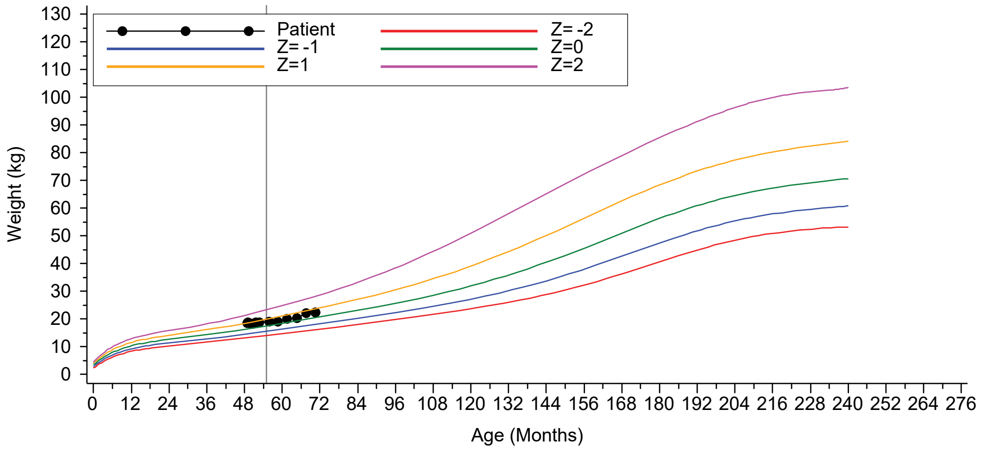

Patient 78

Seizure History: Partial Onset Seizures

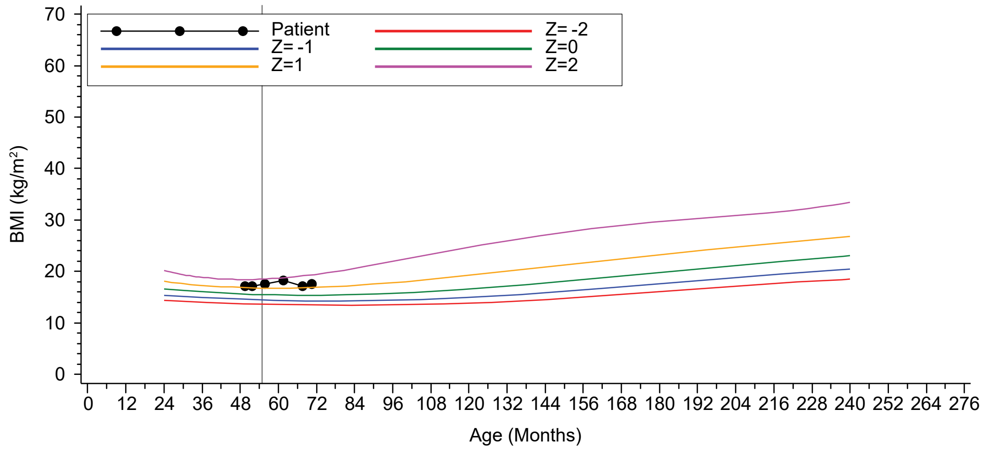

Patient 79
Seizure History: Partial Onset Seizures

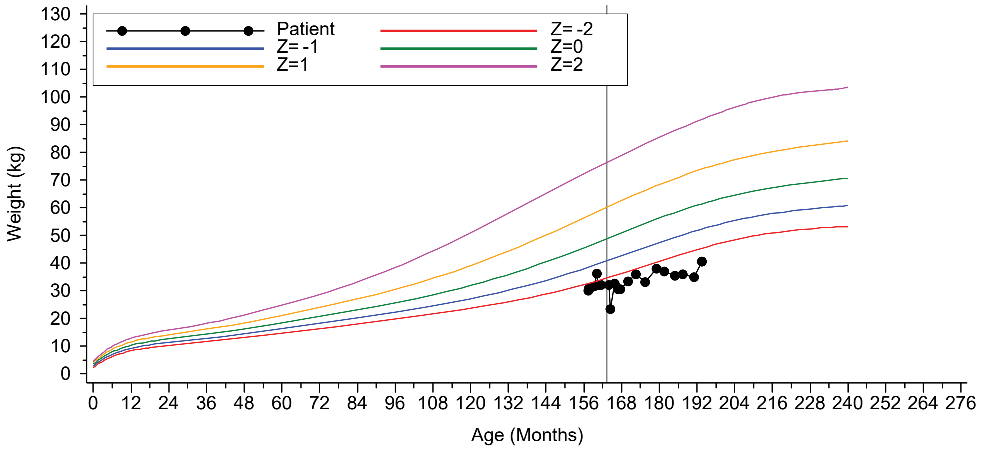

Patient 79
Seizure History: Partial Onset Seizures

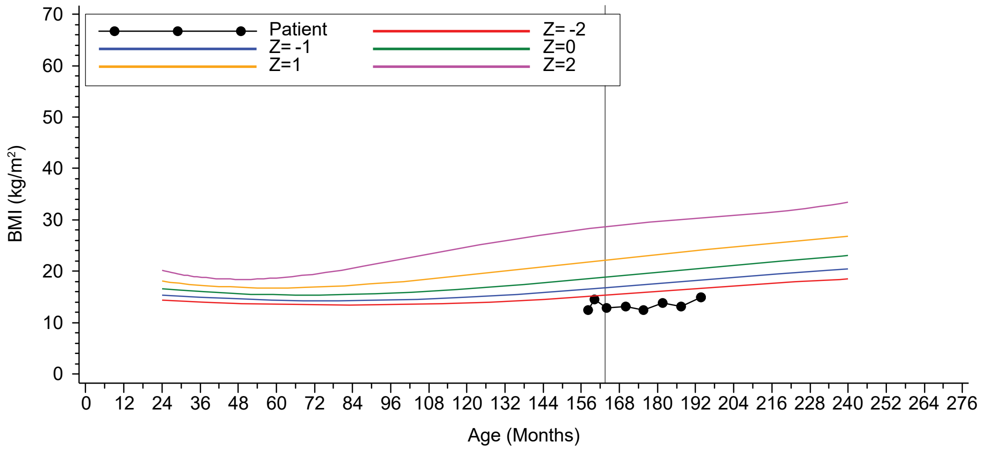

Patient 80
Seizure History: Partial Onset Seizures

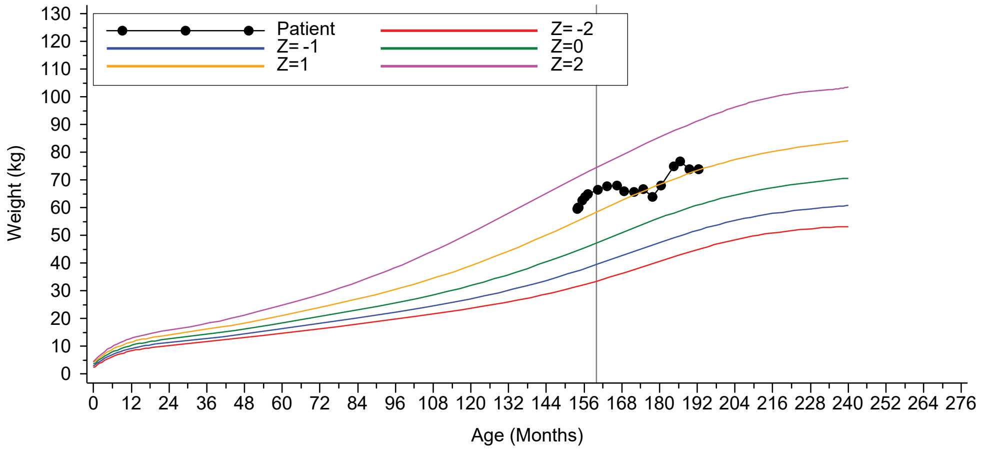

Patient 80
Seizure History: Partial Onset Seizures

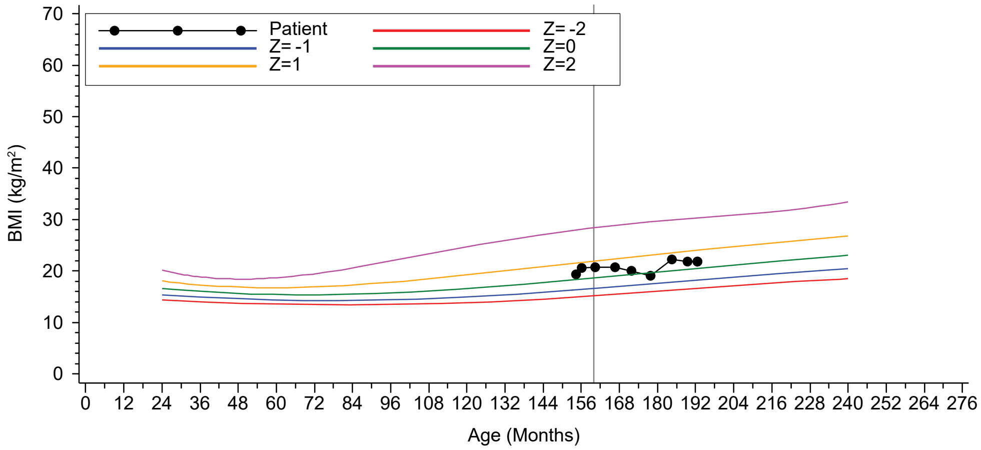

Patient 81

Seizure History: Partial Onset Seizures

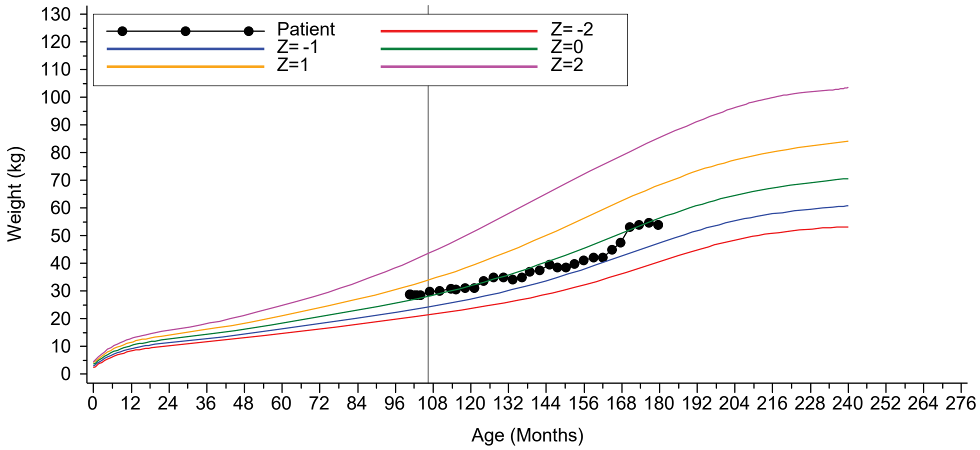

Patient 81

Seizure History: Partial Onset Seizures

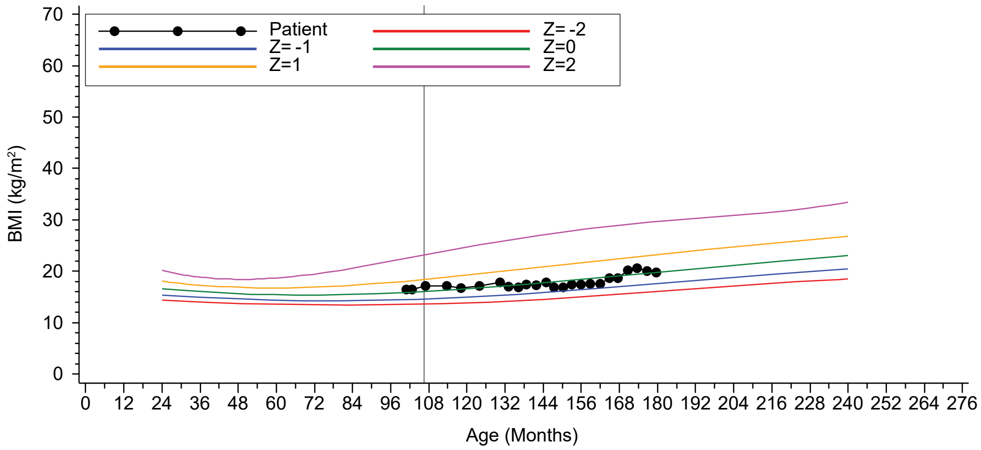

Patient 82

Seizure History: Partial Onset Seizures

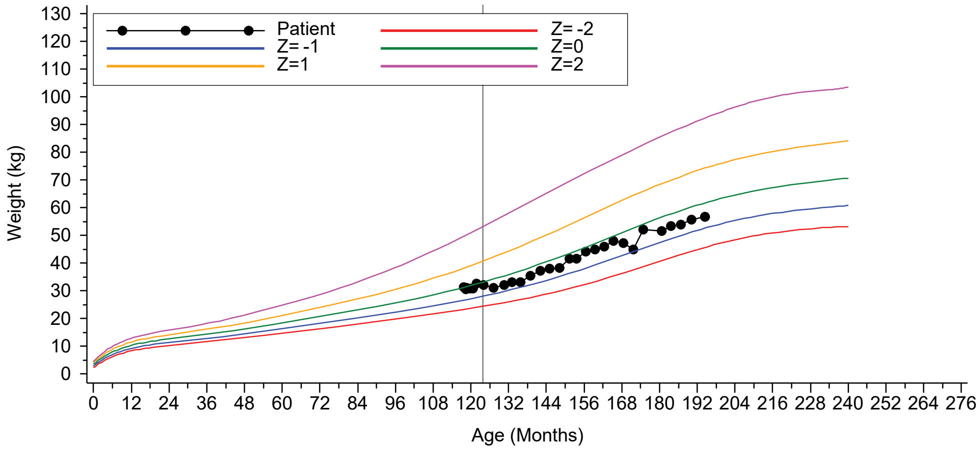

Patient 82

Seizure History: Partial Onset Seizures

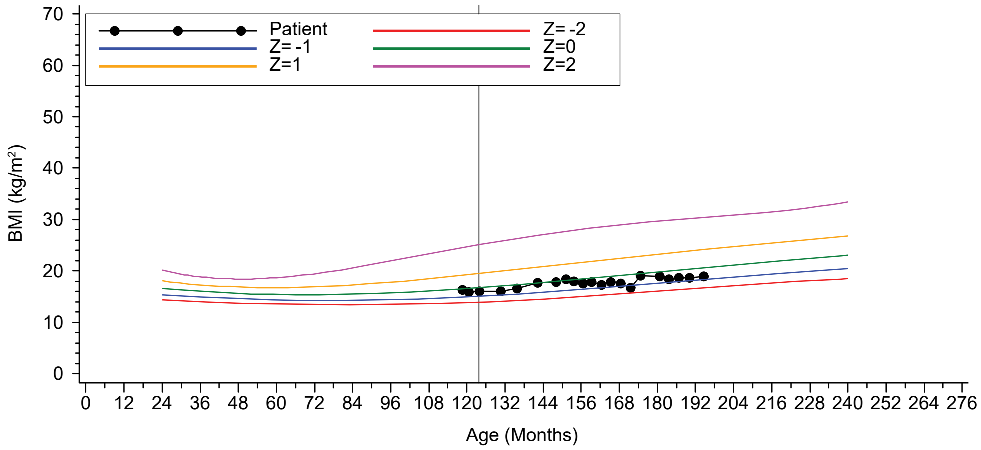

Patient 83

Seizure History: Partial Onset Seizures

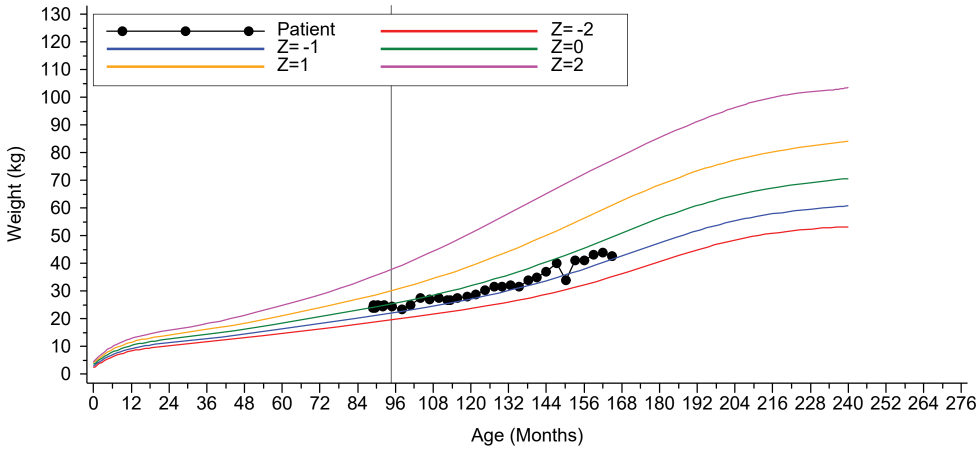

Patient 83

Seizure History: Partial Onset Seizures

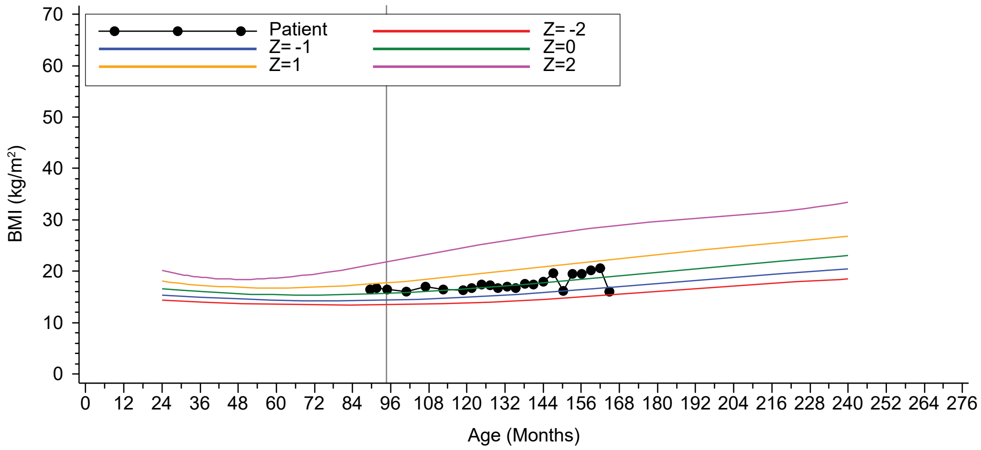

Patient 84

Seizure History: Partial Onset Seizures

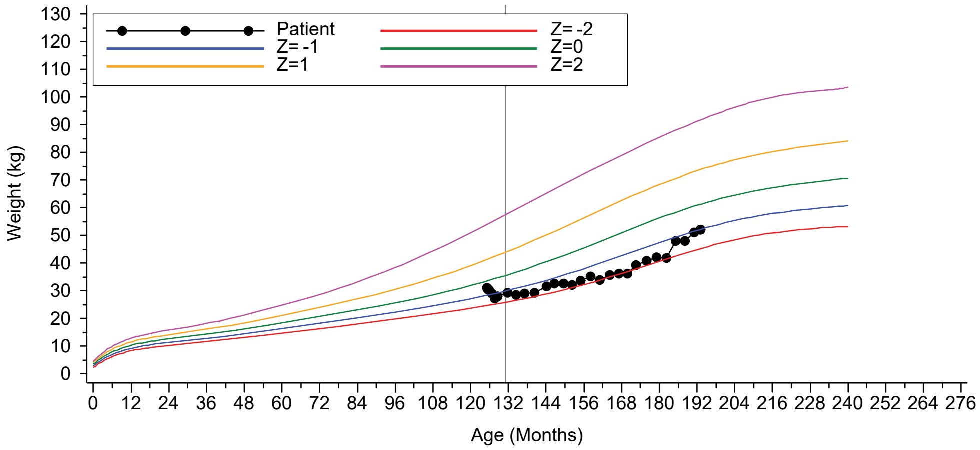

Patient 84

Seizure History: Partial Onset Seizures

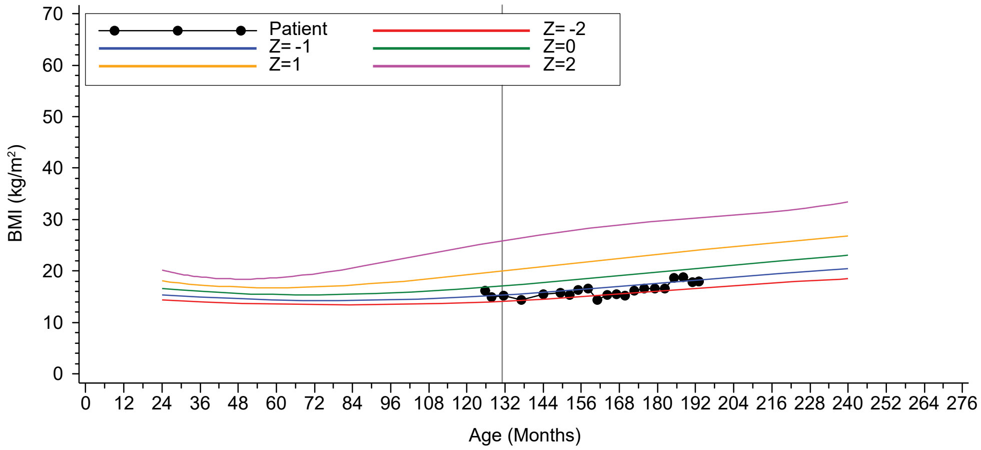

Patient 85

Seizure History: Partial Onset Seizures

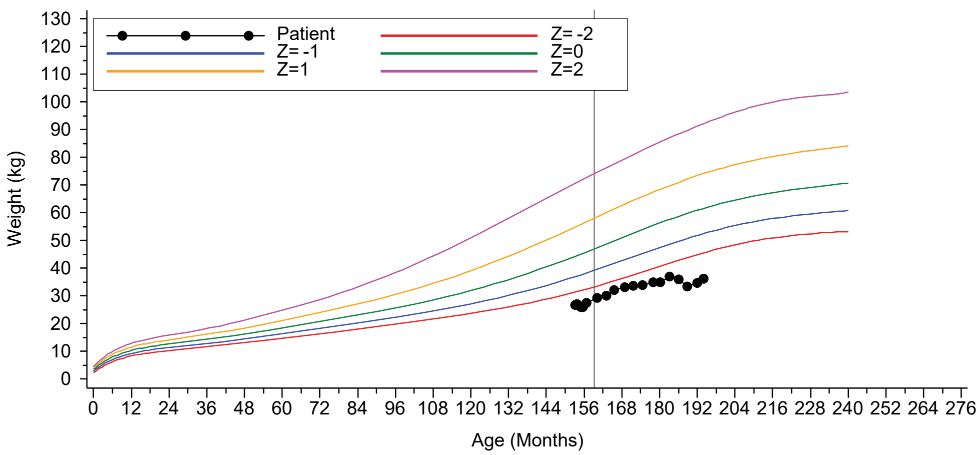

Patient 85

Seizure History: Partial Onset Seizures

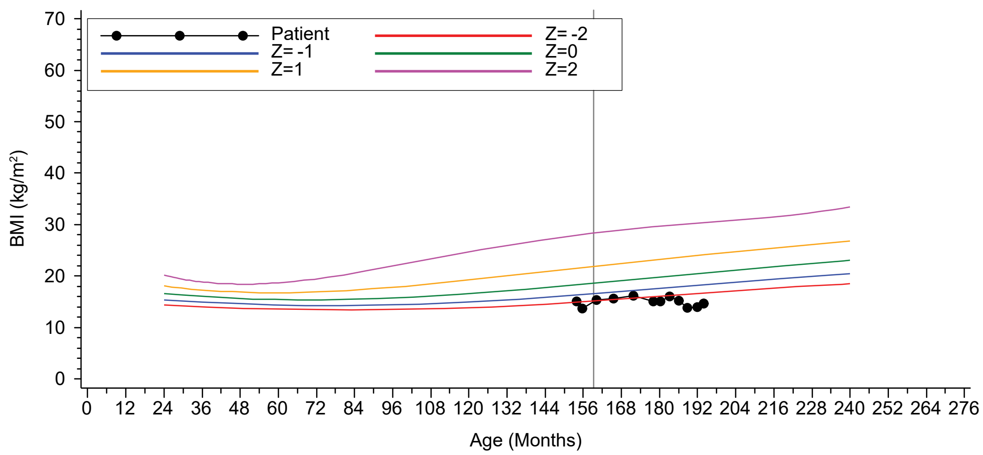

Patient 86

Seizure History: Partial Onset Seizures

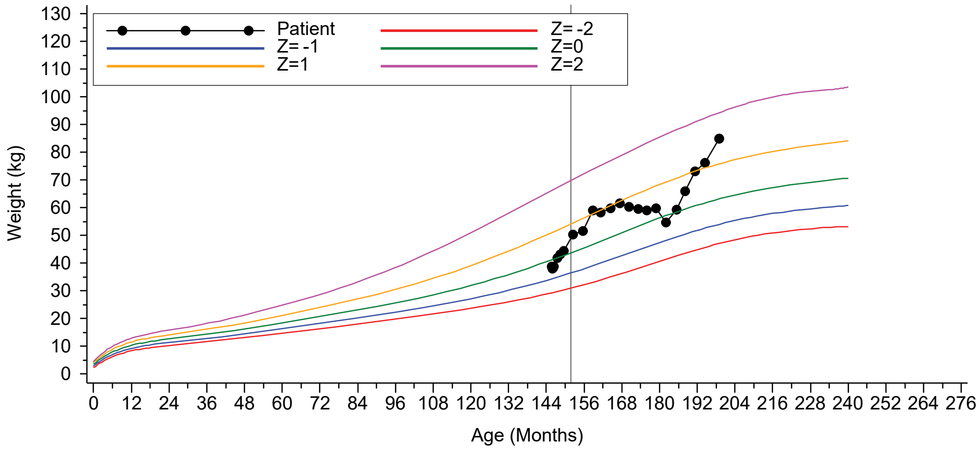

Patient 86

Seizure History: Partial Onset Seizures

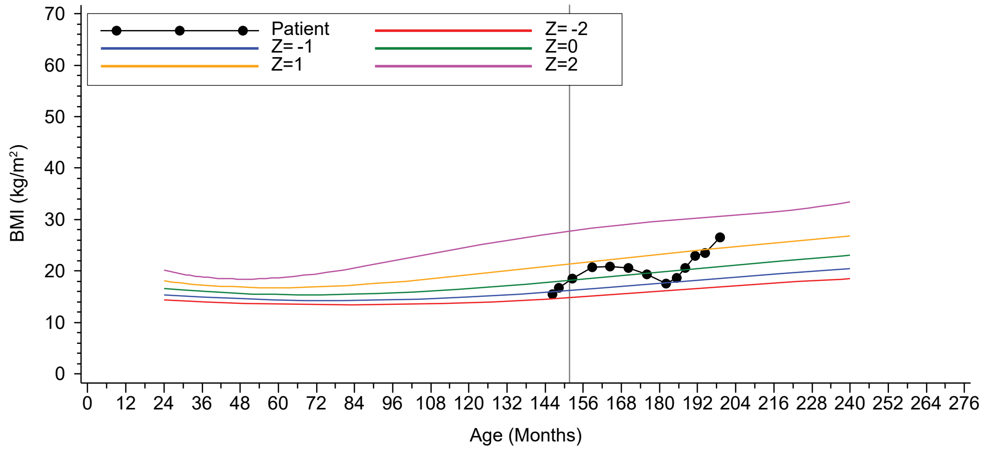

Patient 87

Seizure History: Partial Onset Seizures

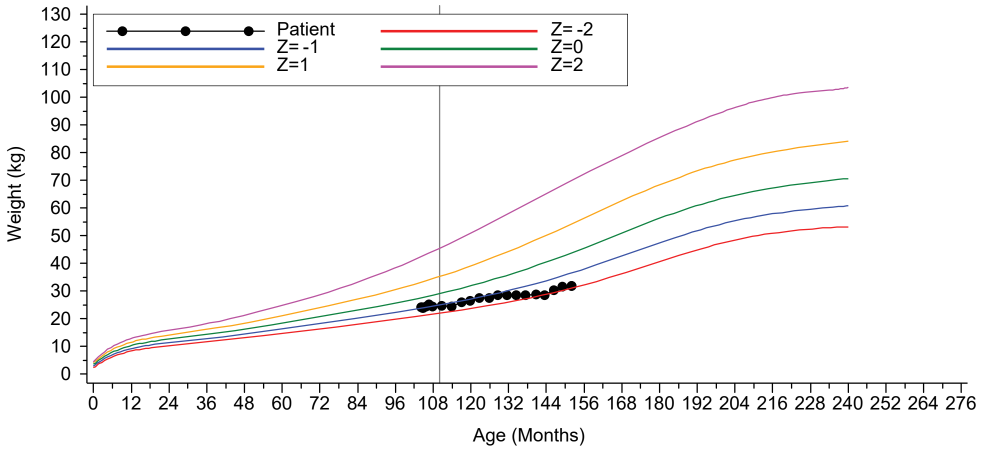

Patient 87

Seizure History: Partial Onset Seizures

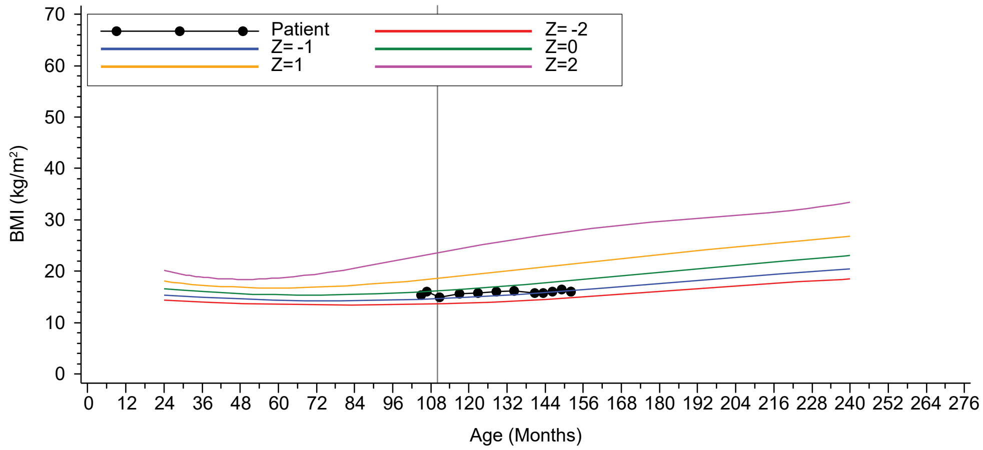

Patient 88

Seizure History: Partial Onset Seizures

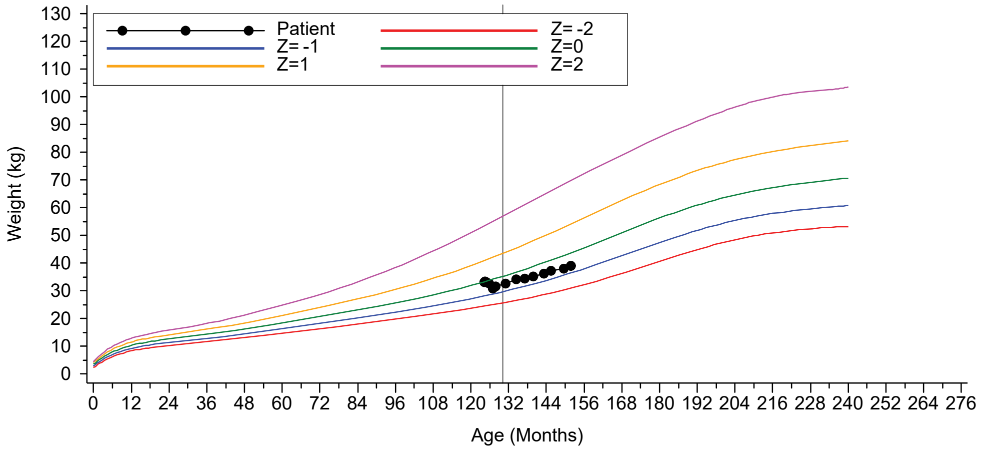

Patient 88

Seizure History: Partial Onset Seizures

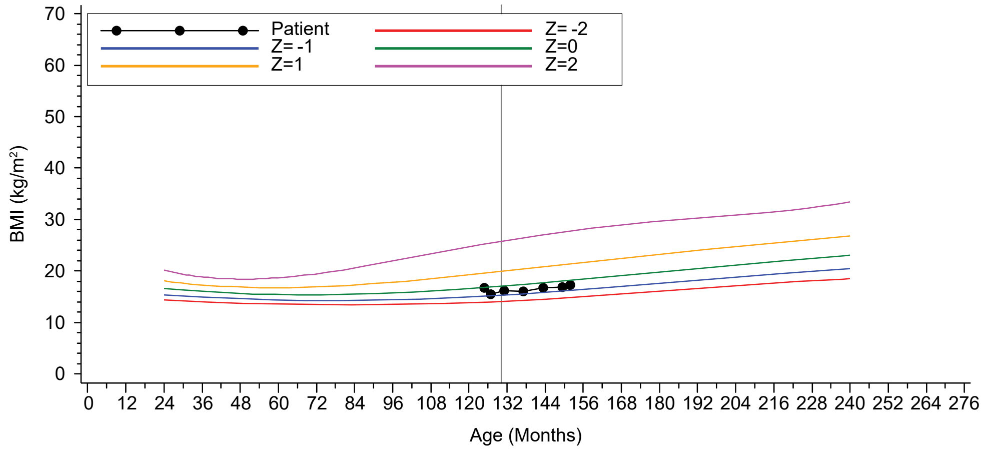

Patient 89

Seizure History: Partial Onset Seizures

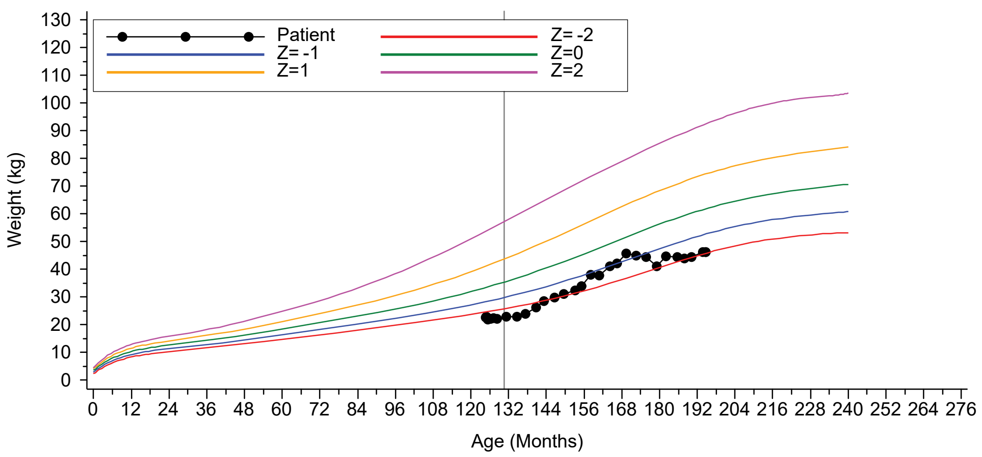

Patient 89

Seizure History: Partial Onset Seizures

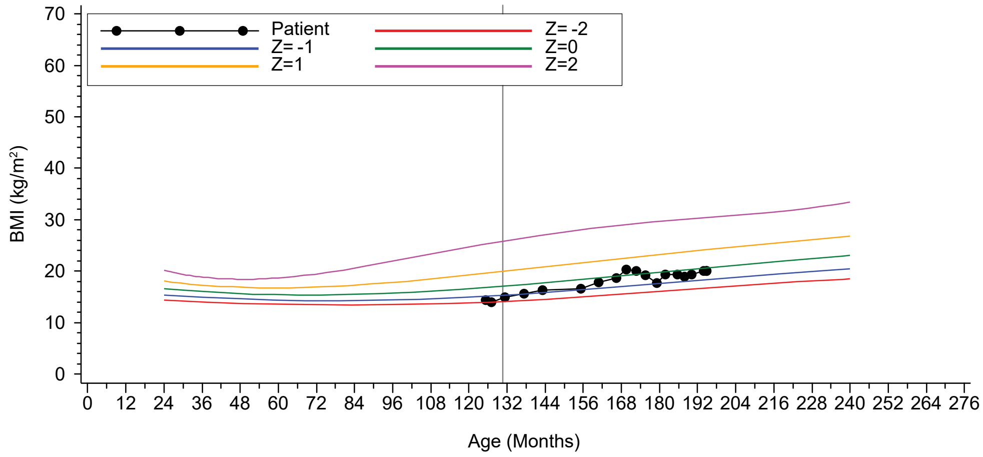

Patient 90

Seizure History: Partial Onset Seizures

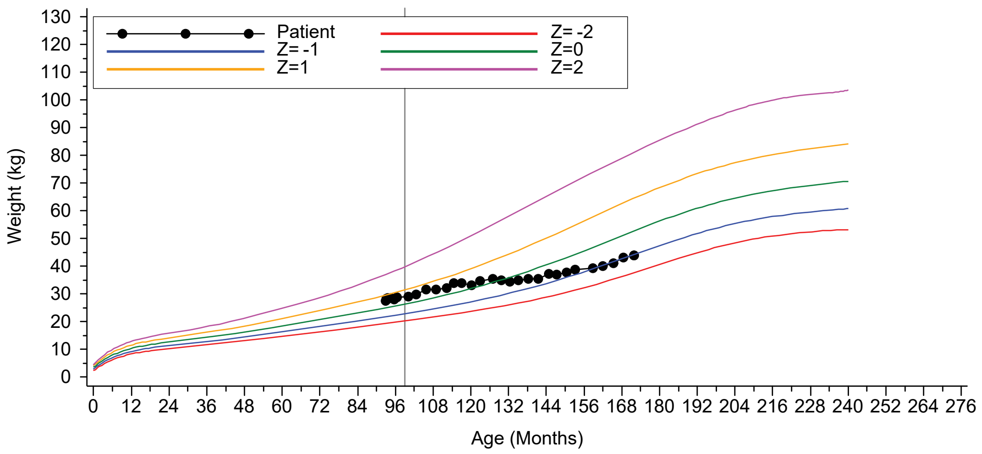

Patient 90

Seizure History: Partial Onset Seizures

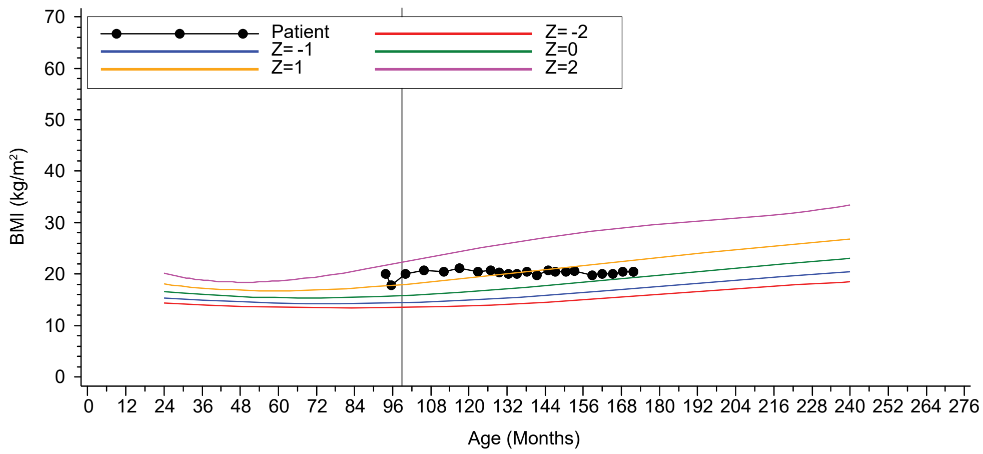

Patient 91

Seizure History: Primary Generalized Seizures and Unknown

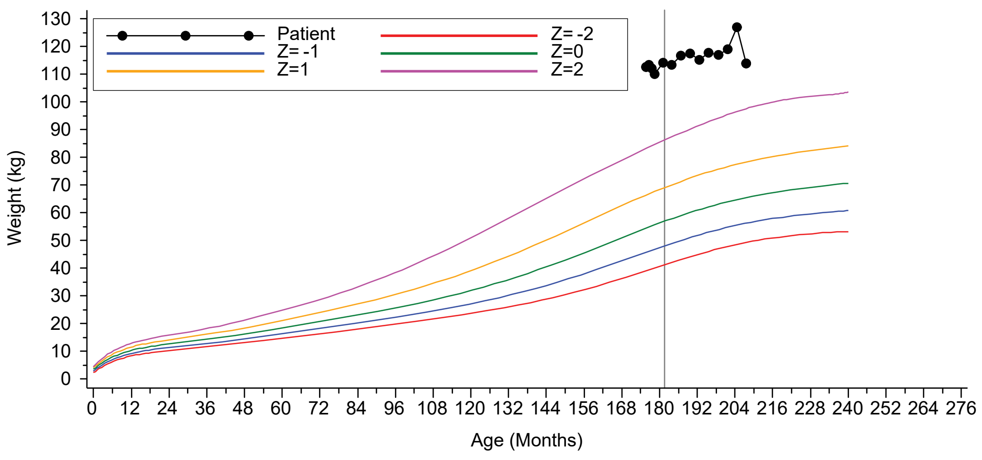

Patient 91

Seizure History: Primary Generalized Seizures and Unknown

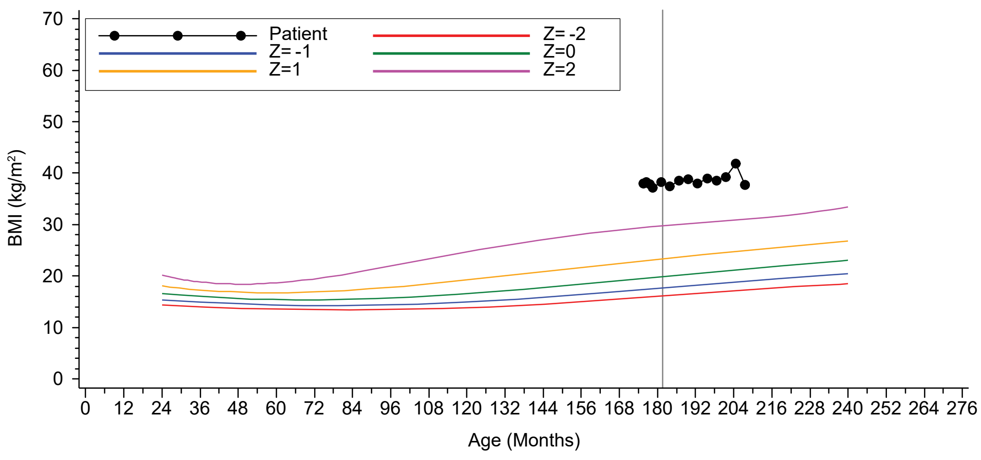

Patient 92

Seizure History: Primary Generalized Seizures and Unknown

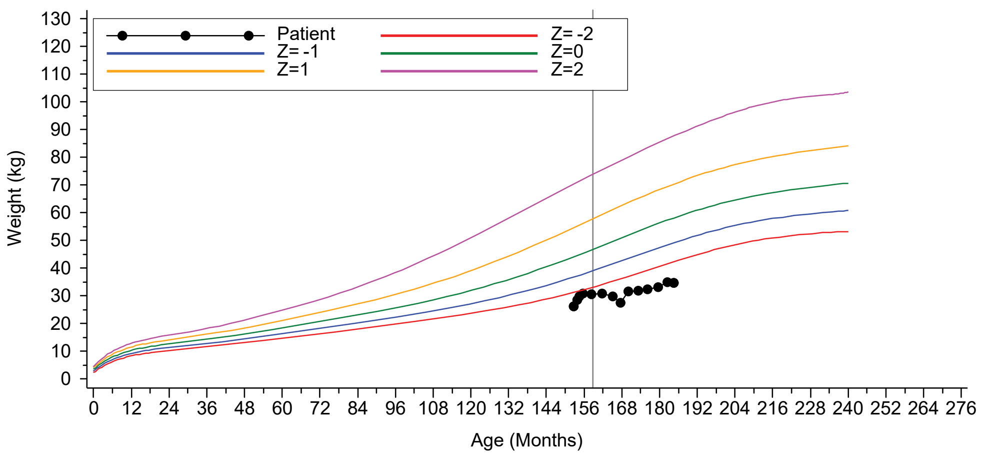

Patient 92

Seizure History: Primary Generalized Seizures and Unknown

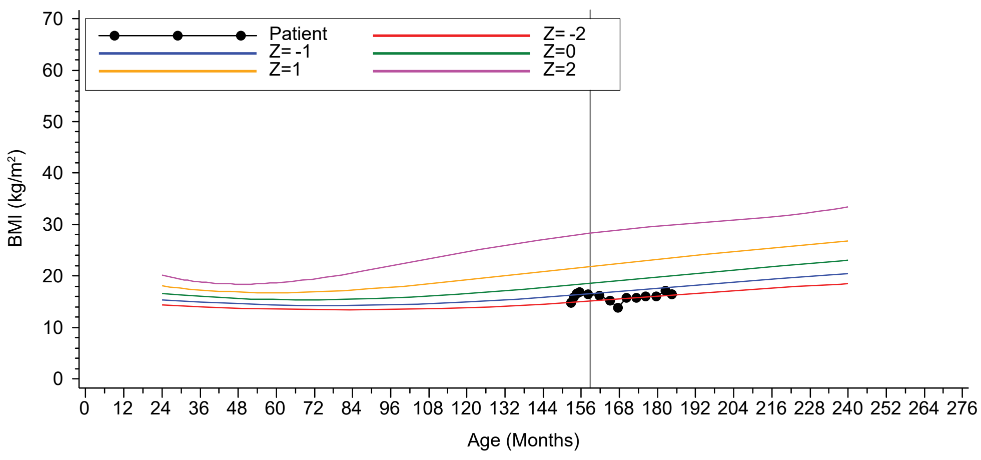

Patient 93

Seizure History: Primary Generalized Seizures and Unknown

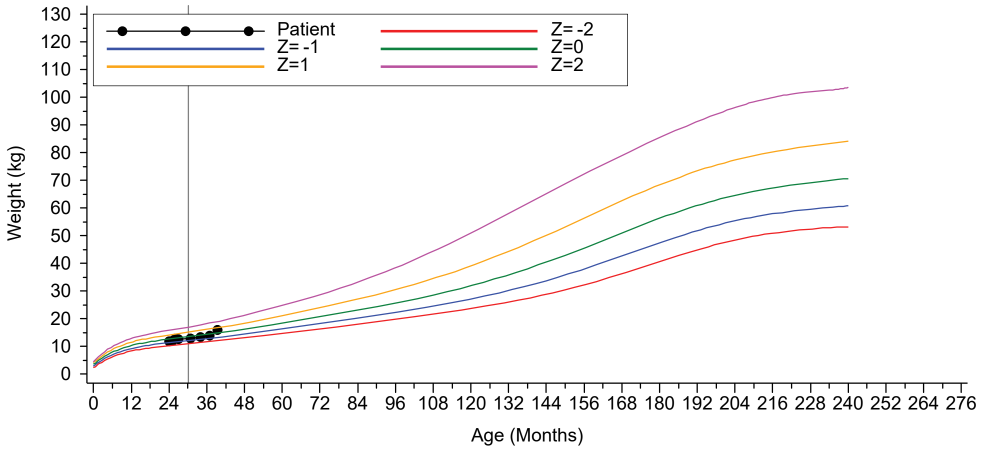

Patient 93

Seizure History: Primary Generalized Seizures and Unknown

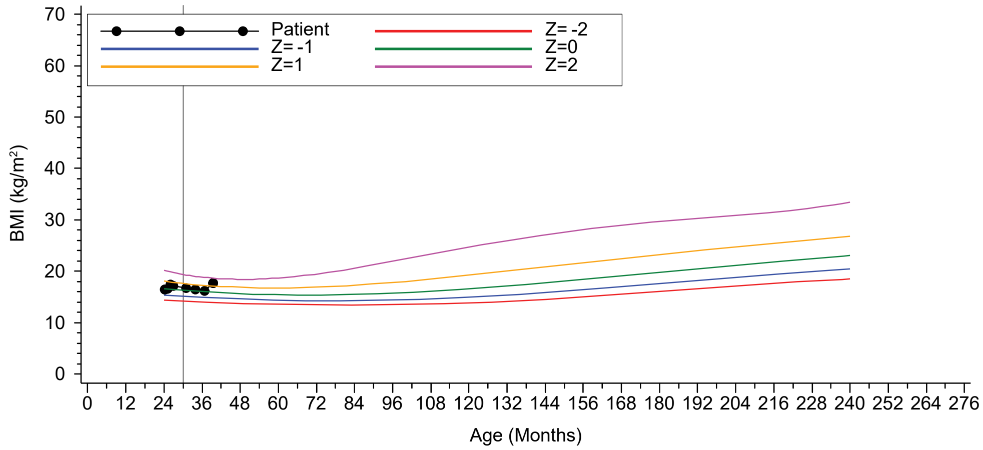

Patient 94

Seizure History: Primary Generalized Seizures and Unknown

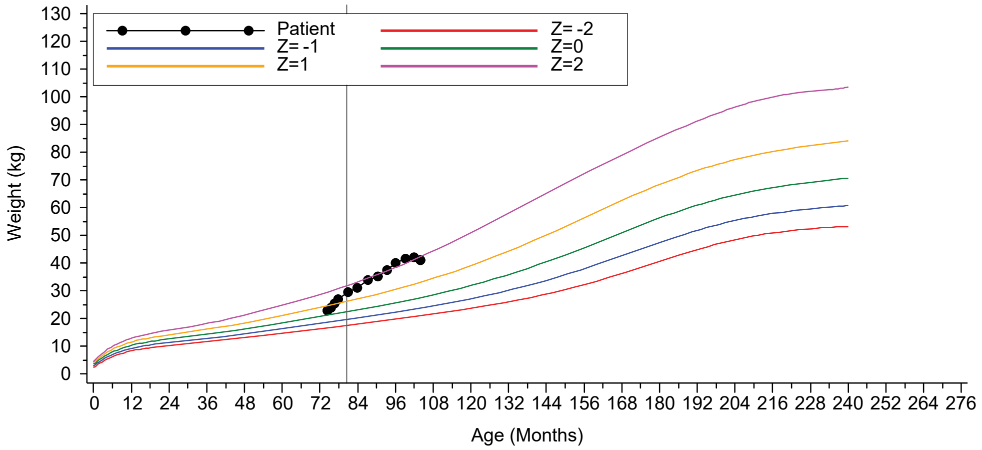

Patient 94

Seizure History: Primary Generalized Seizures and Unknown

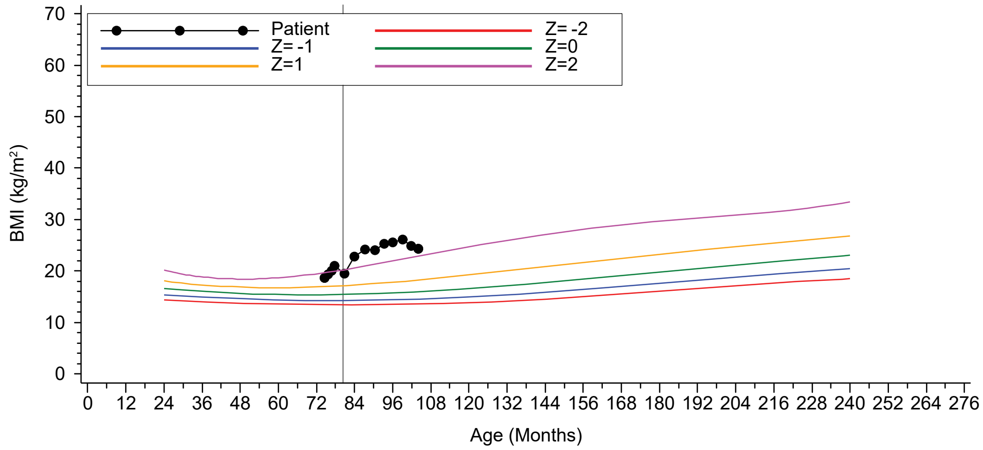

Patient 95

Seizure History: Primary Generalized Seizures and Unknown

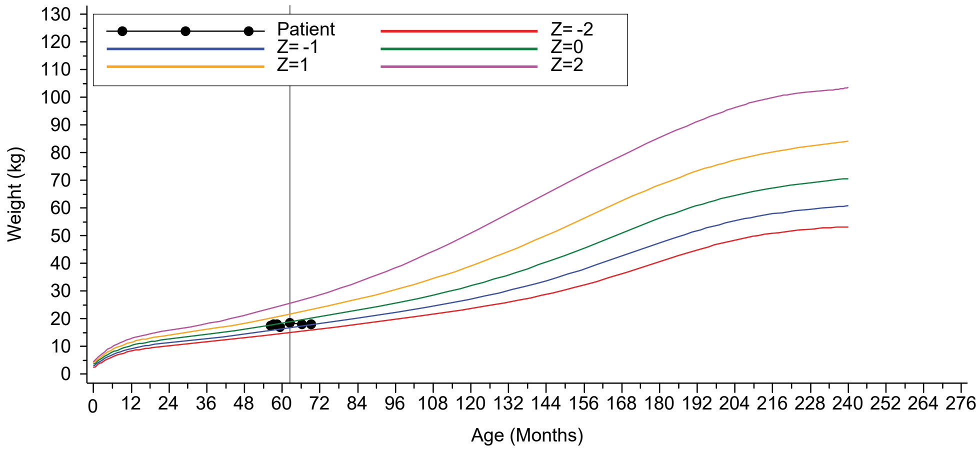

Patient 95

Seizure History: Primary Generalized Seizures and Unknown

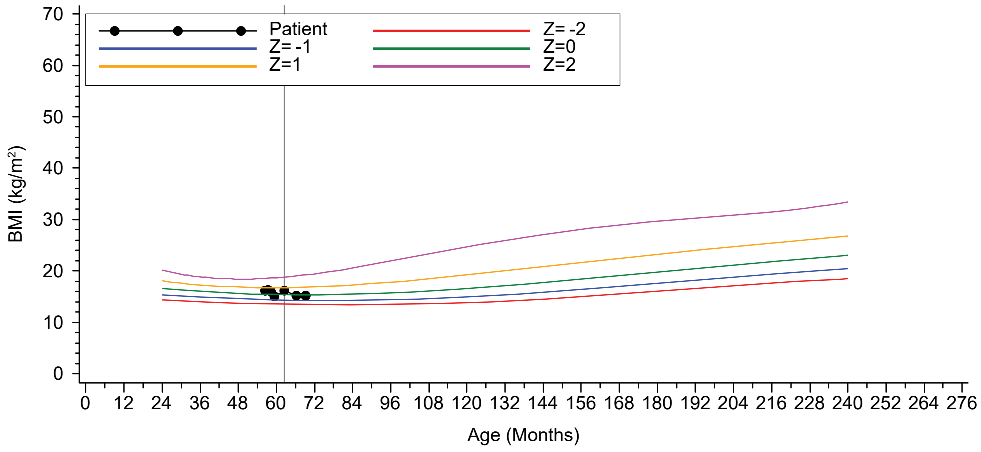

Patient 96

Seizure History: Primary Generalized Seizures and Unknown

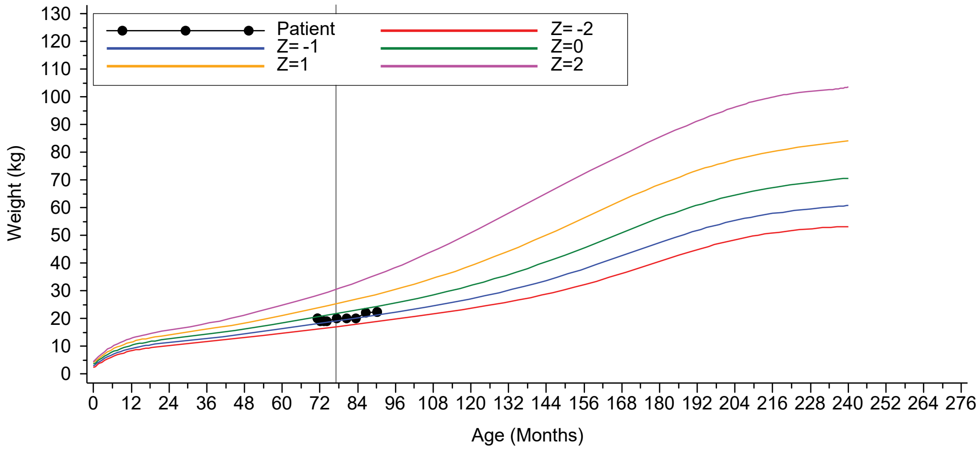

Patient 96

Seizure History: Primary Generalized Seizures and Unknown

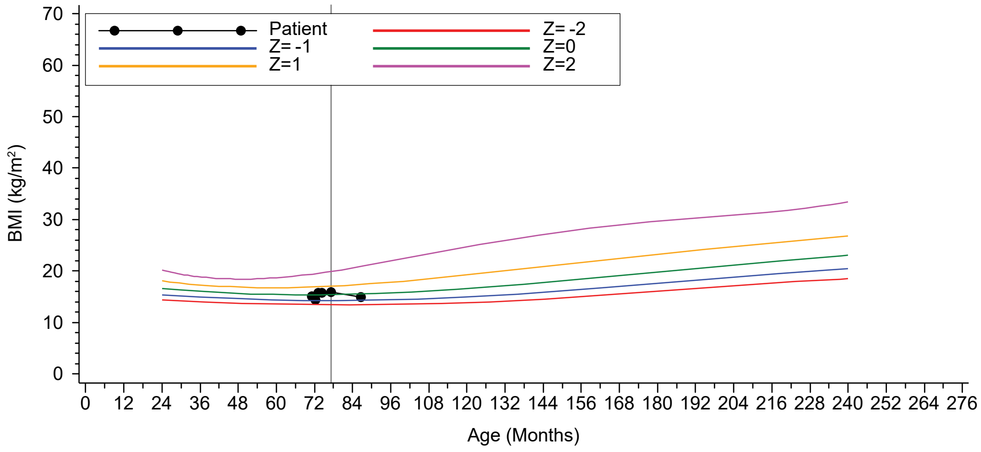

Patient 97

Seizure History: Primary Generalized Seizures and Unknown

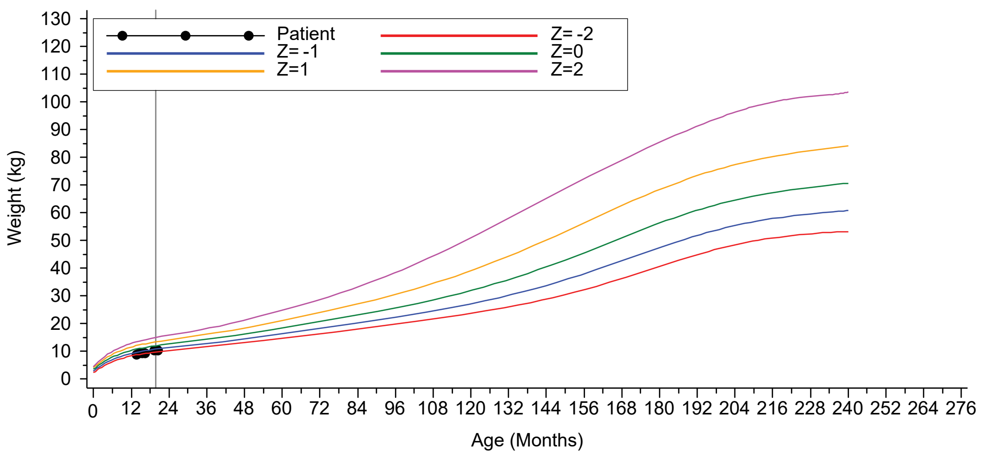

Patient 97

Seizure History: Primary Generalized Seizures and Unknown

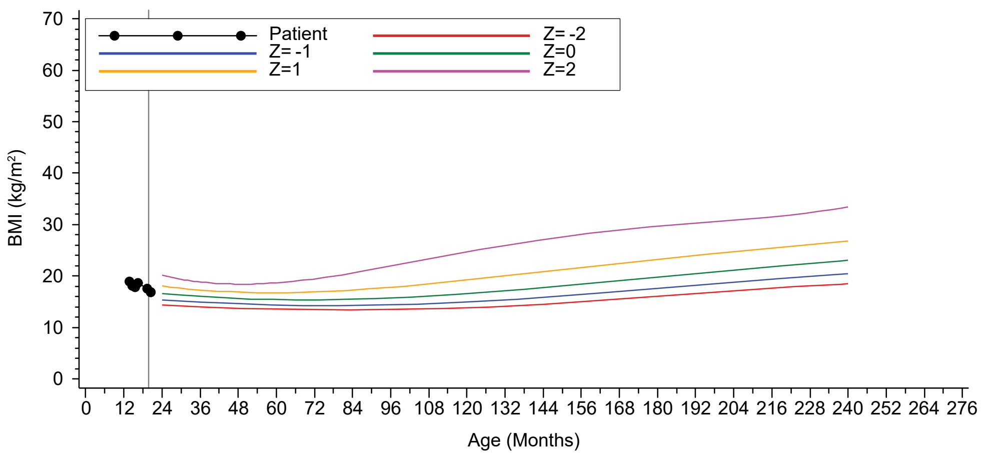

Patient 98

Seizure History: Primary Generalized Seizures and Unknown

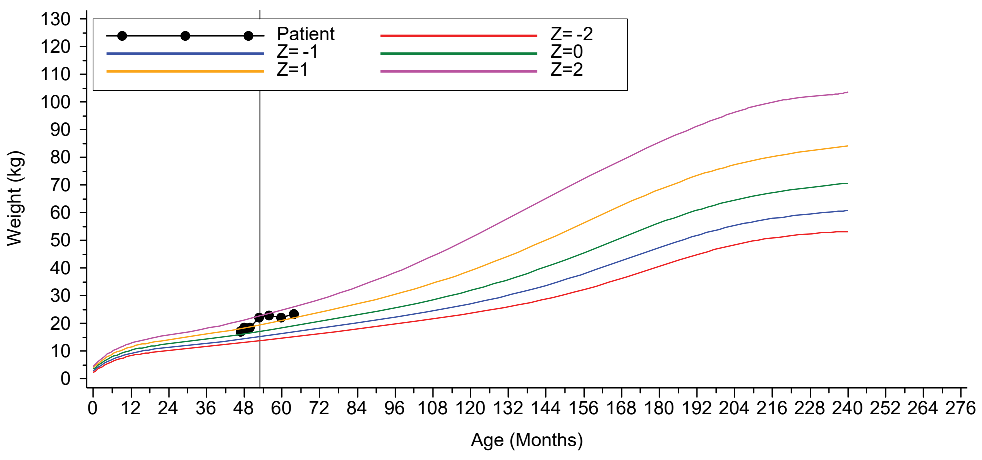

Patient 98

Seizure History: Primary Generalized Seizures and Unknown

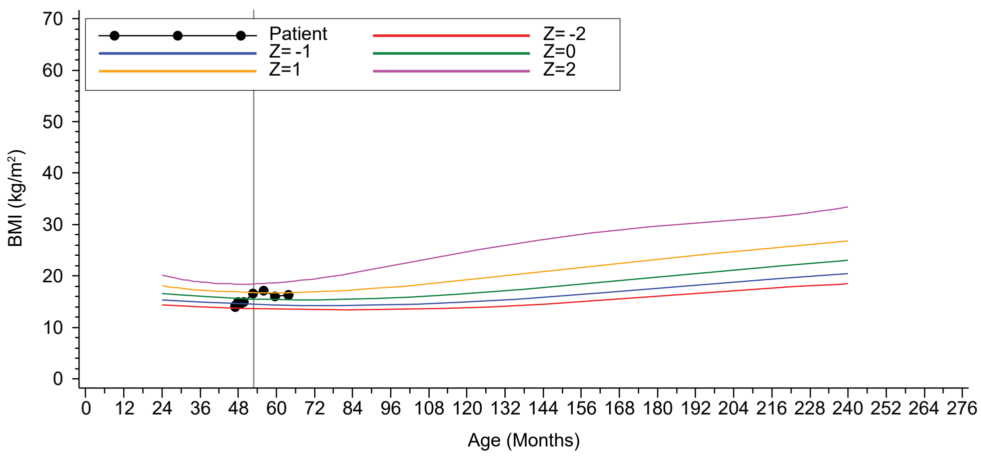

Patient 99

Seizure History: Primary Generalized Seizures and Unknown

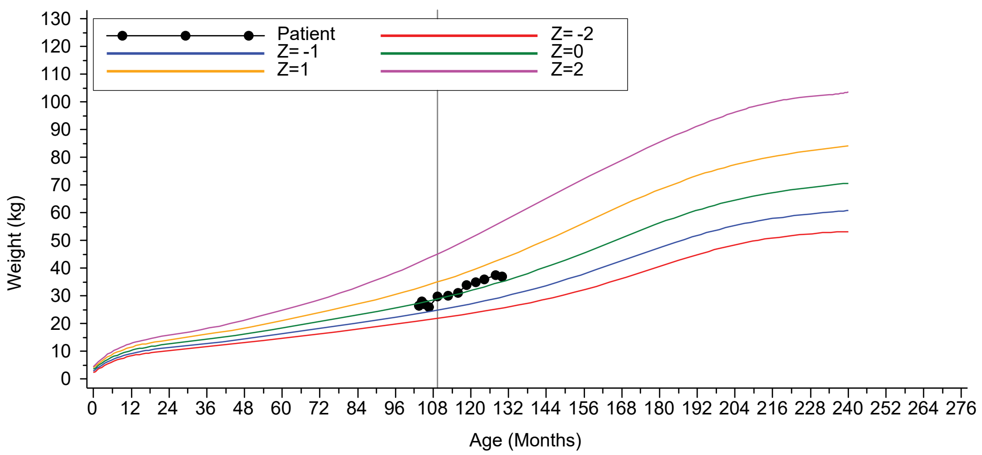

Patient 99

Seizure History: Primary Generalized Seizures and Unknown

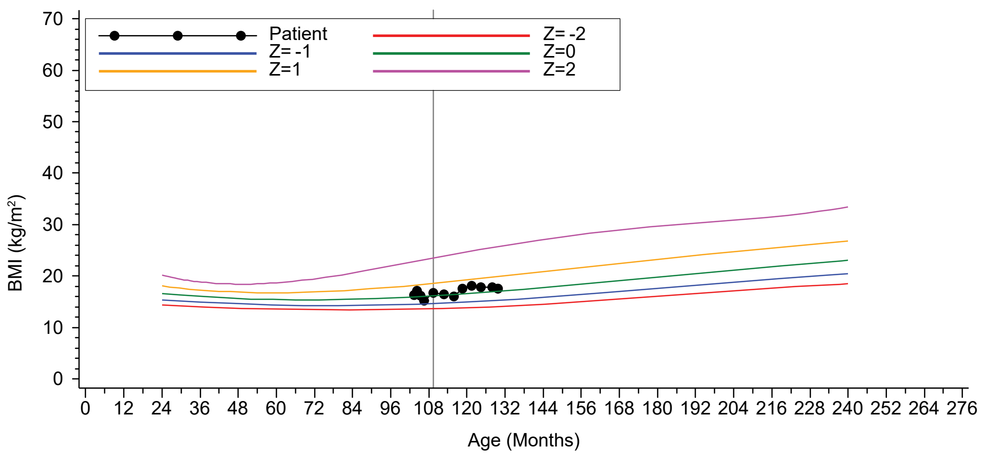

Patient 100

Seizure History: Primary Generalized Seizures and Unknown

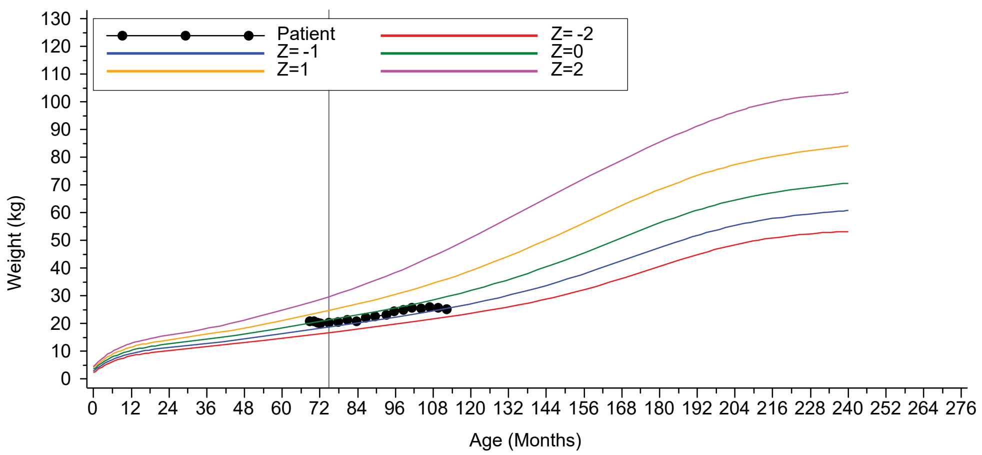

Patient 100

Seizure History: Primary Generalized Seizures and Unknown

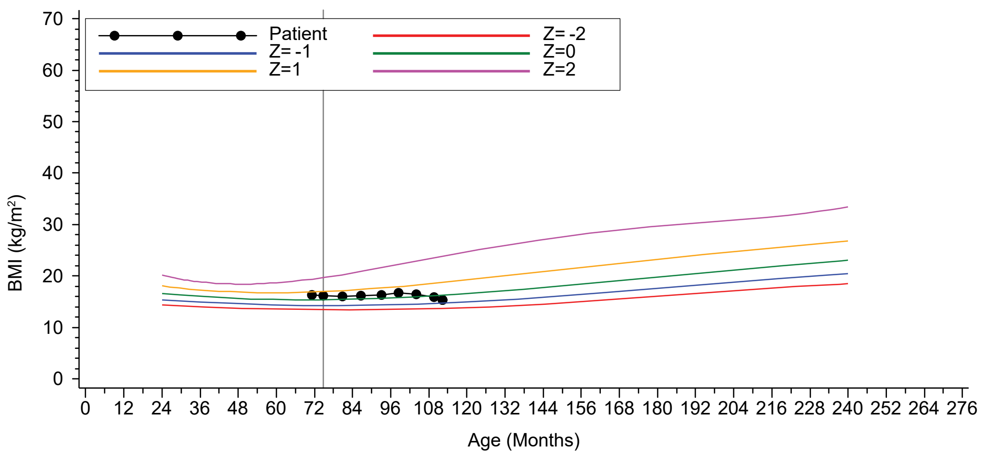

Patient 101

Seizure History: Primary Generalized Seizures and Unknown

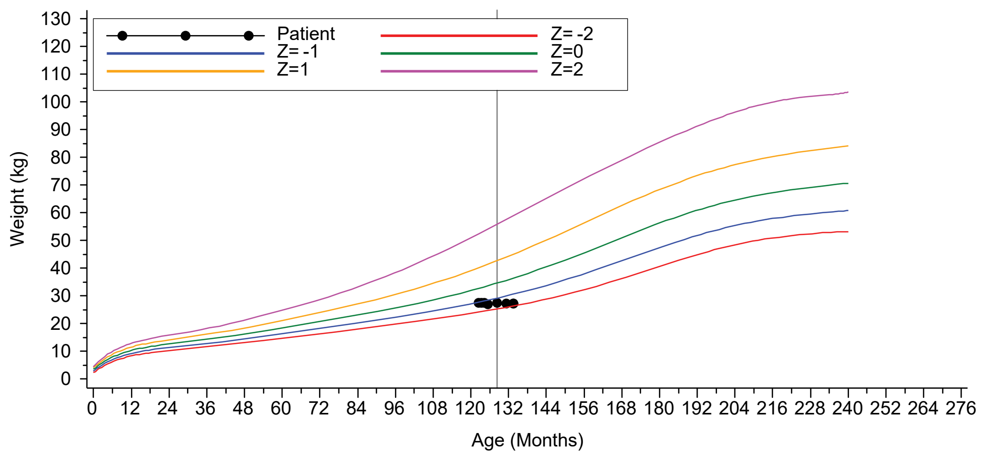

Patient 101

Seizure History: Primary Generalized Seizures and Unknown

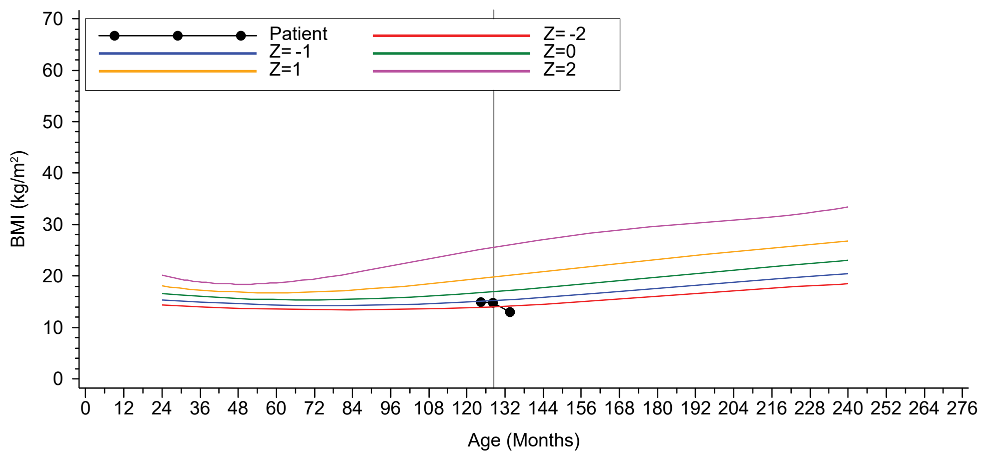

Patient 102

Seizure History: Primary Generalized Seizures and Unknown

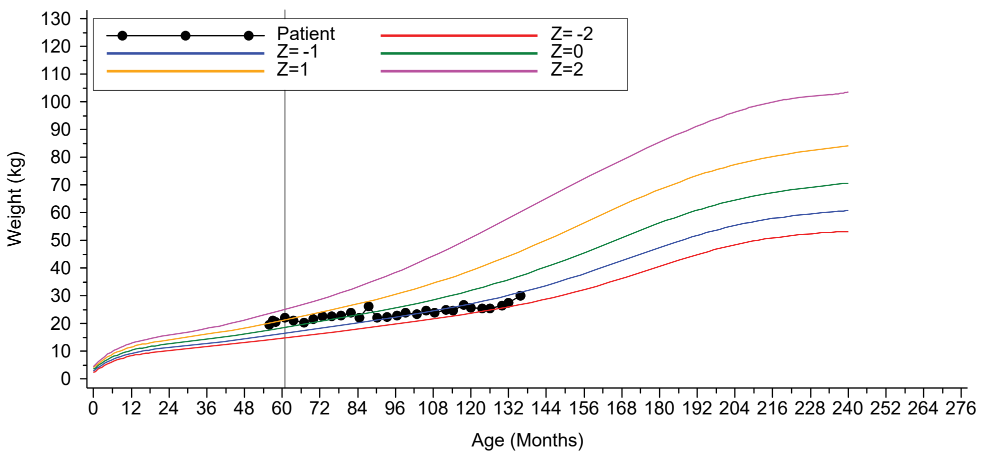

Patient 102

Seizure History: Primary Generalized Seizures and Unknown

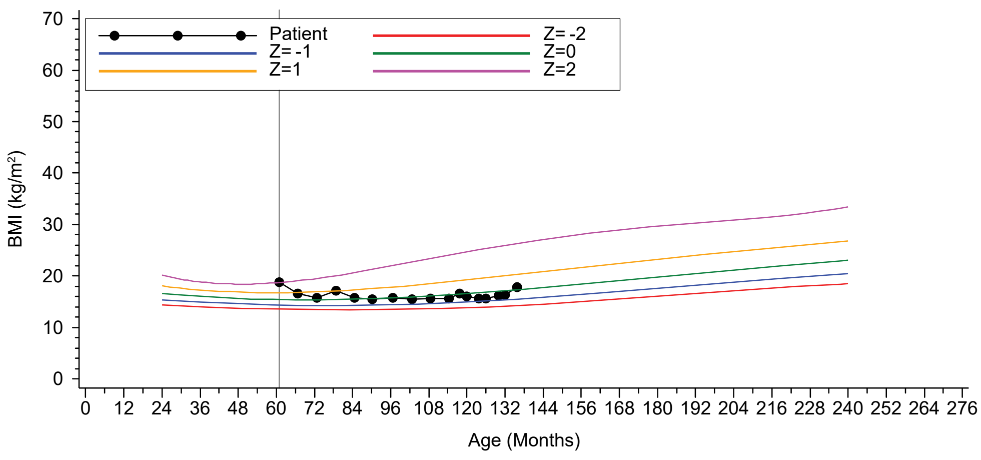

Patient 103

Seizure History: Primary Generalized Seizures and Unknown

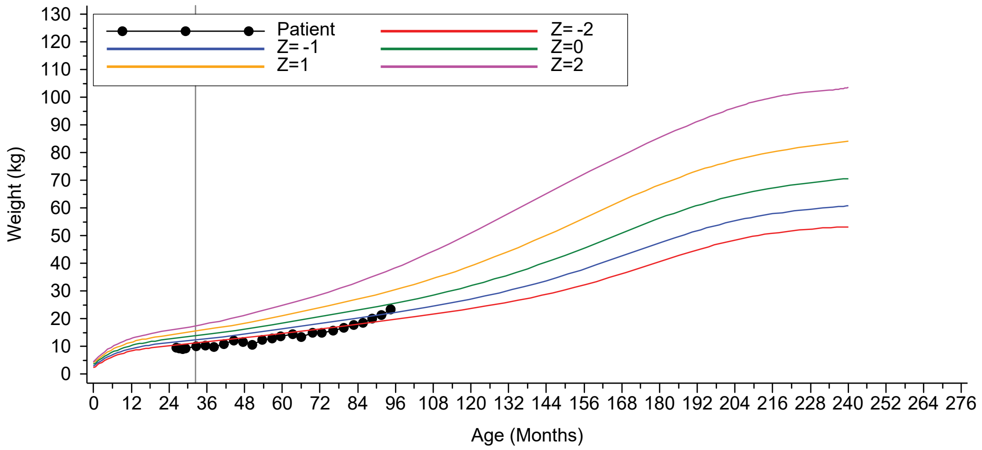

Patient 103

Seizure History: Primary Generalized Seizures and Unknown

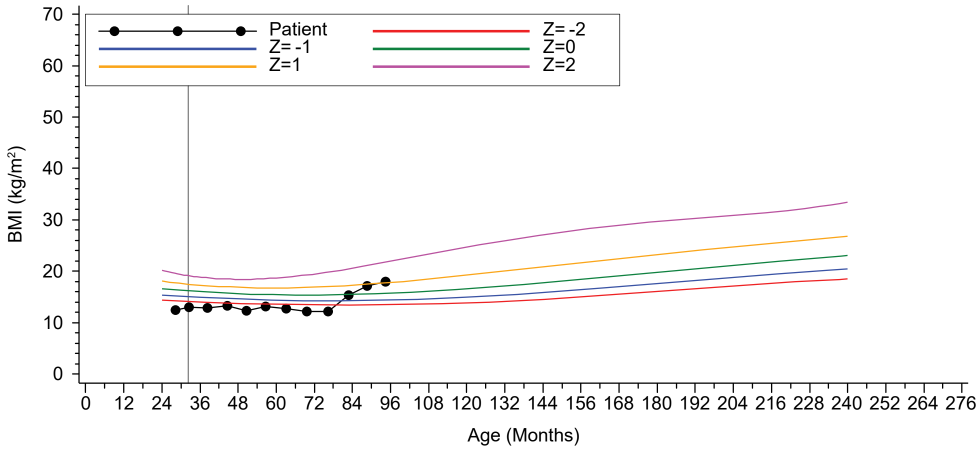

Patient 104

Seizure History: Primary Generalized Seizures and Unknown

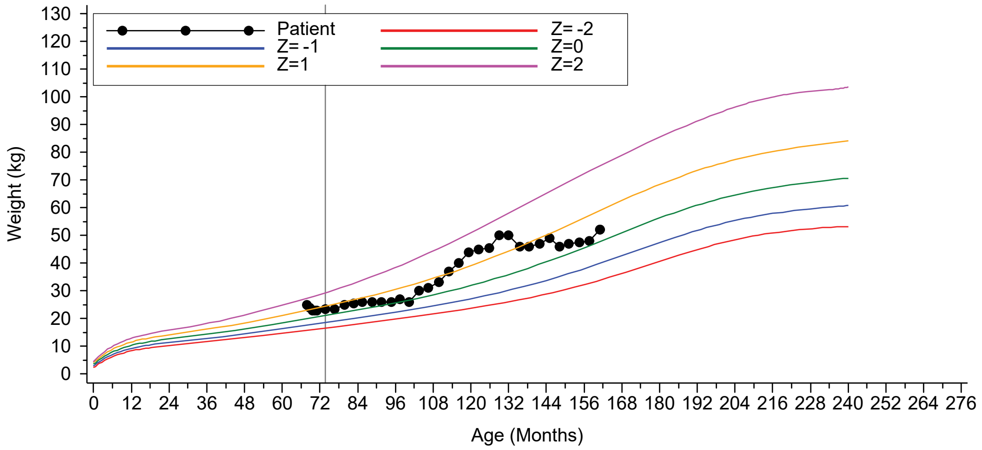

Patient 104

Seizure History: Primary Generalized Seizures and Unknown

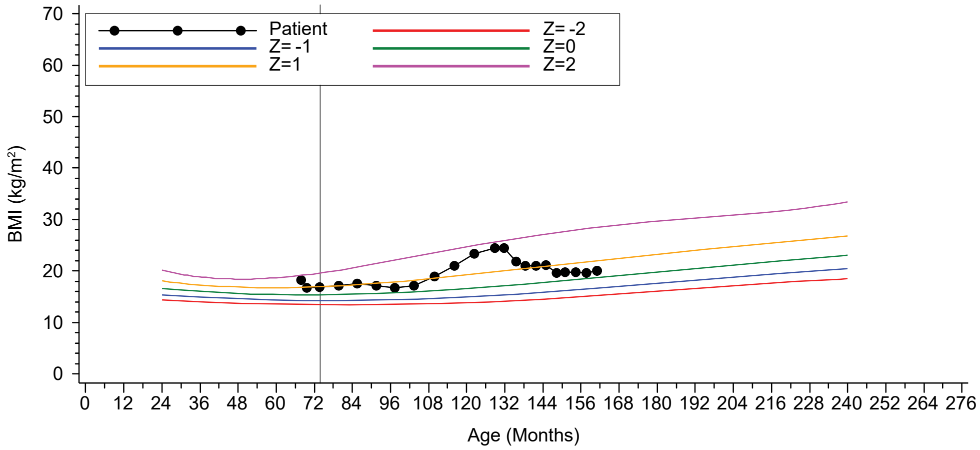

Patient 105

Seizure History: Primary Generalized Seizures and Unknown

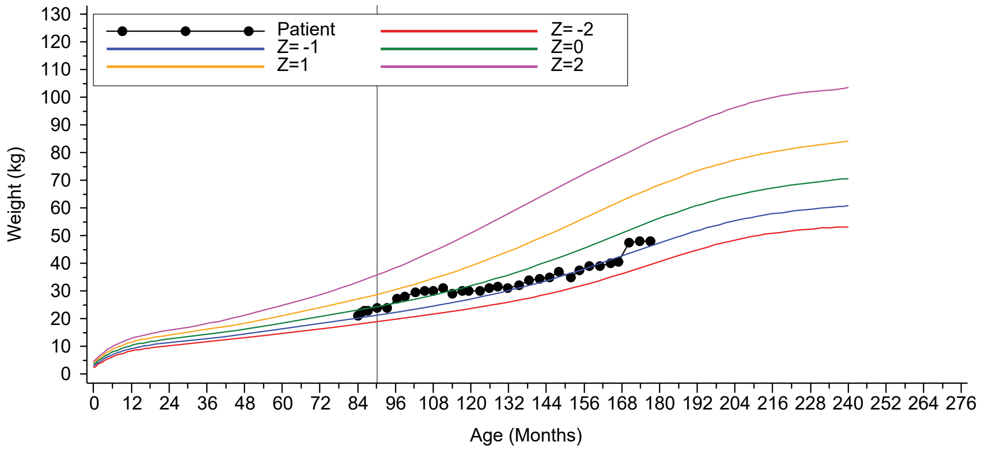

Patient 105

Seizure History: Primary Generalized Seizures and Unknown

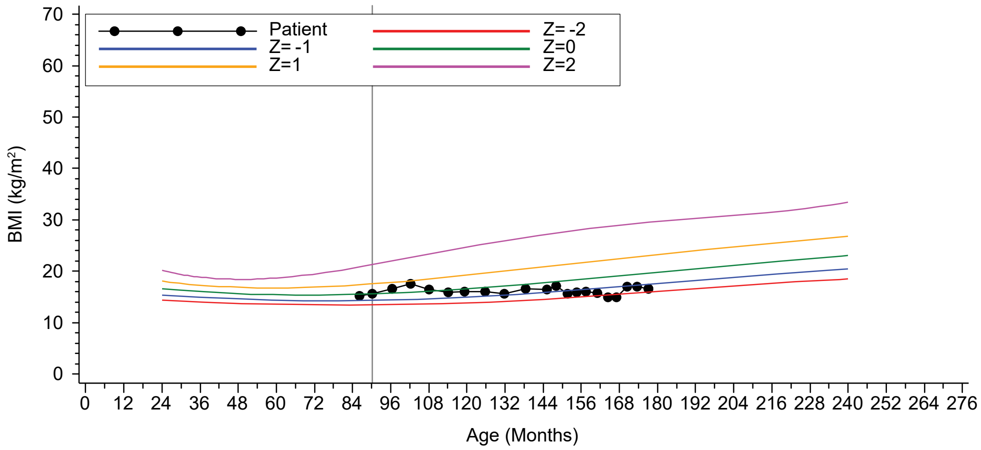

Patient 106

Seizure History: Primary Generalized Seizures and Unknown

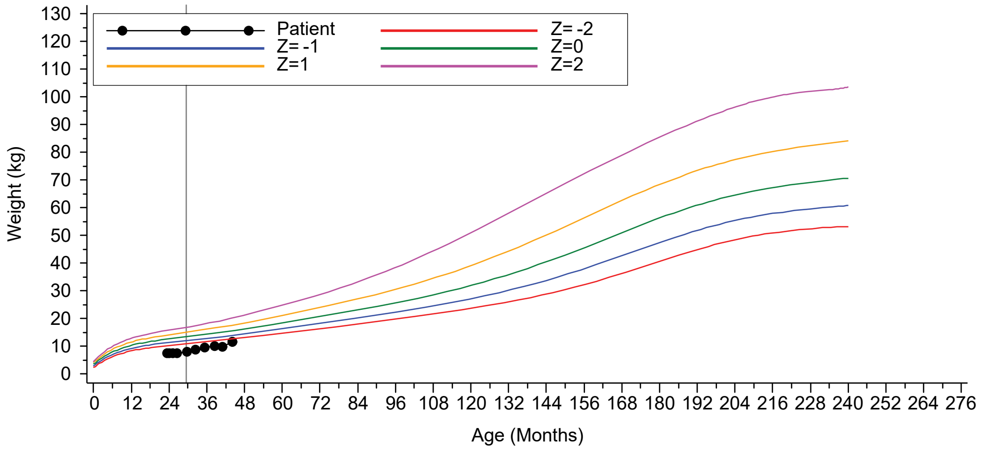

Patient 106

Seizure History: Primary Generalized Seizures and Unknown

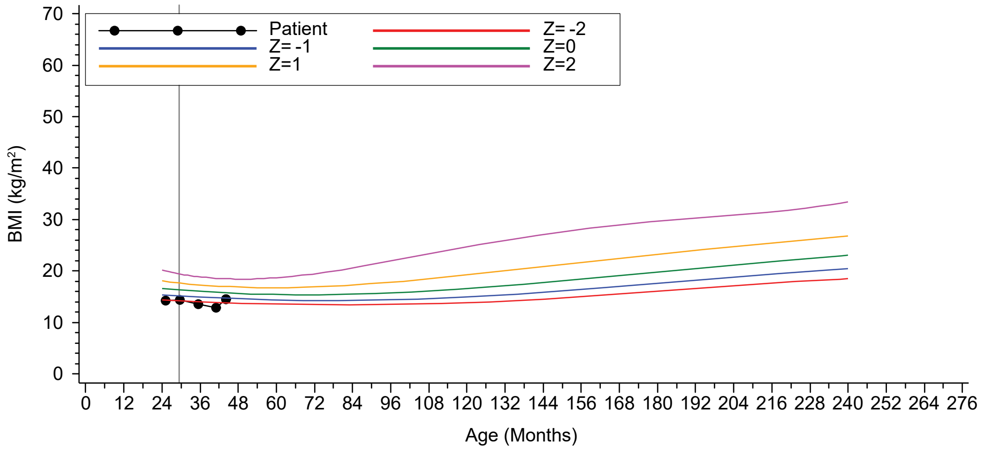

Patient 107

Seizure History: Primary Generalized Seizures and Unknown

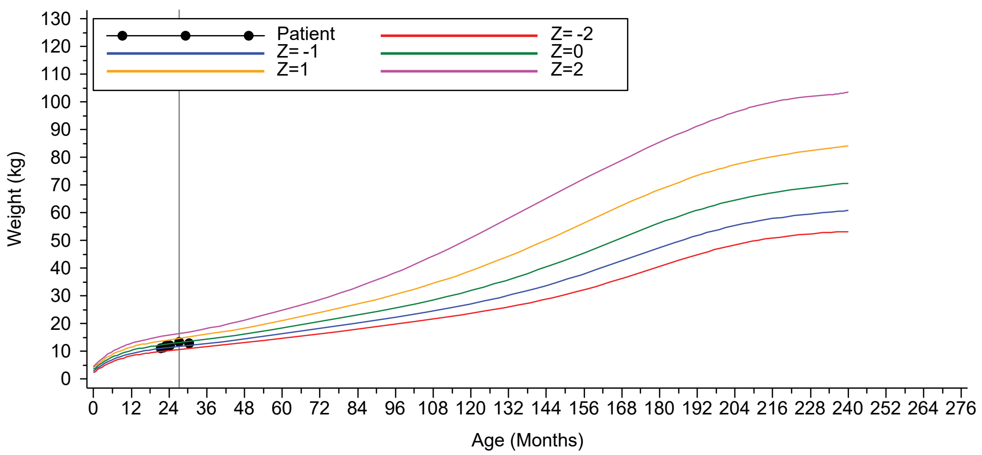

Patient 107

Seizure History: Primary Generalized Seizures and Unknown

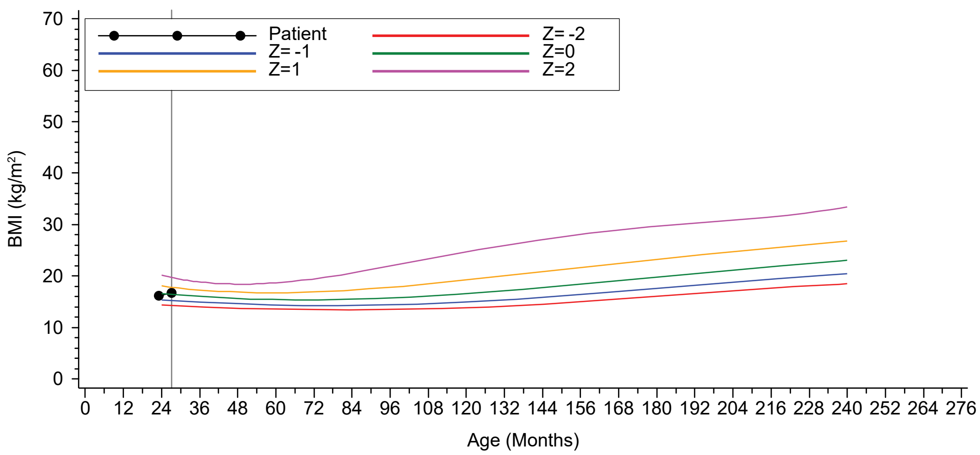

Patient 108

Seizure History: Primary Generalized Seizures and Unknown

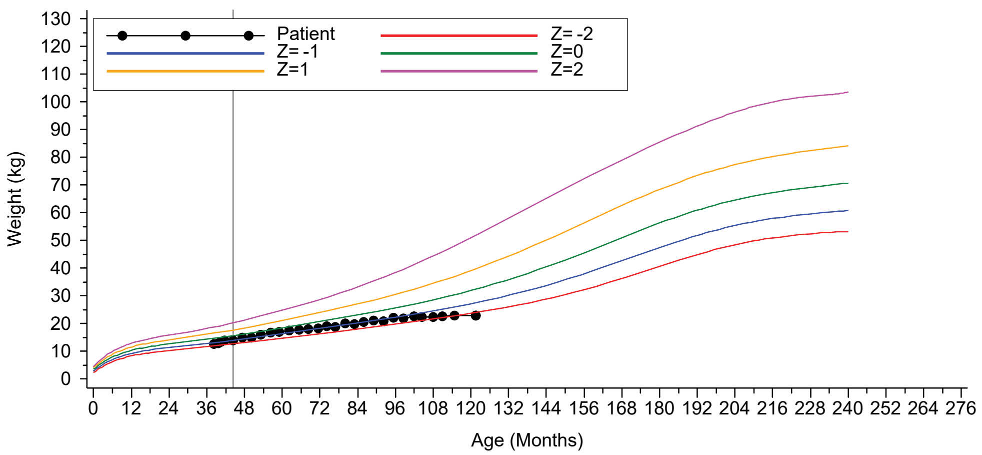

Patient 108

Seizure History: Primary Generalized Seizures and Unknown

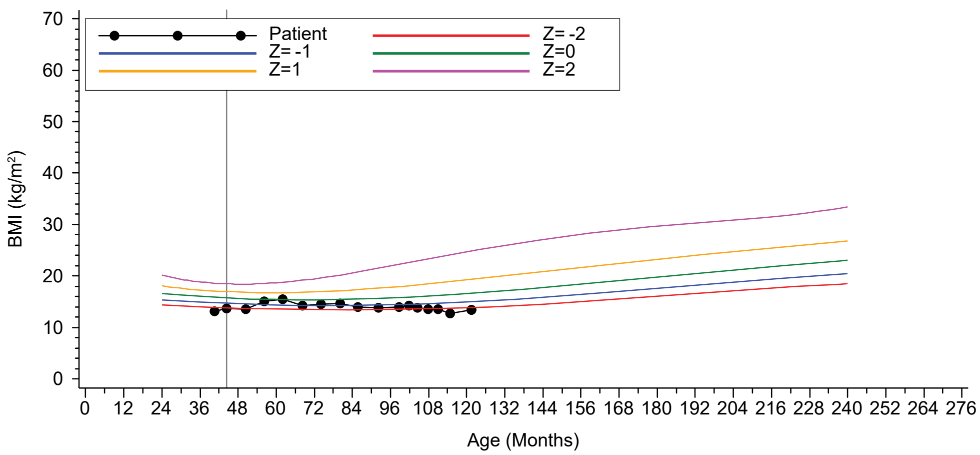

Patient 109

Seizure History: Primary Generalized Seizures and Unknown

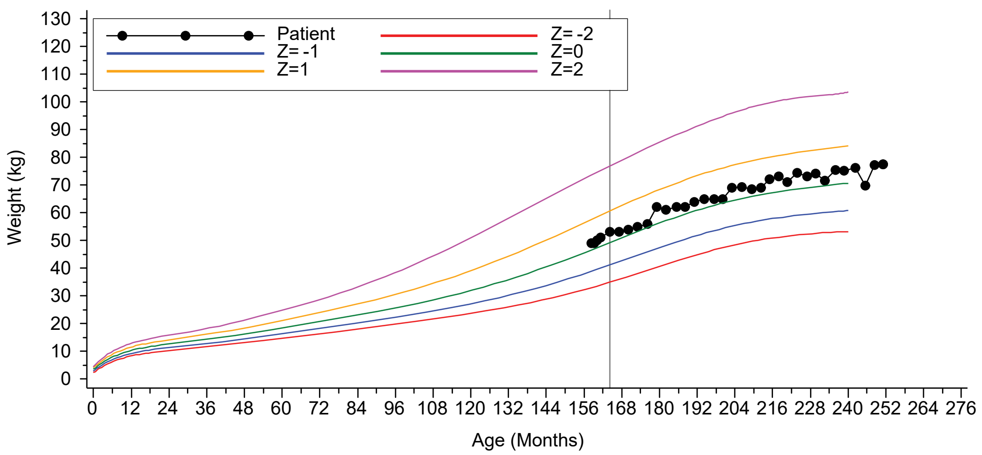

Patient 109

Seizure History: Primary Generalized Seizures and Unknown

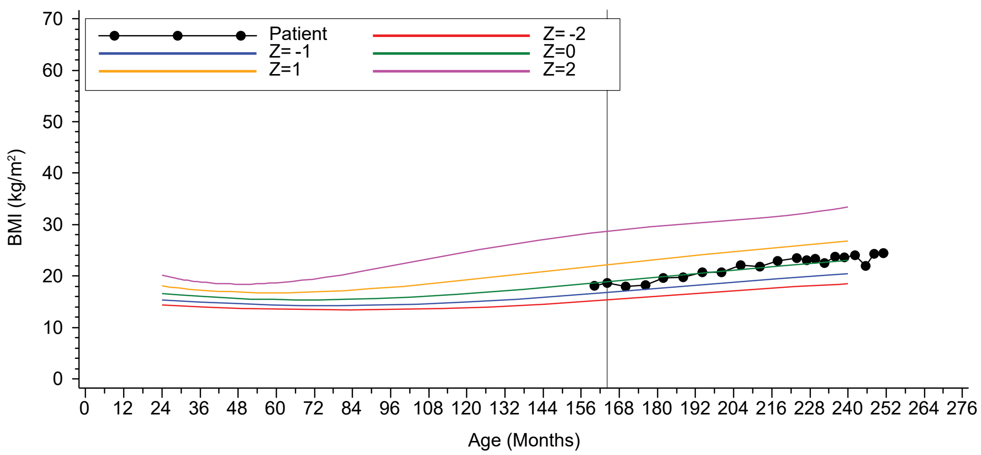

Patient 110

Seizure History: Primary Generalized Seizures and Unknown

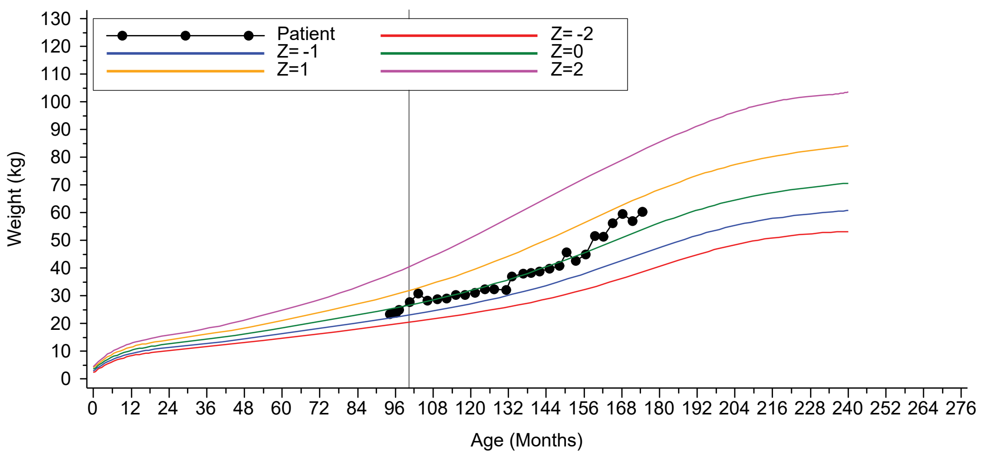

Patient 110

Seizure History: Primary Generalized Seizures and Unknown

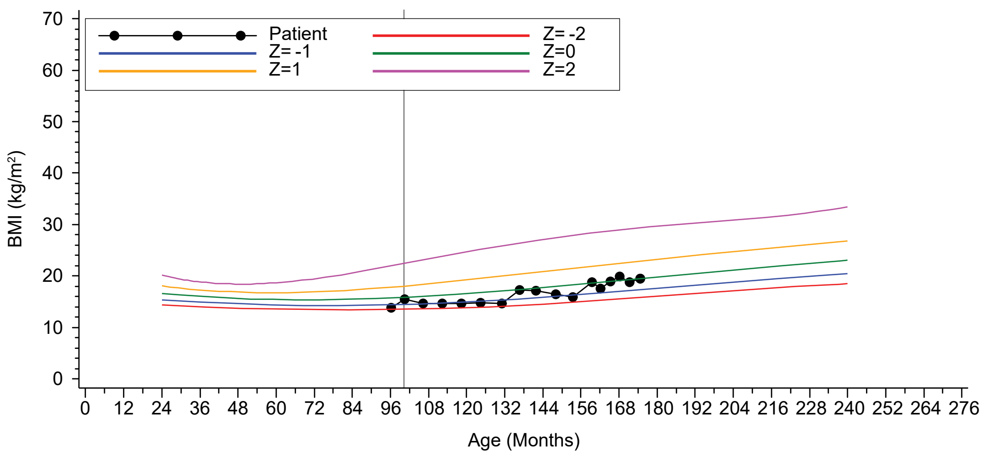

Patient 111

Seizure History: Primary Generalized Seizures and Unknown

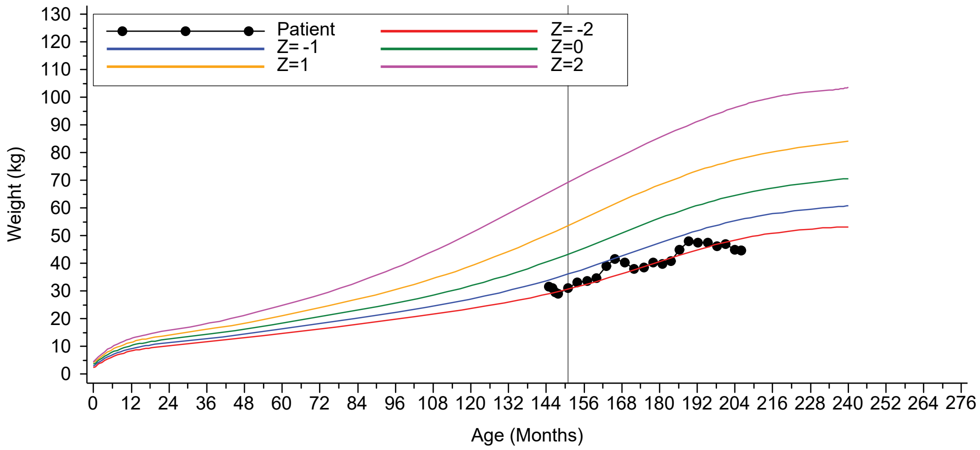

Patient 111

Seizure History: Primary Generalized Seizures and Unknown

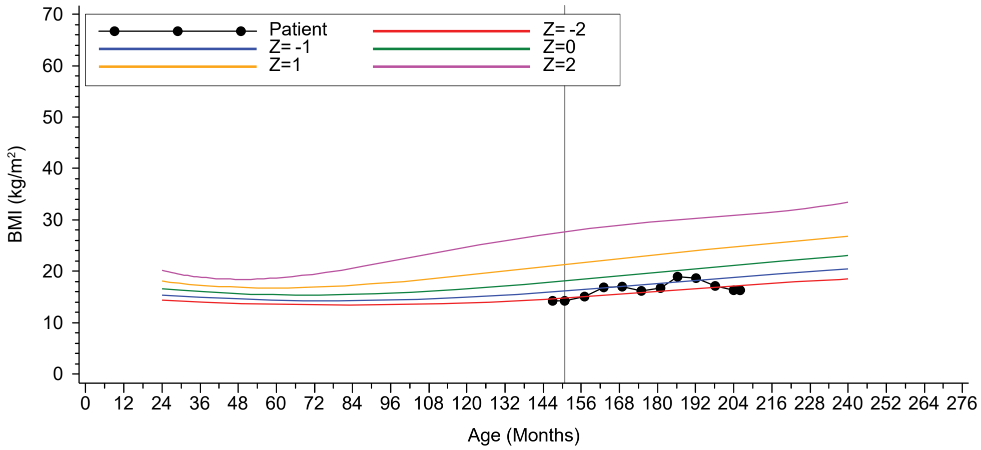

Patient 112

Seizure History: Primary Generalized Seizures and Unknown

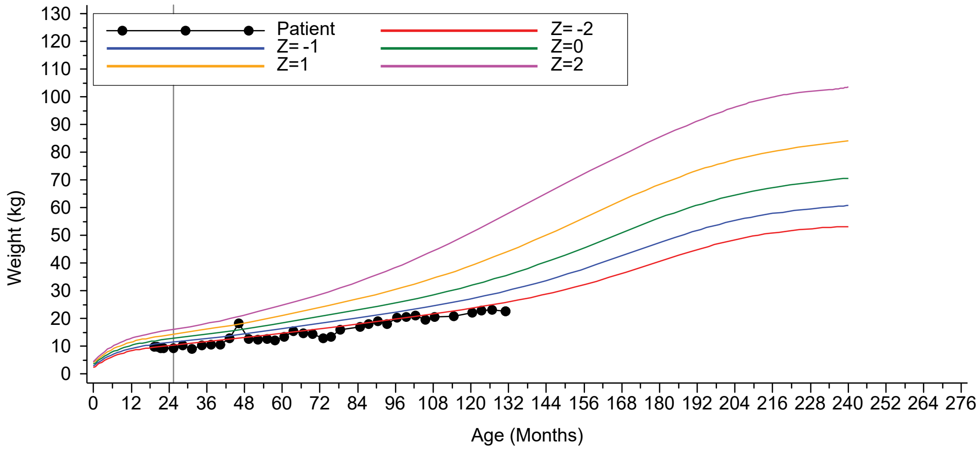

Patient 112

Seizure History: Primary Generalized Seizures and Unknown

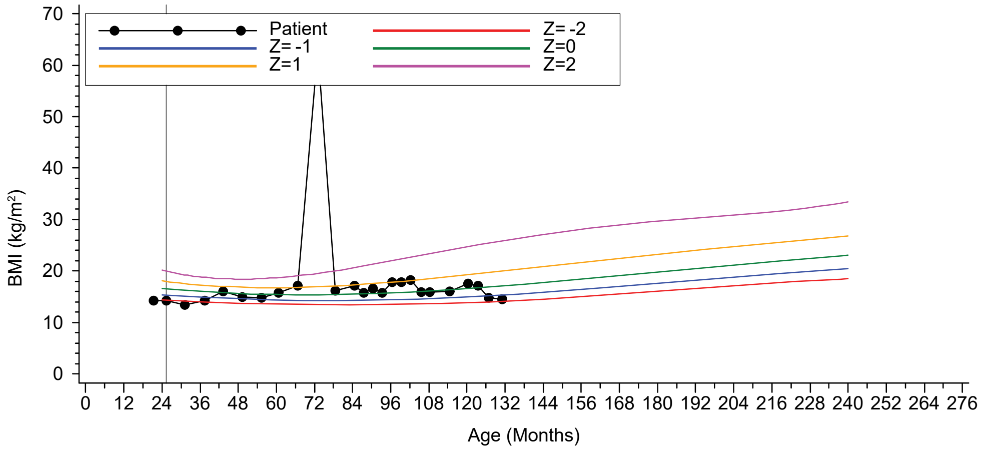

Patient 113

Seizure History: Primary Generalized Seizures and Unknown

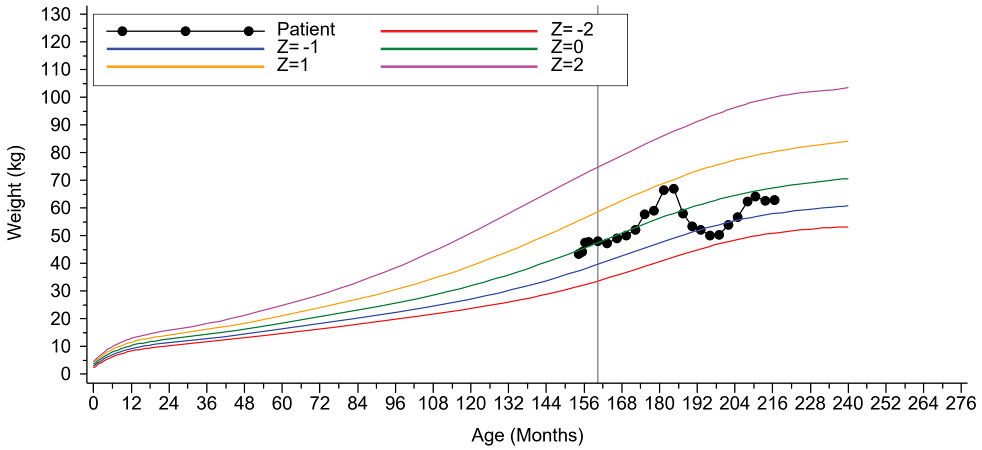

Patient 113

Seizure History: Primary Generalized Seizures and Unknown

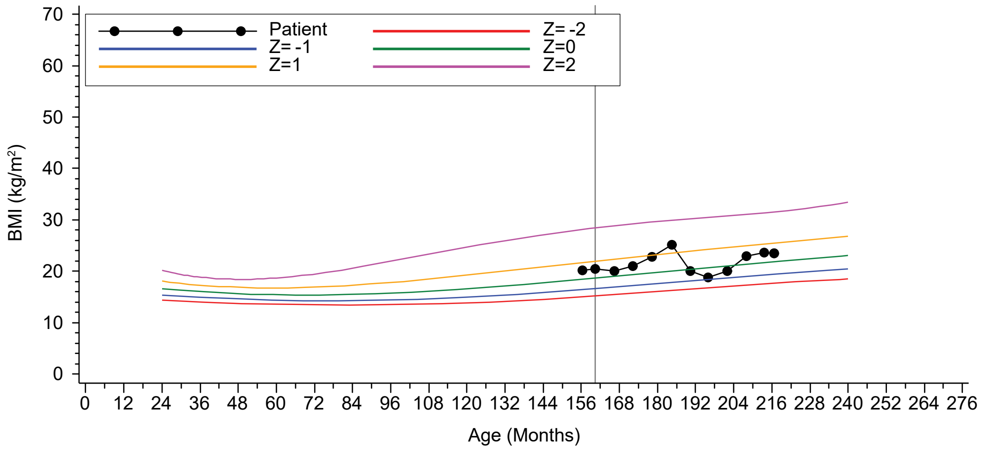

Patient 114

Seizure History: Partial Onset Seizures

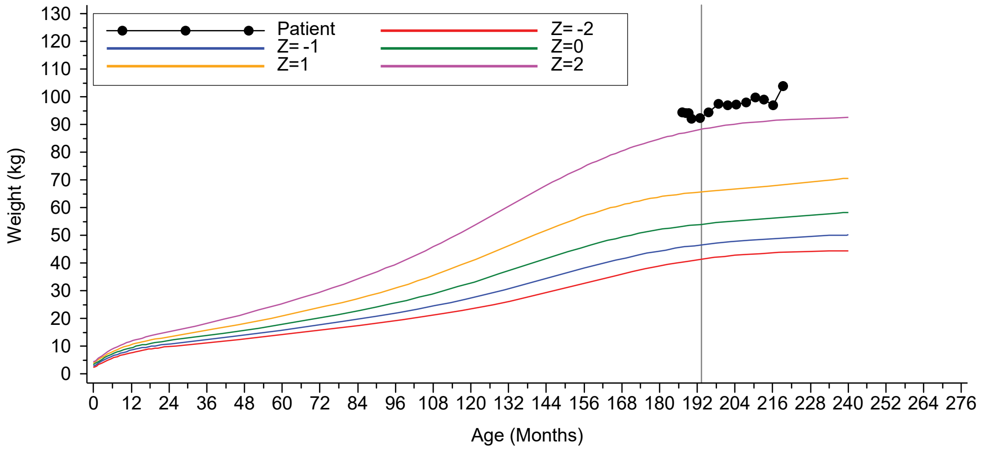

Patient 114

Seizure History: Partial Onset Seizures

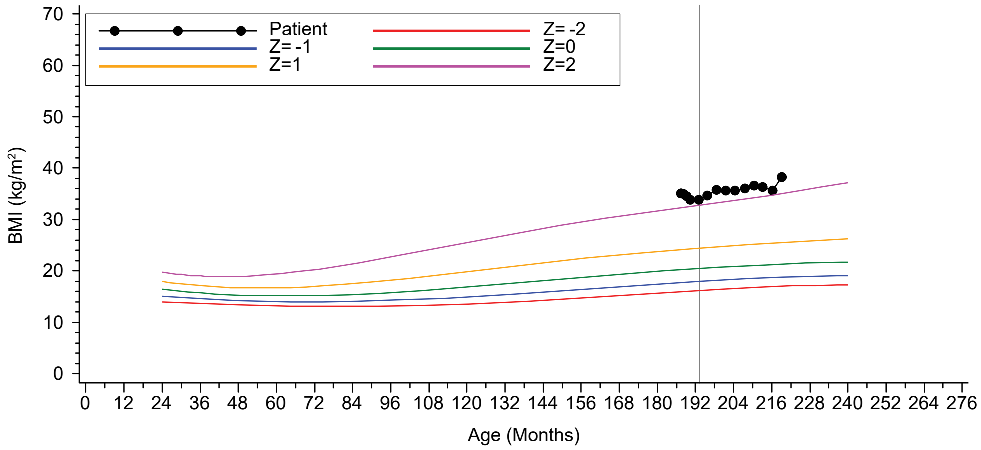

Patient 115

Seizure History: Partial Onset Seizures

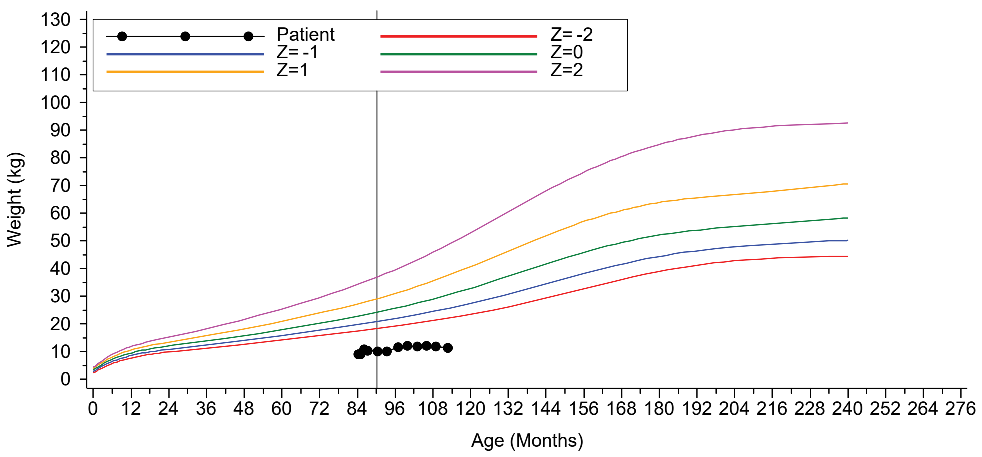

Patient 115

Seizure History: Partial Onset Seizures

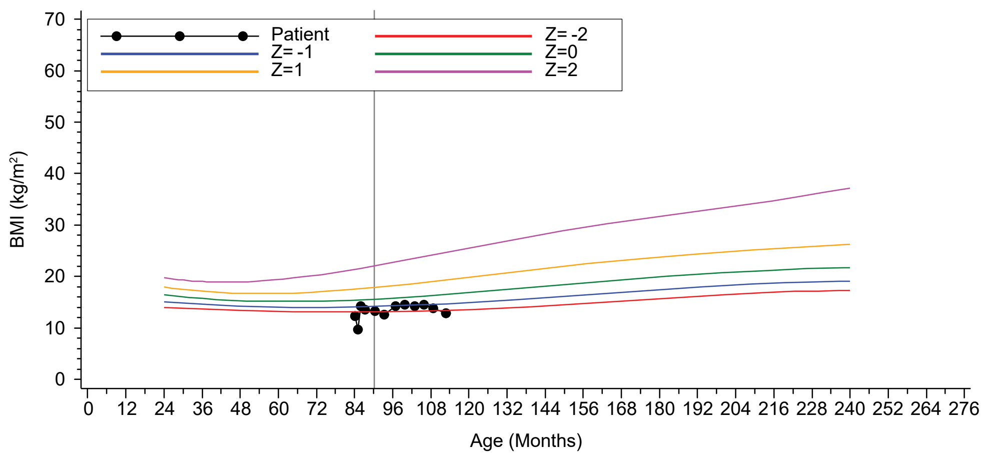

Patient 116

Seizure History: Partial Onset Seizures

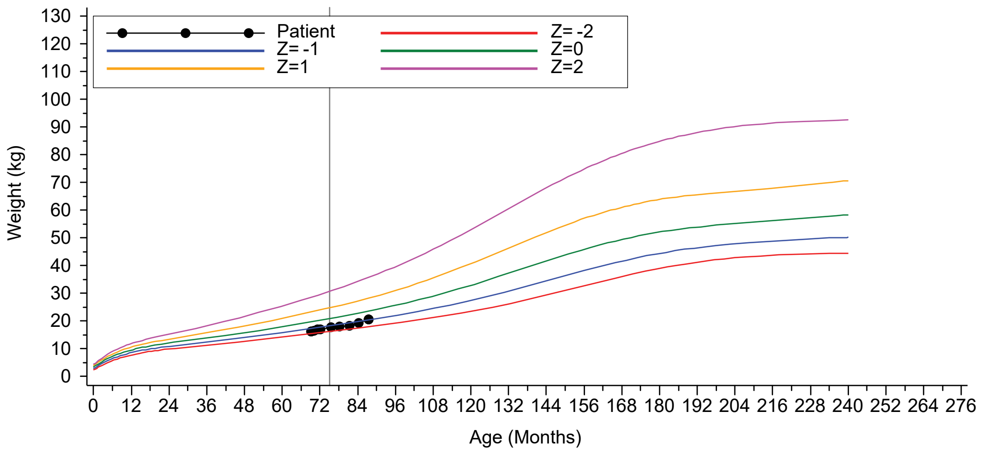

Patient 116

Seizure History: Partial Onset Seizures

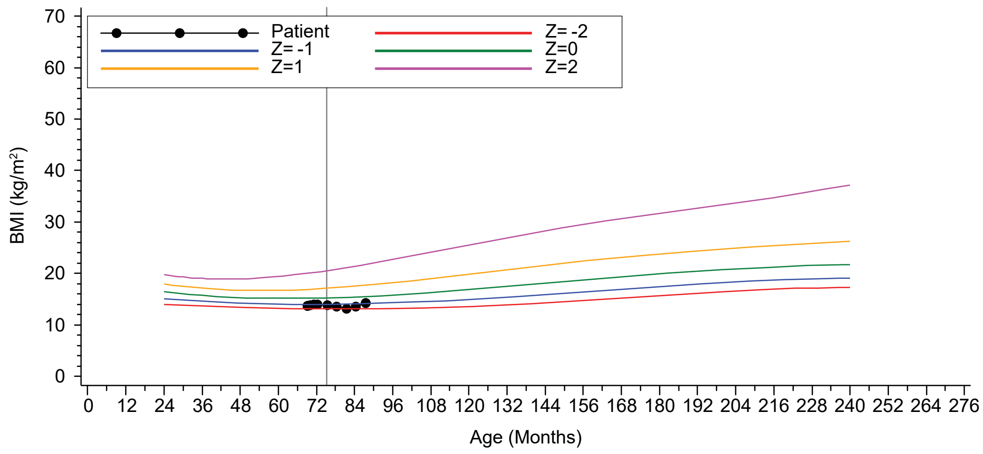

Patient 117

Seizure History: Partial Onset Seizures

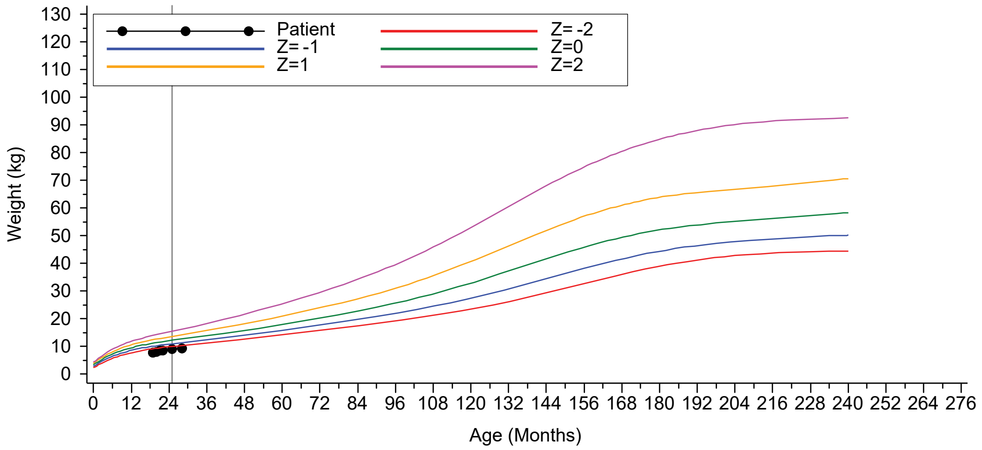

Patient 117

Seizure History: Partial Onset Seizures

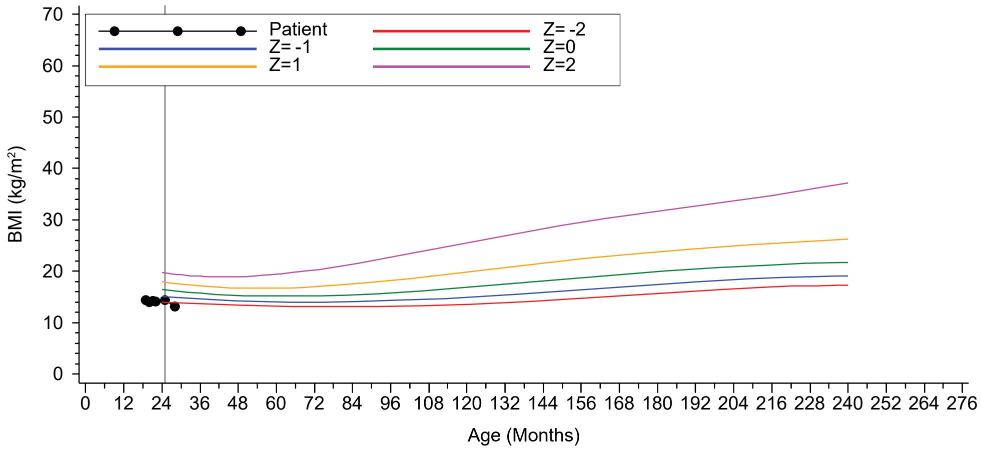

Patient 118

Seizure History: Partial Onset Seizures

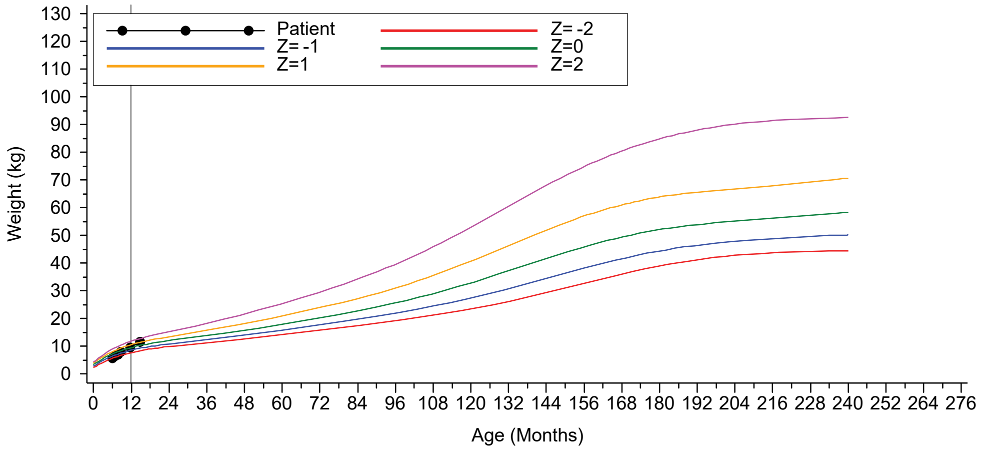

Patient 118

Seizure History: Partial Onset Seizures

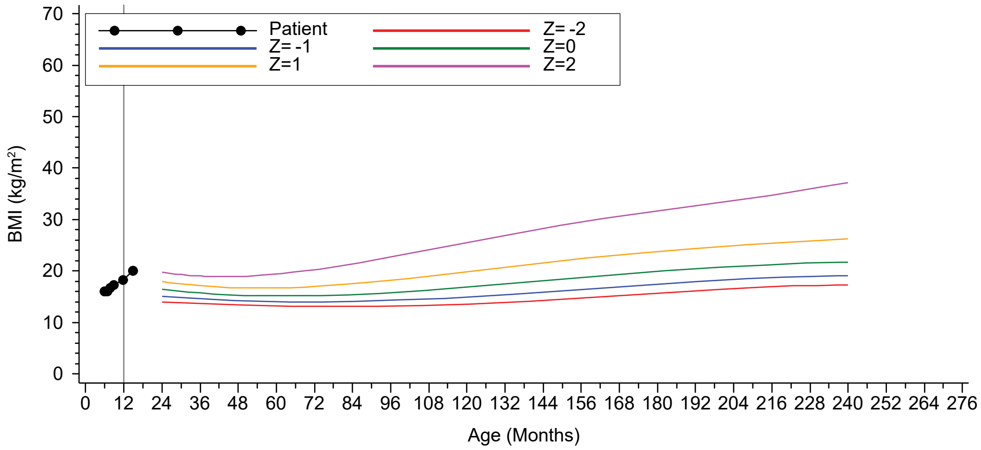

Patient 119

Seizure History: Partial Onset Seizures

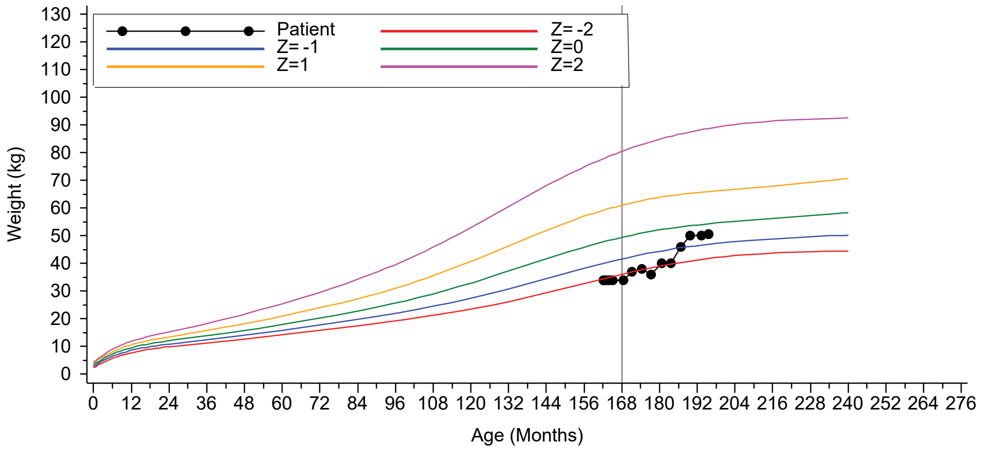

Patient 119

Seizure History: Partial Onset Seizures

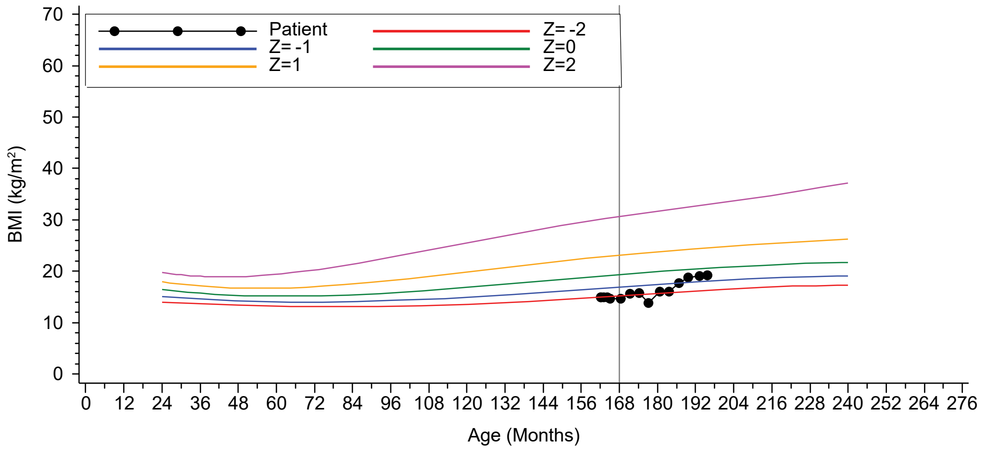

Patient 120

Seizure History: Partial Onset Seizures

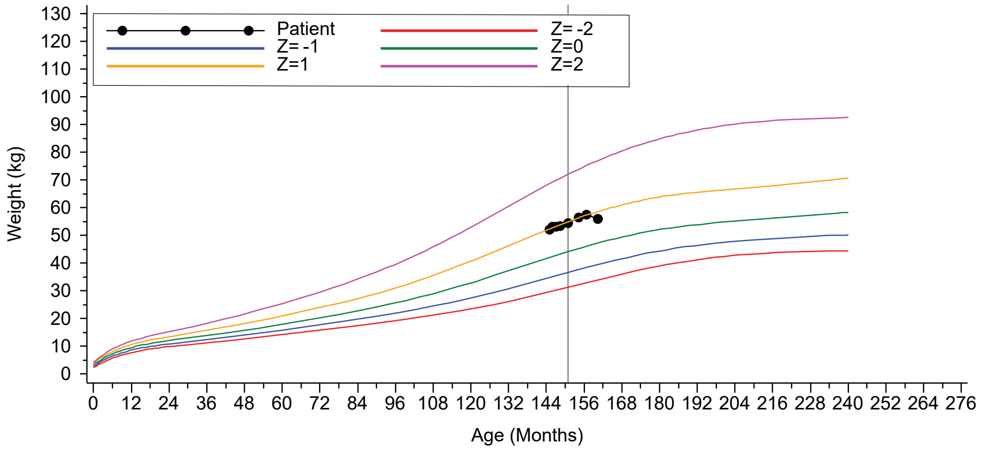

Patient 120

Seizure History: Partial Onset Seizures

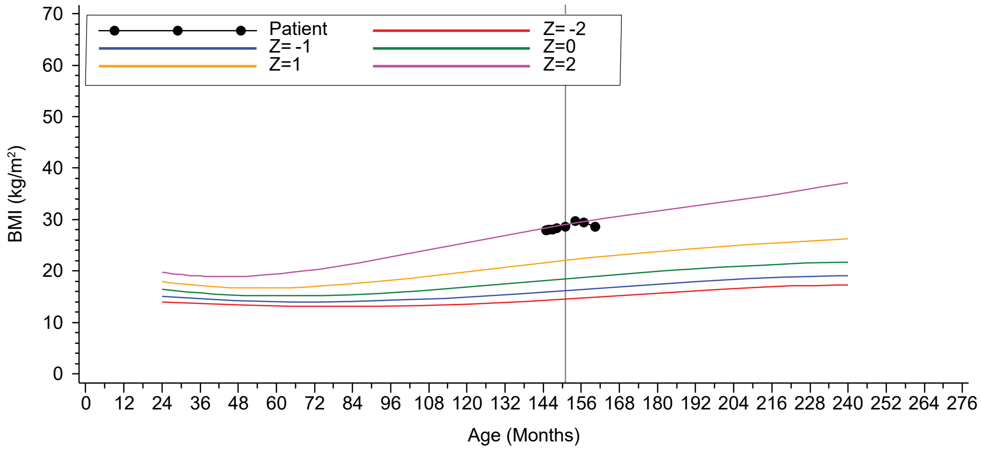

Patient 121

Seizure History: Partial Onset Seizures

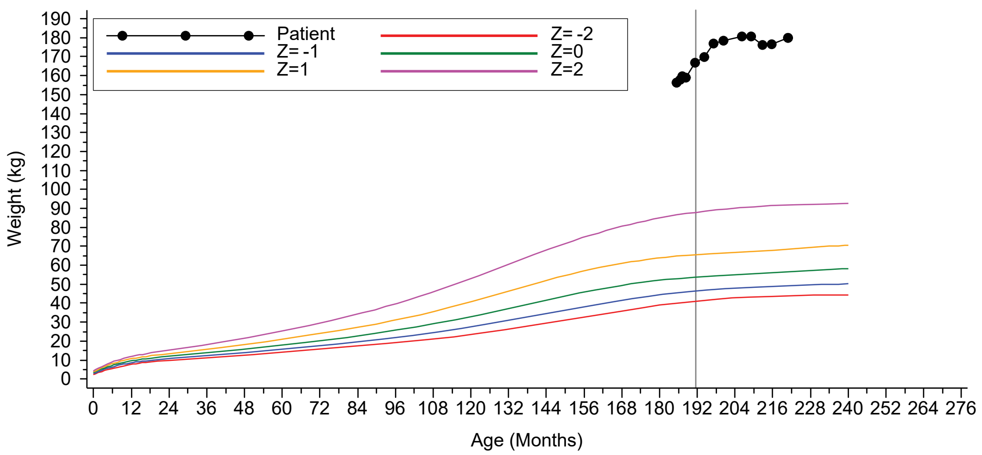

Patient 121

Seizure History: Partial Onset Seizures

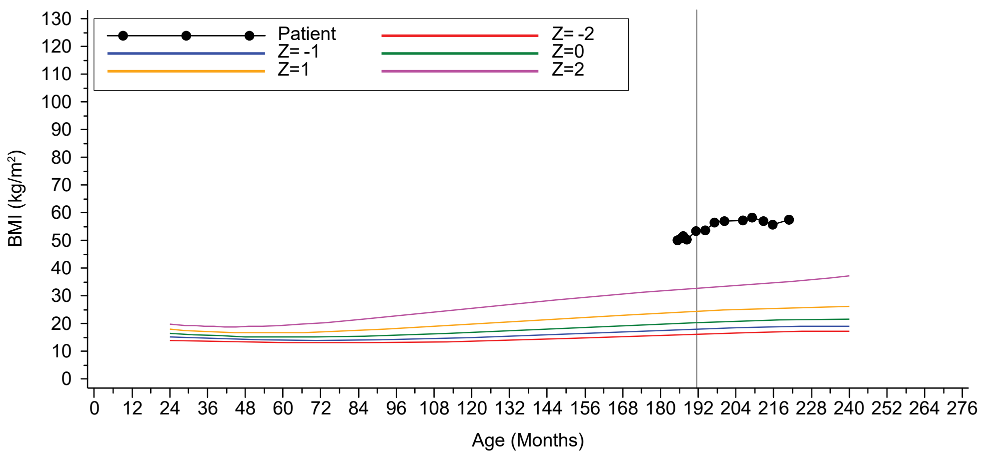

Patient 122

Seizure History: Partial Onset Seizures

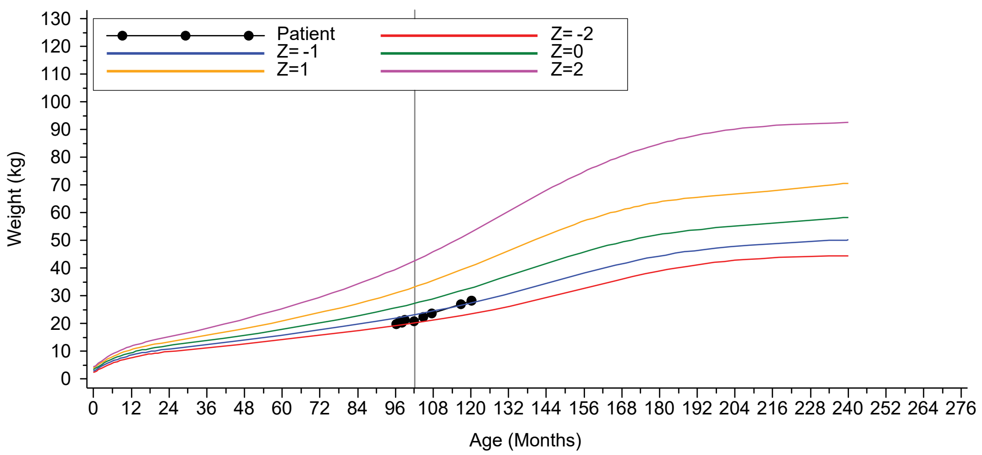

Patient 122

Seizure History: Partial Onset Seizures

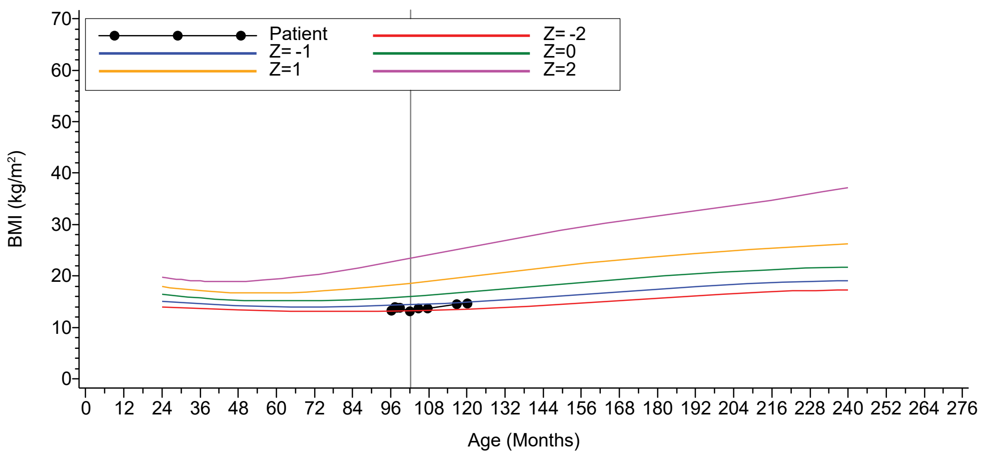

Patient 123

Seizure History: Partial Onset Seizures

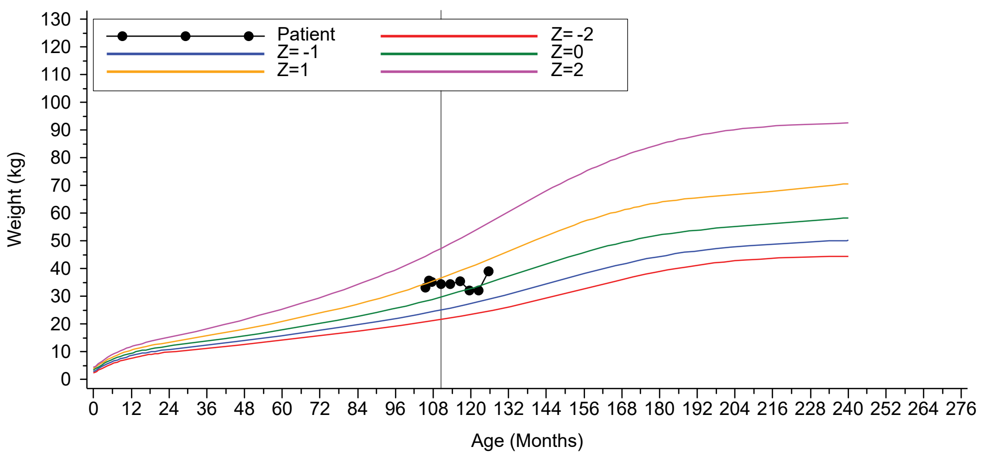

Patient 123

Seizure History: Partial Onset Seizures

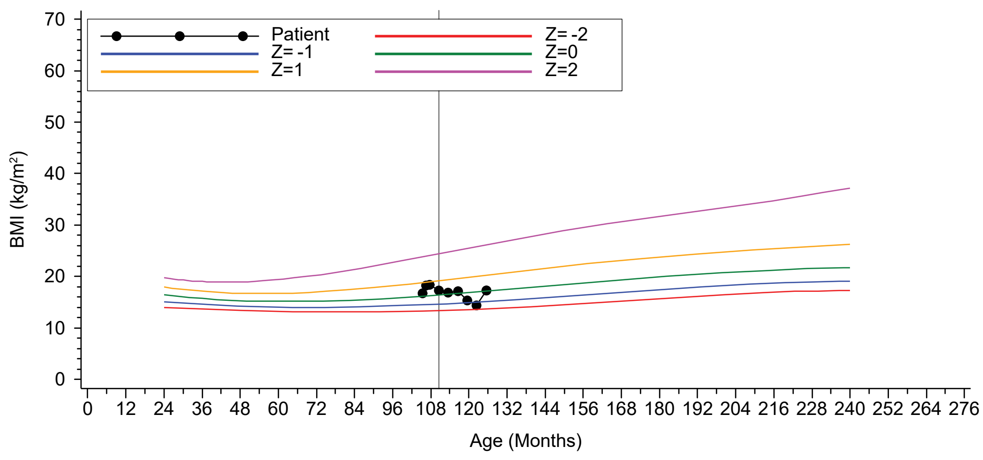

Patient 124

Seizure History: Partial Onset Seizures

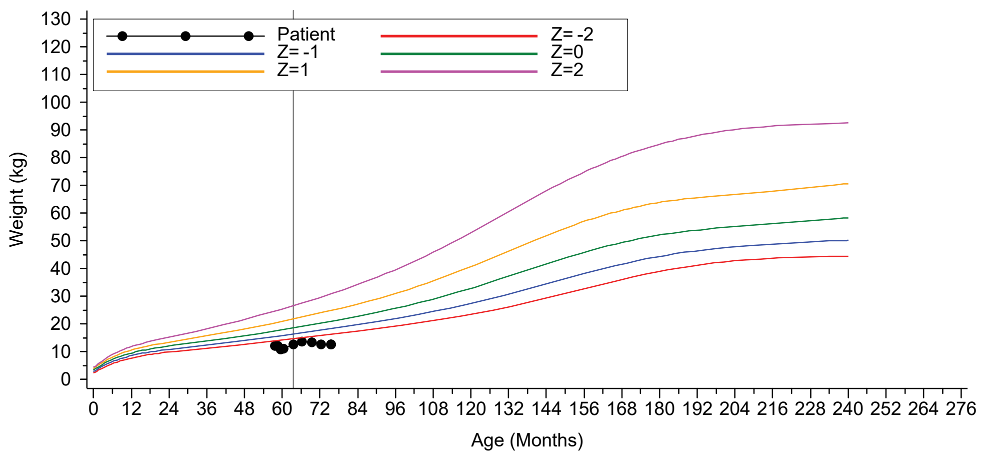

Patient 124

Seizure History: Partial Onset Seizures

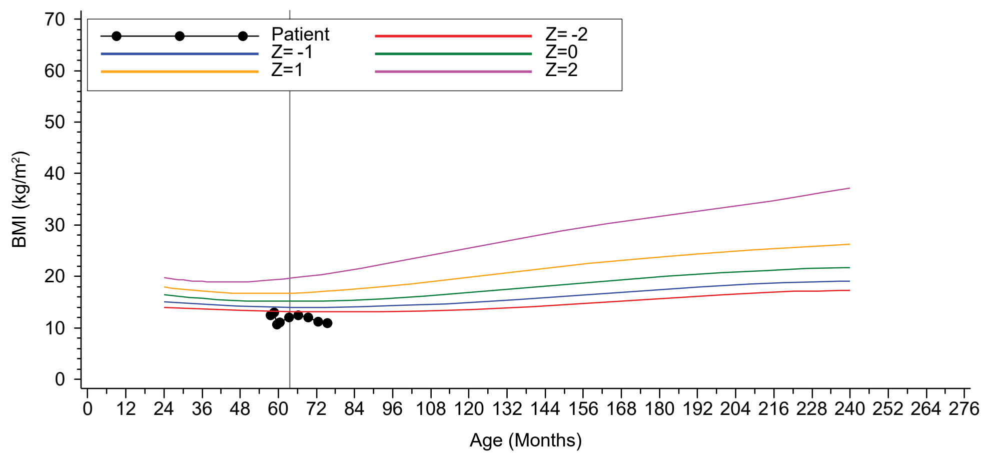

Patient 125

Seizure History: Partial Onset Seizures

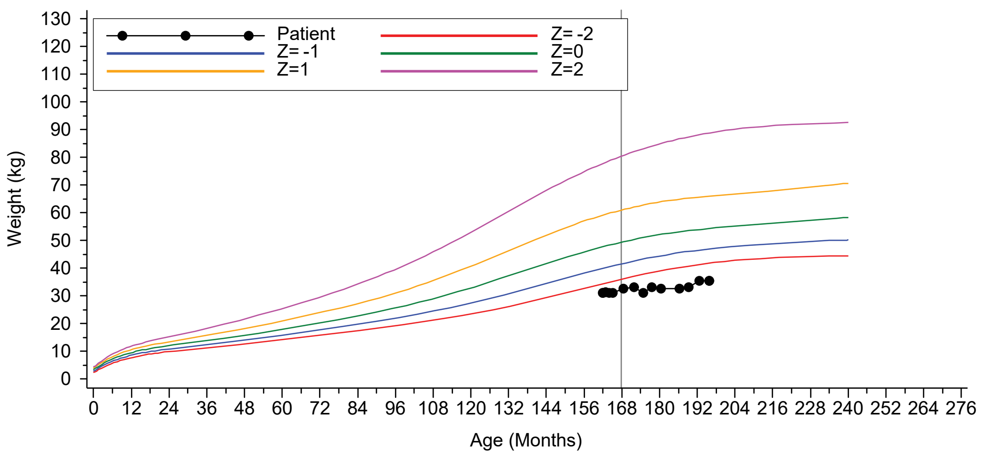

Patient 125

Seizure History: Partial Onset Seizures

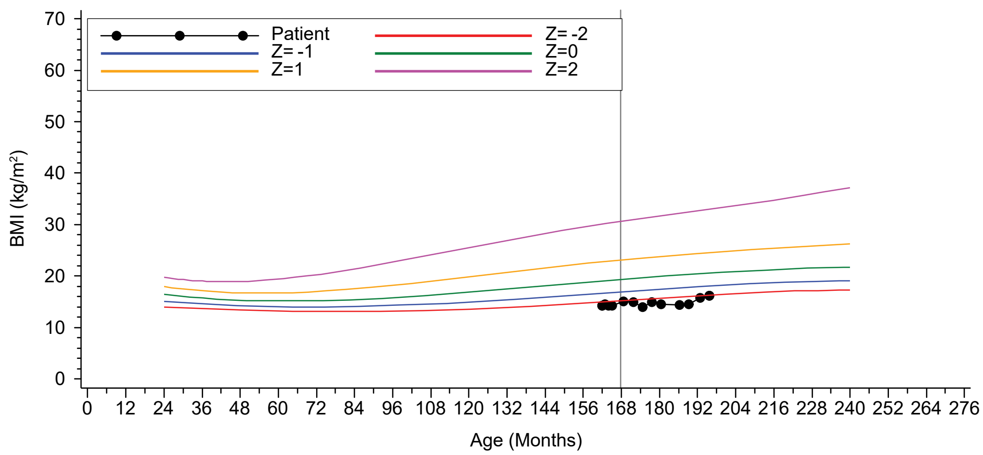

Patient 126

Seizure History: Partial Onset Seizures

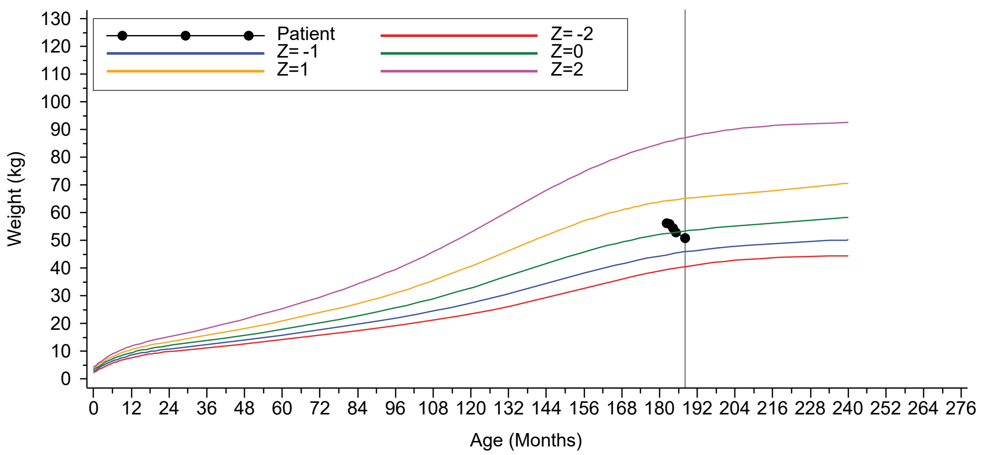

Patient 126

Seizure History: Partial Onset Seizures

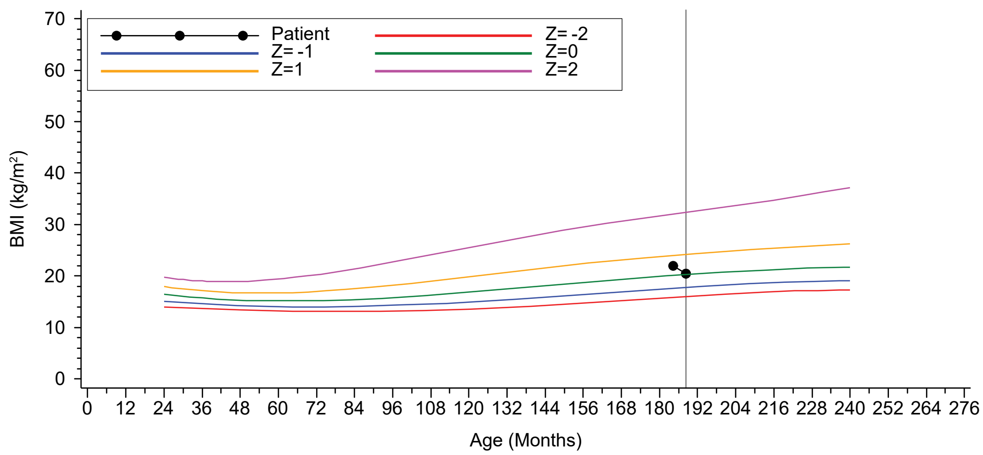

Patient 127

Seizure History: Partial Onset Seizures

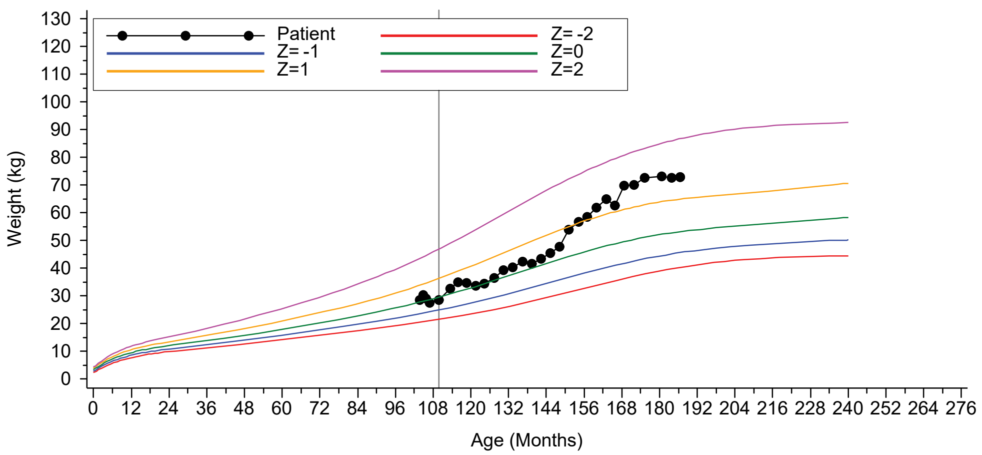

Patient 127

Seizure History: Partial Onset Seizures

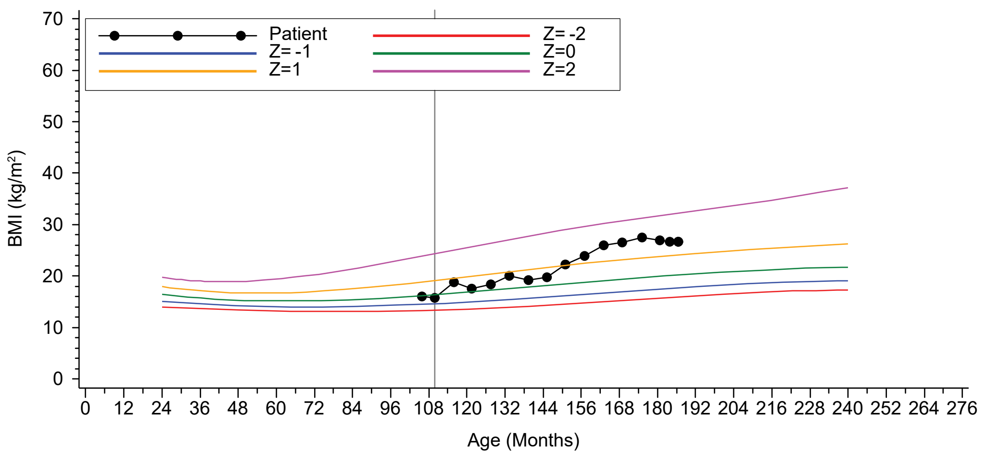

Patient 128

Seizure History: Partial Onset Seizures

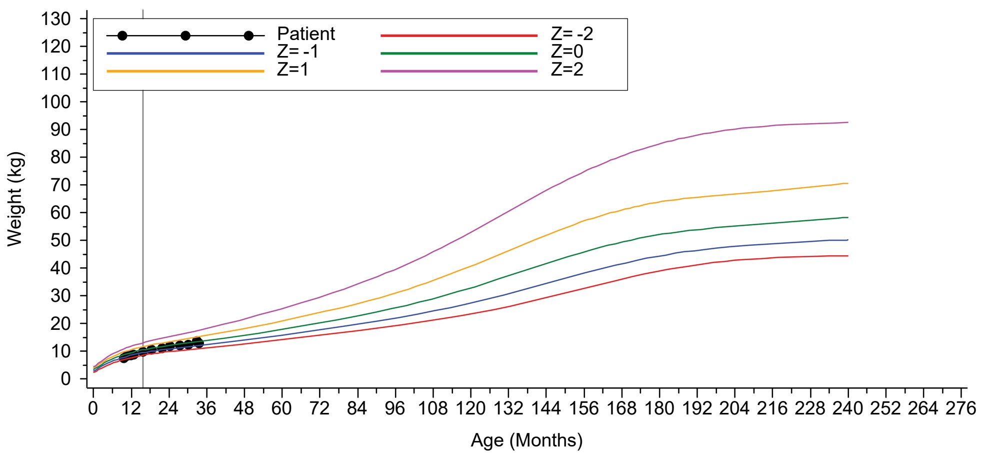

Patient 128

Seizure History: Partial Onset Seizures

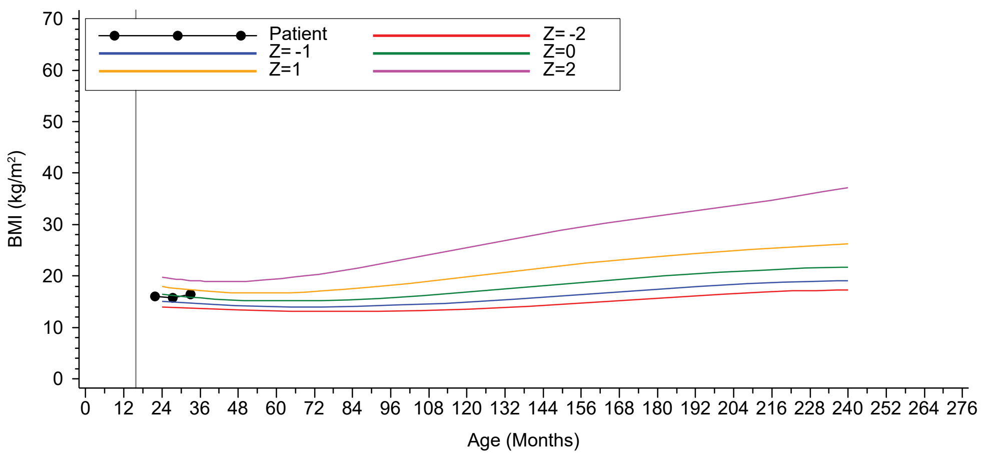

Patient 129

Seizure History: Partial Onset Seizures

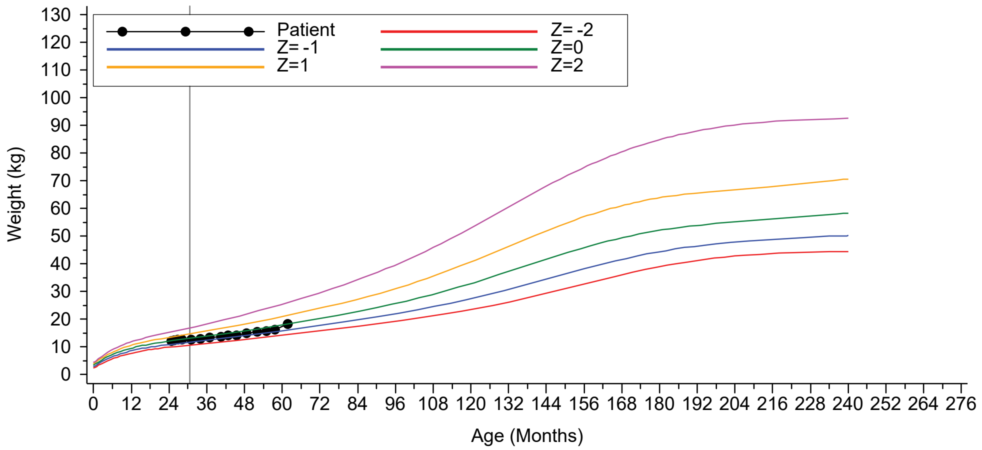

Patient 129

Seizure History: Partial Onset Seizures

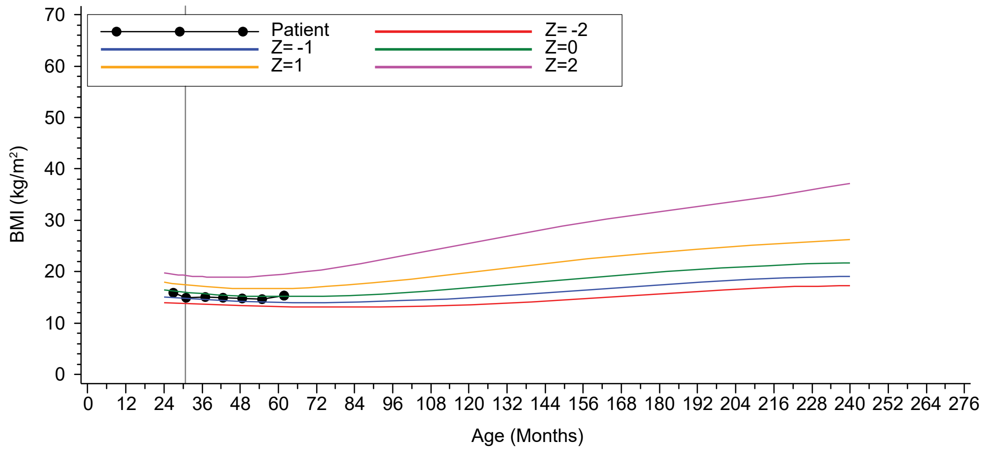

Patient 130

Seizure History: Partial Onset Seizures

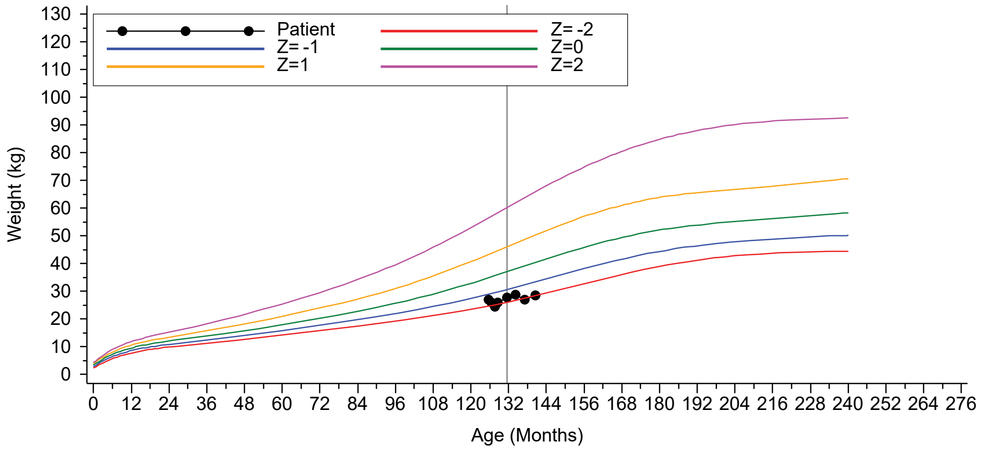

Patient 130

Seizure History: Partial Onset Seizures

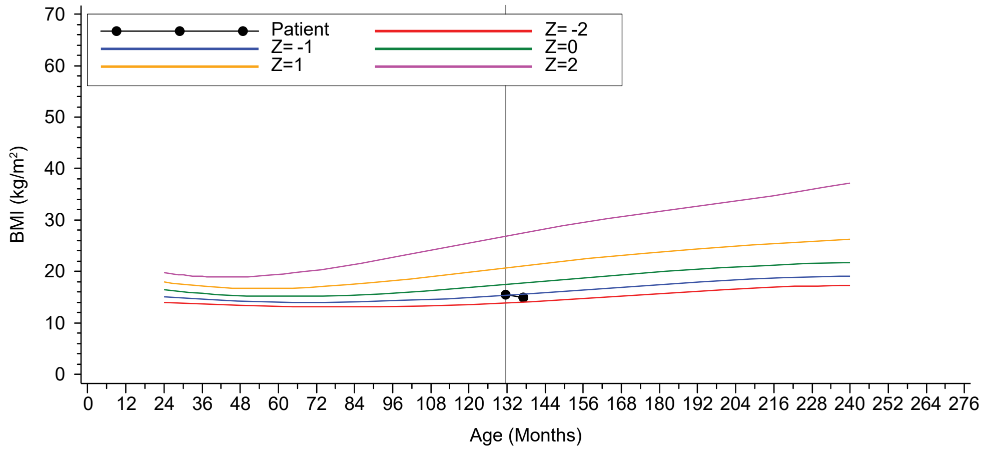

Patient 131

Seizure History: Partial Onset Seizures

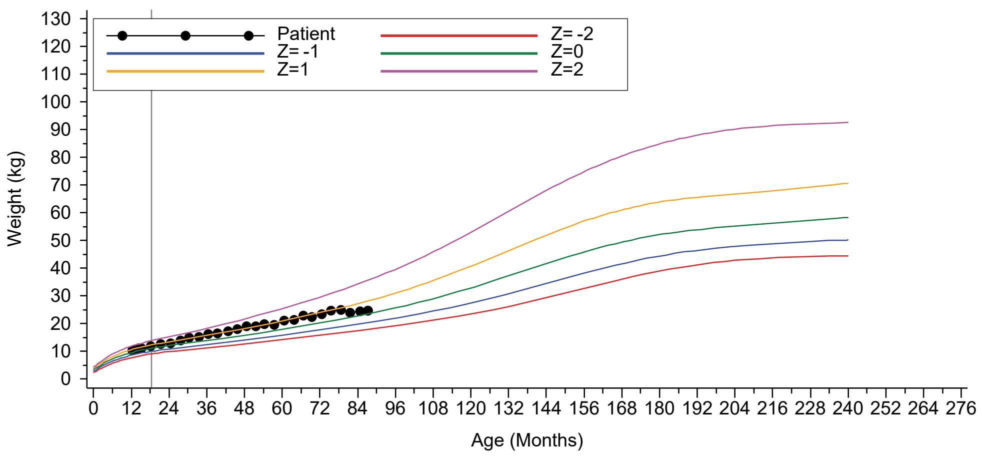

Patient 131

Seizure History: Partial Onset Seizures

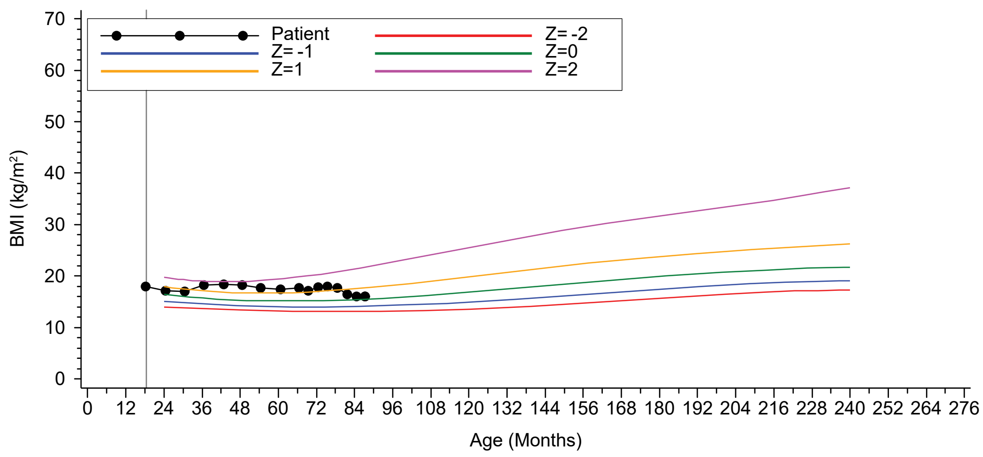

Patient 132

Seizure History: Partial Onset Seizures

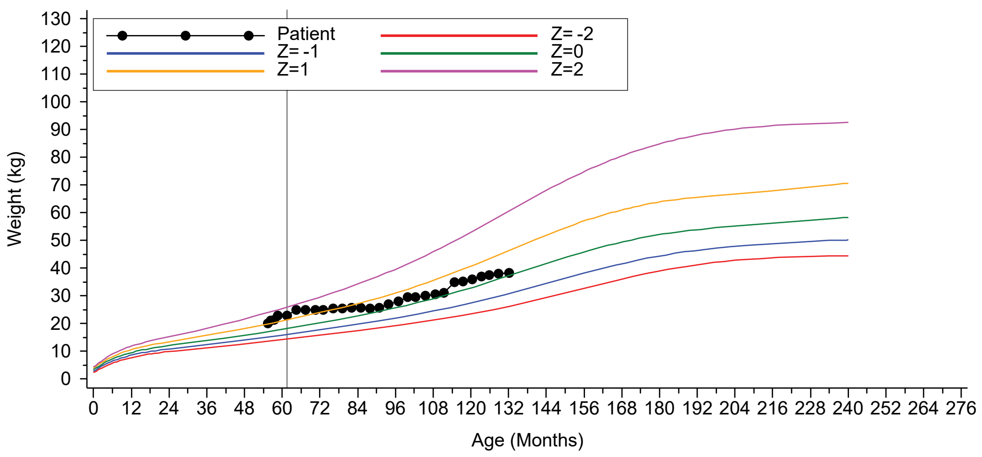

Patient 132

Seizure History: Partial Onset Seizures

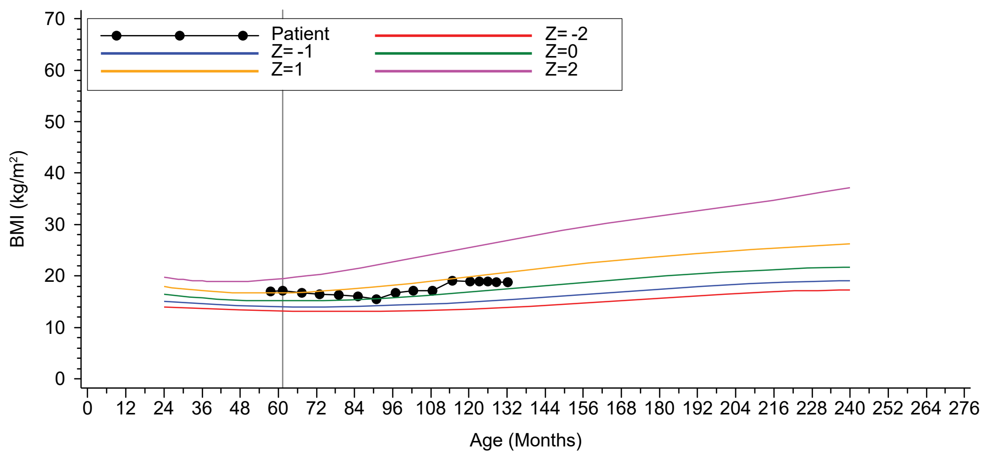

Patient 133

Seizure History: Partial Onset Seizures

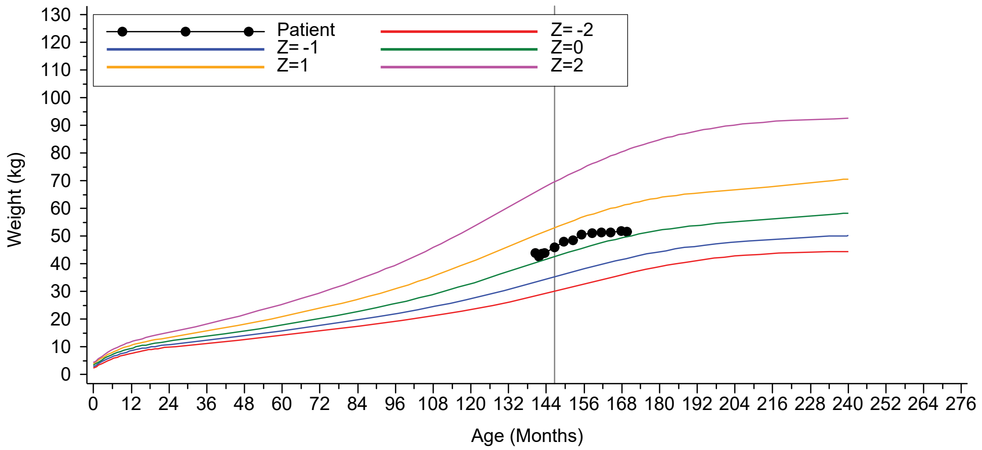

Patient 133

Seizure History: Partial Onset Seizures

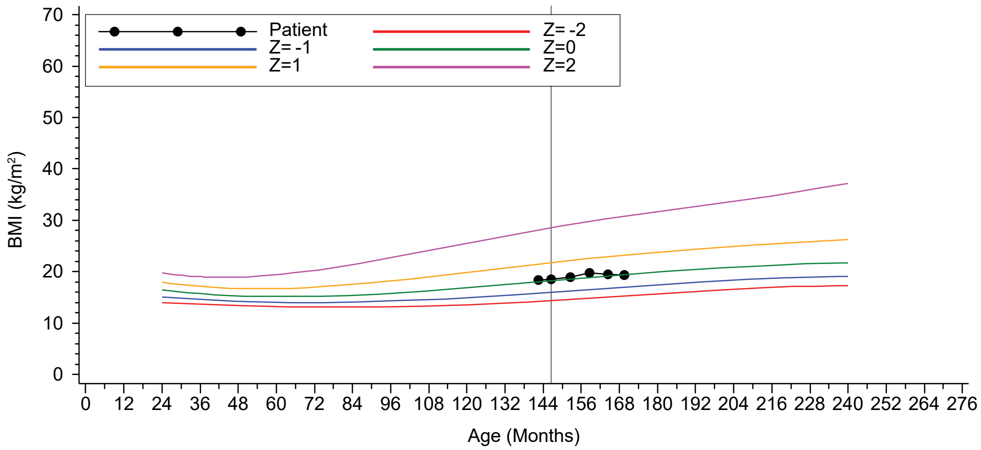

Patient 134

Seizure History: Partial Onset Seizures

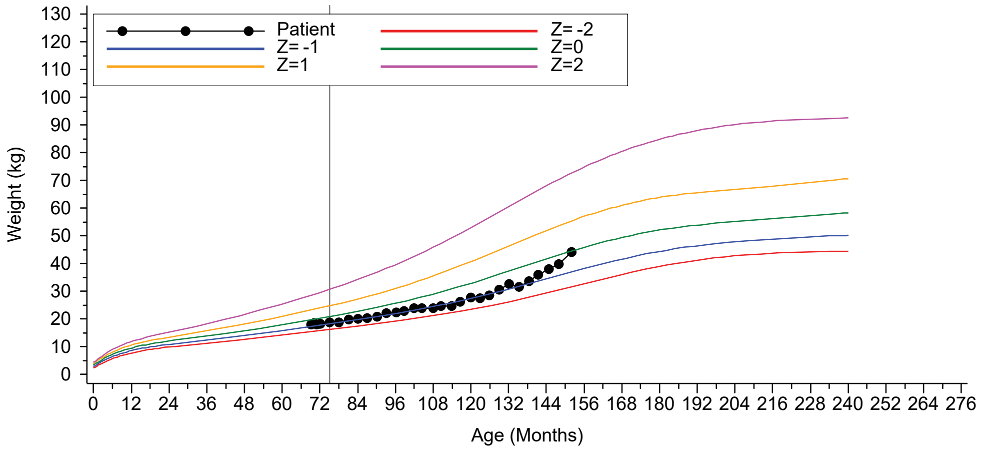

Patient 134

Seizure History: Partial Onset Seizures

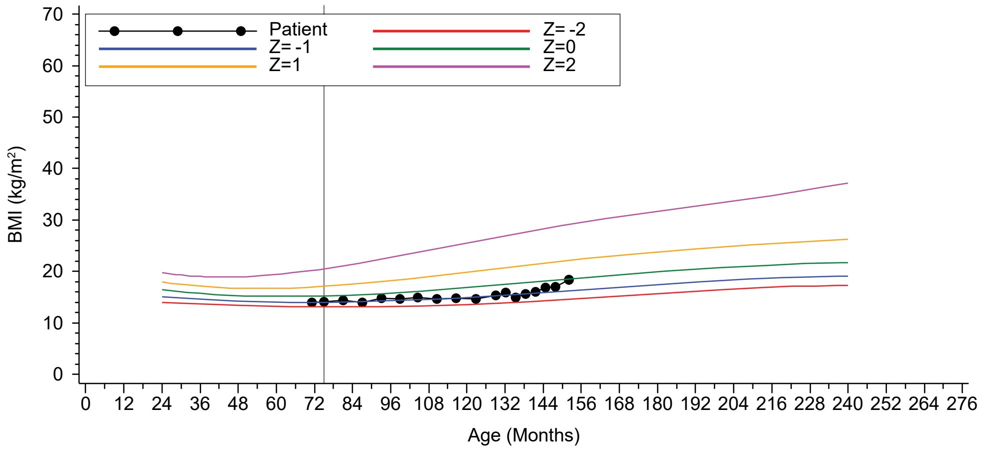

Patient 135

Seizure History: Partial Onset Seizures

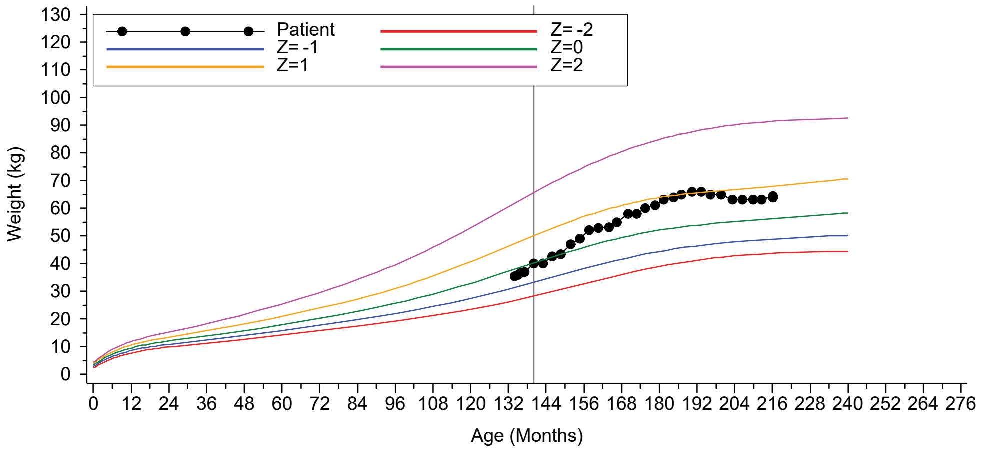

Patient 135

Seizure History: Partial Onset Seizures

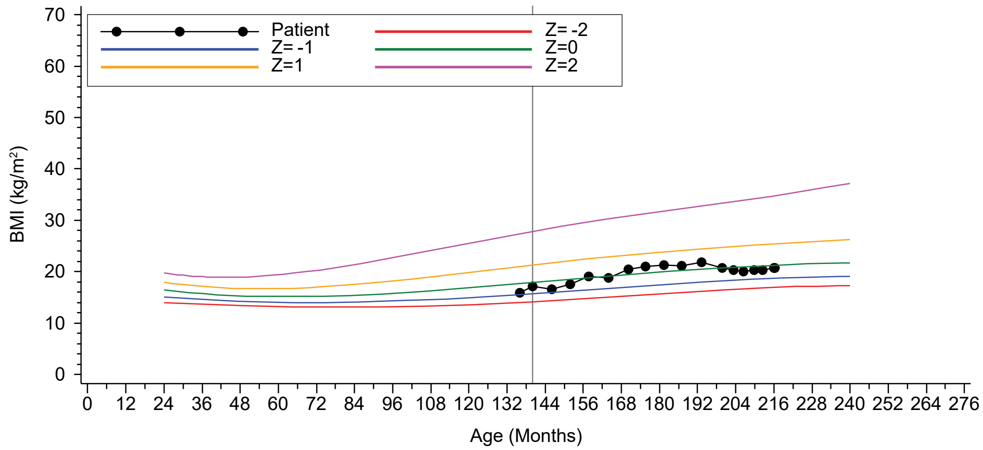

Patient 136

Seizure History: Partial Onset Seizures

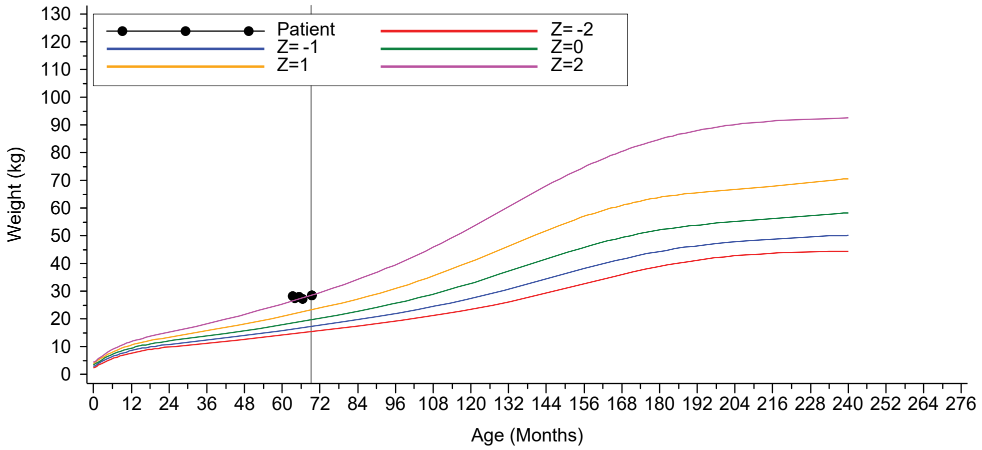

Patient 136

Seizure History: Partial Onset Seizures

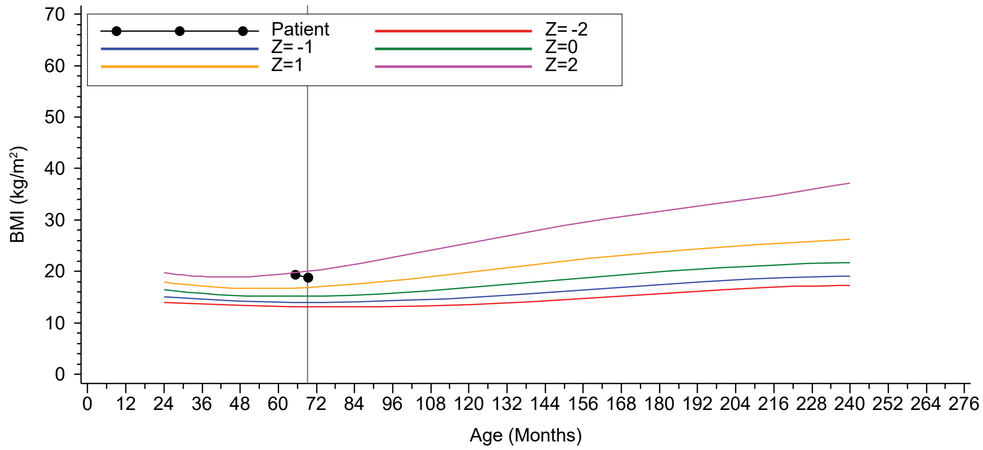

Patient 137

Seizure History: Partial Onset Seizures

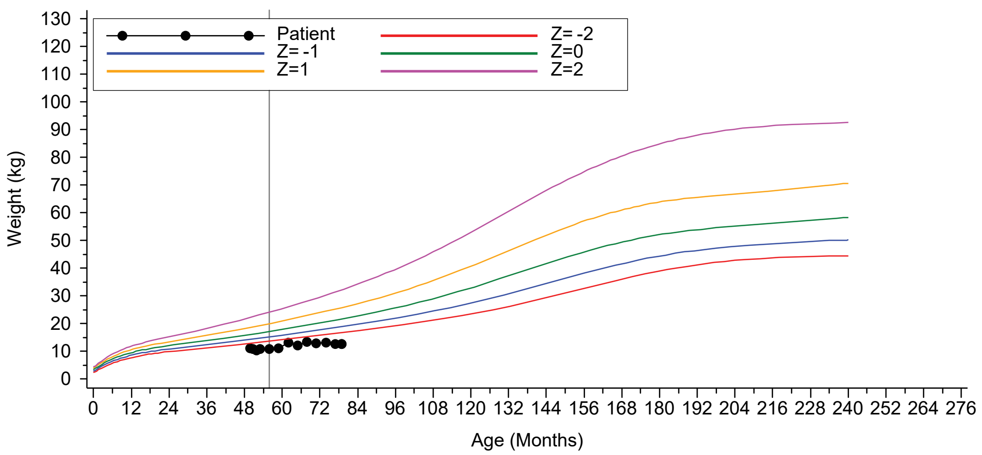

Patient 137

Seizure History: Partial Onset Seizures

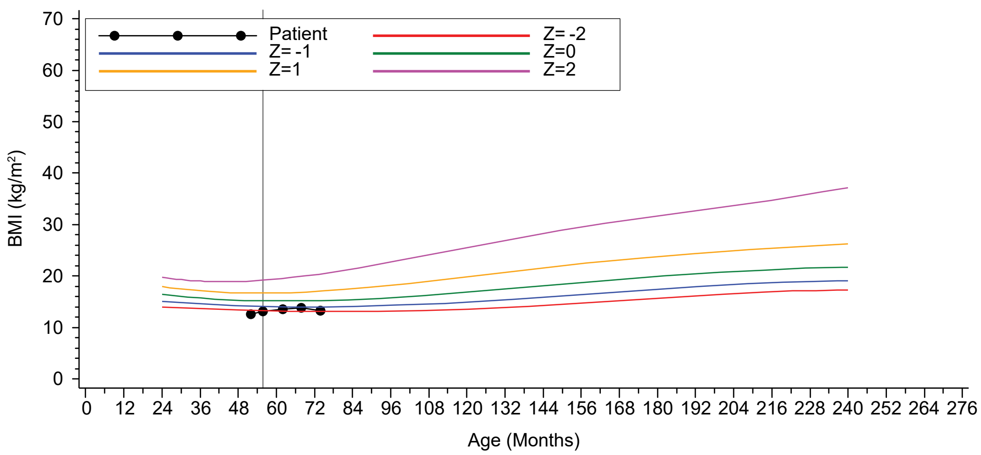

Patient 138

Seizure History: Partial Onset Seizures

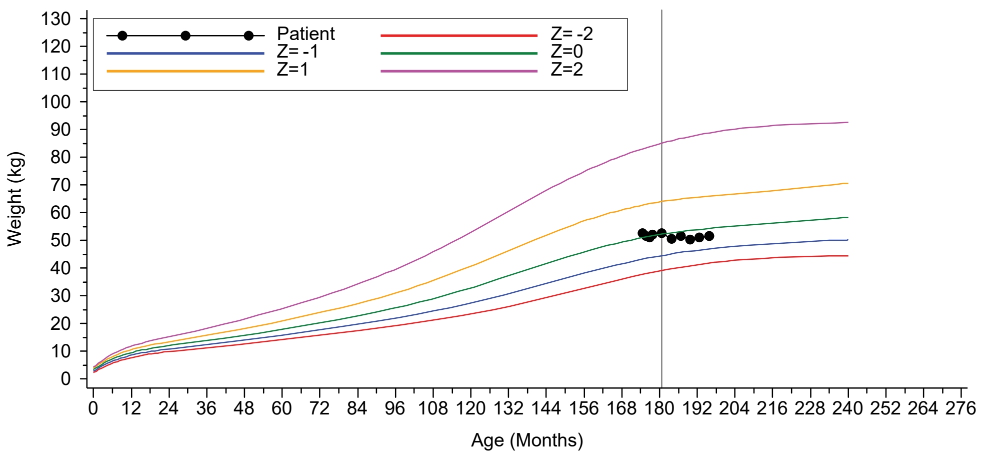

Patient 138

Seizure History: Partial Onset Seizures

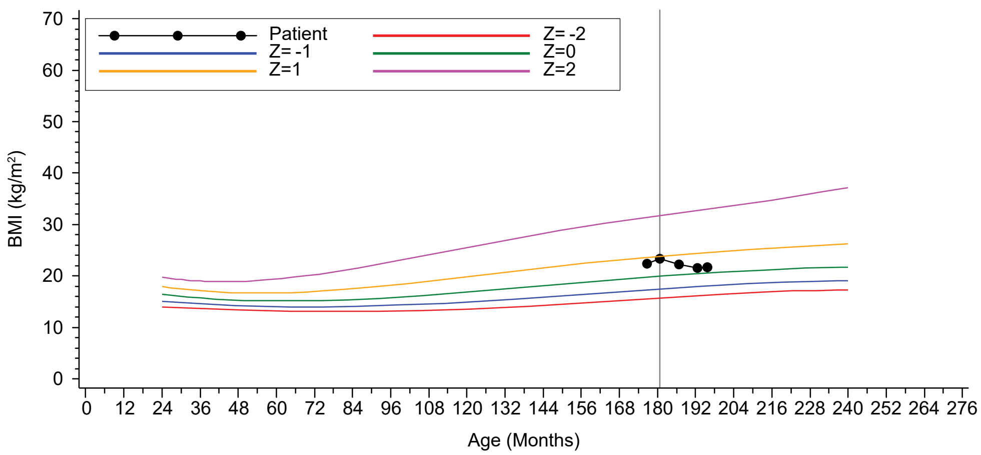

Patient 139

Seizure History: Partial Onset Seizures

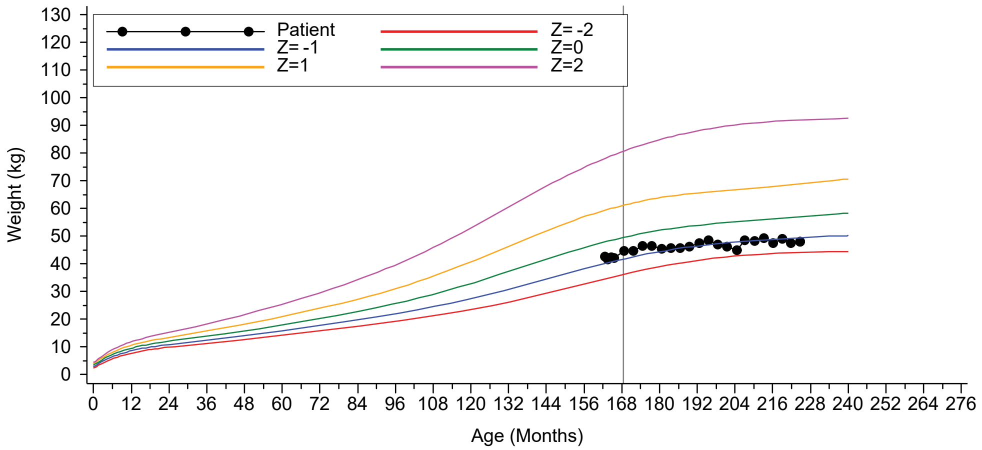

Patient 139

Seizure History: Partial Onset Seizures

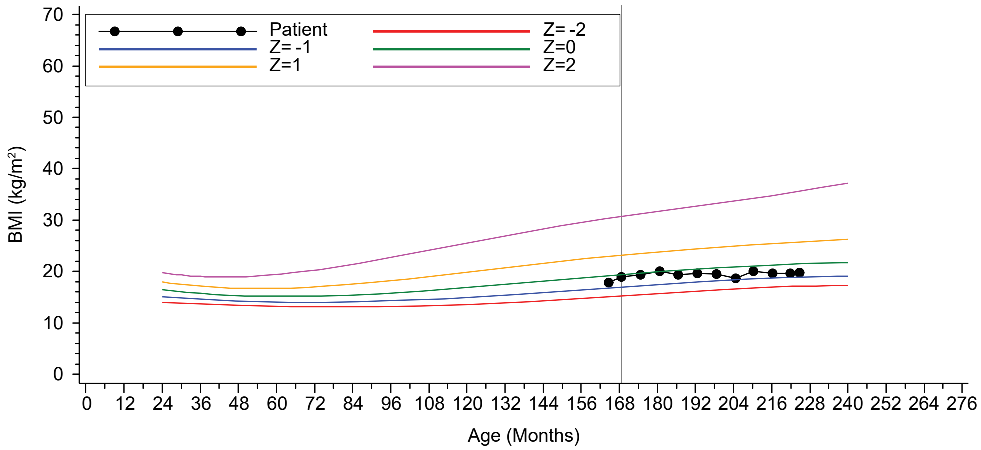

Patient 140

Seizure History: Partial Onset Seizures

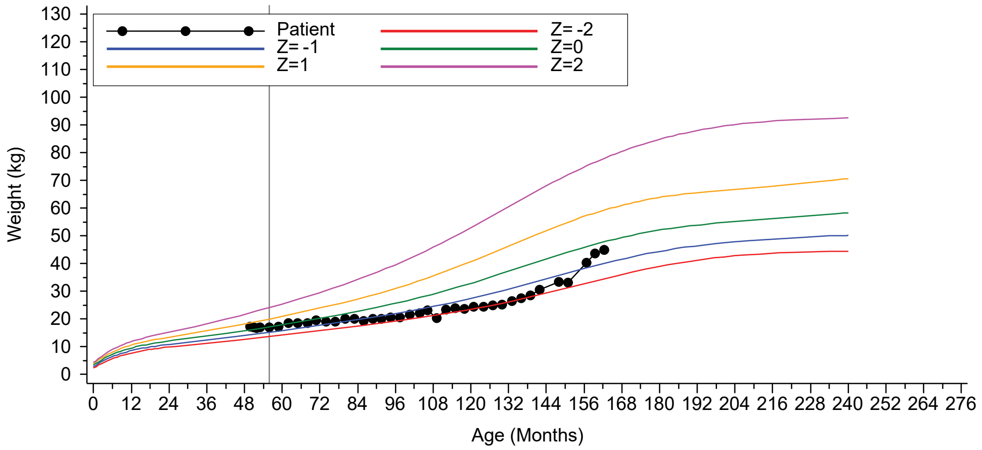

Patient 140

Seizure History: Partial Onset Seizures

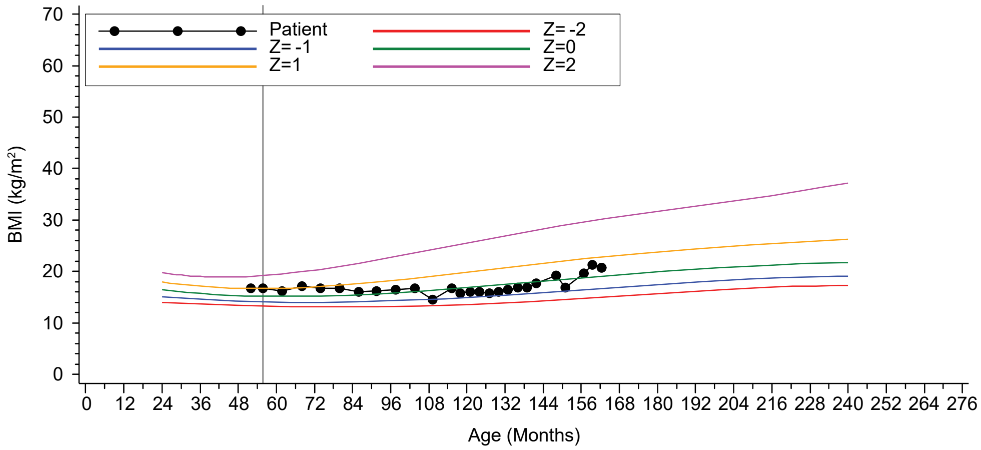

Patient 141

Seizure History: Partial Onset Seizures

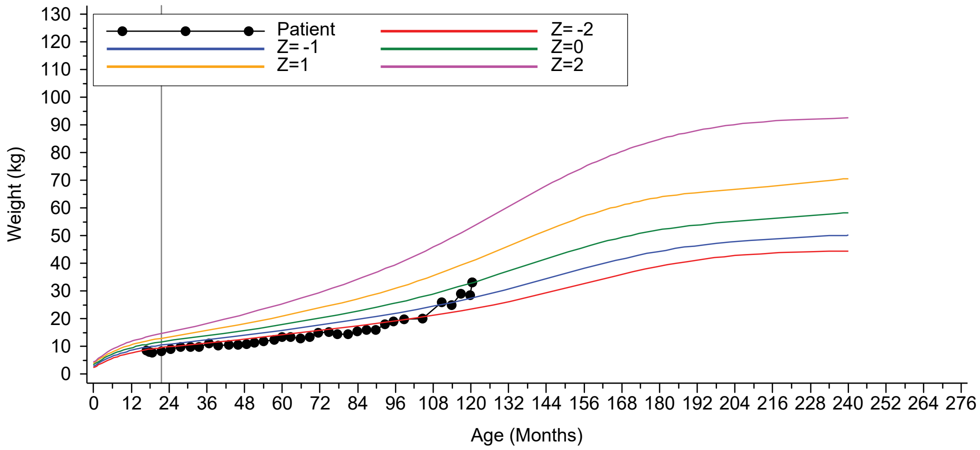

Patient 141

Seizure History: Partial Onset Seizures

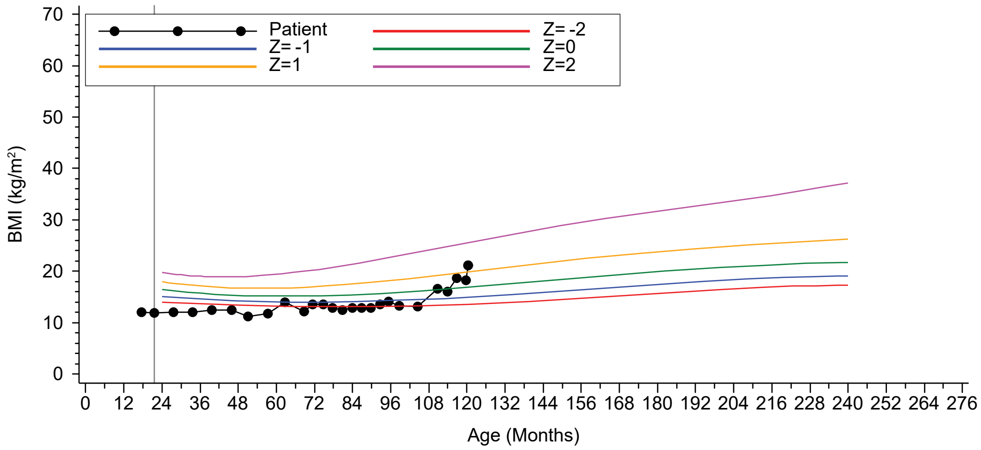

Patient 142

Seizure History: Partial Onset Seizures

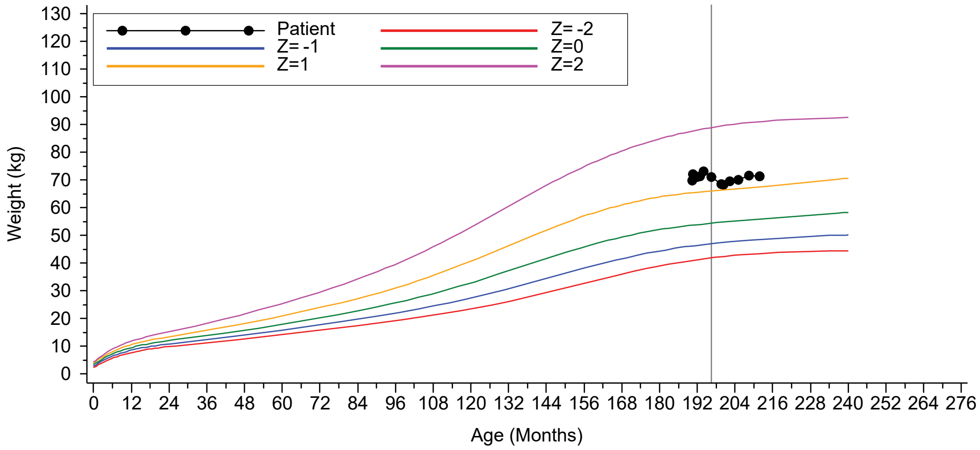

Patient 142

Seizure History: Partial Onset Seizures

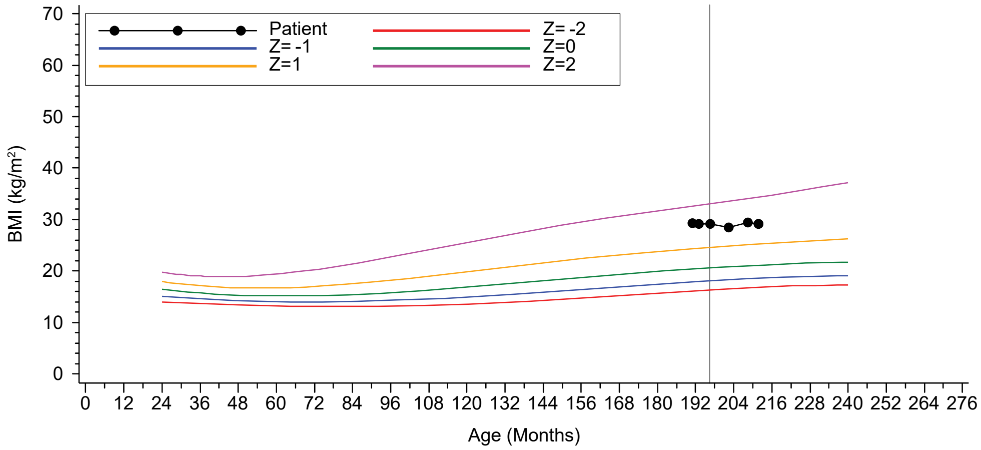

Patient 143

Seizure History: Partial Onset Seizures

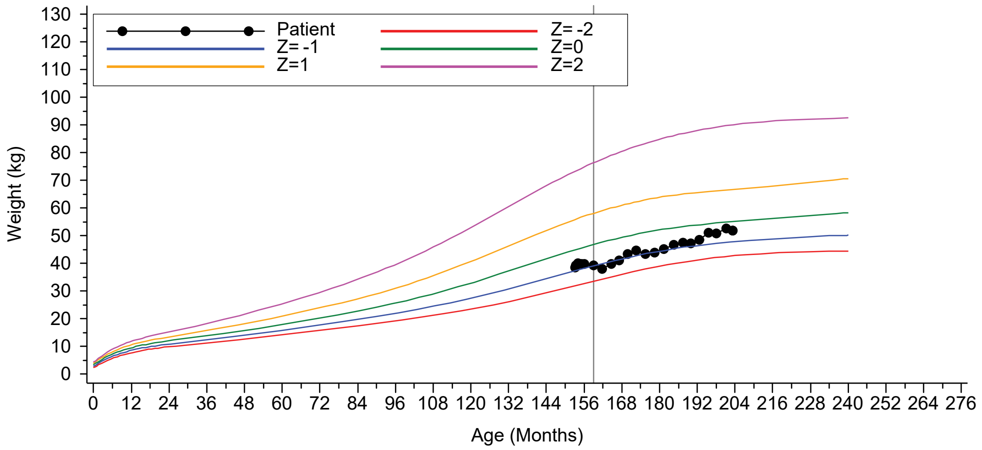

Patient 143

Seizure History: Partial Onset Seizures

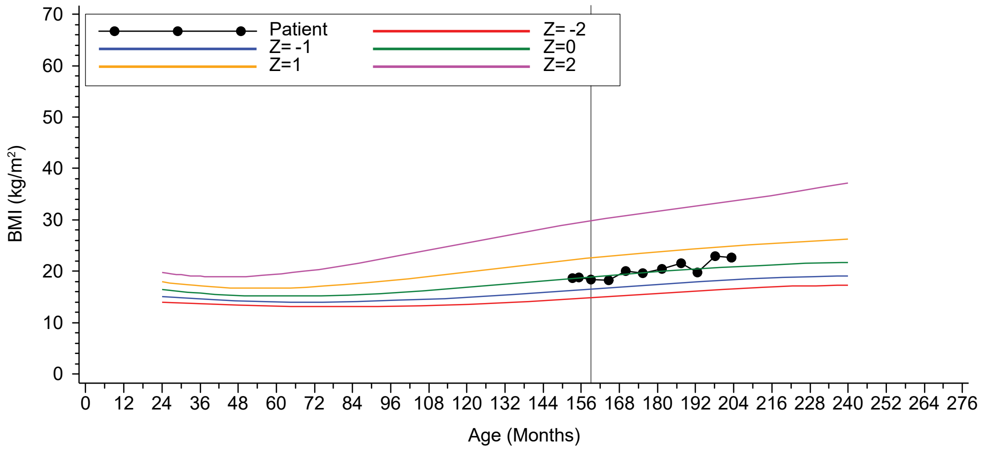

Patient 144

Seizure History: Partial Onset Seizures

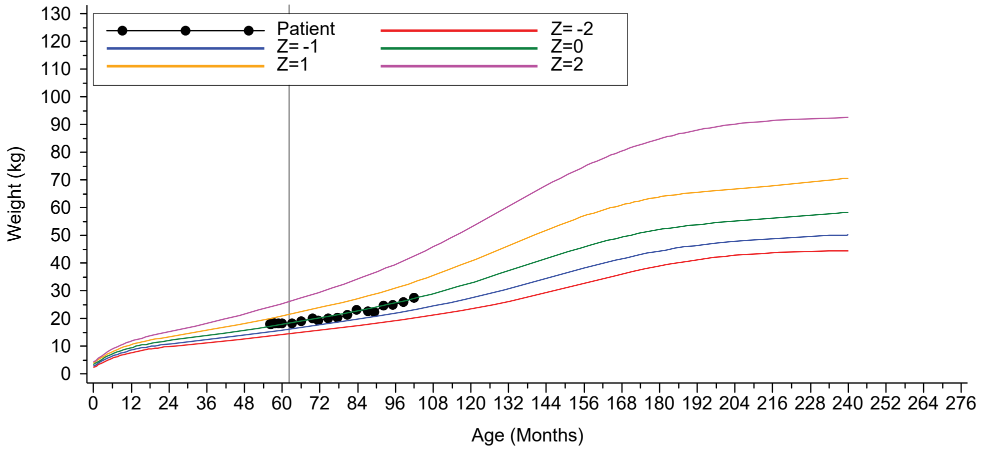

Patient 144

Seizure History: Partial Onset Seizures

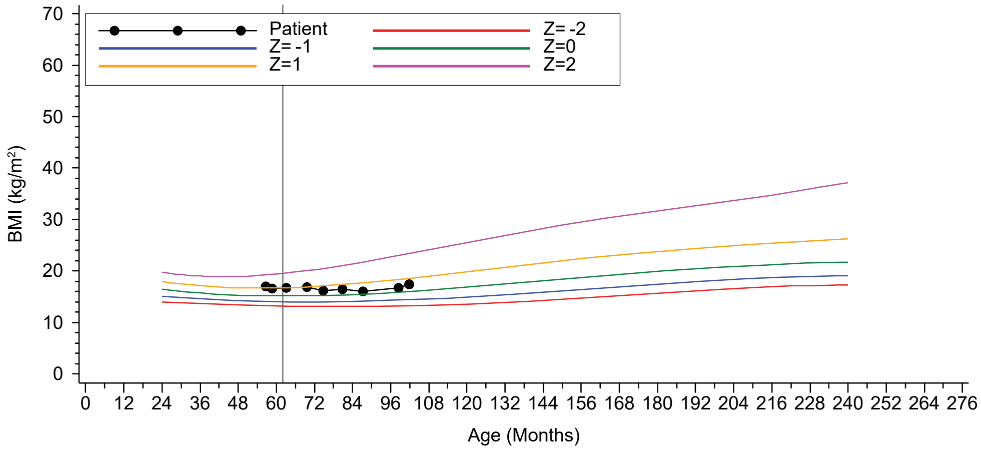

Patient 145

Seizure History: Partial Onset Seizures

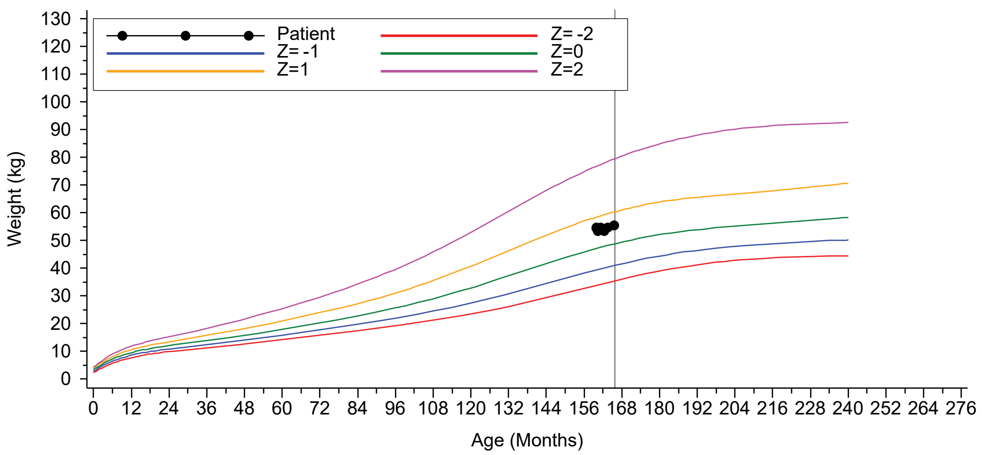

Patient 145

Seizure History: Partial Onset Seizures

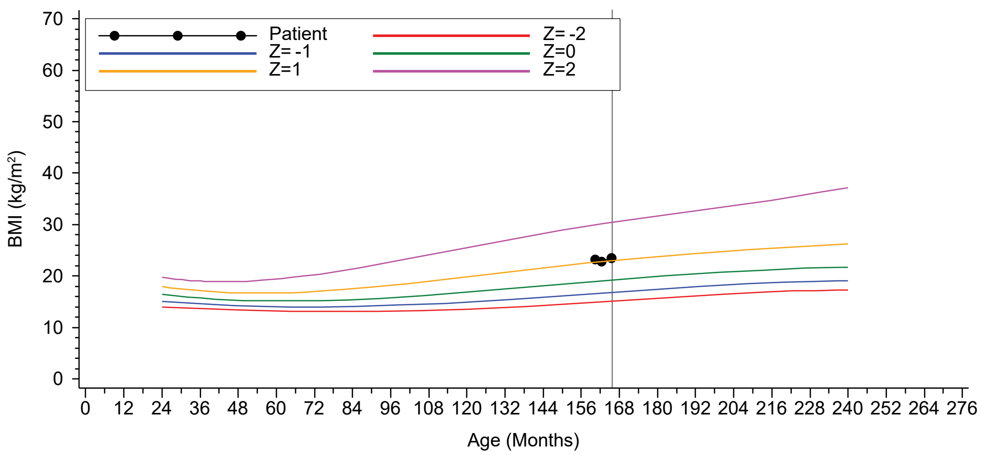

Patient 146

Seizure History: Partial Onset Seizures

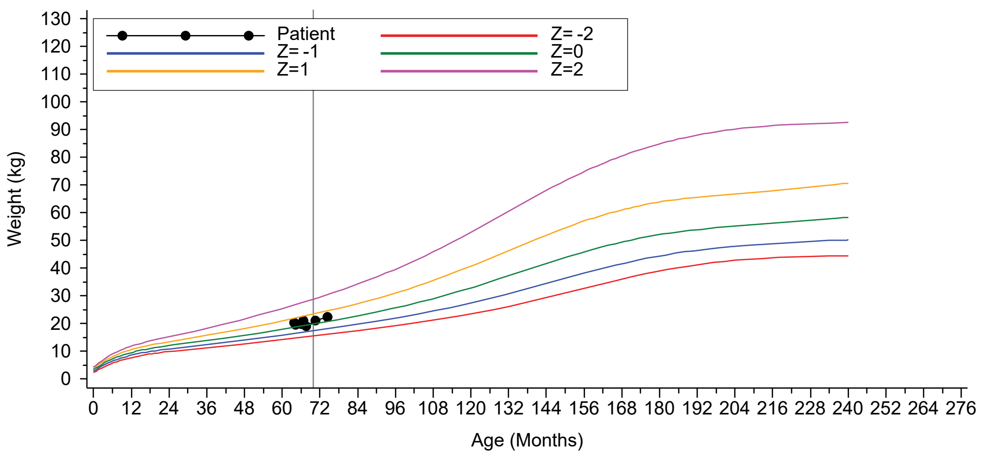

Patient 146

Seizure History: Partial Onset Seizures

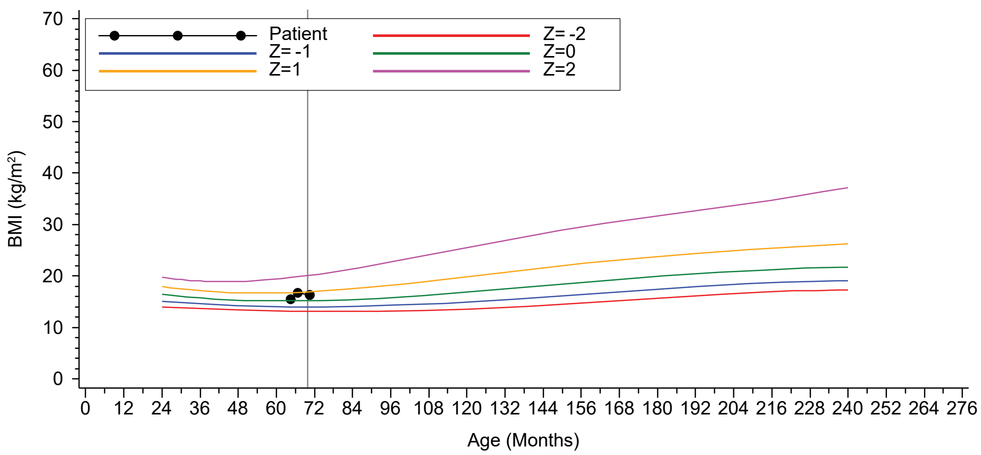

Patient 147

Seizure History: Partial Onset Seizures

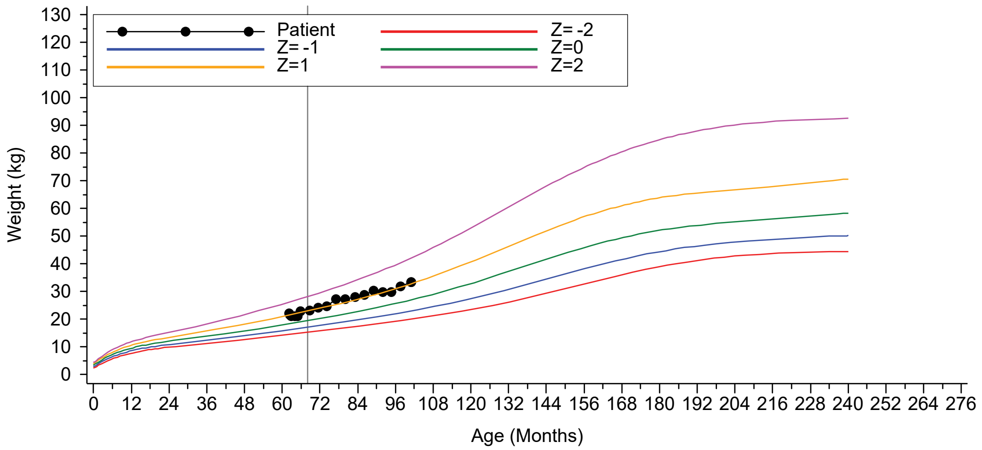

Patient 147

Seizure History: Partial Onset Seizures

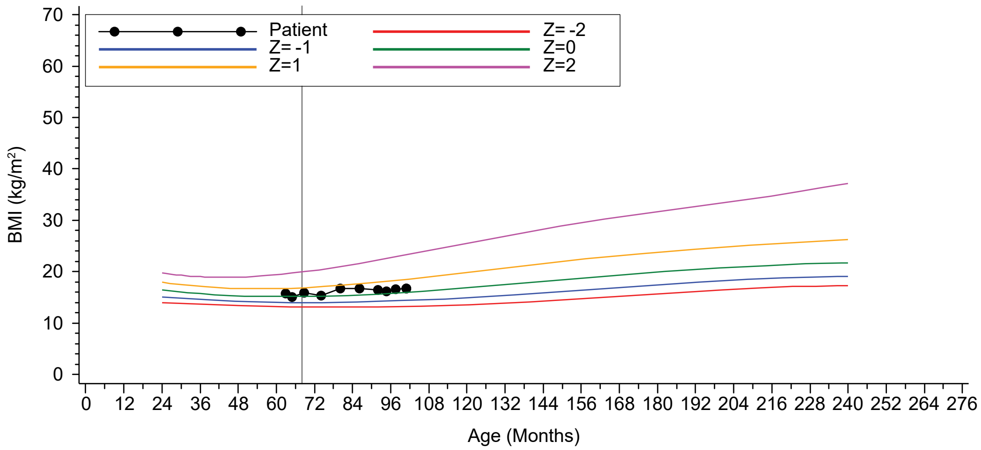

Patient 148

Seizure History: Partial Onset Seizures

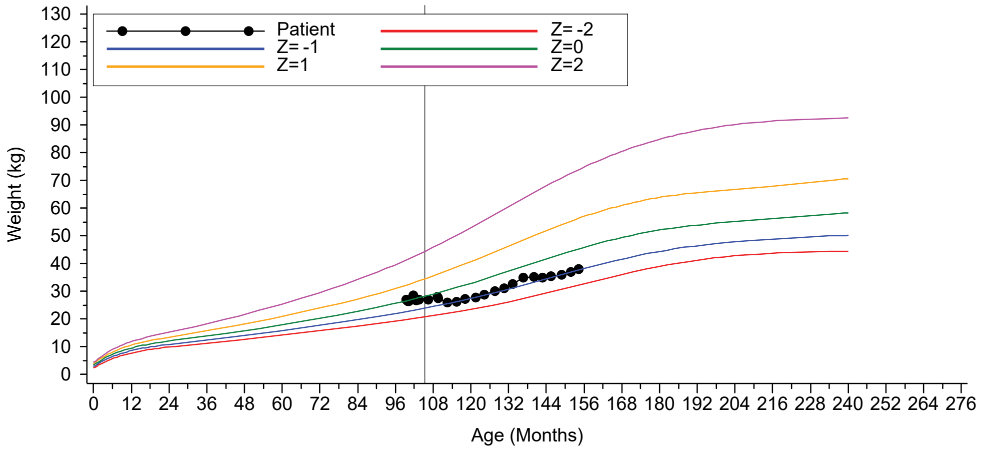

Patient 148

Seizure History: Partial Onset Seizures

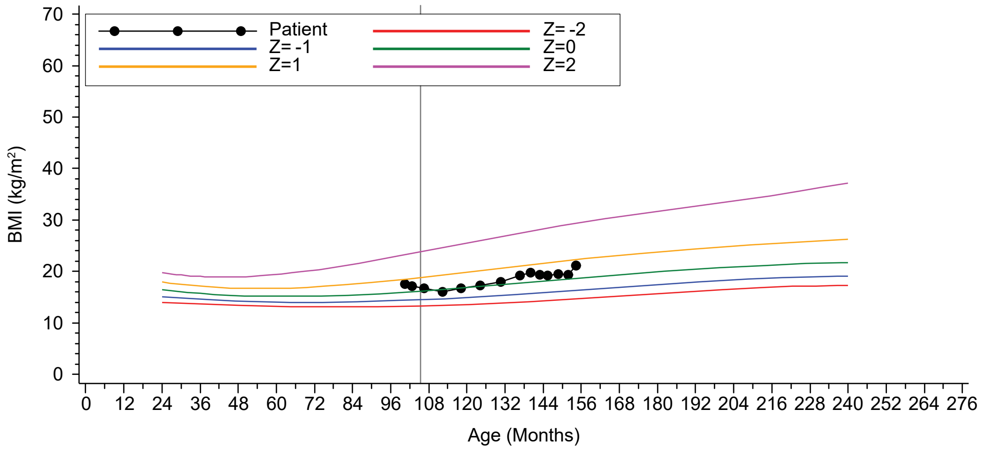

Patient 149

Seizure History: Partial Onset Seizures

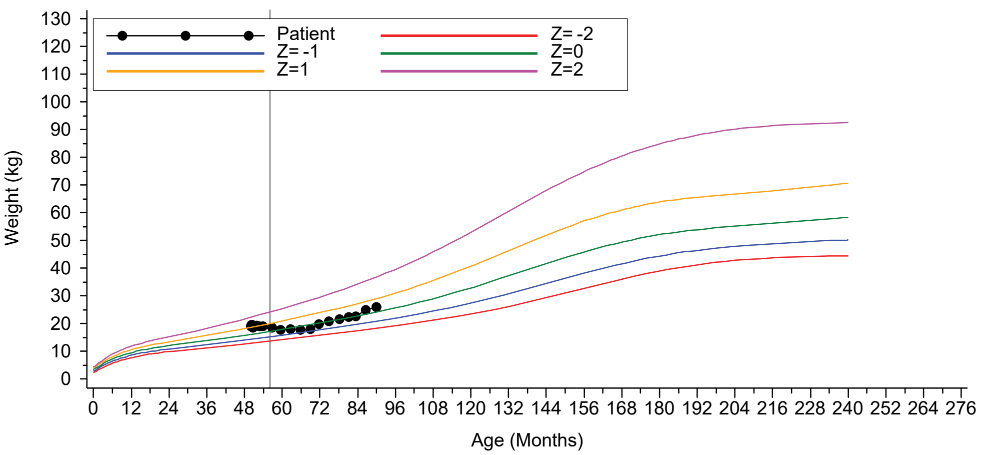

Patient 149

Seizure History: Partial Onset Seizures

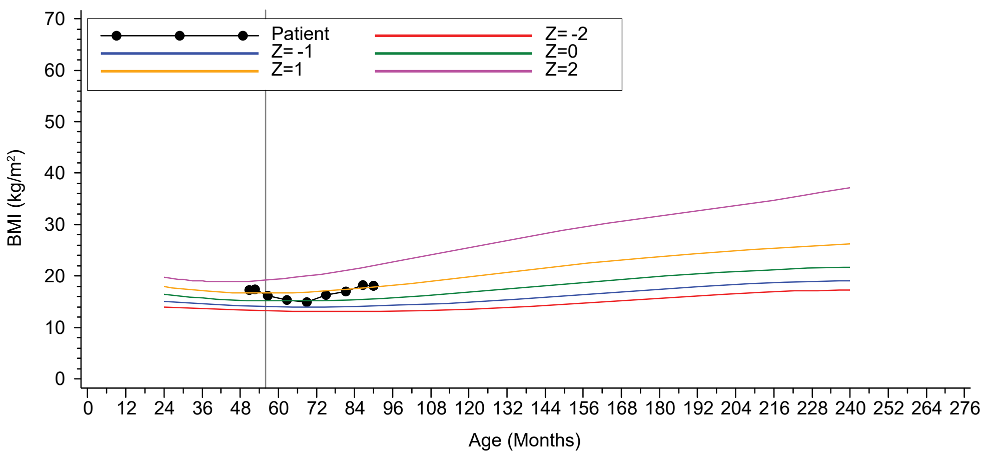

Patient 150

Seizure History: Partial Onset Seizures

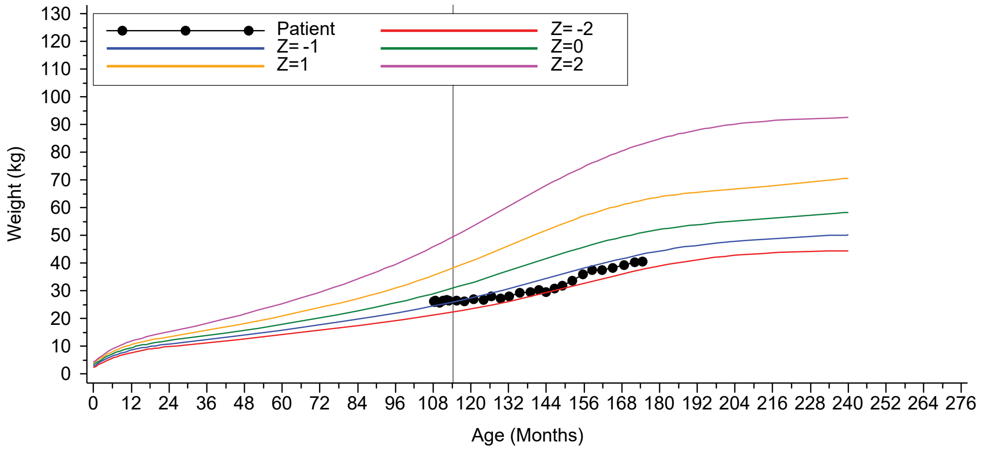

Patient 150

Seizure History: Partial Onset Seizures

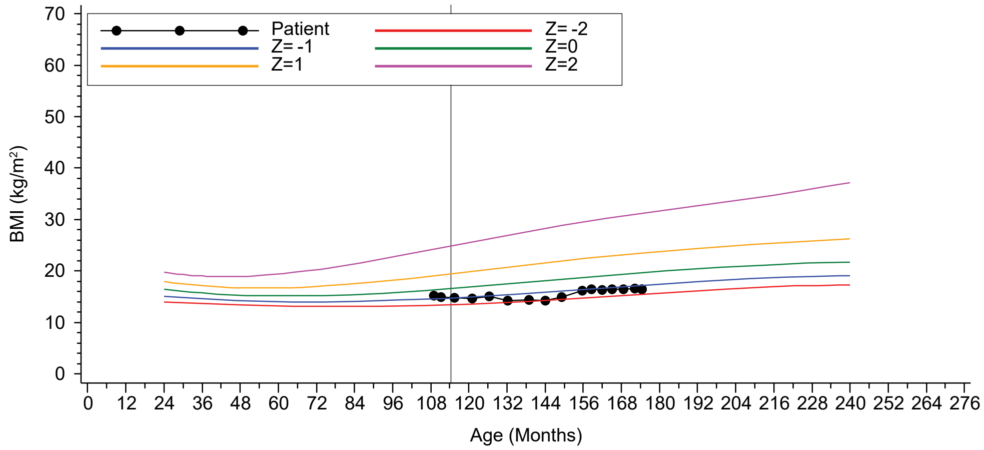

Patient 151

Seizure History: Partial Onset Seizures

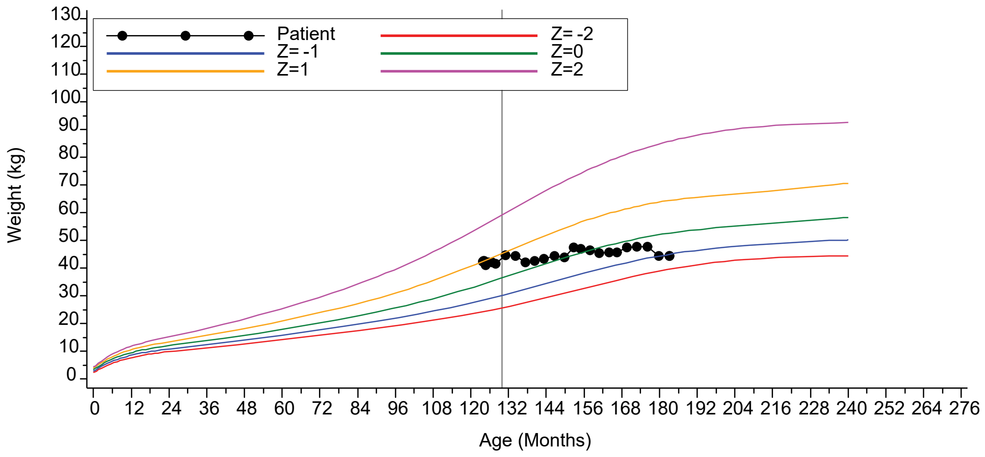

Patient 151

Seizure History: Partial Onset Seizures

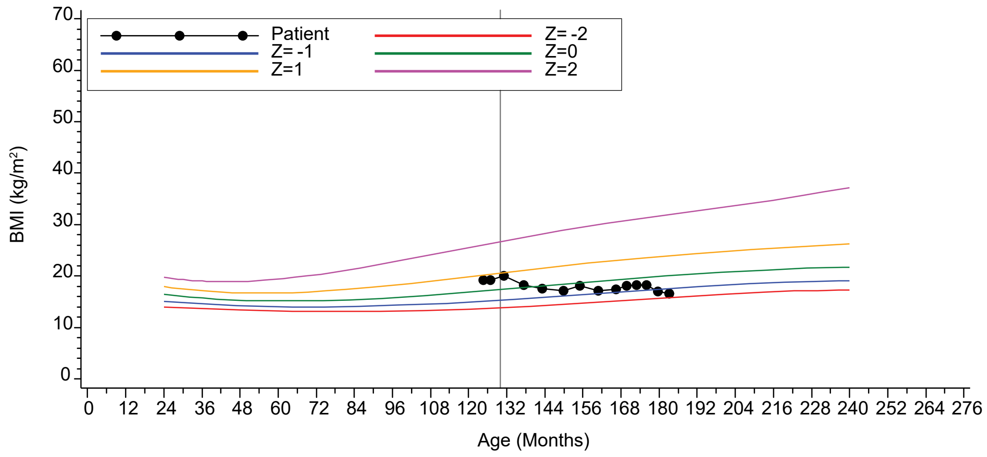

Patient 152

Seizure History: Partial Onset Seizures

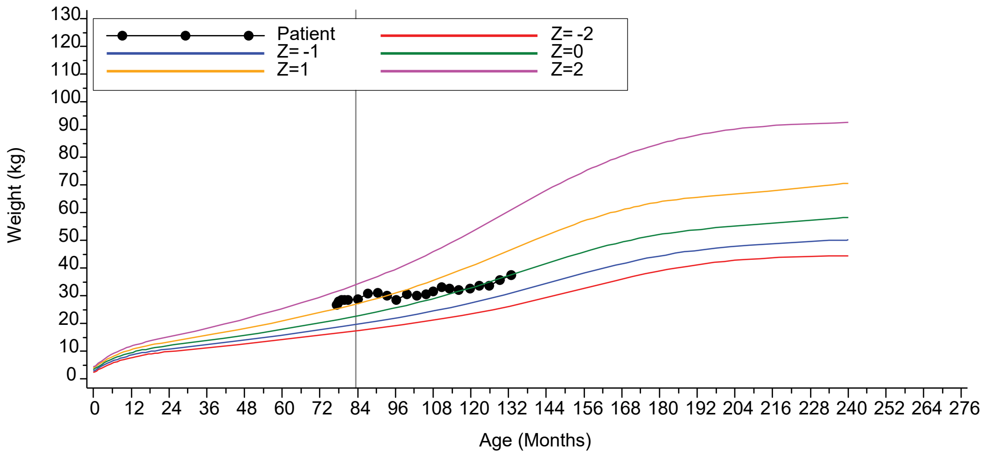

Patient 152

Seizure History: Partial Onset Seizures

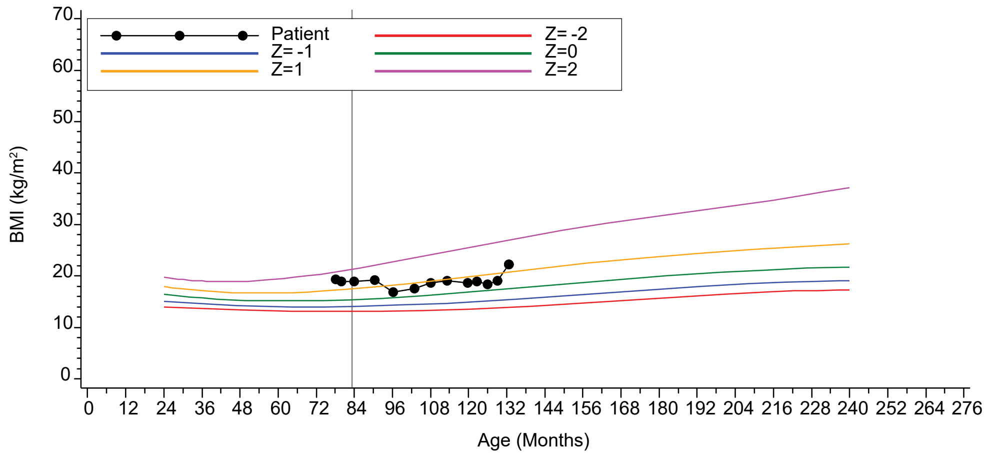

Patient 153

Seizure History: Partial Onset Seizures

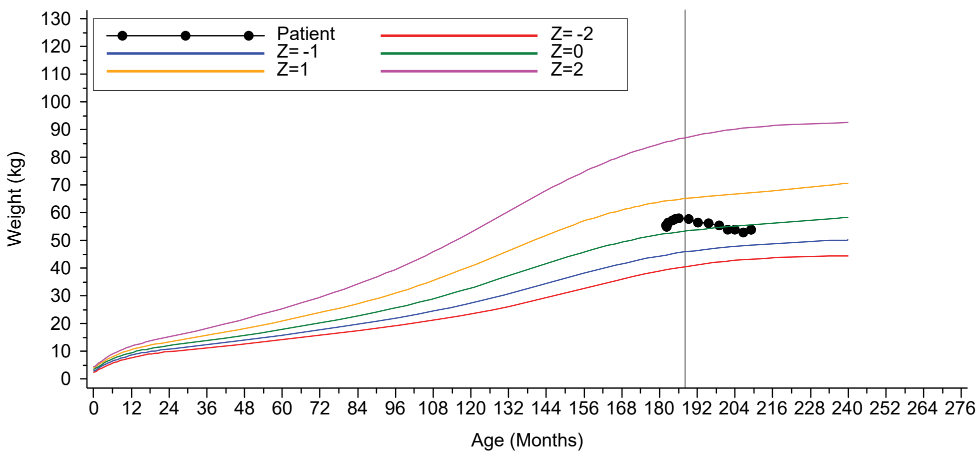

Patient 153

Seizure History: Partial Onset Seizures

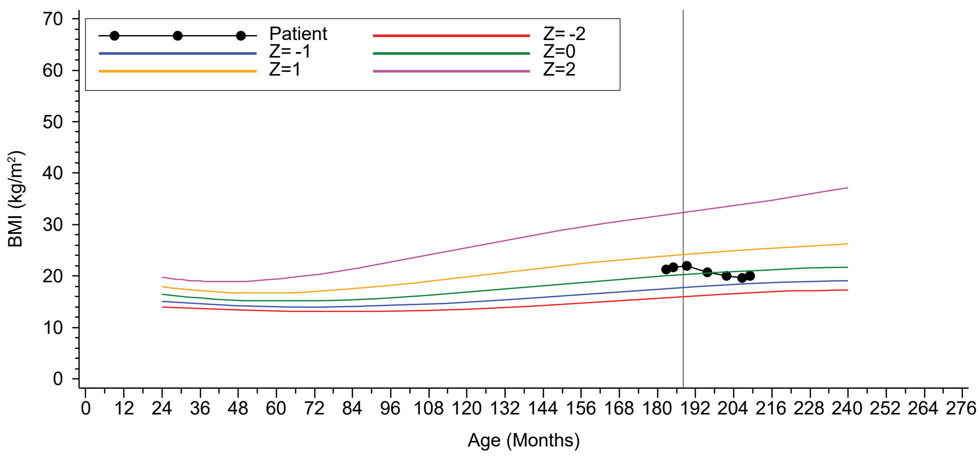

Patient 154

Seizure History: Partial Onset Seizures

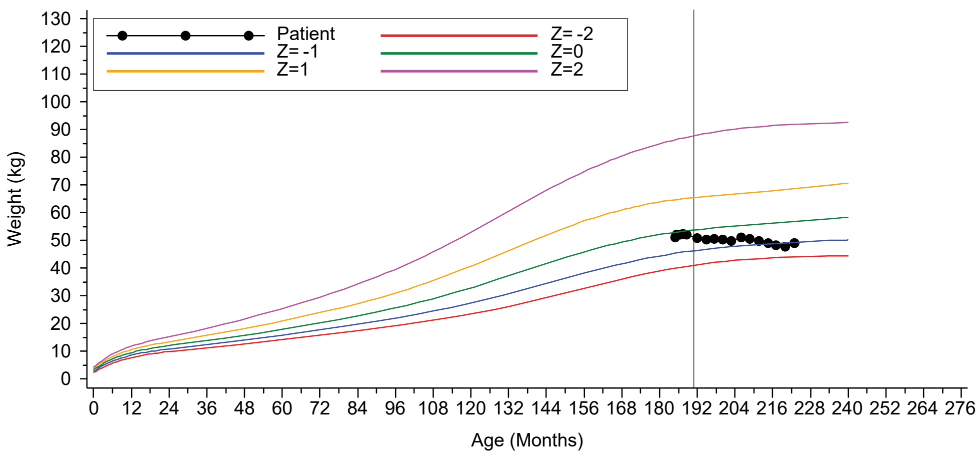

Patient 154

Seizure History: Partial Onset Seizures

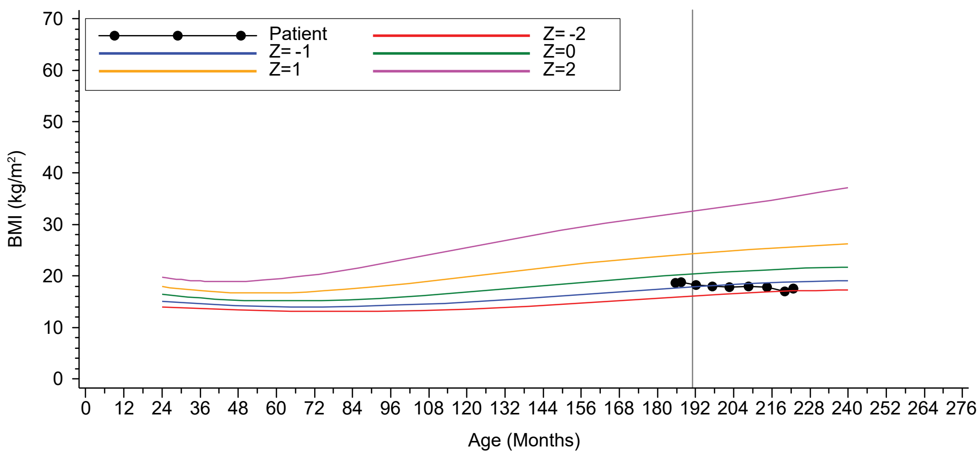

Patient 155
Seizure History: Partial Onset Seizures

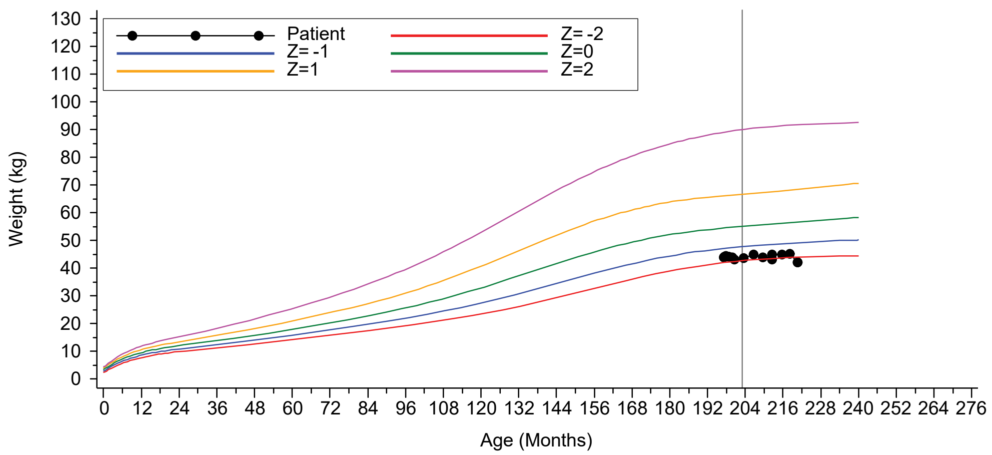

Patient 155
Seizure History: Partial Onset Seizures

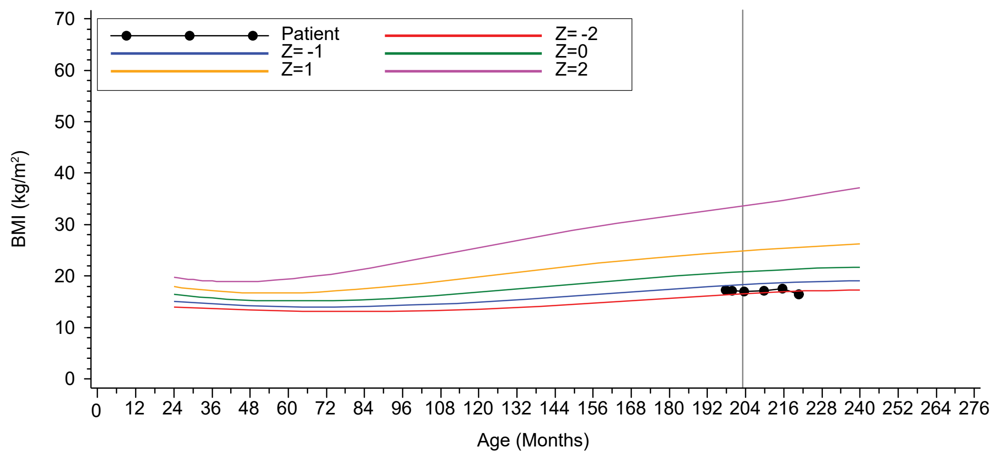

Patient 156
Seizure History: Partial Onset Seizures

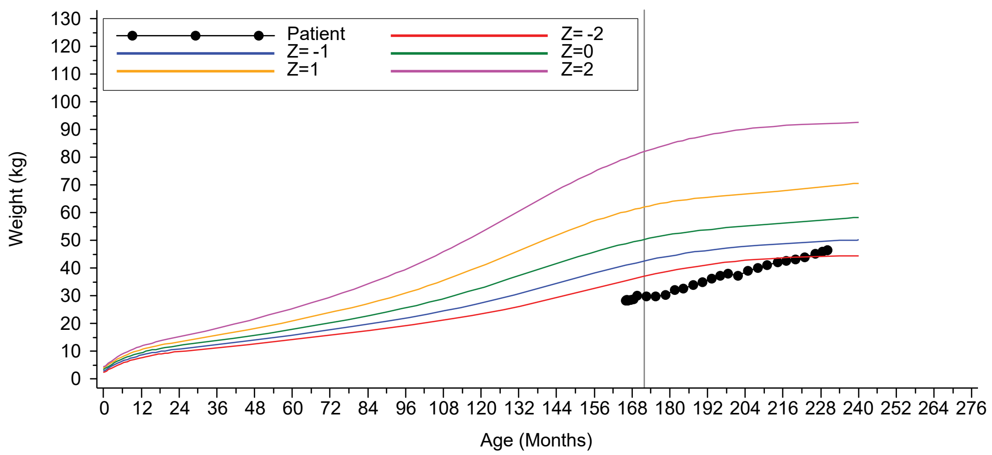

Patient 156
Seizure History: Partial Onset Seizures

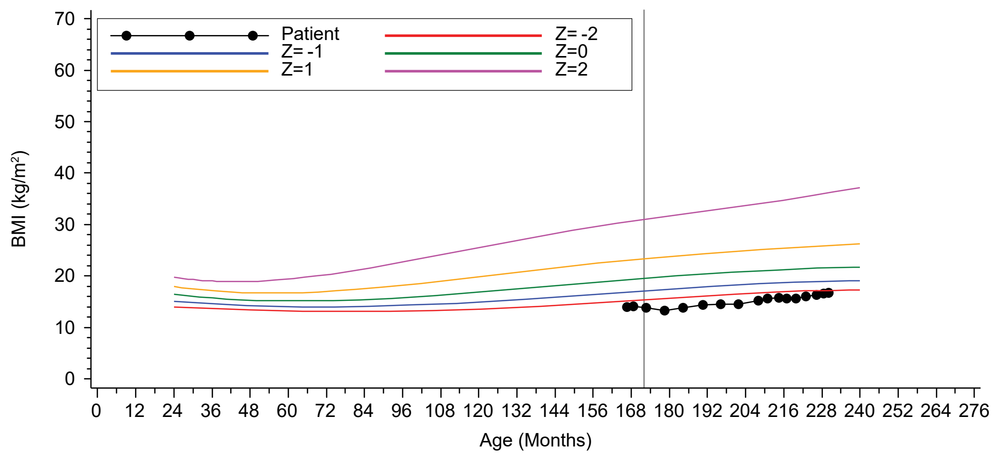

Patient 157

Seizure History: Partial Onset Seizures

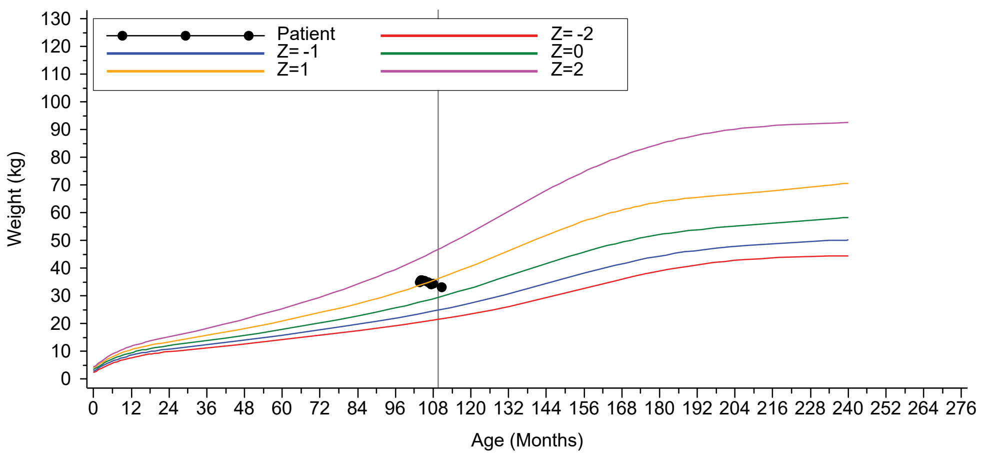

Patient 157

Seizure History: Partial Onset Seizures

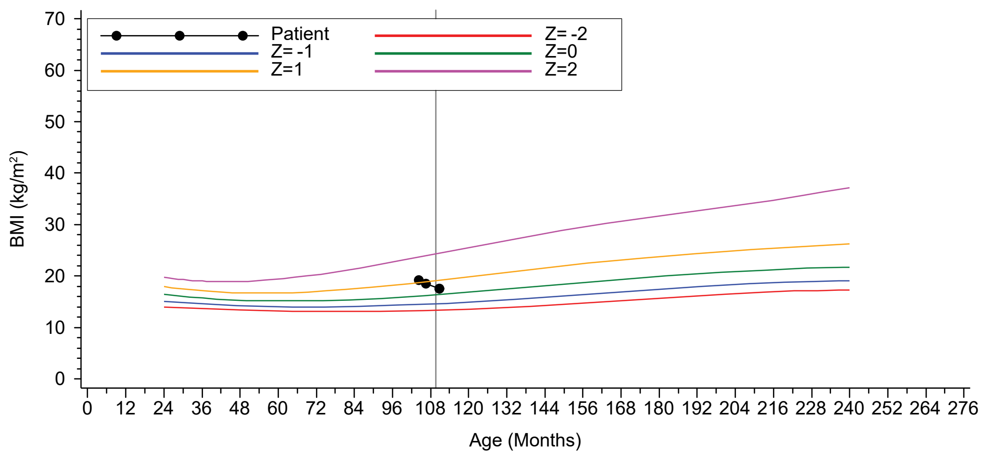

Patient 158

Seizure History: Partial Onset Seizures

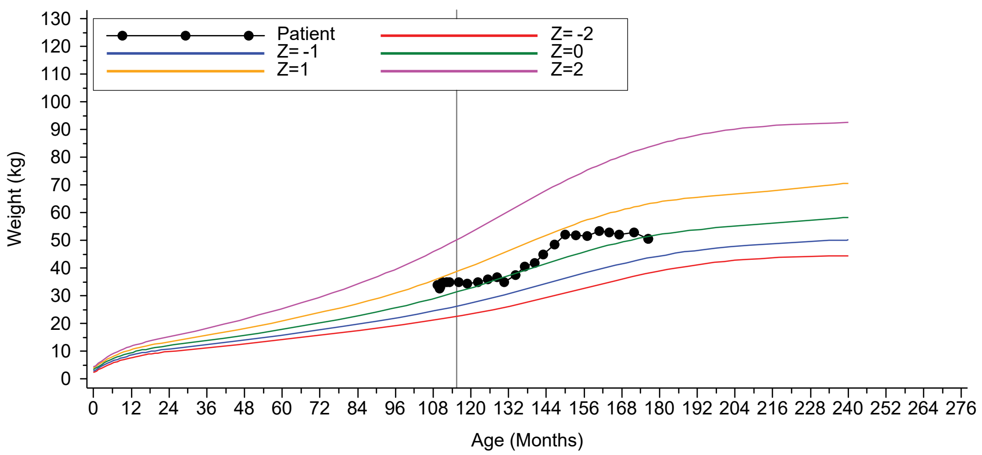

Patient 158

Seizure History: Partial Onset Seizures

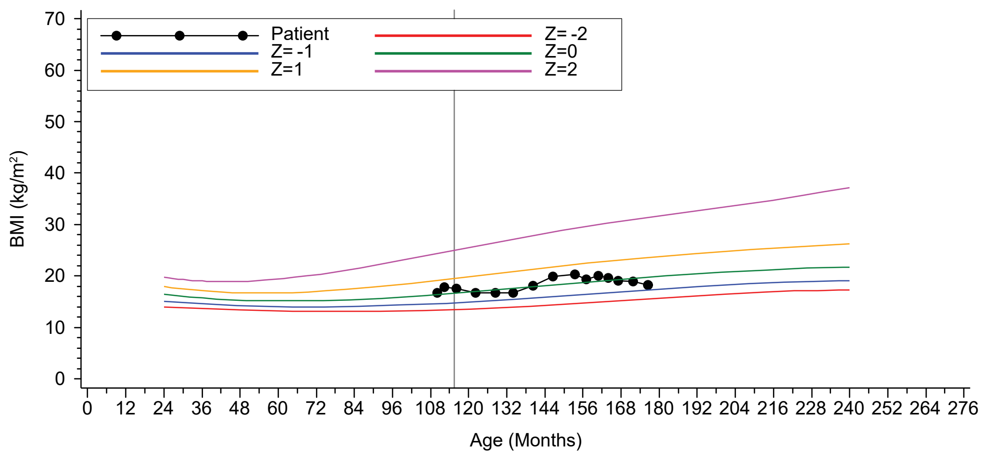

Patient 159

Seizure History: Partial Onset Seizures

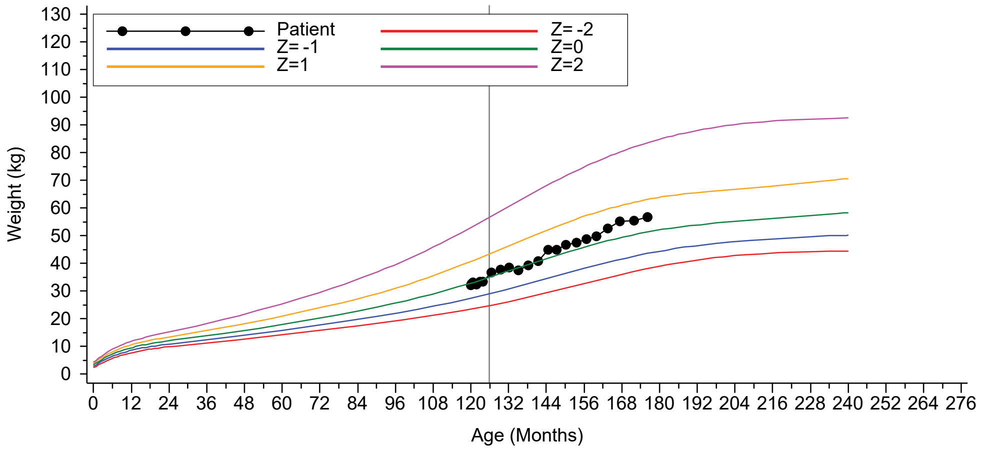

Patient 159

Seizure History: Partial Onset Seizures

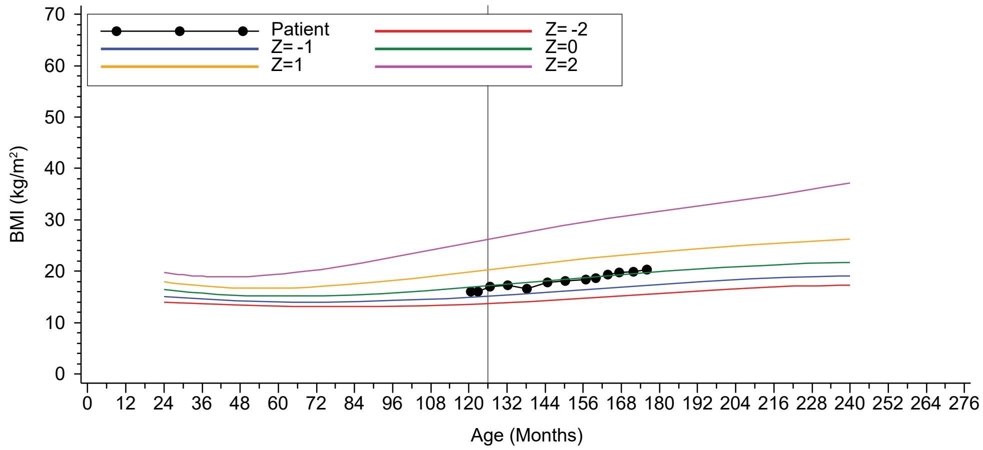

Patient 160

Seizure History: Partial Onset Seizures

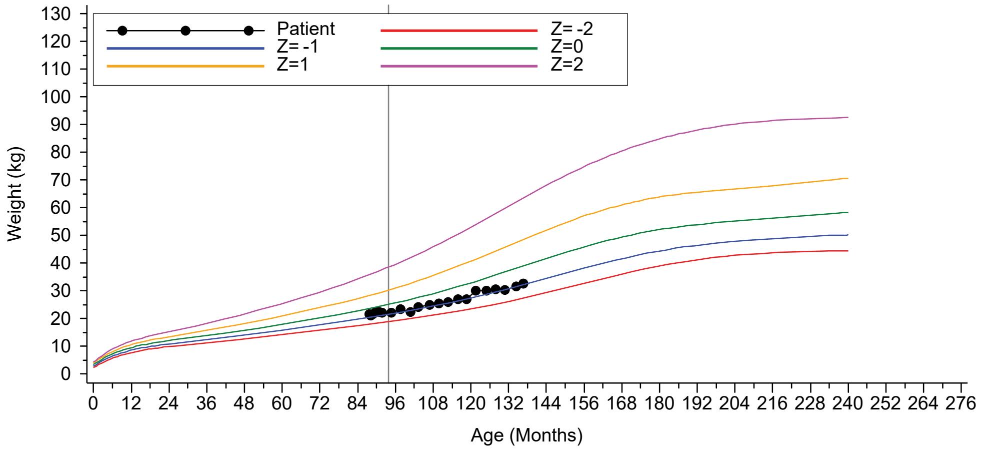

Patient 160

Seizure History: Partial Onset Seizures

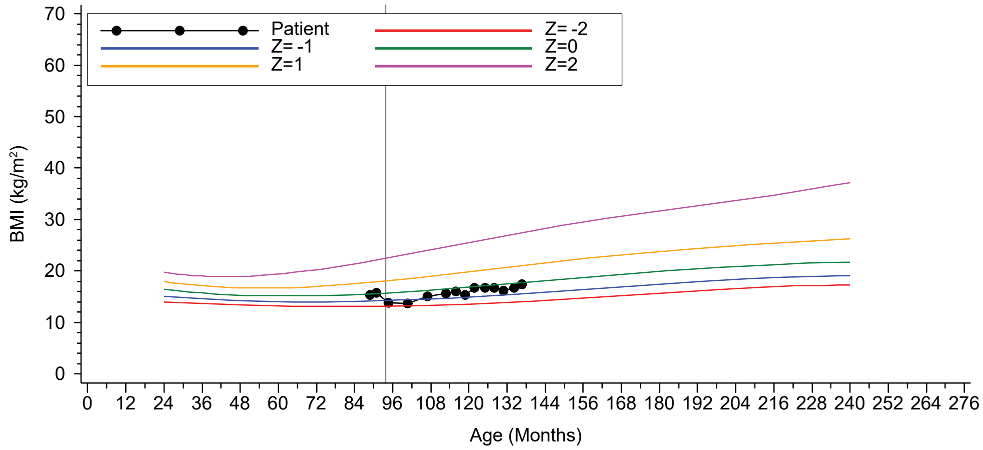

Patient 161

Seizure History: Partial Onset Seizures

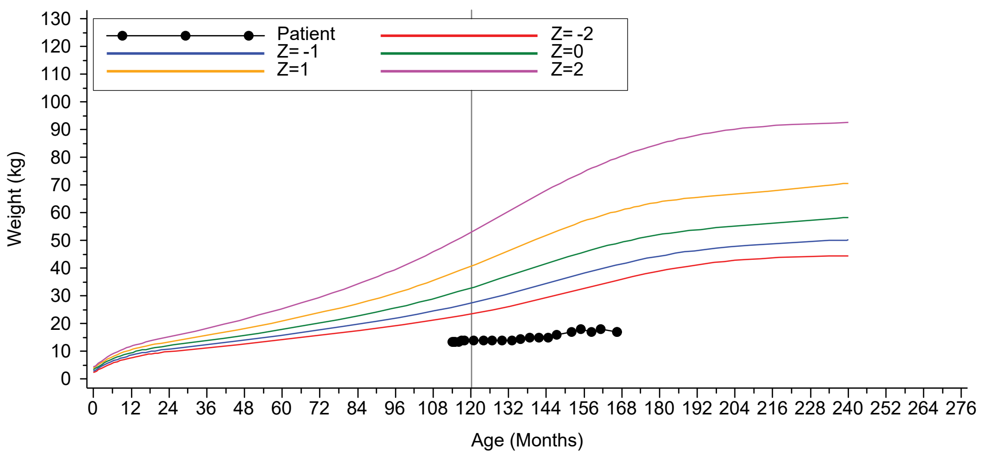

Patient 161

Seizure History: Partial Onset Seizures

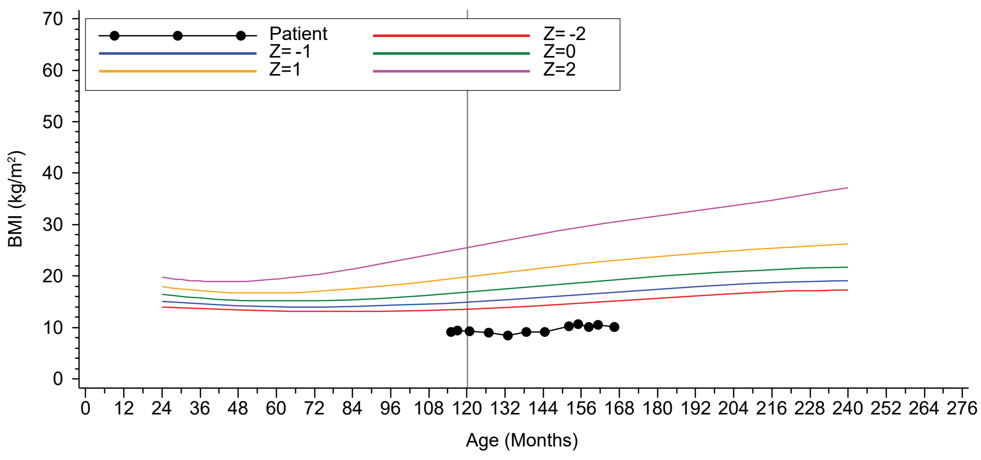

Patient 162

Seizure History: Partial Onset Seizures

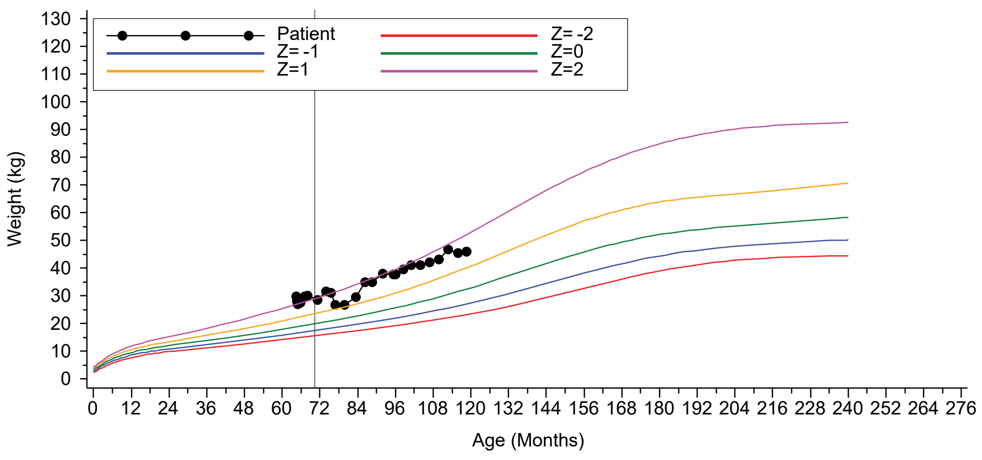

Patient 162

Seizure History: Partial Onset Seizures

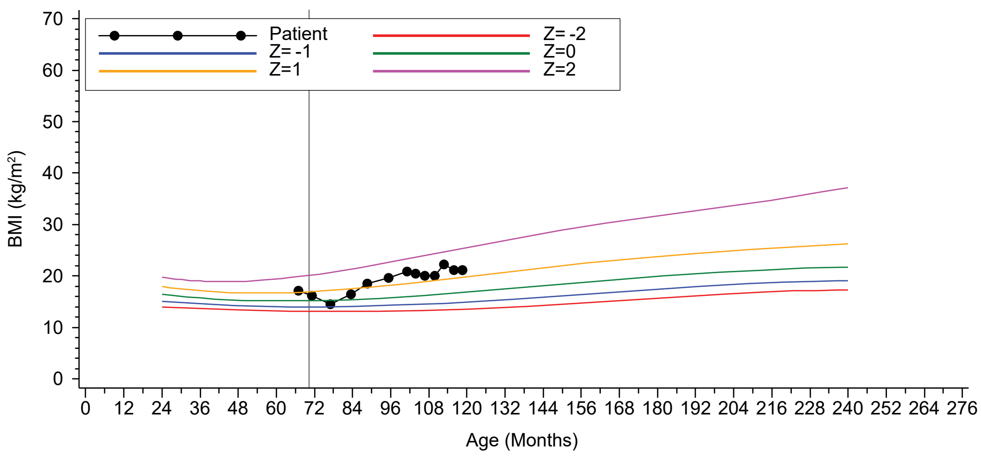

Patient 163

Seizure History: Partial Onset Seizures

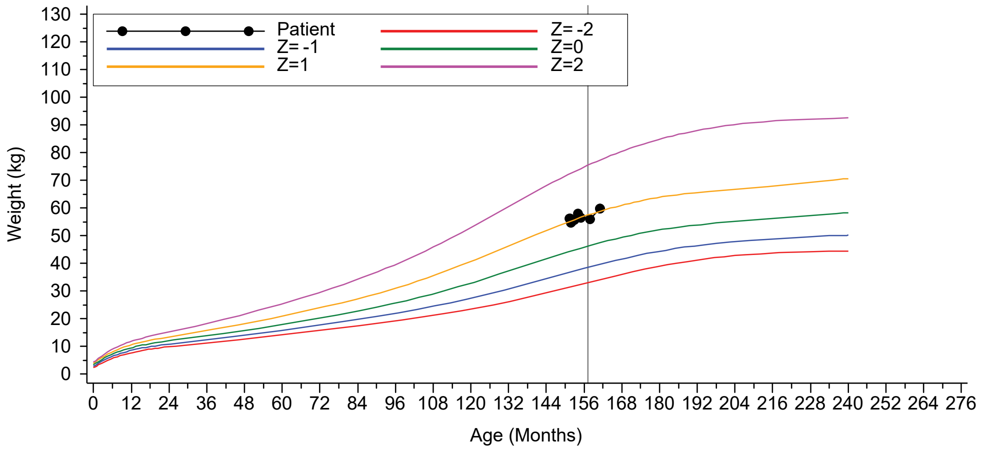

Patient 163

Seizure History: Partial Onset Seizures

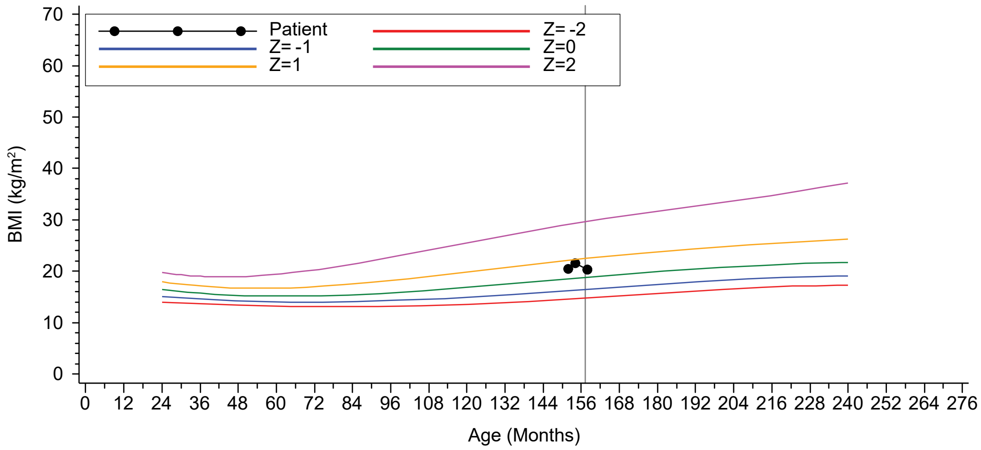

Patient 164

Seizure History: Partial Onset Seizures

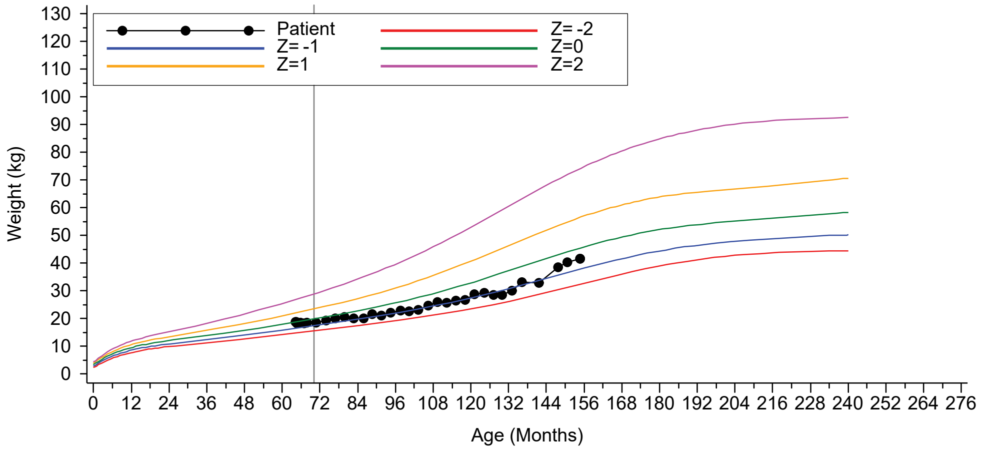

Patient 164

Seizure History: Partial Onset Seizures

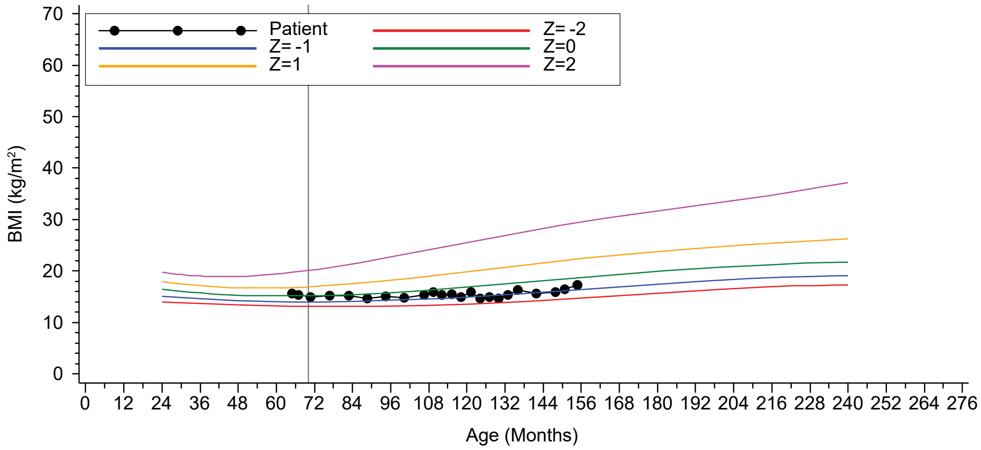

Patient 165

Seizure History: Partial Onset Seizures

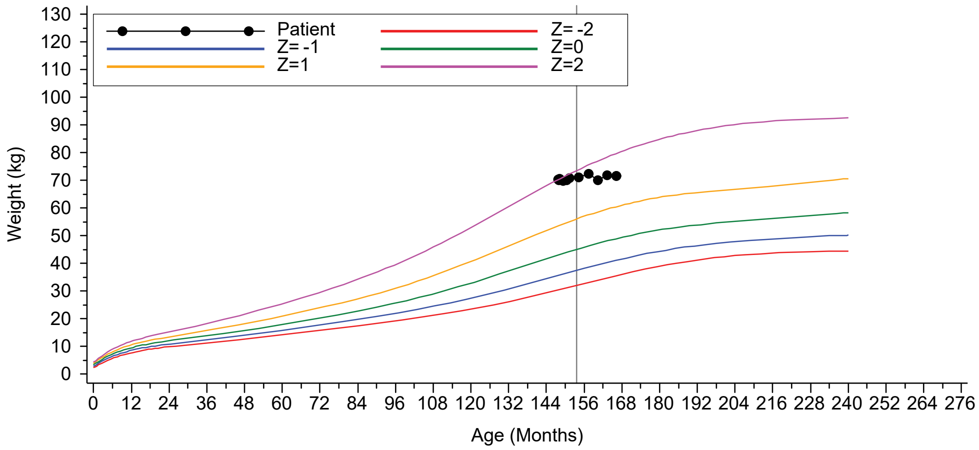

Patient 165

Seizure History: Partial Onset Seizures

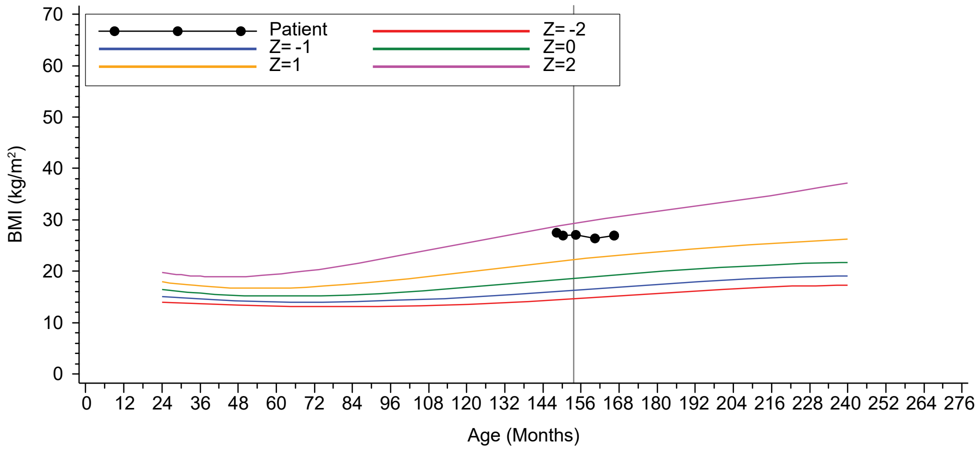

Patient 166

Seizure History: Partial Onset Seizures

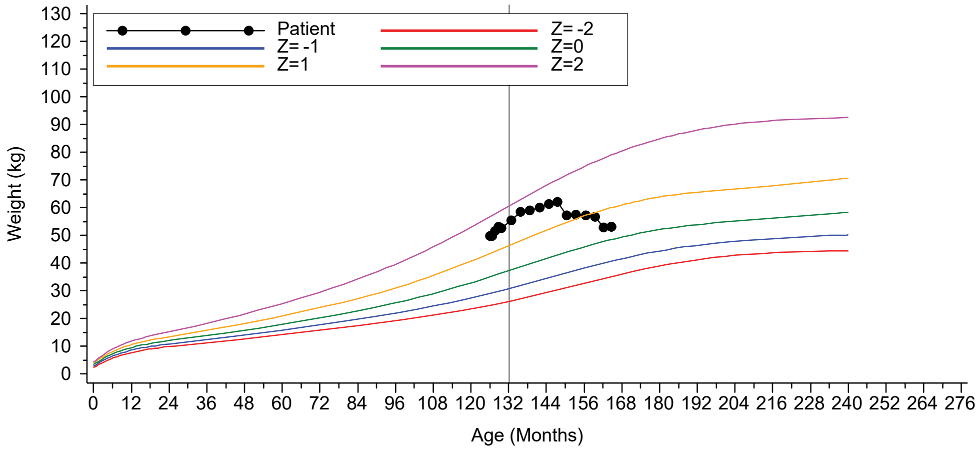

Patient 166

Seizure History: Partial Onset Seizures

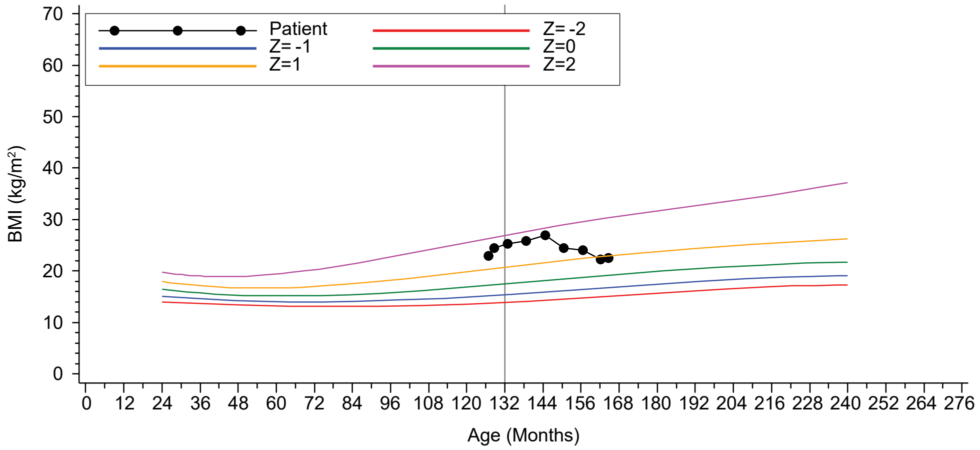

Patient 167

Seizure History: Partial Onset Seizures

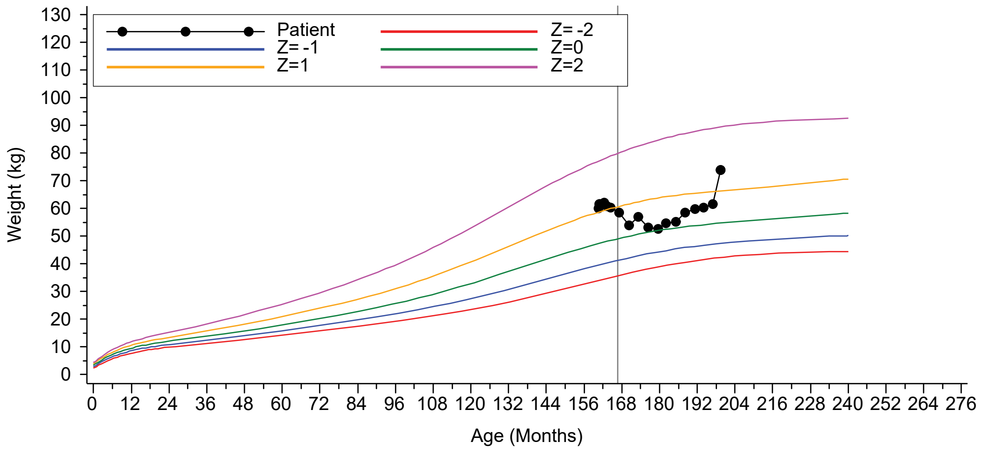

Patient 167

Seizure History: Partial Onset Seizures

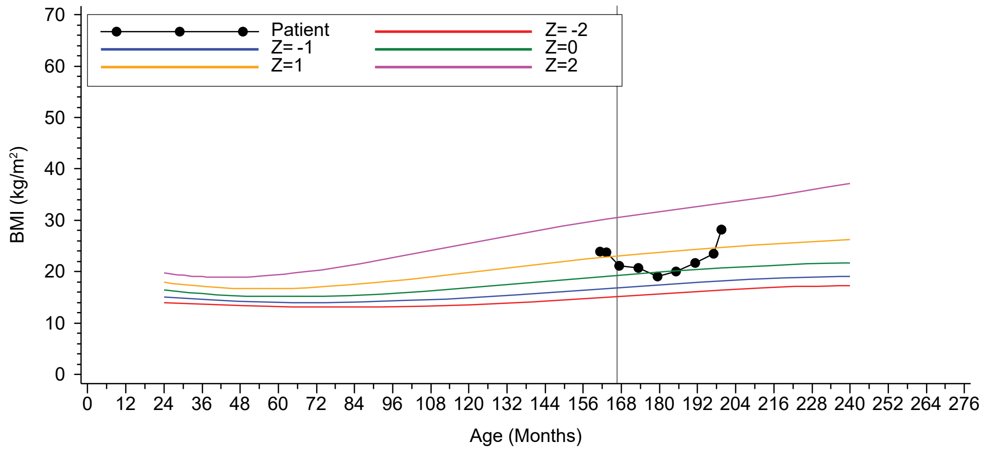

Patient 168

Seizure History: Partial Onset Seizures

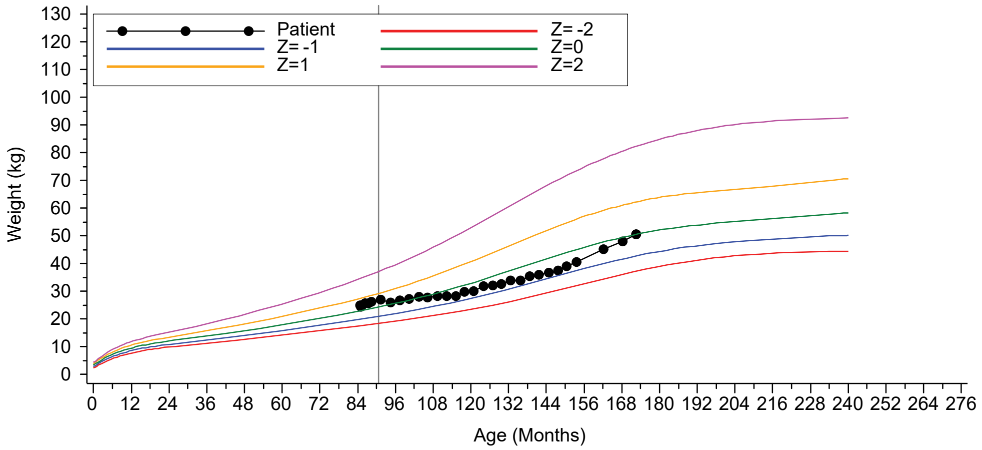

Patient 168

Seizure History: Partial Onset Seizures

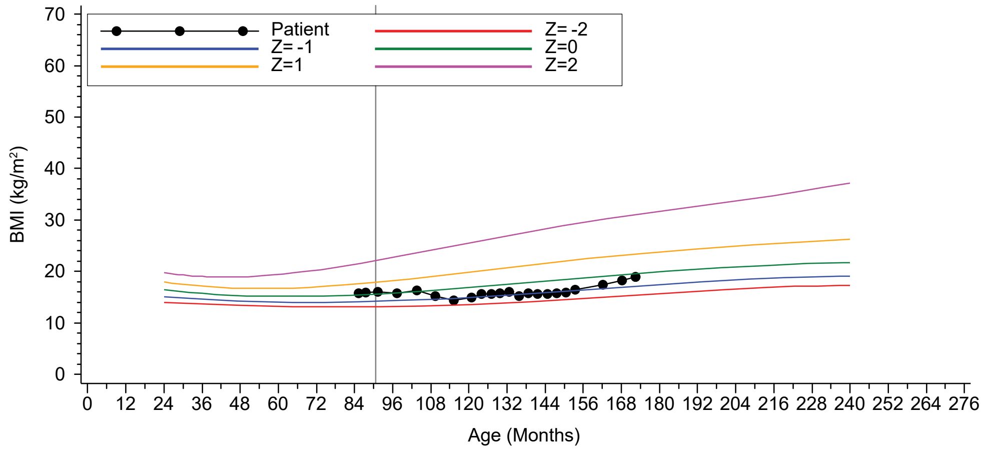

Patient 169

Seizure History: Partial Onset Seizures

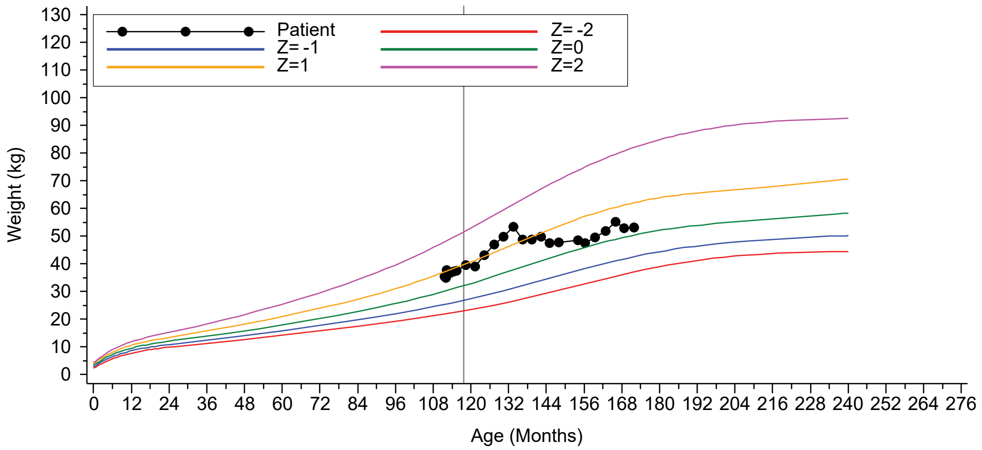

Patient 169

Seizure History: Partial Onset Seizures

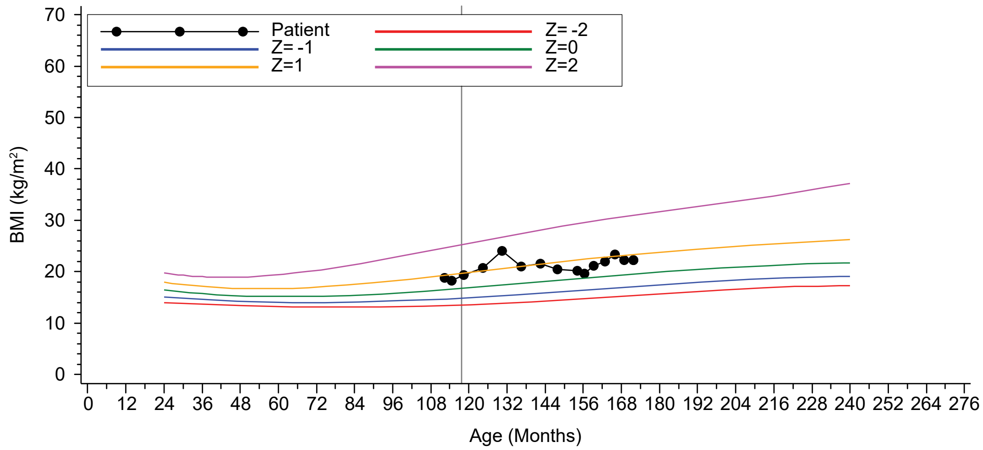

Patient 170

Seizure History: Partial Onset Seizures

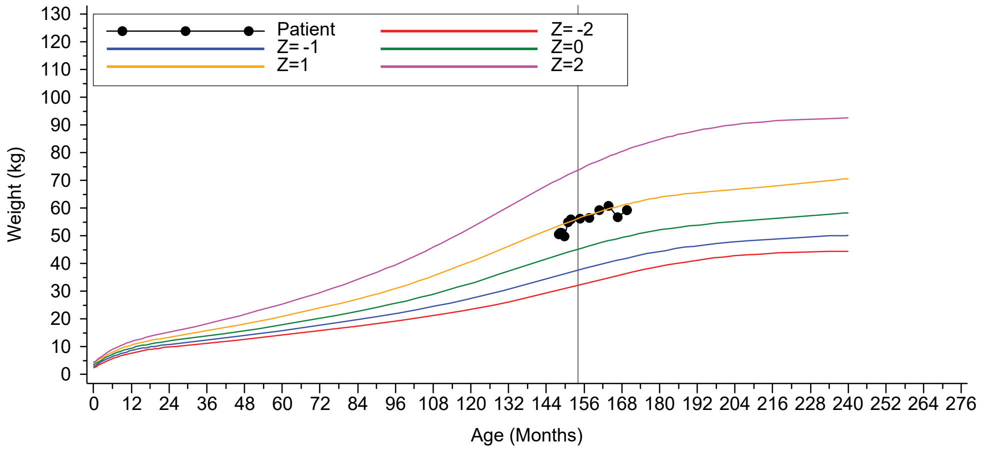

Patient 170

Seizure History: Partial Onset Seizures

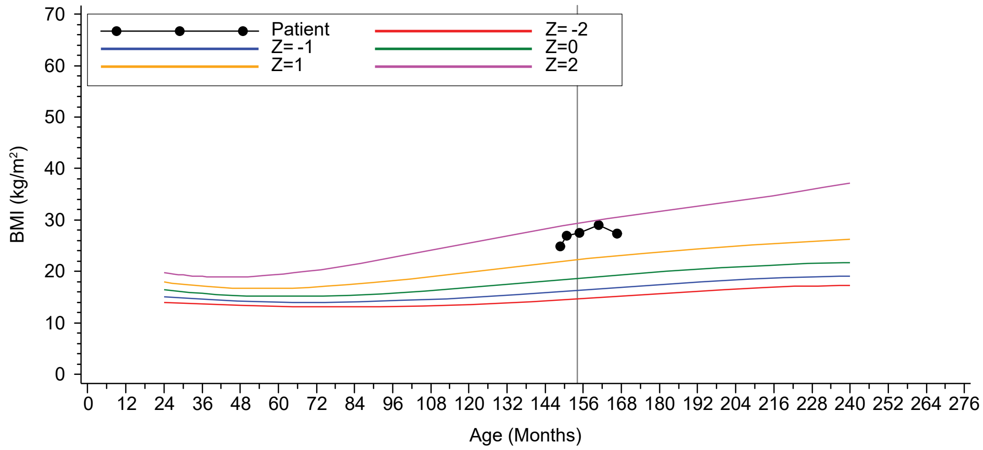

Patient 171

Seizure History: Partial Onset Seizures

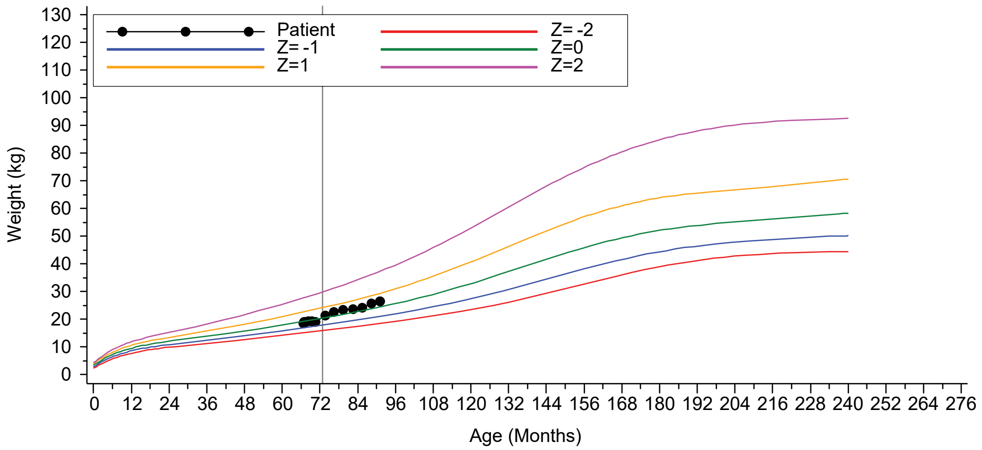

Patient 171

Seizure History: Partial Onset Seizures

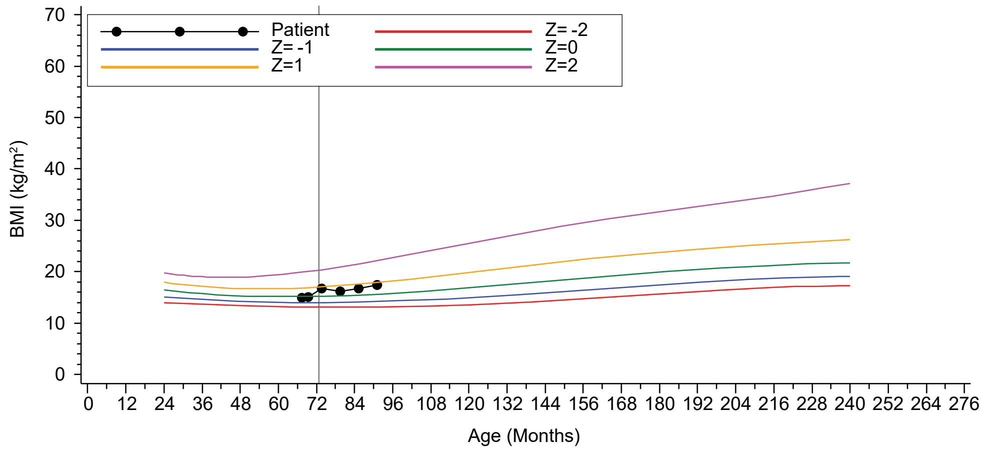

Patient 172

Seizure History: Partial Onset Seizures

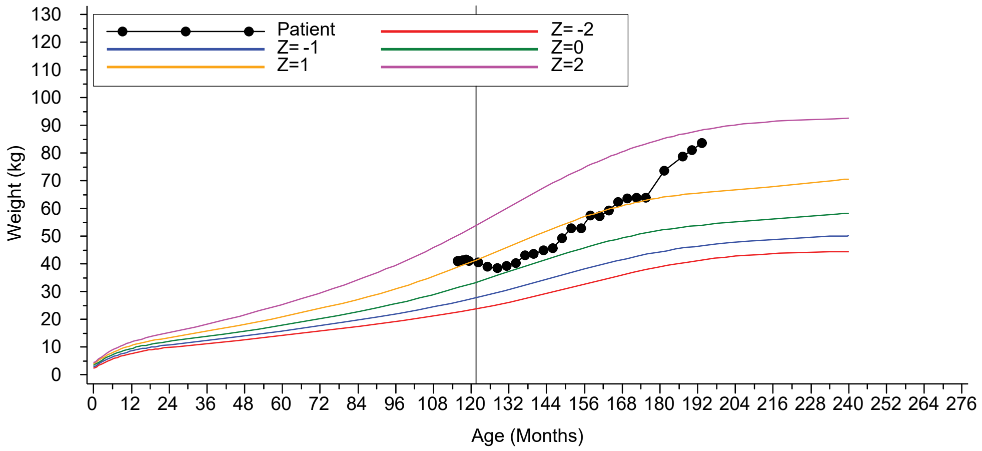

Patient 172

Seizure History: Partial Onset Seizures

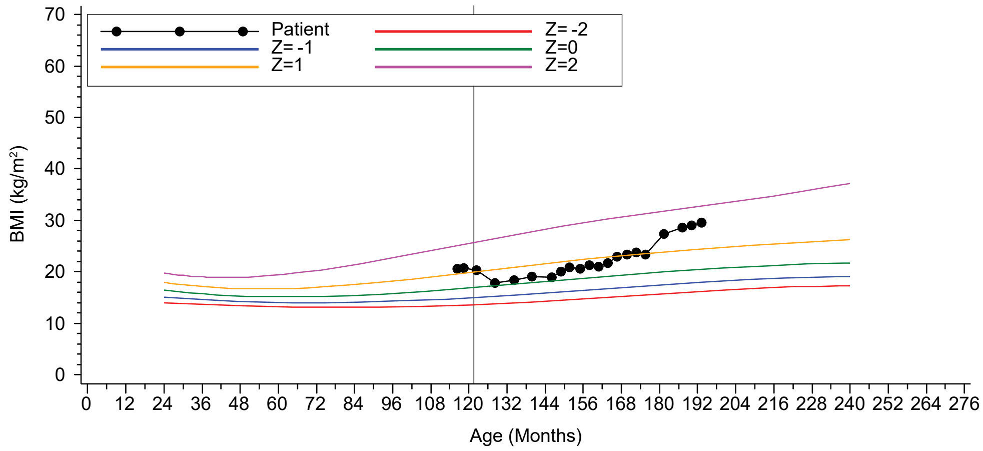

Patient 173

Seizure History: Partial Onset Seizures

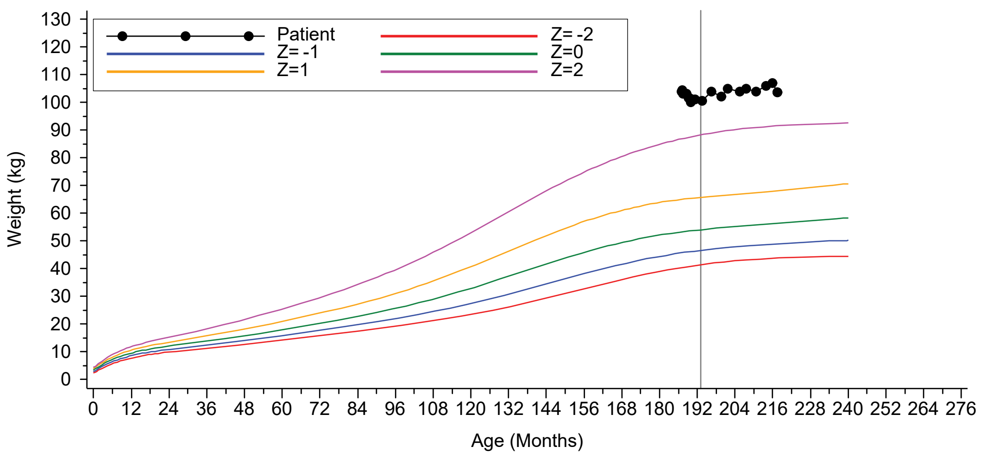

Patient 173

Seizure History: Partial Onset Seizures

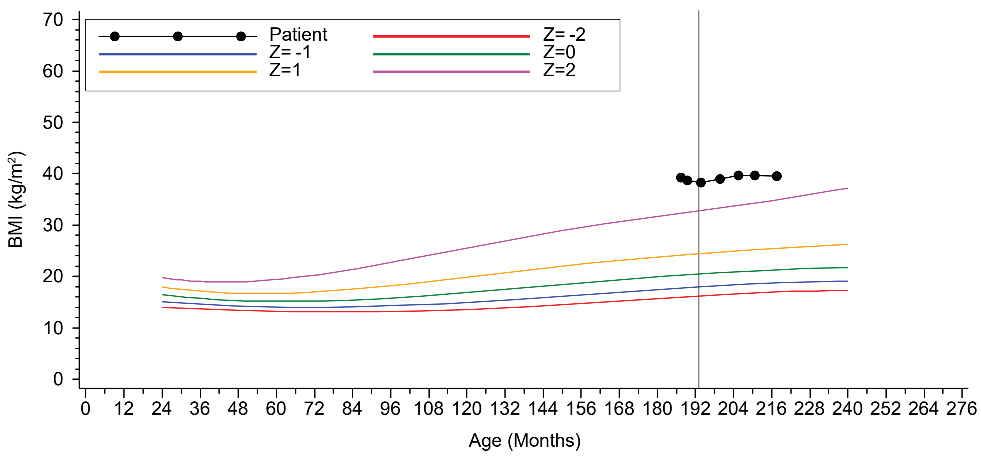

Patient 174

Seizure History: Partial Onset Seizures

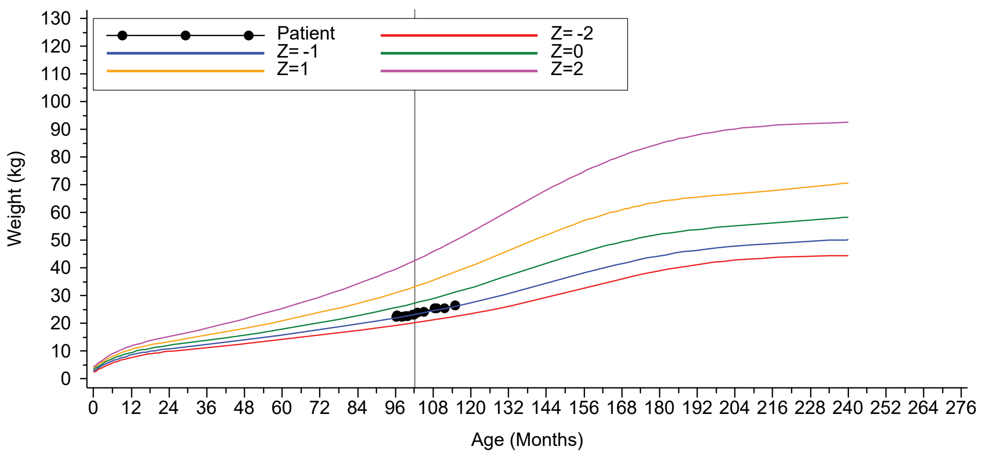

Patient 174

Seizure History: Partial Onset Seizures

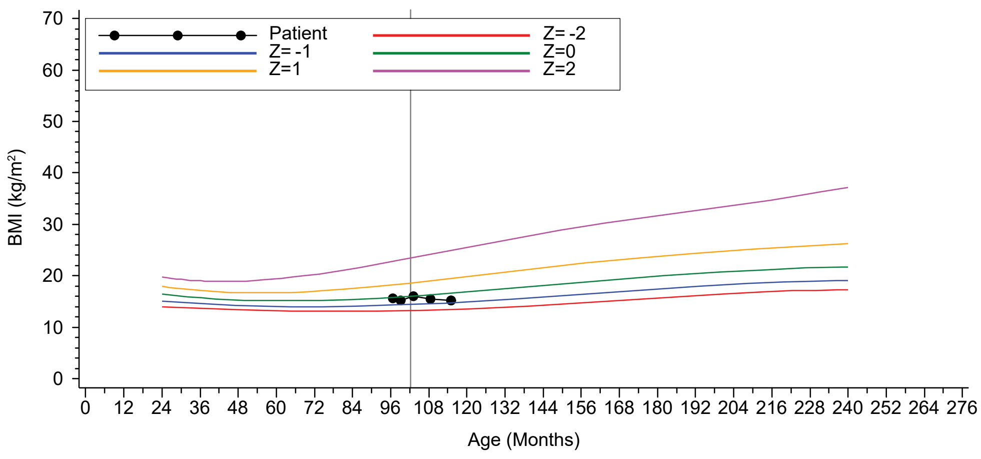

Patient 175

Seizure History: Partial Onset Seizures

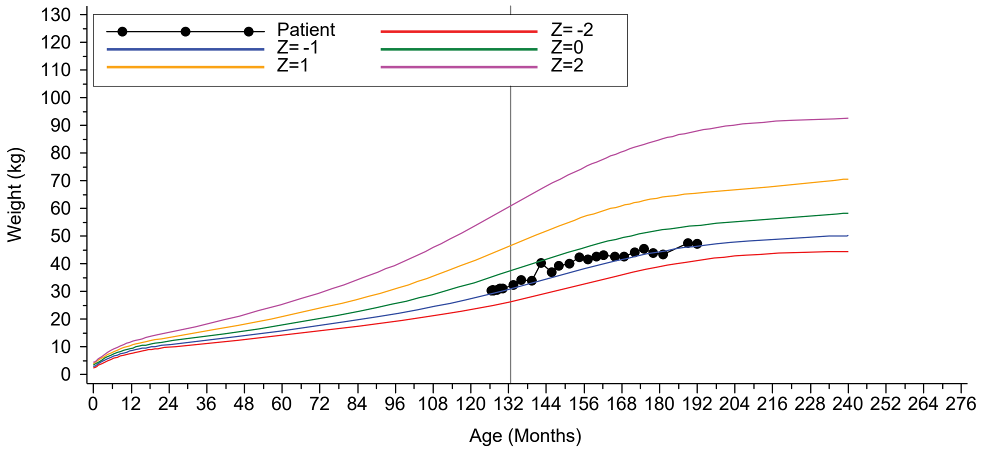

Patient 175

Seizure History: Partial Onset Seizures

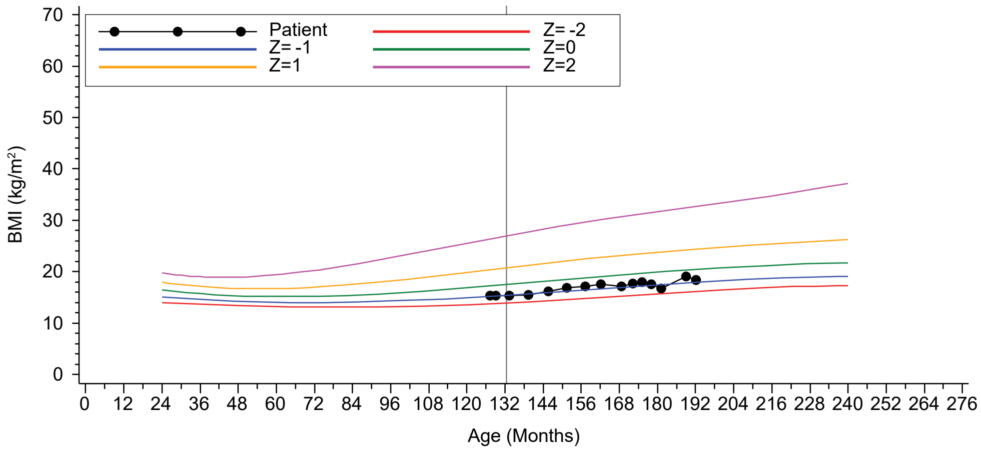

Patient 176

Seizure History: Partial Onset Seizures

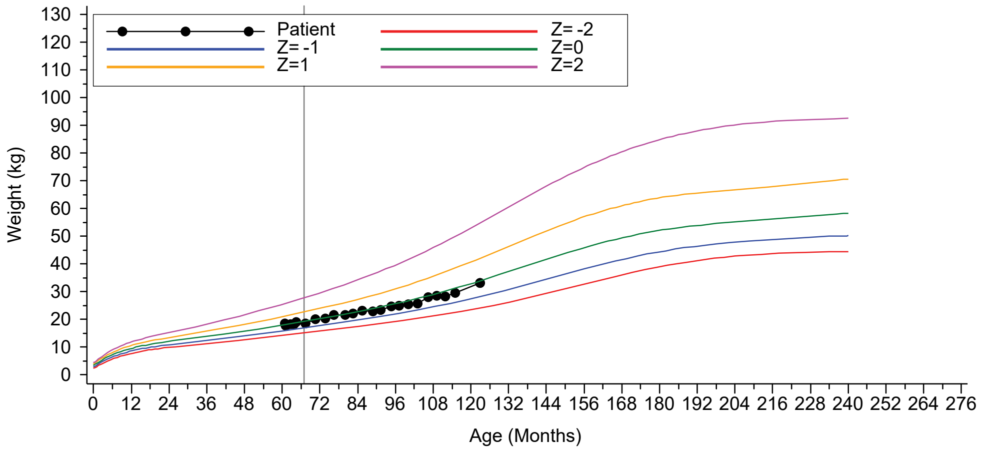

Patient 176

Seizure History: Partial Onset Seizures

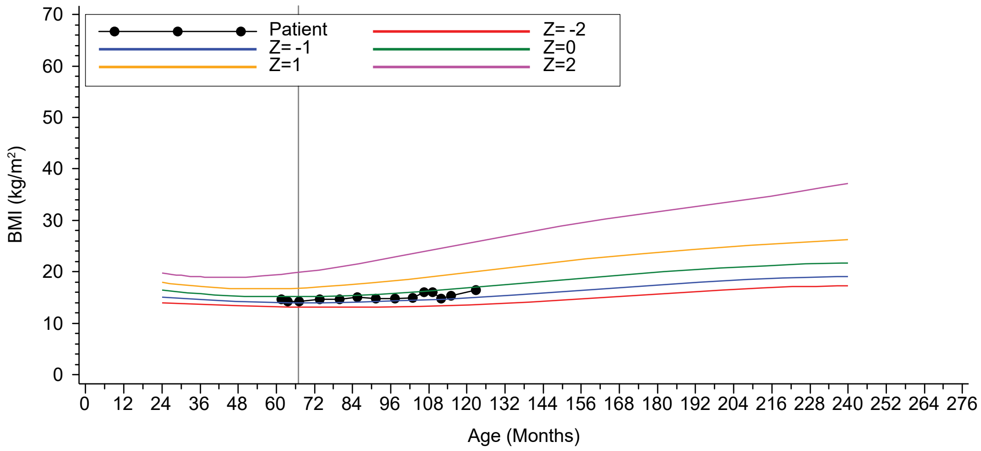

Patient 177

Seizure History: Partial Onset Seizures

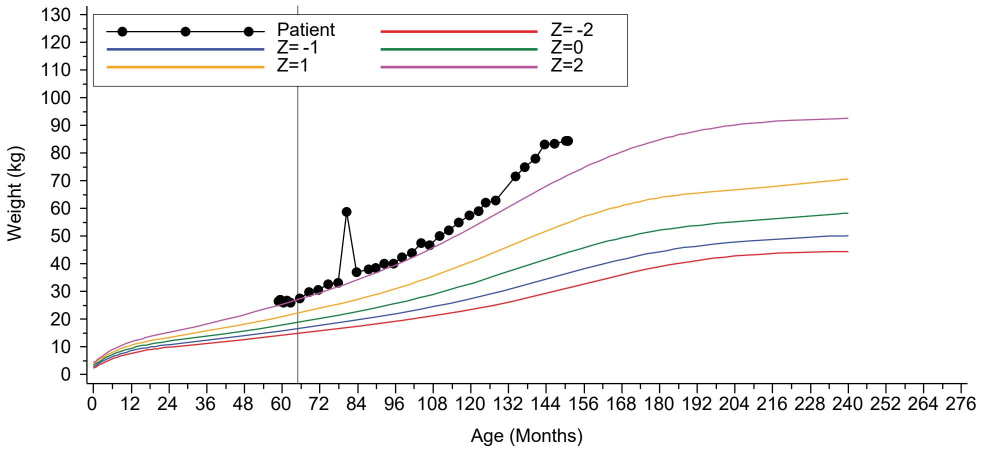

Patient 177

Seizure History: Partial Onset Seizures

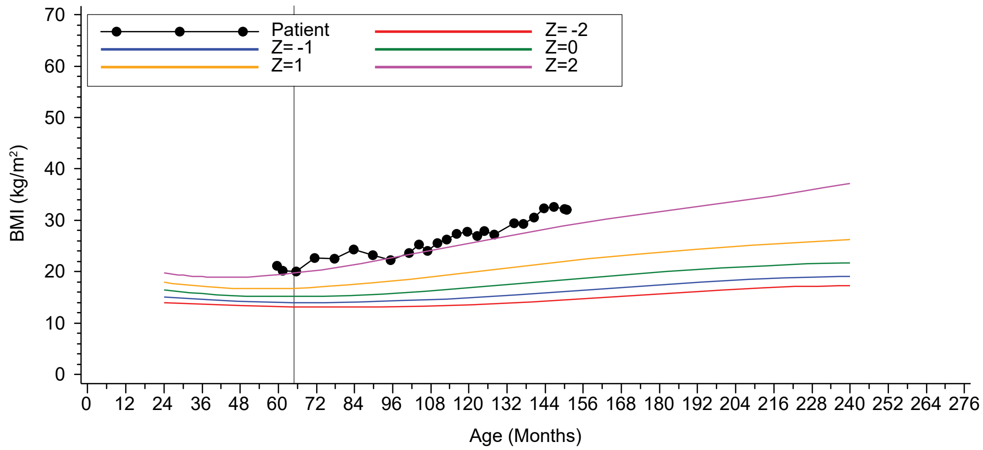

Patient 178

Seizure History: Primary Generalized Seizures and Unknown

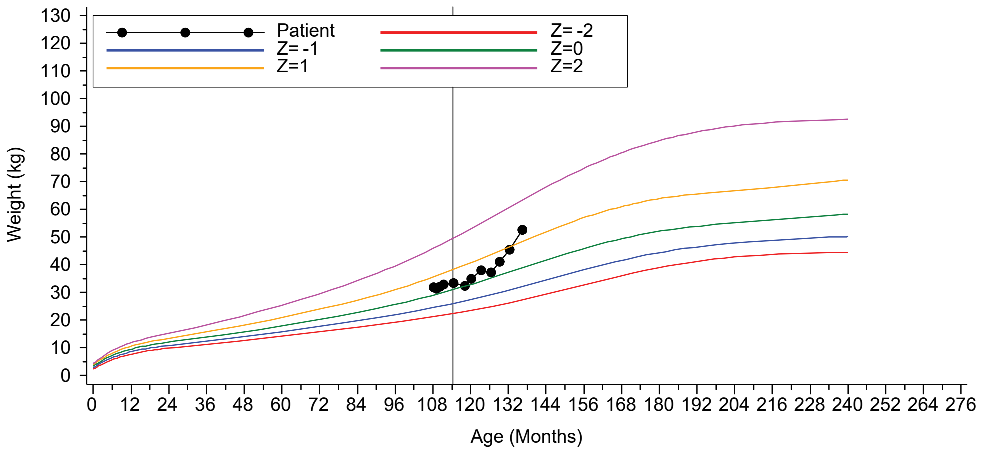

Patient 178

Seizure History: Primary Generalized Seizures and Unknown

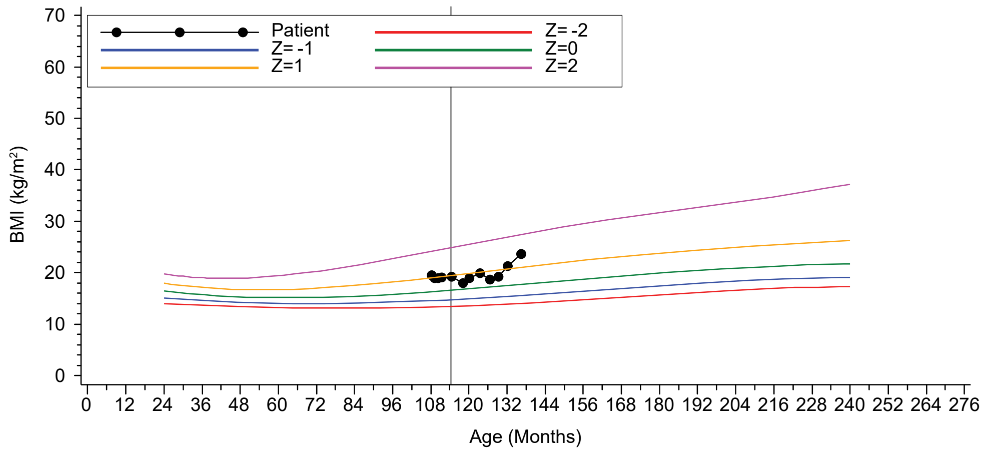

Patient 179

Seizure History: Primary Generalized Seizures and Unknown

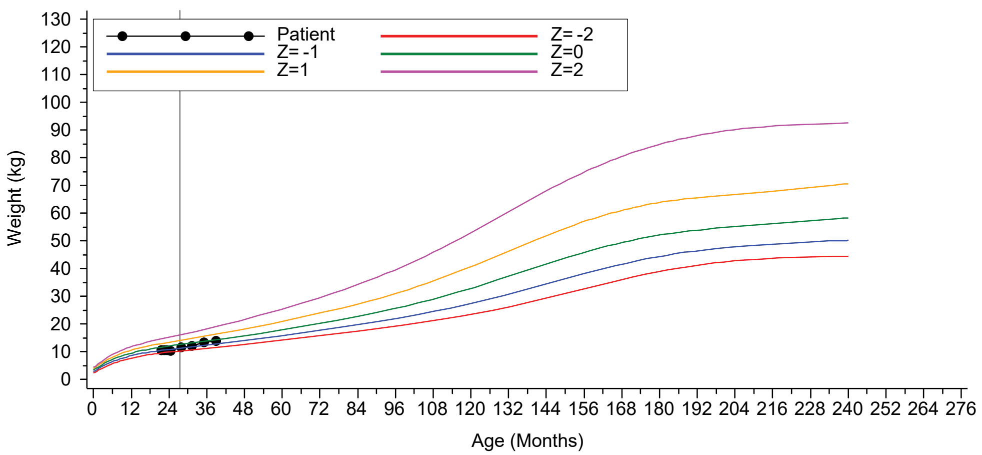

Patient 179

Seizure History: Primary Generalized Seizures and Unknown

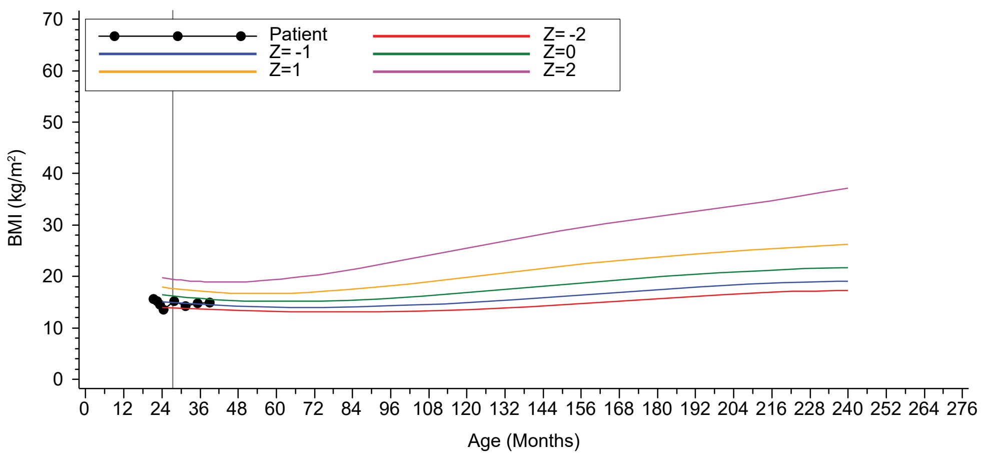

Patient 180

Seizure History: Primary Generalized Seizures and Unknown

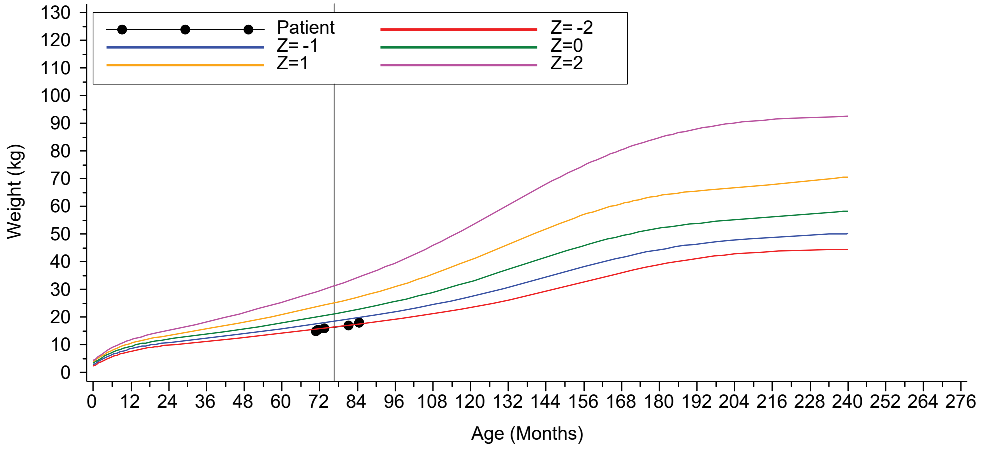

Patient 180

Seizure History: Primary Generalized Seizures and Unknown

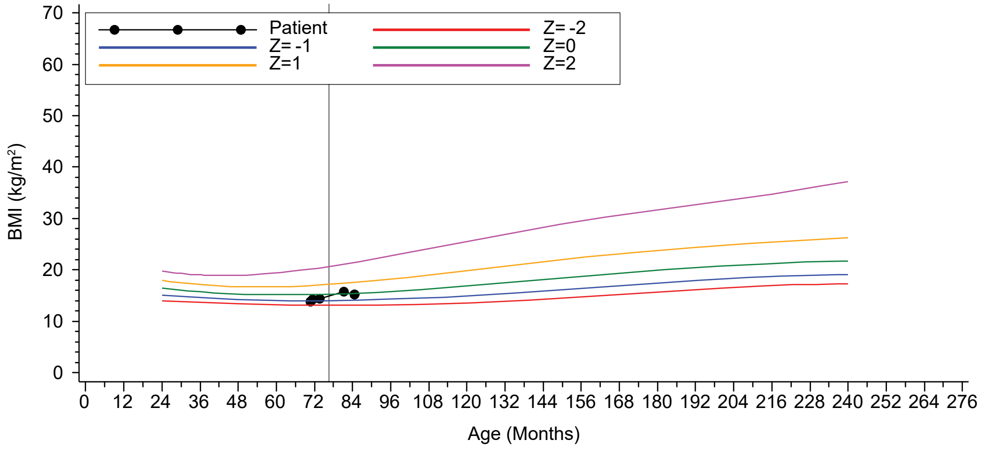

Patient 181
Seizure History: Primary Generalized Seizures and Unknown

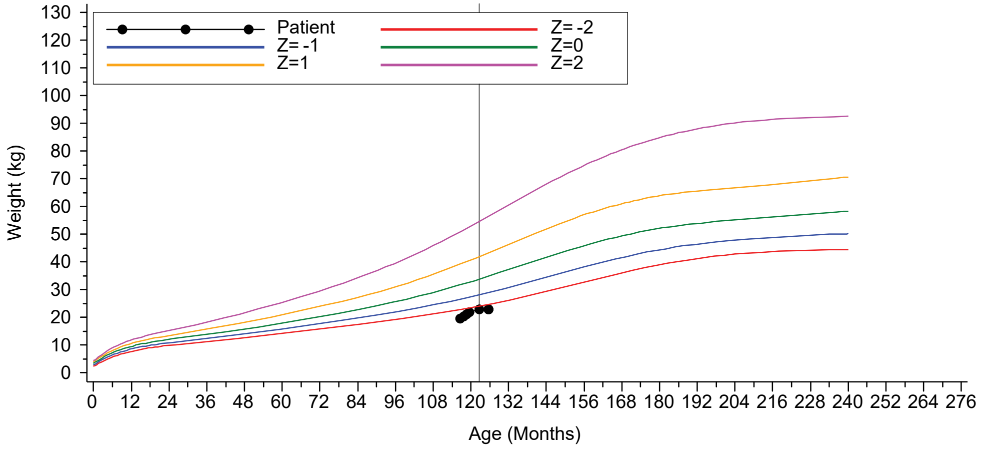

Patient 181
Seizure History: Primary Generalized Seizures and Unknown

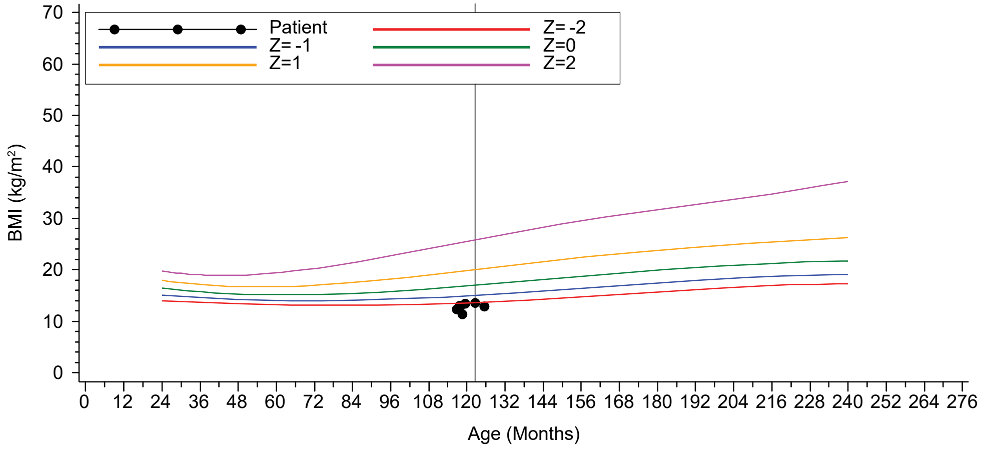

Patient 182
Seizure History: Primary Generalized Seizures and Unknown

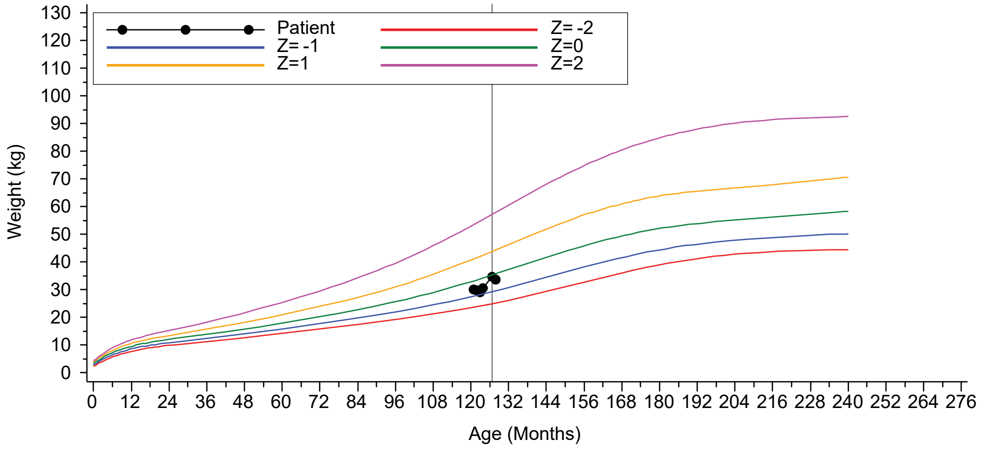

Patient 182
Seizure History: Primary Generalized Seizures and Unknown

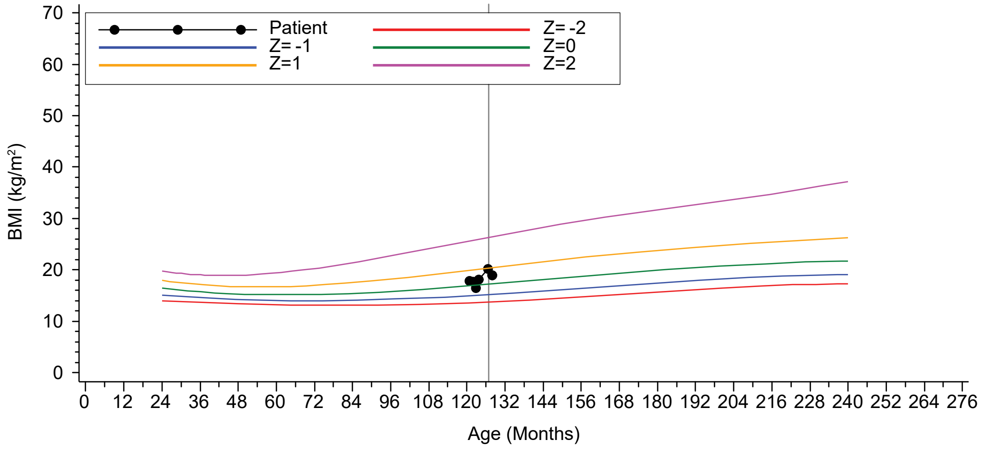

Patient 183

Seizure History: Primary Generalized Seizures and Unknown

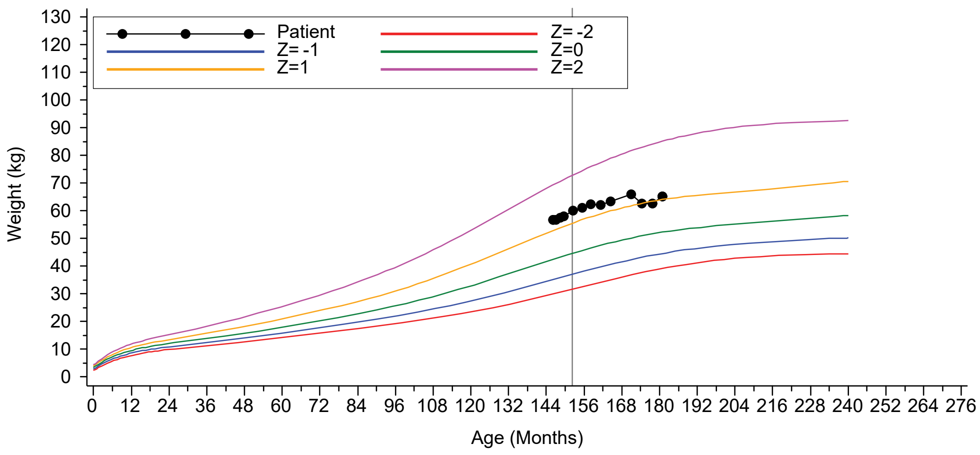

Patient 183

Seizure History: Primary Generalized Seizures and Unknown

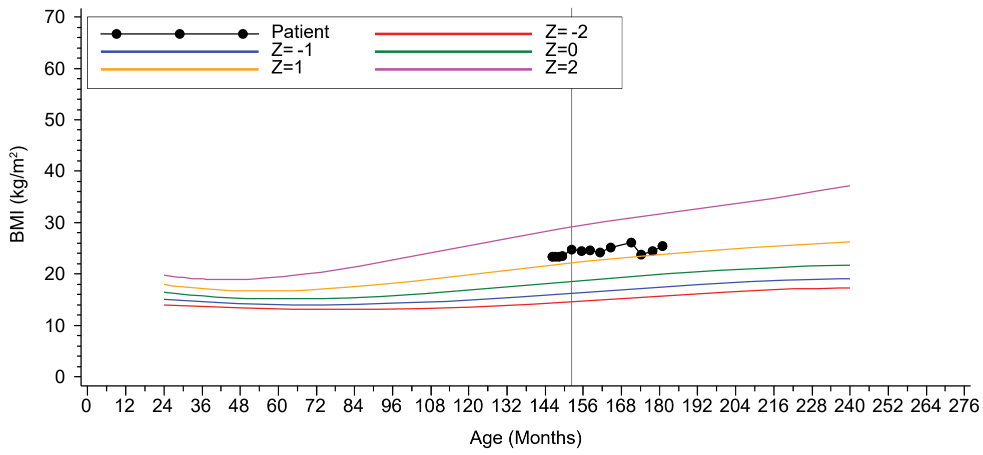

Patient 184

Seizure History: Primary Generalized Seizures and Unknown

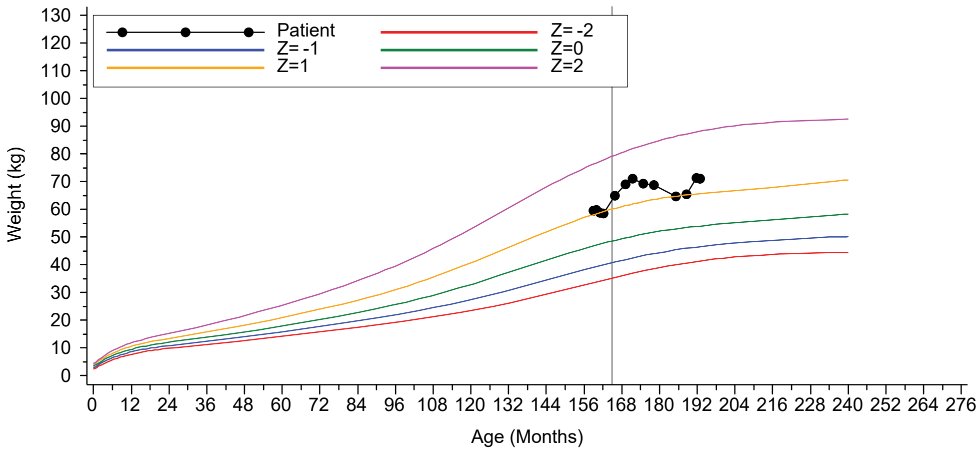

Patient 184

Seizure History: Primary Generalized Seizures and Unknown

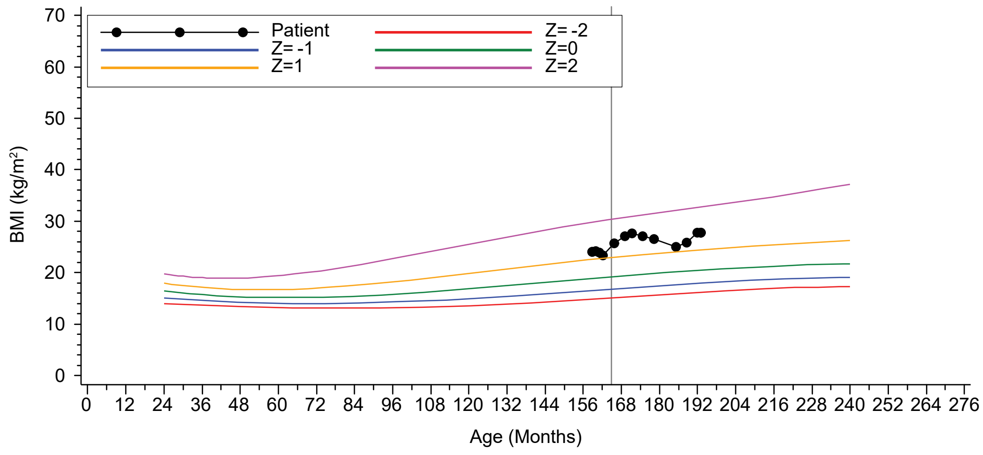

Patient 185

Seizure History: Primary Generalized Seizures and Unknown

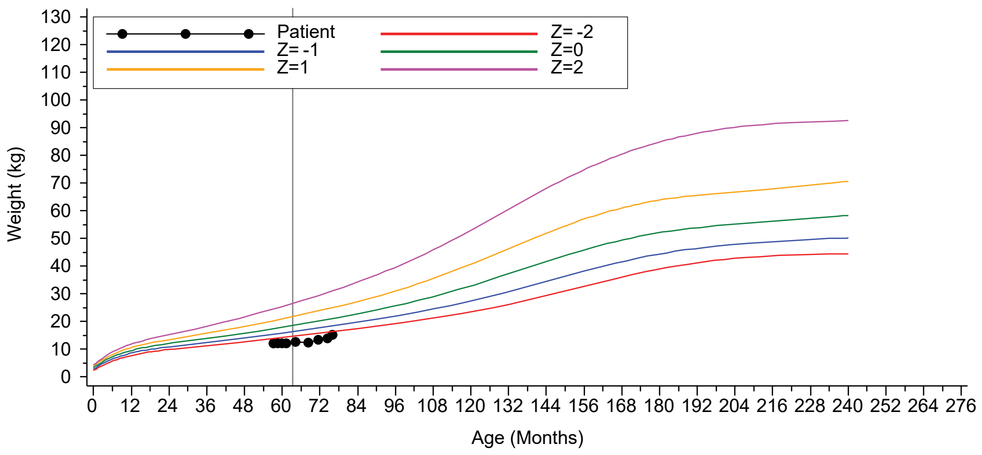

Patient 185

Seizure History: Primary Generalized Seizures and Unknown

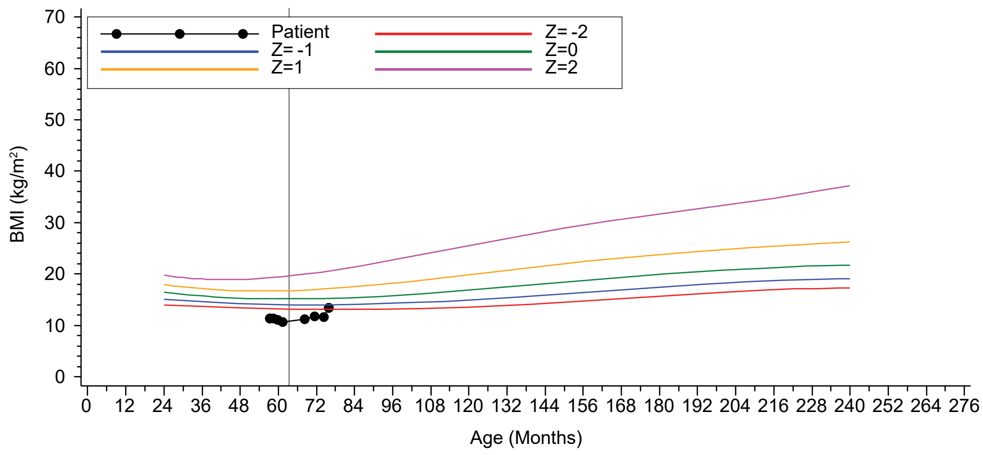

Patient 186

Seizure History: Primary Generalized Seizures and Unknown

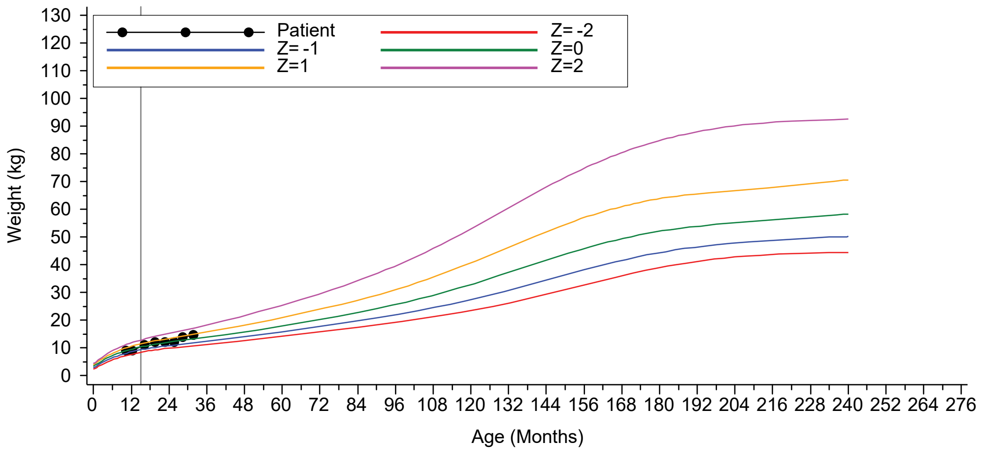

Patient 186

Seizure History: Primary Generalized Seizures and Unknown

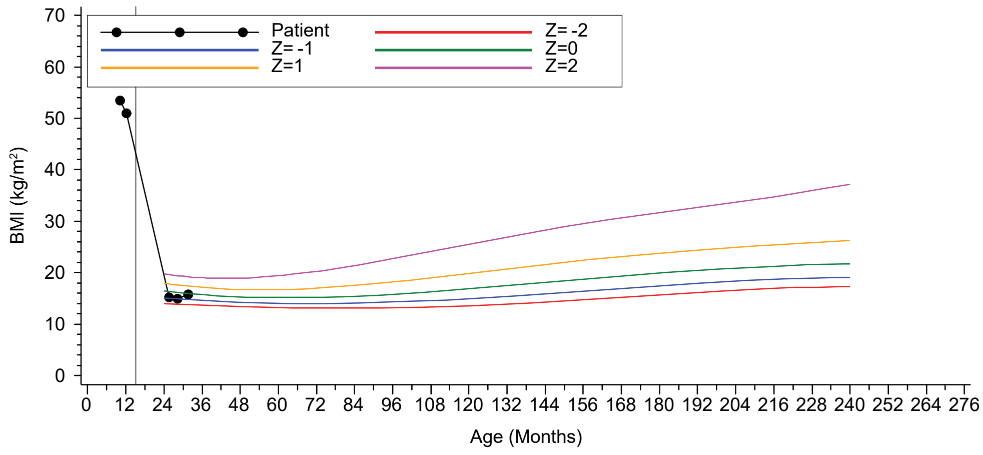

Patient 187

Seizure History: Primary Generalized Seizures and Unknown

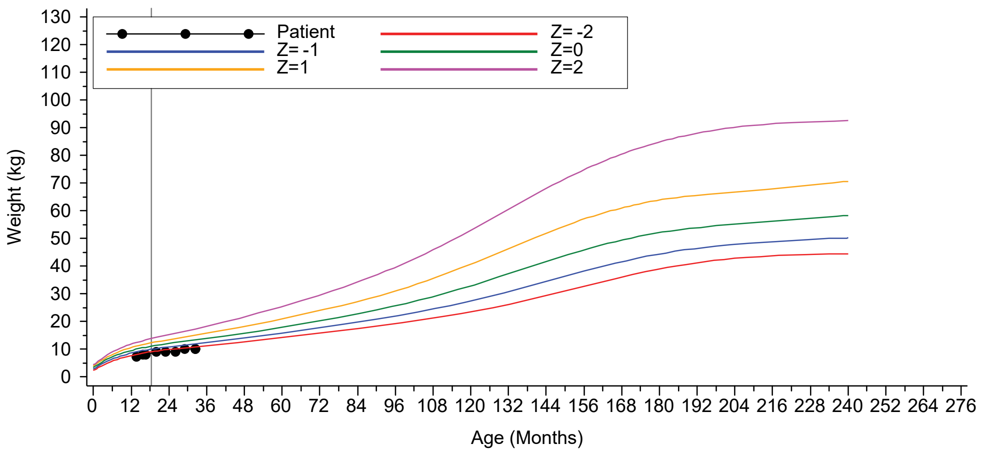

Patient 187

Seizure History: Primary Generalized Seizures and Unknown

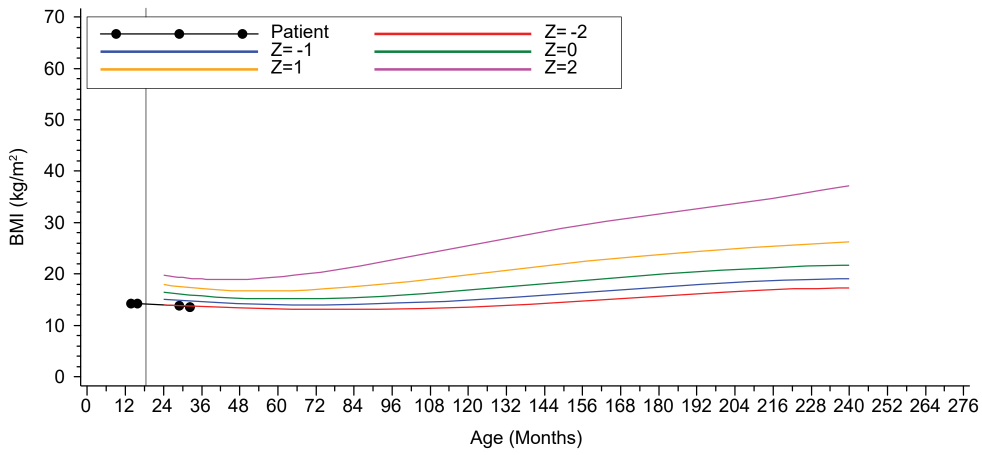

Patient 188

Seizure History: Primary Generalized Seizures and Unknown

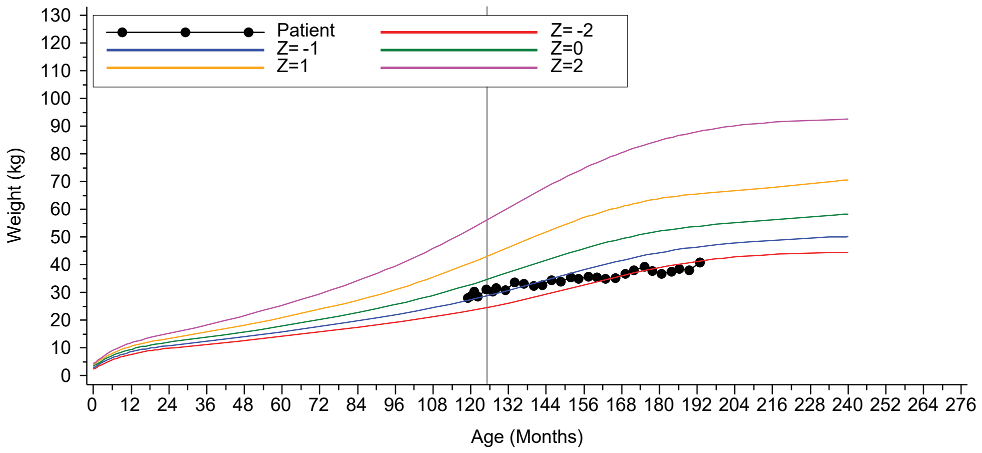

Patient 188

Seizure History: Primary Generalized Seizures and Unknown

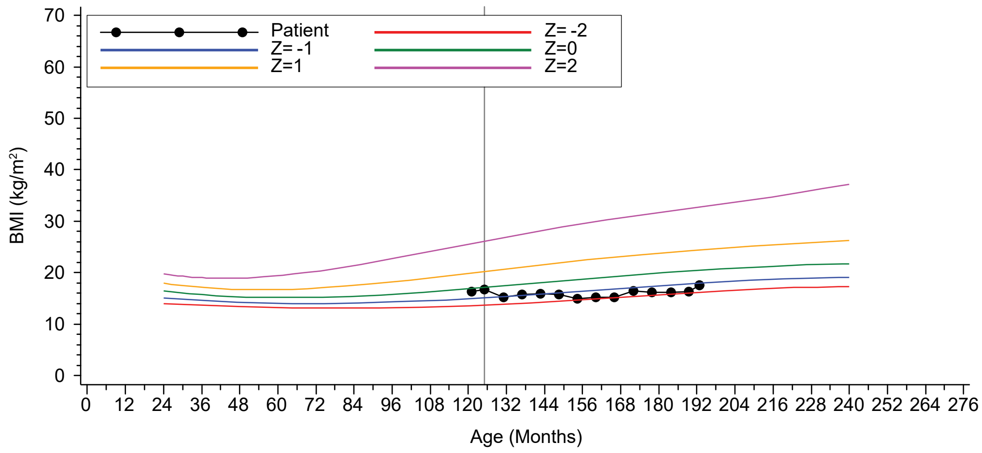

Patient 189

Seizure History: Primary Generalized Seizures and Unknown

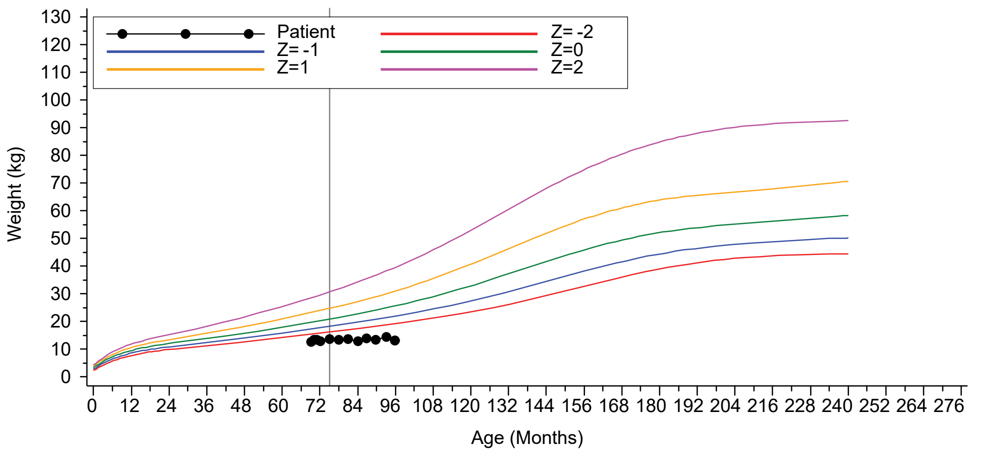

Patient 189

Seizure History: Primary Generalized Seizures and Unknown

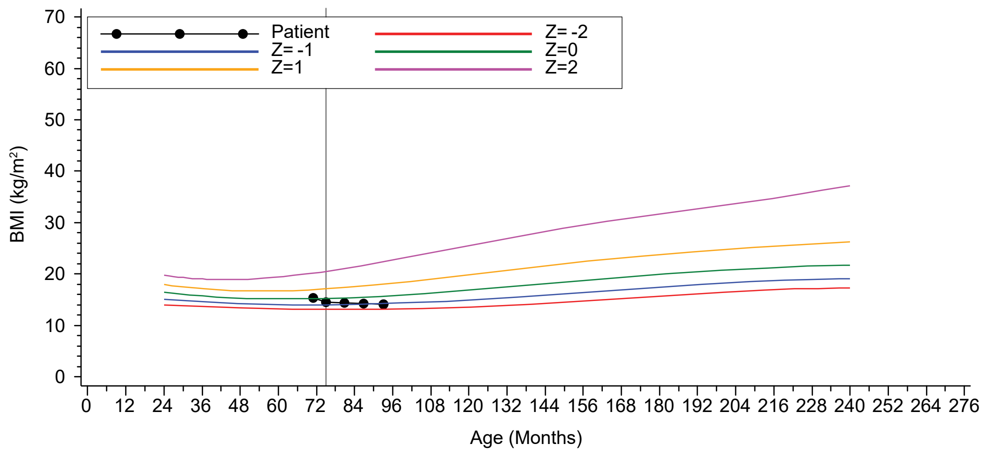

Patient 190

Seizure History: Primary Generalized Seizures and Unknown

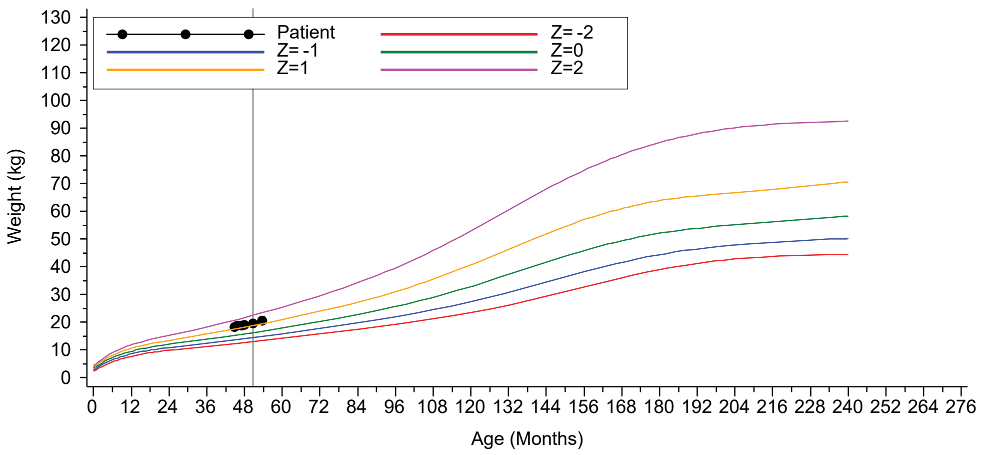

Patient 190

Seizure History: Primary Generalized Seizures and Unknown

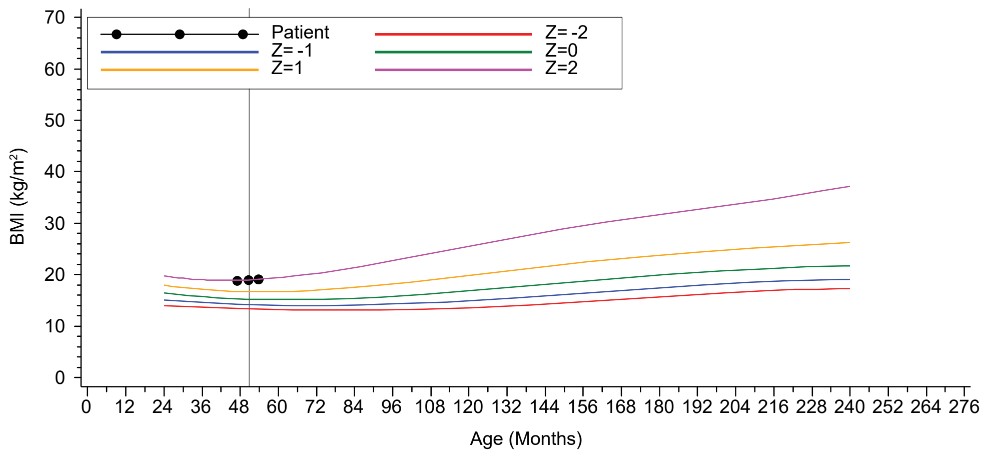

Patient 191

Seizure History: Primary Generalized Seizures and Unknown

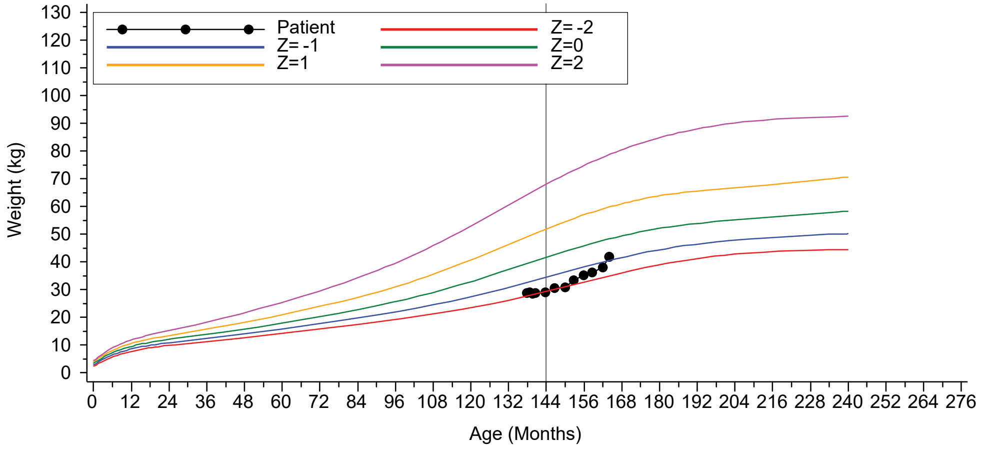

Patient 191

Seizure History: Primary Generalized Seizures and Unknown

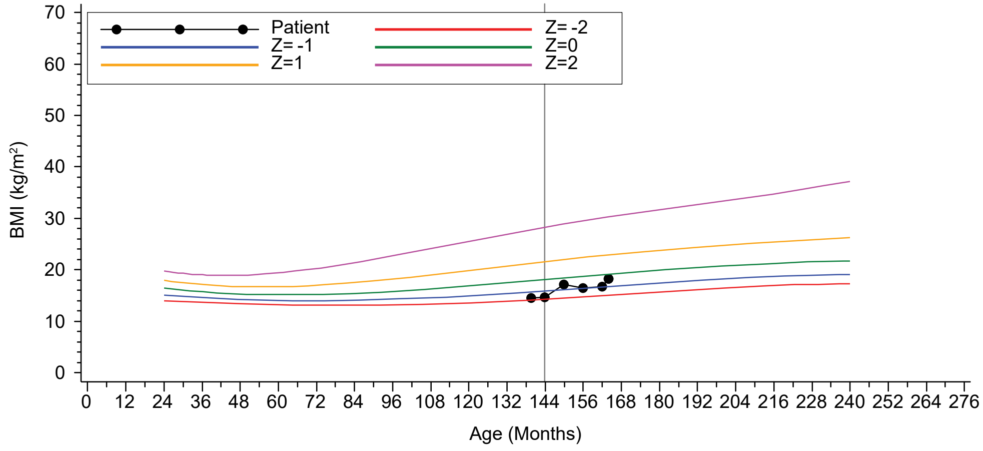

Patient 192

Seizure History: Primary Generalized Seizures and Unknown

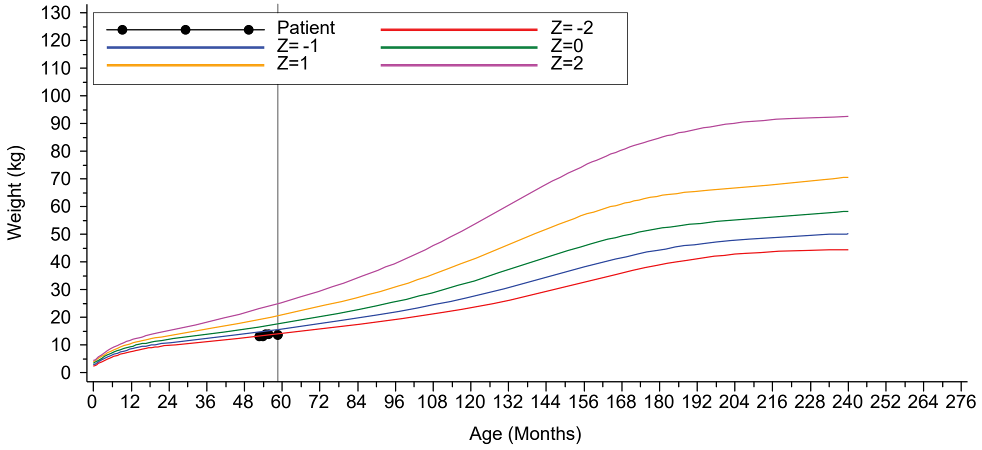

Patient 192

Seizure History: Primary Generalized Seizures and Unknown

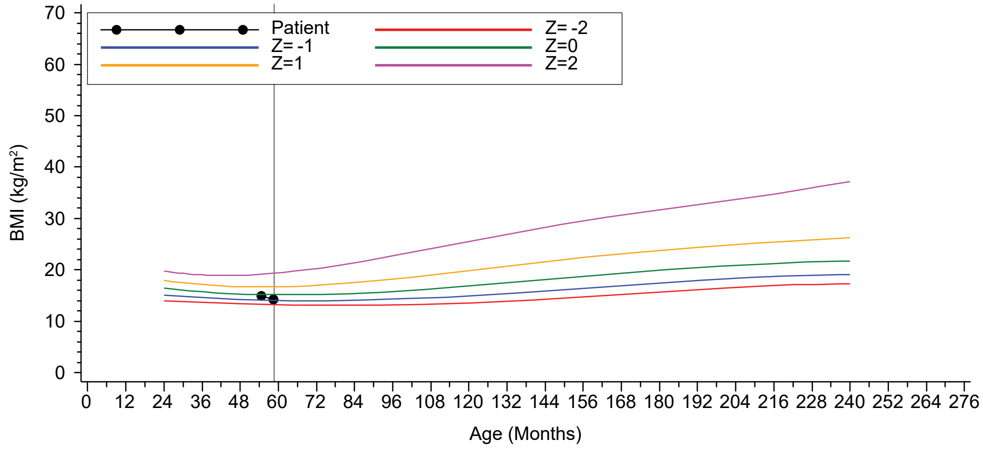

Patient 193

Seizure History: Primary Generalized Seizures and Unknown

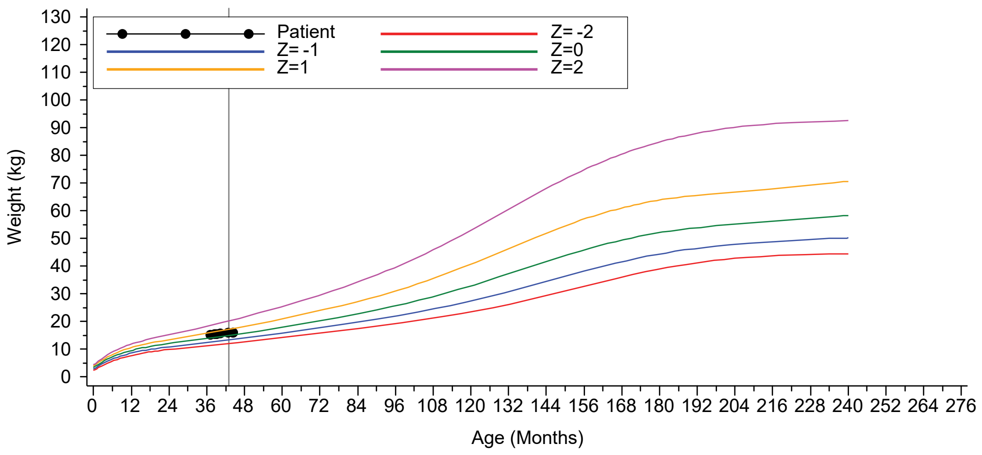

Patient 193

Seizure History: Primary Generalized Seizures and Unknown

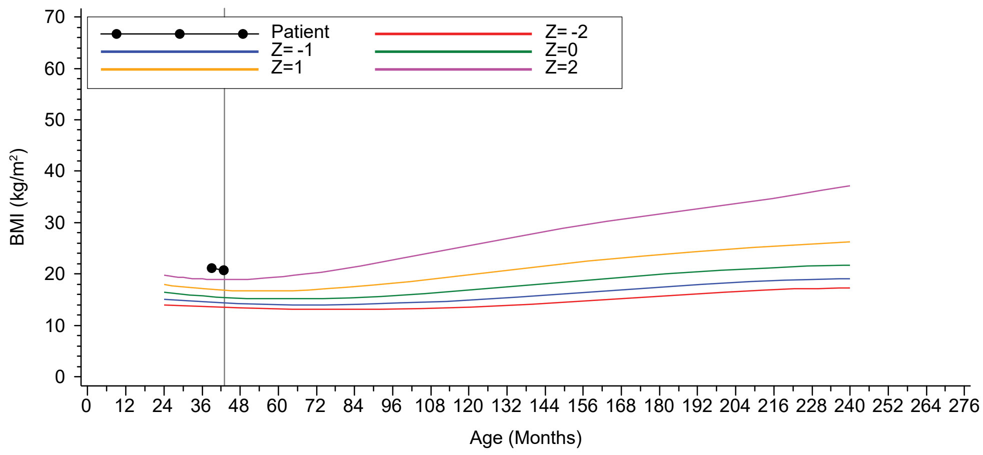

Patient 194

Seizure History: Primary Generalized Seizures and Unknown

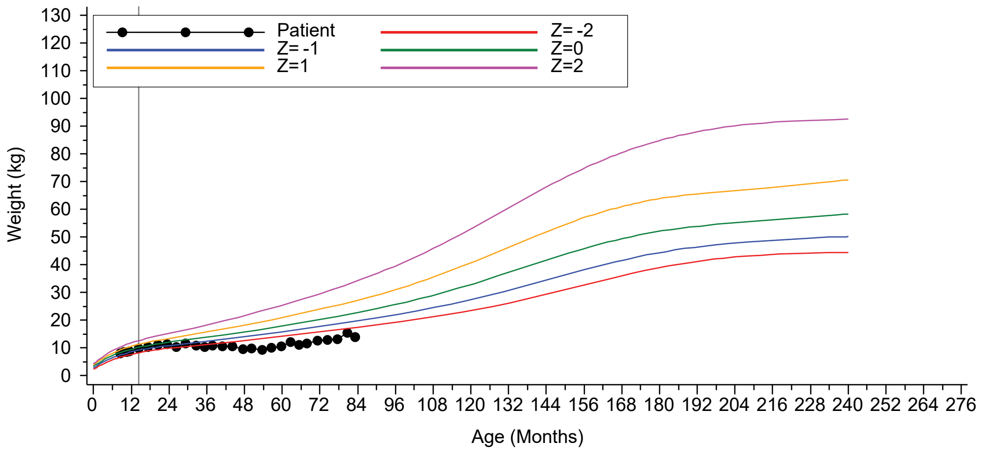

Patient 194

Seizure History: Primary Generalized Seizures and Unknown

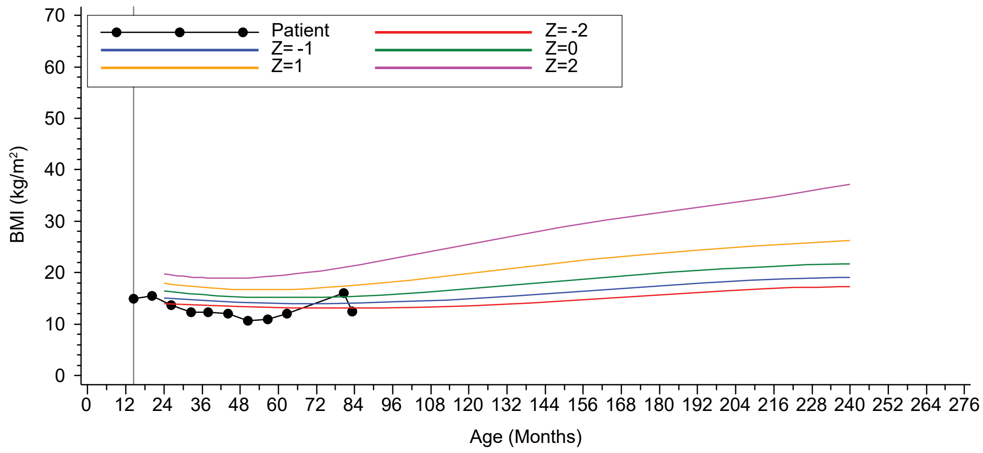

Patient 195

Seizure History: Primary Generalized Seizures and Unknown

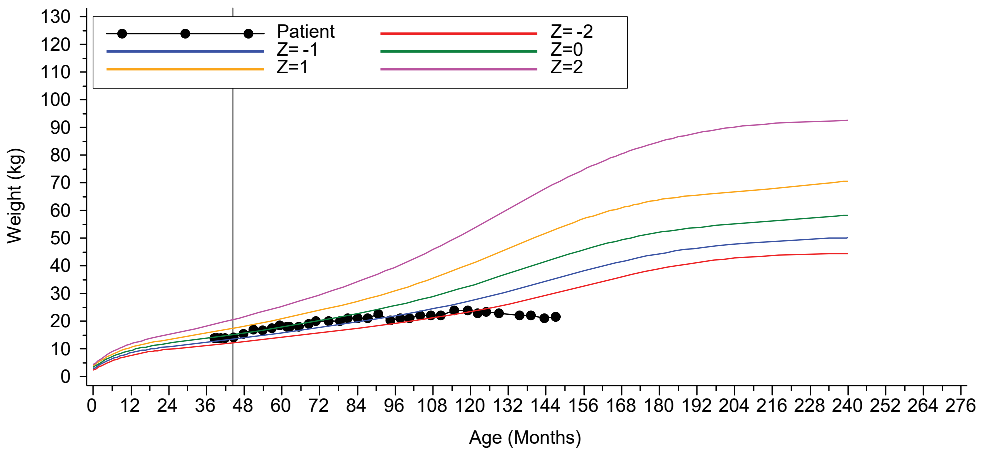

Patient 195

Seizure History: Primary Generalized Seizures and Unknown

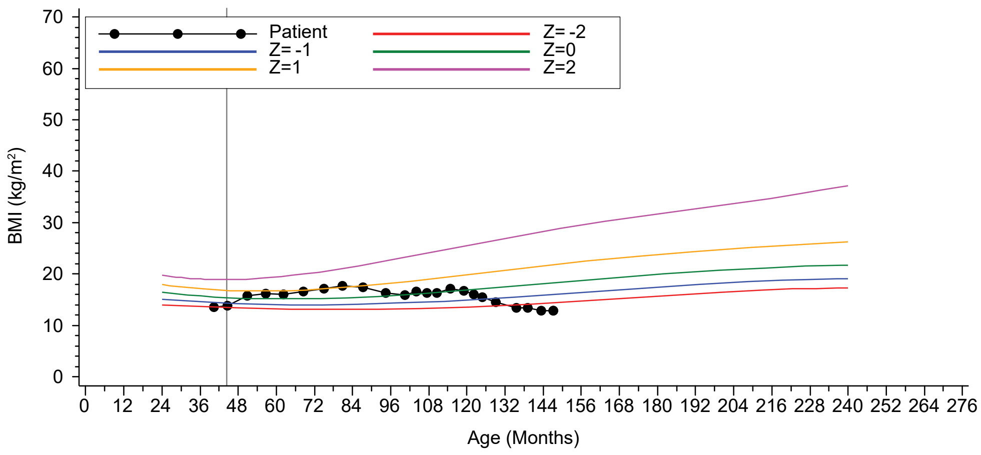

Patient 196

Seizure History: Primary Generalized Seizures and Unknown

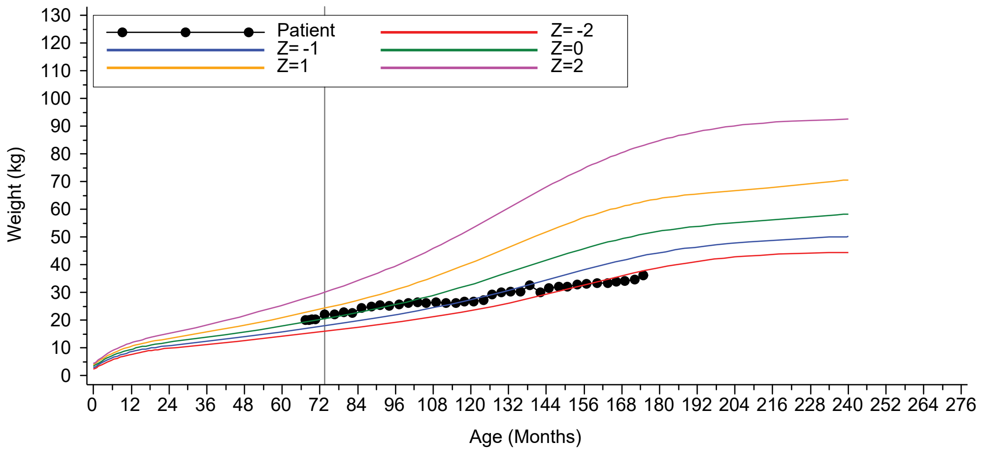

Patient 196

Seizure History: Primary Generalized Seizures and Unknown

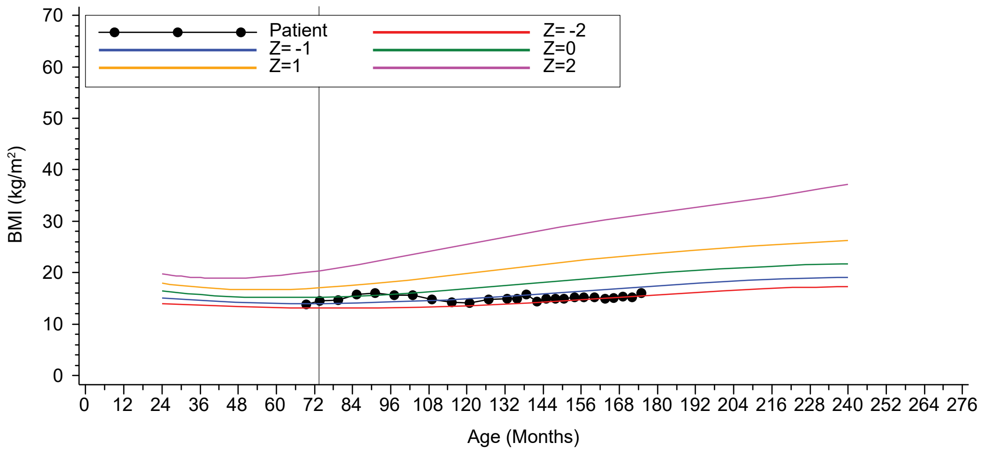

Patient 197

Seizure History: Primary Generalized Seizures and Unknown

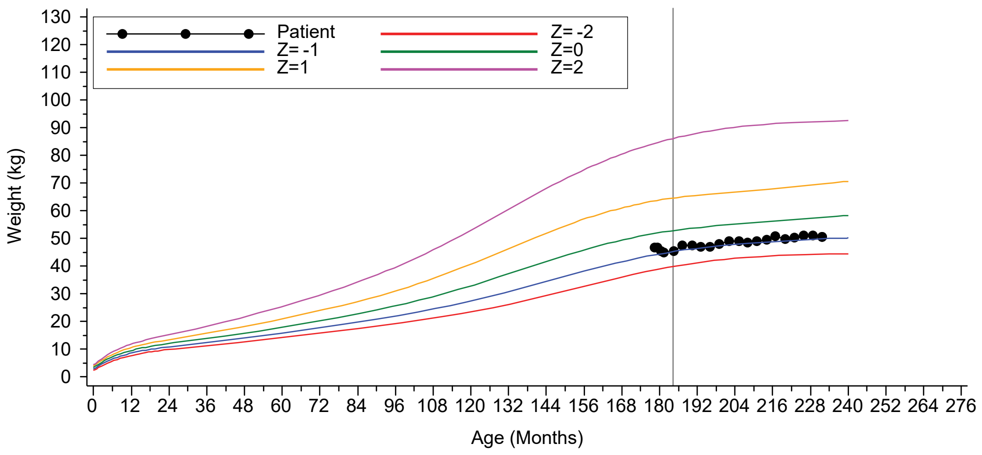

Patient 197

Seizure History: Primary Generalized Seizures and Unknown

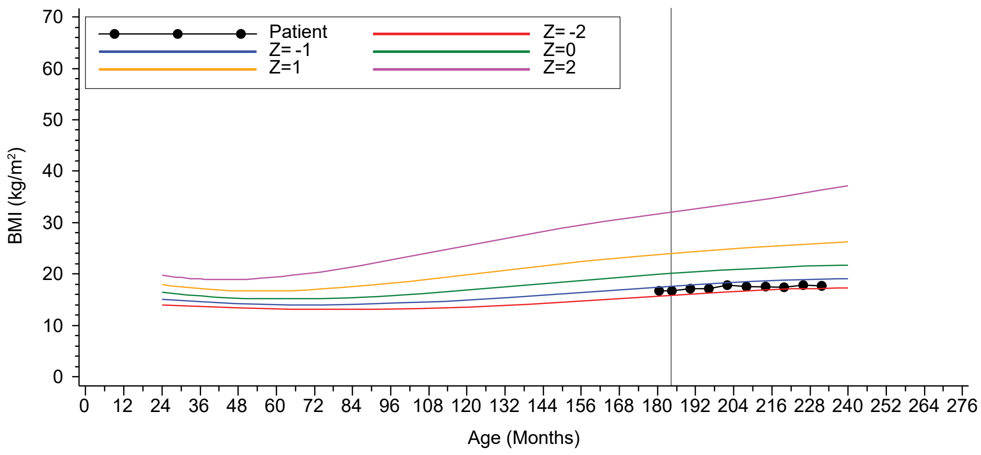

Patient 198

Seizure History: Primary Generalized Seizures and Unknown

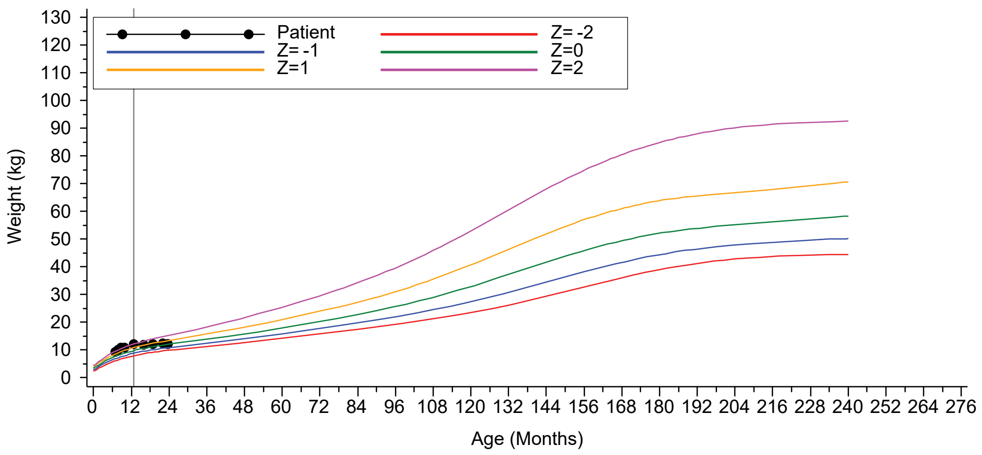

Patient 198

Seizure History: Primary Generalized Seizures and Unknown

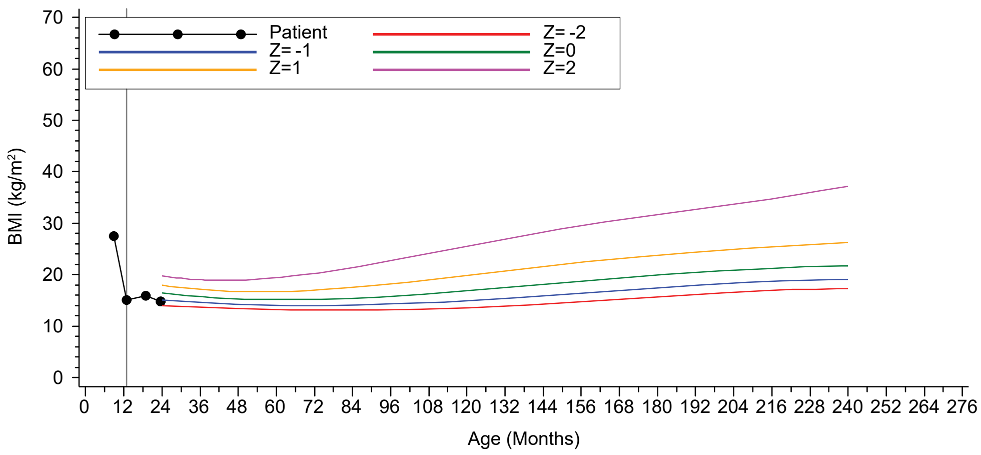

Patient 199

Seizure History: Primary Generalized Seizures and Unknown

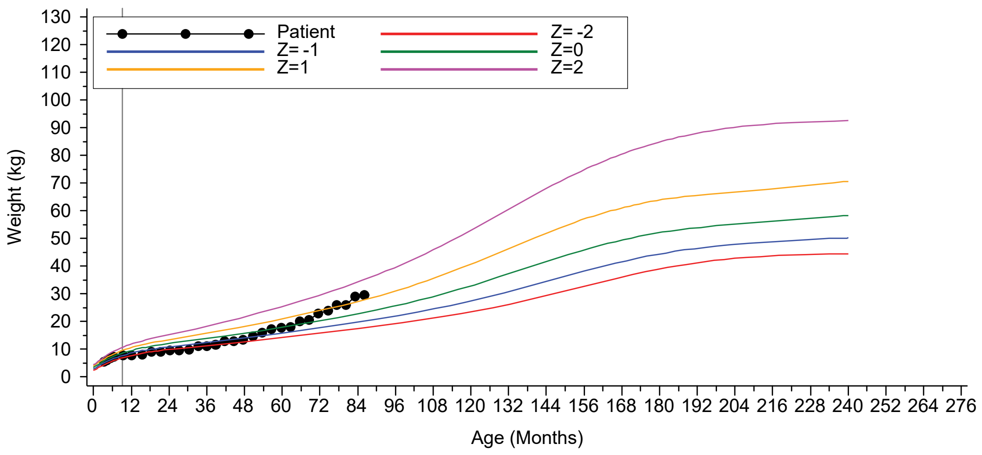

Patient 199

Seizure History: Primary Generalized Seizures and Unknown

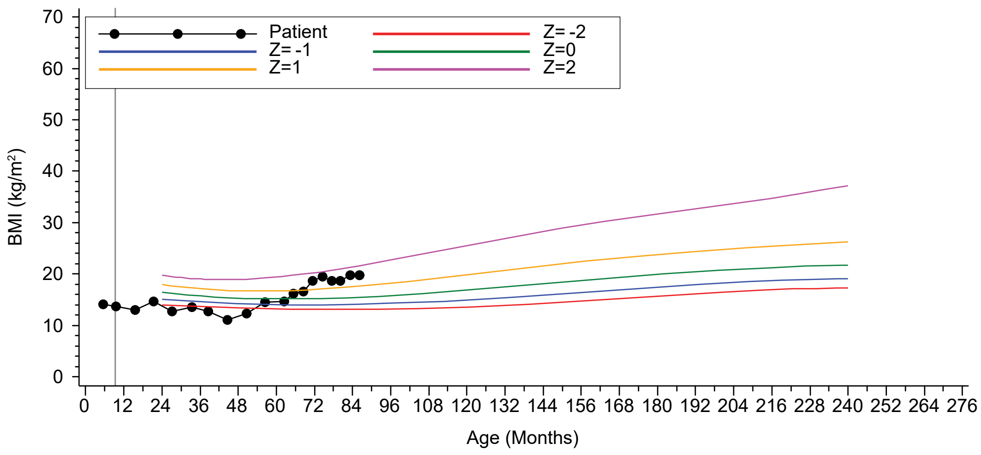

Patient 200

Seizure History: Primary Generalized Seizures and Unknown

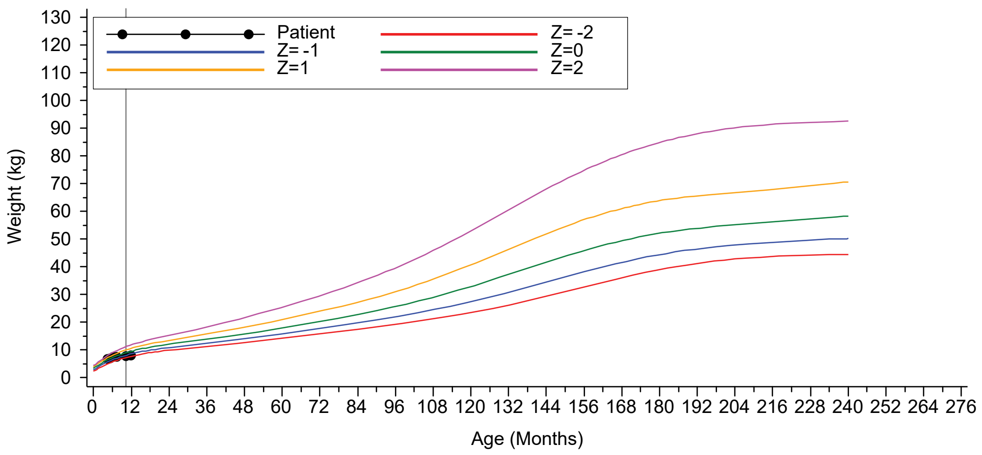

Patient 200

Seizure History: Primary Generalized Seizures and Unknown

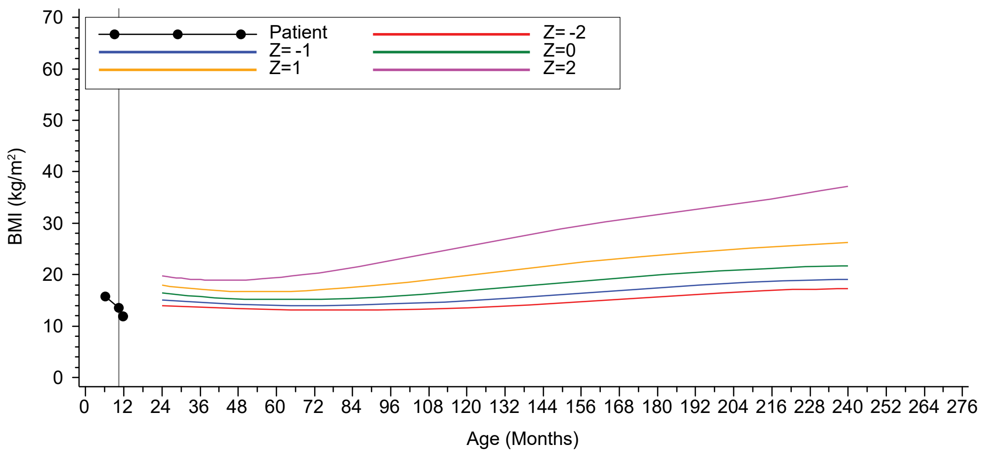

Patient 201
Seizure History: Primary Generalized Seizures and Unknown

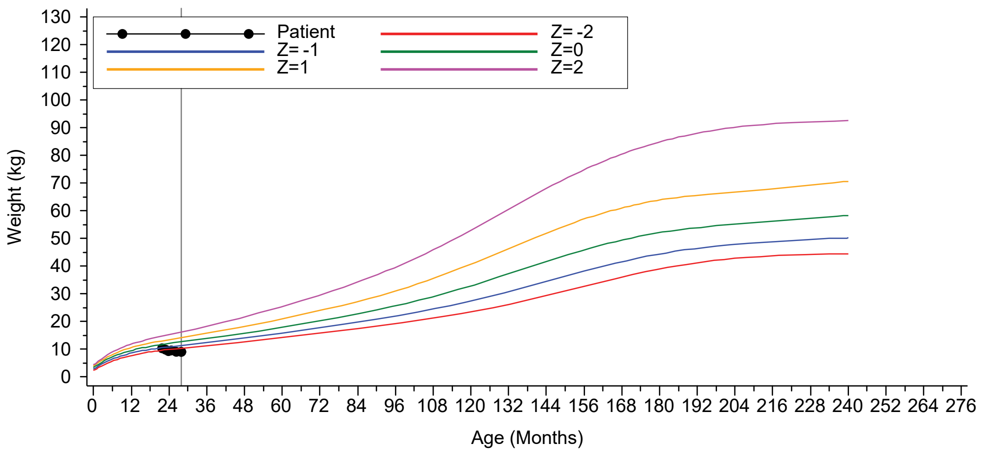

Patient 201
Seizure History: Primary Generalized Seizures and Unknown

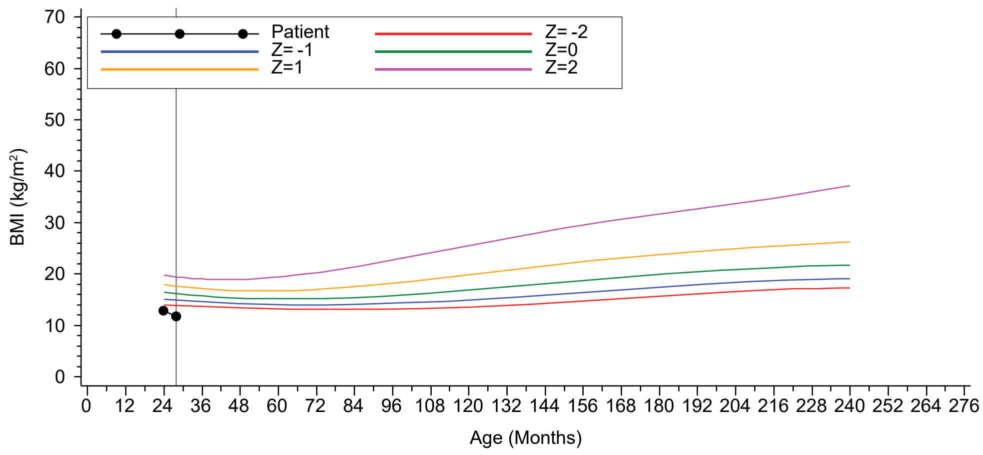

Patient 202
Seizure History: Primary Generalized Seizures and Unknown

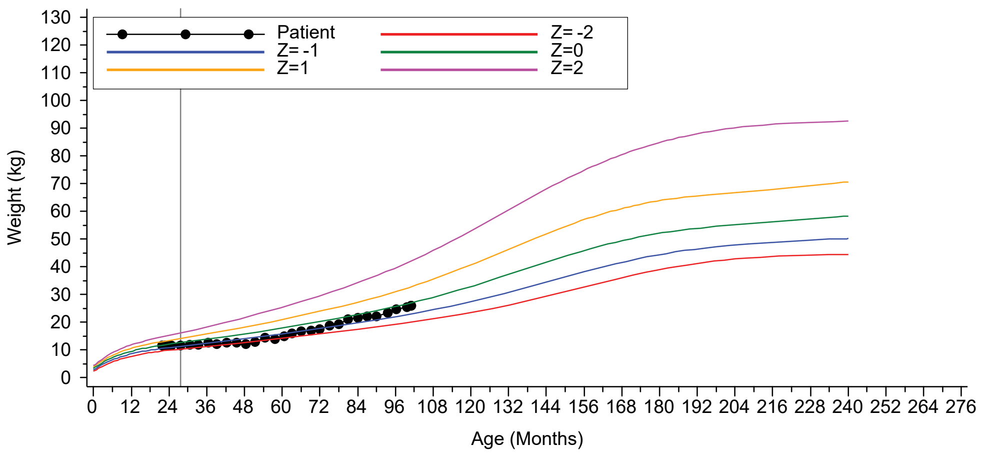

Patient 202
Seizure History: Primary Generalized Seizures and Unknown

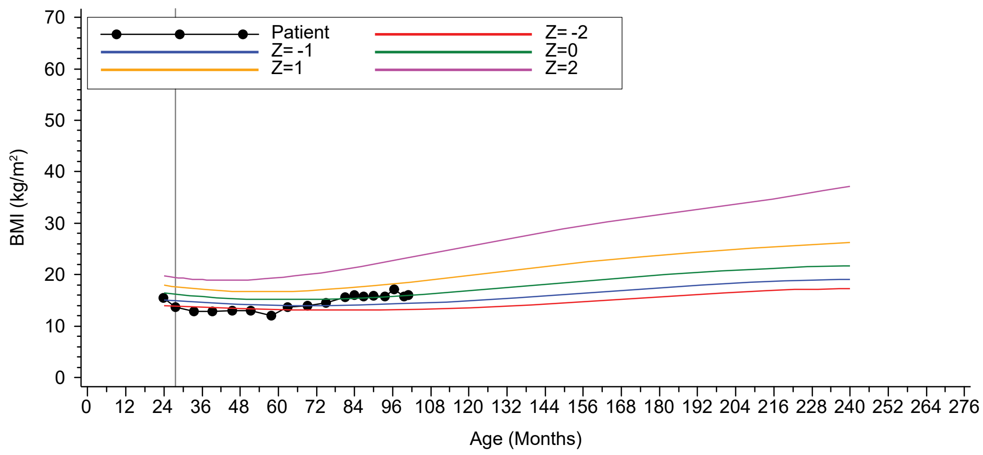

Patient 203
Seizure History: Primary Generalized Seizures and Unknown

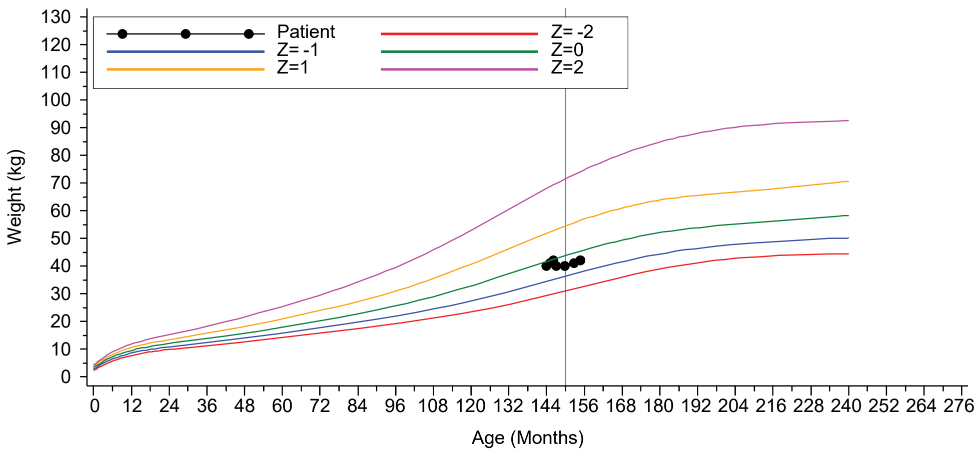

Patient 203
Seizure History: Primary Generalized Seizures and Unknown

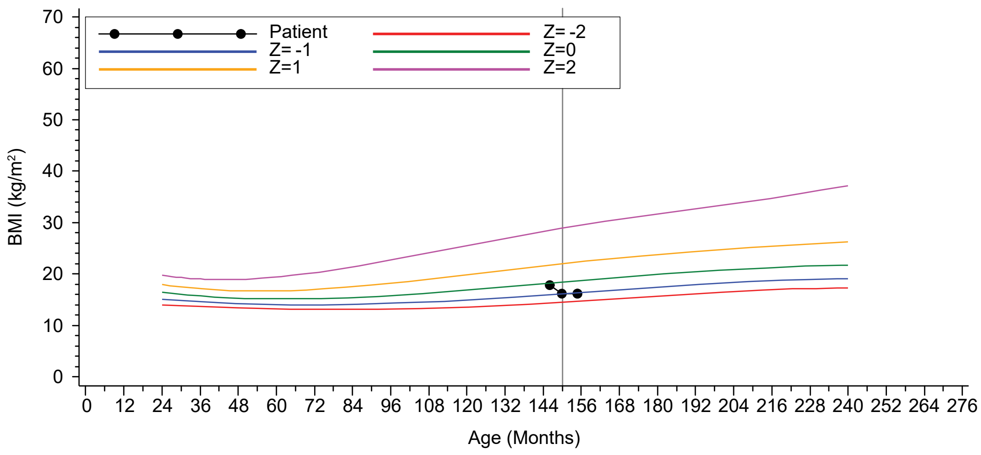

Patient 204
Seizure History: Primary Generalized Seizures and Unknown

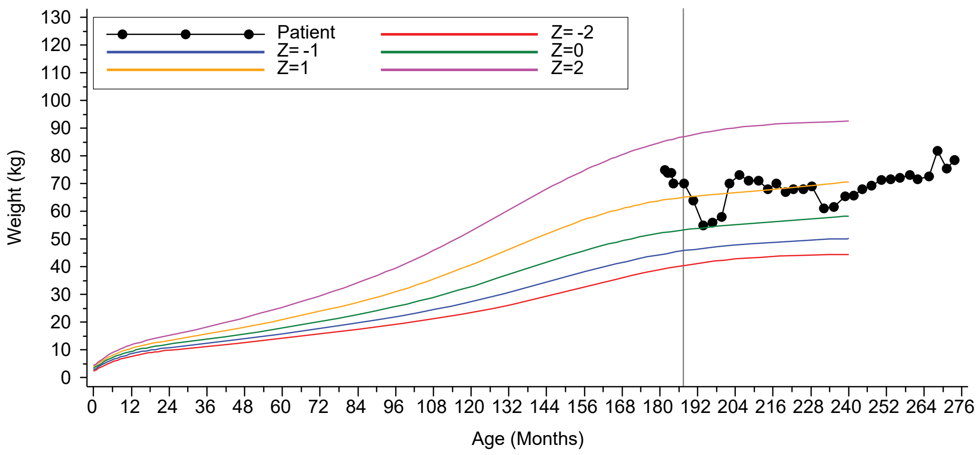

Patient 204
Seizure History: Primary Generalized Seizures and Unknown

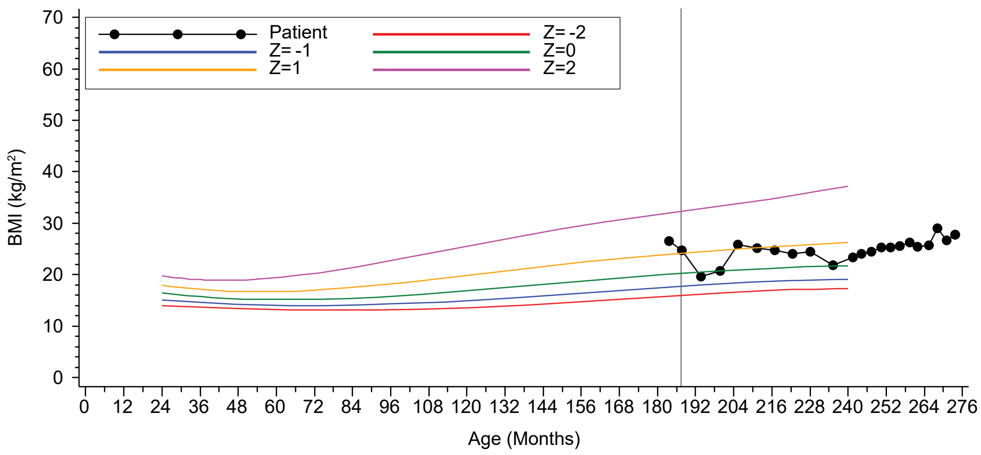

Patient 205

Seizure History: Primary Generalized Seizures and Unknown

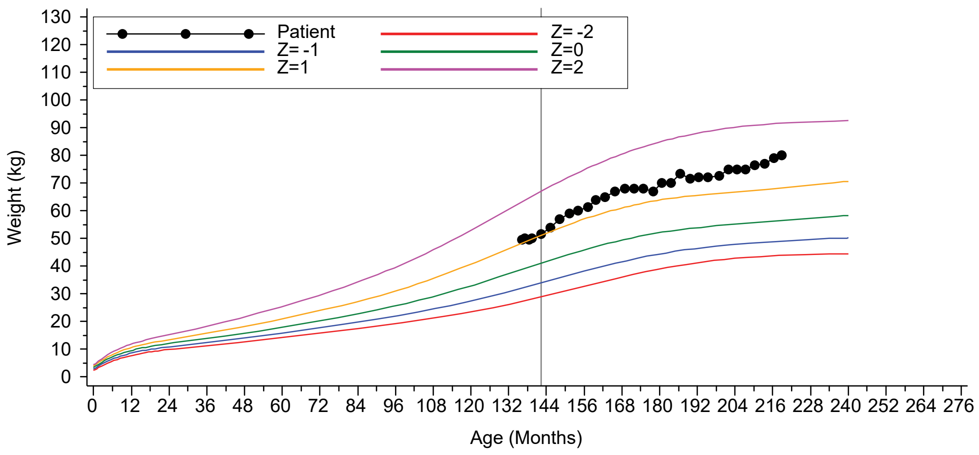

Patient 205

Seizure History: Primary Generalized Seizures and Unknown

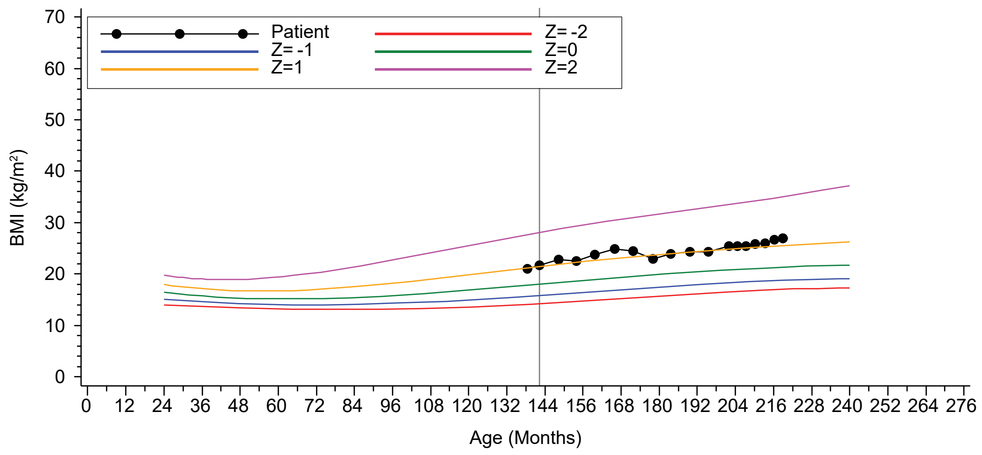

Patient 206

Seizure History: Primary Generalized Seizures and Unknown

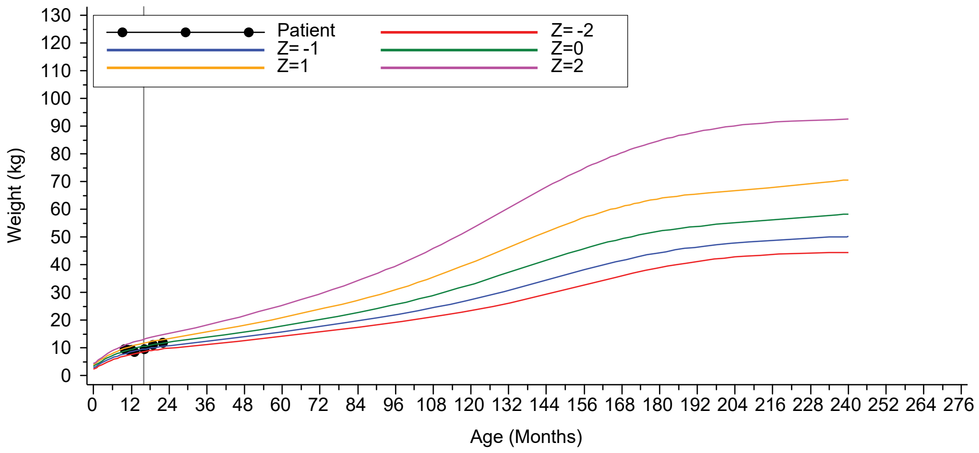

Patient 206

Seizure History: Primary Generalized Seizures and Unknown

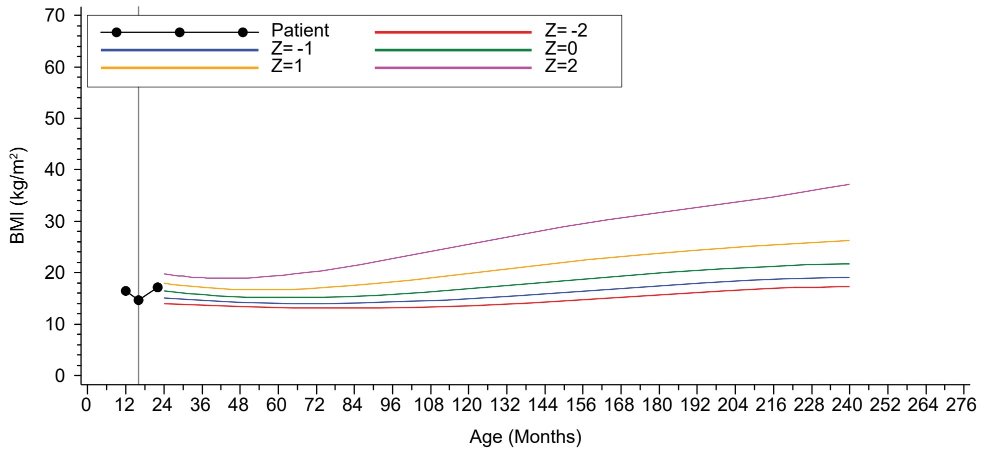

Patient 207

Seizure History: Primary Generalized Seizures and Unknown

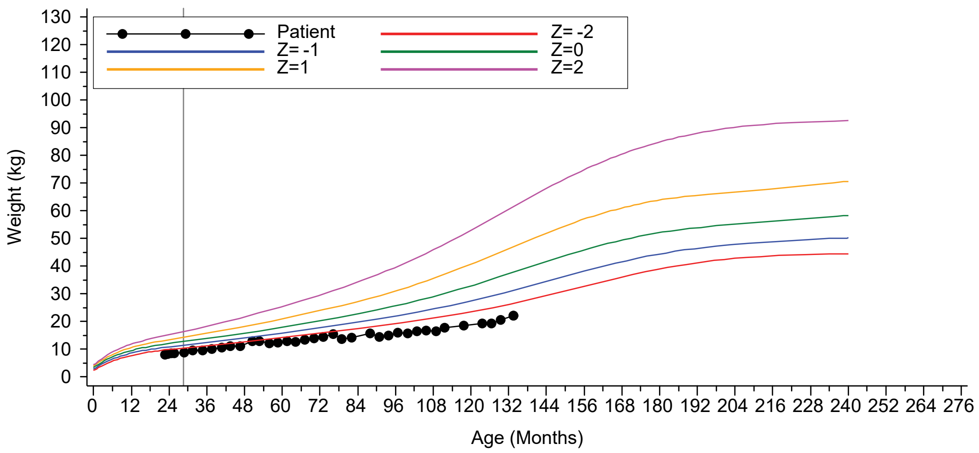

Patient 207

Seizure History: Primary Generalized Seizures and Unknown

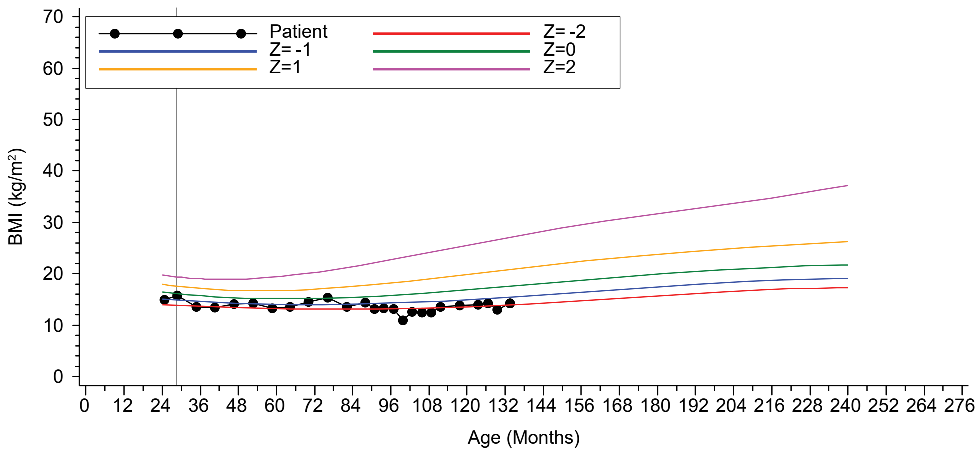

Patient 208

Seizure History: Primary Generalized Seizures and Unknown

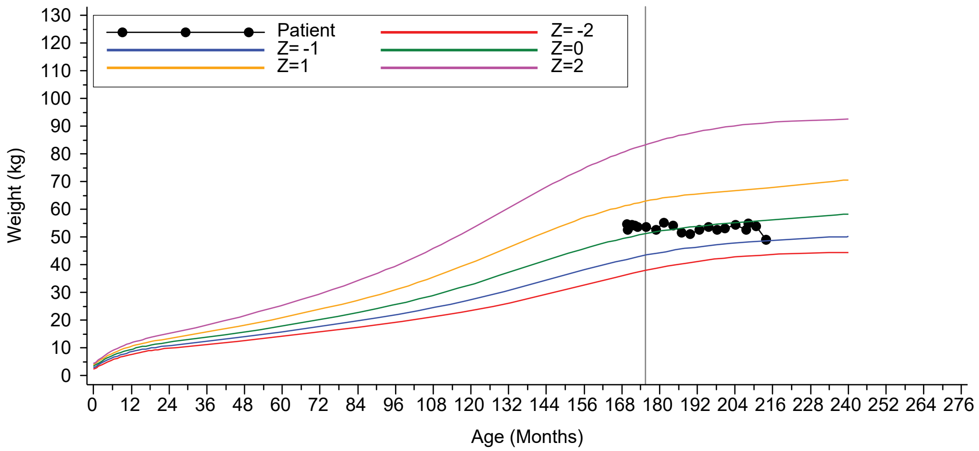

Patient 208

Seizure History: Primary Generalized Seizures and Unknown

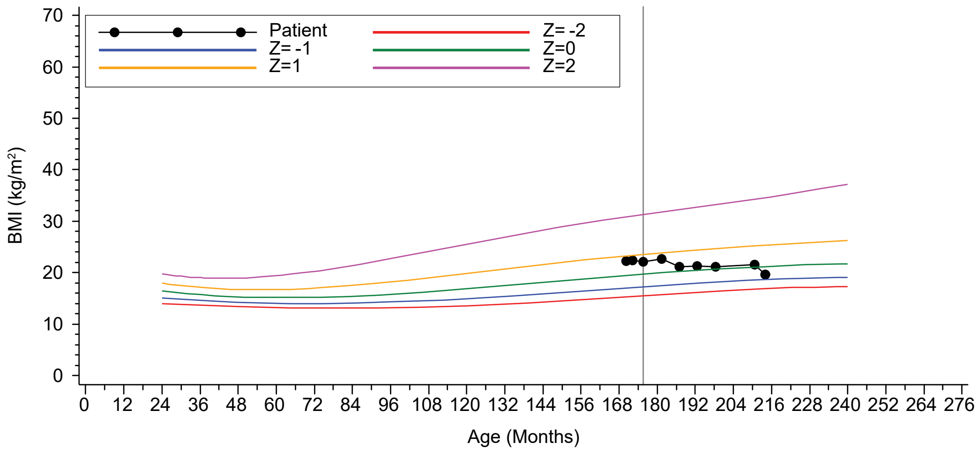

Patient 209

Seizure History: Primary Generalized Seizures and Unknown

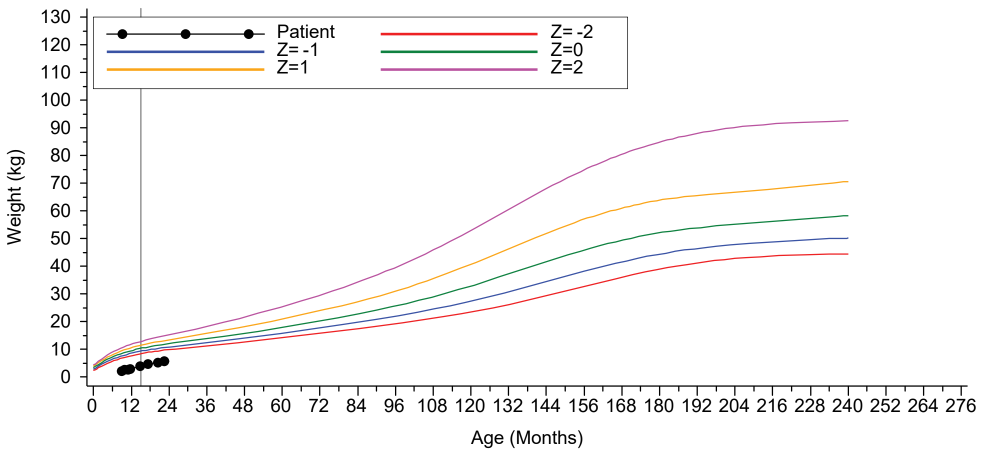

Patient 209

Seizure History: Primary Generalized Seizures and Unknown

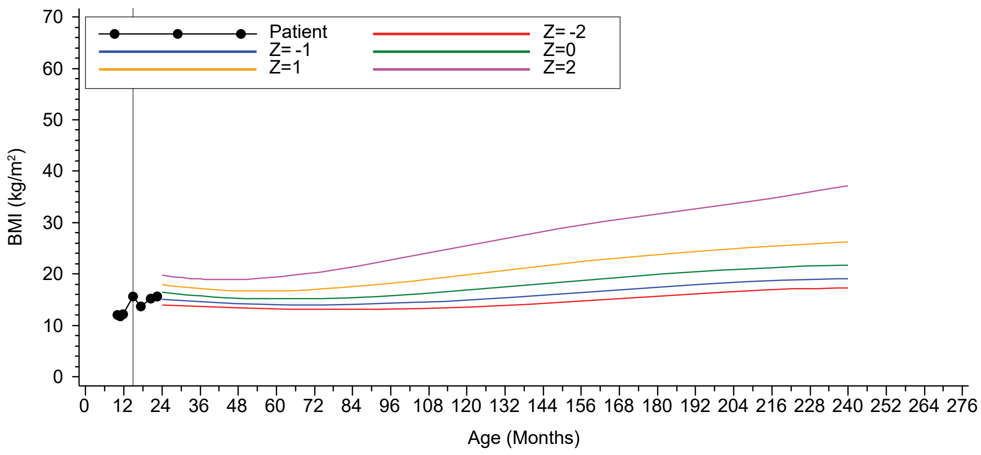

Supplement: Supplementary file 1 — Data S1. [file EPI4-9-2230-s003.pdf]
