# Supplementary material for: Treatment with brivaracetam has no apparent long‐term effects on body weight in pediatric patients with epilepsy
Source: Epilepsia Open. 2024 Oct 2;9(6):2230–40. doi: 10.1002/epi4.13045 (PMC11633704; doi:10.1002/epi4.13045)
Supplement: Supplementary file 2 — Data S2. [file EPI4-9-2230-s002.pdf]

Treatment with brivaracetam has no apparent long-term effects on body weight in pediatric patients with epilepsy

Florin I. Floricel | Paula E. Reichel | Najla Dickson | Sofia Fleyshman | Christoph Reichel | Jan-Peer Elshoff

Study inclusion and withdrawal criteria

- Female patients of childbearing age were required to be sexually inactive with a negative pregnancy test, or use adequate contraception.
- Planned trial participation was ≥3 years until pediatric approval of BRV for the patient’s age group was obtained, a managed access program was established, patients transitioned to another BRV trial, or the trial was terminated by sponsor.
- Patients were withdrawn from the trial if they developed an illness that, in the opinion of the Investigator, would have interfered with their continued participation or would potentially have been detrimental to their physical/mental health; took prohibited concomitant medications; became pregnant; or had an episode of convulsive status epilepticus, prolongation of seizure duration, worsening of seizure frequency, or emergence of a new seizure type, that was considered by the Investigator to require intervention.
