# Supplementary material for: Treatment with brivaracetam has no apparent long‐term effects on body weight in pediatric patients with epilepsy
Source: Epilepsia Open. 2024 Oct 2;9(6):2230–40. doi: 10.1002/epi4.13045 (PMC11633704; doi:10.1002/epi4.13045)
Supplement: Supplementary file 3 — Table S1. [file EPI4-9-2230-s001.pdf]

Treatment with brivaracetam has no apparent long-term effects on body weight in pediatric patients with epilepsy

Florin I. Floricel | Paula E. Reichel | Najla Dickson | Sofia Fleyshman | Christoph Reichel | Jan-Peer Elshoff

TABLE S1 Definitions of growth status for weight and BMI compared with equivalent percentiles and z-scores.[a]

Percentile	z-score	Weight	BMI
≥97th	≥2	Overweight	Obesity
85th to 97th	1 to 2	Normal	Overweight
3rd to 97th	–2 to 2	Normal	Normal
3rd to 16th	–2 to –1	At risk for underweight	At risk for underweight
<3rd	–2 to –3	Moderate underweight	Moderate underweight
<3rd	<–3	Severe malnutrition (underweight)	Severe malnutrition (wasting)

[a]Adapted from Table 3 of Sentongo, 2019.[1]

Abbreviations: BMI, body mass index.

[1]Sentongo T. A new approach to comprehensive growth and nutrition assessment in children. Pediatr Ann 2019;48(11):e425–e33.
